# Gene expression profiling reveals consistent differences between clinical samples of human leukaemias and their model cell lines

**DOI:** 10.1111/j.1365-2141.2006.06342.x

**Published:** 2006-11

**Authors:** Nicolas Leupin, Alexandre Kuhn, Barbara Hügli, Tobias J Grob, Rolf Jaggi, Andreas Tobler, Mauro Delorenzi, Martin F Fey

**Affiliations:** 1 Department of Medical Oncology, University of Berne Inselspital, Berne, Switzerland; 2 Department of Clinical Research, University of Berne Inselspital, Berne, Switzerland; 3 Swiss Institute of Bioinformatics Lausanne, Switzerland; 4 Department of Haematology, University of Berne Inselspital, Berne, Switzerland; 5 NCCR Molecular Oncology, Swiss Institute of Cancer Research Epalinges, Switzerland

**Keywords:** chronic myeloid leukaemia, acute myeloid leukaemia, BCR/ABL, cell lines, gene expression

## Abstract

Microarray gene expression profiles of fresh clinical samples of chronic myeloid leukaemia in chronic phase, acute promyelocytic leukaemia and acute monocytic leukaemia were compared with profiles from cell lines representing the corresponding types of leukaemia (K562, NB4, HL60). In a hierarchical clustering analysis, all clinical samples clustered separately from the cell lines, regardless of leukaemic subtype. Gene ontology analysis showed that cell lines chiefly overexpressed genes related to macromolecular metabolism, whereas in clinical samples genes related to the immune response were abundantly expressed. These findings must be taken into consideration when conclusions from cell line-based studies are extrapolated to patients.

In experimental cancer research, the study of clinical material, i.e. tumour biopsies, must often be complemented by *in vitro* experiments on cancer cell lines, as these enable functional molecular studies to be performed that would not be possible with biopsy material. In leukaemia research, cell lines, such as the BCR/ABL-positive K562 myeloid cell line, derived from a chronic myeloid leukaemia (CML) patient in blast crisis, or the leukaemic t(15;17)-positive NB4 cell line, derived from a patient with acute promyelocytic leukaemia (APL), are often used to study the molecular pathology of CML and APL respectively.

To extrapolate conclusions from cell line data to the clinical setting, it is crucial to determine how closely a given cell line and its molecular features resemble the respective clinical material. Such comparisons of leukaemic cell lines and patient samples can now be obtained with the help of the DNA microarray technology (Golub *et al*, 1999). In this study, we assessed the degree of resemblance of gene expression profiles between fresh clinical samples and the corresponding leukaemic cell lines.

## Patients and methods

### Patients and cell lines

We analysed peripheral white blood cell samples from six untreated patients with CML in chronic phase (all BCR/ABL-positive), as well as four patients with APL; acute myeloid leukaemia French–American–British (AML FAB) subtype M3; t(15;17)) and from four patients with acute monocytic leukaemia (AML FAB M5; no specific karyotypic abnormality). Informed consent was obtained from all patients. For comparison, the human myeloid BCR/ABL+ leukaemia cell line K562, the t(15;17)-positive NB4 cell line and the HL60 cell line, established from a patient with AML FAB subtype M2, were also analysed.

### Sample preparation

Preparation of Biotinylated cRNA and profiling with Human Genome U133 Gene Chips was performed according to standard protocols (Affymetrix, Santa Clara, CA, USA). Cell lines were analysed in duplicate, clinical samples were analysed with one chip per patient. The array images were quantified utilising Micro Array Suite (MAS) software (Affymetrix).

### Microarray quality control and normalisation

After visual inspection of each microarray scan for irregularities, the quality of the whole microarray set was assessed using the ‘affyPLM’ package from the Bioconductor project (Gentleman *et al*, 2004). Expression values were obtained after background subtraction (Irizarry *et al*, 2003), normalisation (Bolstad *et al*, 2003) and probe set summarisation (Irizarry *et al*, 2003) on a logarithmic (base 2) scale with the ‘affy’ package (Gautier *et al*, 2004).

### Data analysis

Hierarchical clustering analysis of expression profiles was performed using one minus Pearson's correlation coefficient as a measure of pairwise distance between samples and Ward's linkage as the agglomeration method. All 22’216 probe sets were used. The differential expression between fresh clinical samples and cell line samples was assessed using an empirical Bayes test statistic (Smyth, 2004) available through the ‘limma’ software package (Smyth *et al*, 2005). The obtained *P*-values were corrected for multiple testing using the False Discovery Rate method (Benjamini & Hochberg, 1995).

GOstat (Beissbarth & Speed, 2004) was used to perform a gene ontology analysis of differentially expressed genes. A separate analysis was carried out for the top 1000 up- and top 1000 downregulated genes.

## Results

**Fig 1:**
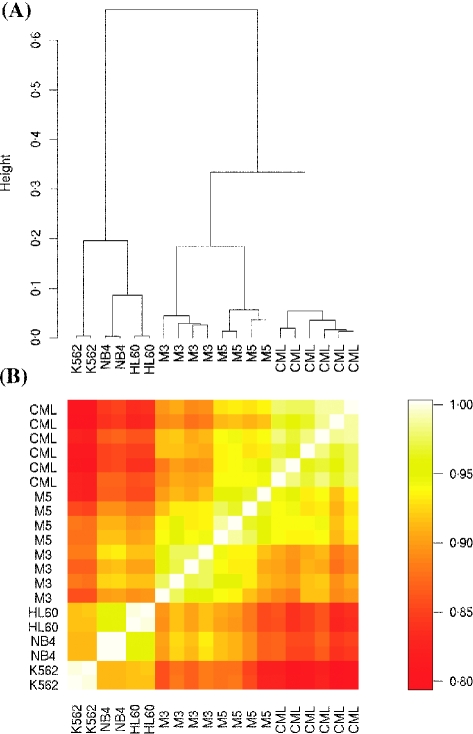
(A) Hierarchical clustering of samples was used to explore the similarities between expression profiles. The branch length represents the distance between two samples or two sample groups. (B) Colour-coded correlation matrix. The colour represents the Pearson's correlation coefficient of the gene expression profiles of each sample pair (scale on the right).

A hierarchical clustering analysis was performed to investigate the global similarity between the 20 expression profiles (Fig 1A). Remarkably, the main split in the dendrogram perfectly separated leukaemic cell lines from fresh patient samples. Cell lines clustered with a distinct common expression profile, and accordingly, the dendrogram united both fresh AML and CML samples in a separate common group. Specifically, the K562 and the NB4 cell lines did not cluster with the clinical samples bearing the same chromosomal translocation, i.e. with the CML and the APL samples respectively. In contrast, CML patient samples were clearly separated from fresh AML samples, which in turn clustered according to their morphological and biological features [APL (M3) or acute monocytic leukaemia (M5) respectively]. The correlation matrix (Fig 1B) visualises the pairwise similarity of all fresh patient samples and cell lines directly. Surprisingly, the K562 cell line showed a higher resemblance to AML samples than to CML samples.

Table I displays the top 24 probe sets ordered by decreasing evidence for differential expression between fresh samples and cell lines (See also Tables SI, SII, SIII). For example, the E2F6 gene was upregulated in cell lines compared with clinical samples. It belongs to a group of genes that have a pivotal role in the regulation of cellular proliferation by controlling the expression of genes that are essential for either entry into, or passage through, the cell cycle (Bell & Ryan, 2004).

**Table I:** Top 24 differentially expressed genes in cell lines (K562, NB4, HL60) compared with clinical samples (CML, APL, AML M5).

Rank	Probeset Id	Gene symbol	Log 2 fold change	Adjusted *P*-value	Gene title
1	203820_s_at	*IMP-3*	3·9	1·2 × 10^−15^	IGF-II mRNA-binding protein 3
2	209120_at	*NR2F2*	3·3	4·0 × 10^−14^	Nuclear receptor subfamily 2, group F, member 2
3	218976_at	*DNAJC12*	3·3	4·9 × 10^−13^	DnaJ (Hsp40) homologue, subfamily C, member 12
4	205194_at	*PSPH*	2·3	8·9 × 10^−12^	Phosphoserine phosphatase
5	219371_s_at	*KLF2*	−3·9	1·7 × 10^−11^	Kruppel-like factor 2 (lung)
6	209434_s_at	*PPAT*	2·1	2·1 × 10^−11^	Phosphoribosyl pyrophosphate amidotransferase
7	208961_s_at	*COPEB*	−3·5	2·9 × 10^−11^	Core promoter element binding protein
8	204228_at	*PPIH*	2·1	3·7 × 10^−11^	Peptidyl prolyl isomerase H
9	205394_at	*CHEK1*	2·1	5·2 × 10^−11^	CHK1 checkpoint homologue
10	214155_s_at	*LOC113251*	1·8	8·8 × 10^−11^	c-Mpl binding protein
11	213435_at	*SATB2*	2·7	8·8 × 10^−11^	SATB family member 2
12	219006_at	*C6orf66*	2·3	2·2 × 10^−10^	Chromosome 6 open reading frame 66
13	208763_s_at	*DSIPI*	−2·8	2·3 × 10^−10^	Delta sleep inducing peptide, immunoreactor
14	219479_at	*KDELC1*	1·9	7·2 × 10^−10^	KDEL (Lys-Asp-Glu-Leu) containing 1
15	203696_s_at	*RFC2*	1·5	7·2 × 10^−10^	Replication factor C (activator 1) 2,
16	209406_at	*BAG2*	3·0	1·1 × 10^−9^	BCL2-associated athanogene 2
17	209891_at	*Spc25*	2·0	1·1 × 10^−9^	Kinetochore protein Spc25
18	203281_s_at	*UBE1L*	−1·4	1·7 × 10^−9^	Ubiquitin-activating enzyme E1-like
19	204795_at	*PRR3*	1·3	2·3 × 10^−9^	Proline rich 3
20	209832_s_at	*CDT1*	3·0	2·3 × 10^−9^	DNA replication factor
21	222024_s_at	*AKAP13*	−2·7	4·3 × 10^−9^	A kinase (PRKA) anchor protein 13
22	209900_s_at	*SLC16A1*	2·7	4·3 × 10^−9^	Solute carrier family 16
23	203957_at	*E2F6*	1·6	4·5 × 10^−9^	E2F transcription factor 6
24	213320_at	*HRMT1L3*	1·8	4·6 × 10^−9^	HMT1 hnRNP methyltransferase-like 3

Positive (or negative) mean log_2_ fold change indicates upregulation (or downregulation) in cell lines compared with fresh samples (refer to Table S1. for the extensive gene list).

*P*-values were adjusted to account for multiple testing with a false discovery rate approach (Benjamini & Hochberg, 1995).

A gene ontology analysis of the top 1000 discriminatory genes showed that genes with an increased expression in cell lines were significantly related to macromolecular synthesis and nucleic acid metabolism. Genes with an increased expression in fresh patient samples, on the other hand, were significantly related to defence and immune response (see Tables SI, SII, SIII).

## Discussion

Much of our knowledge on the molecular functional pathways of human leukaemia is derived from experiments with cell lines rather than from work on clinical samples (Sandberg & Ernberg, 2005). In our present comparison we would have expected that, for example, BCR/ABL-positive leukaemias, i.e. the clinical material and the respective cell line, would primarily be allocated to a common gene expression profile group, and clearly be separated from BCR/ABL-negative leukaemias, given the strong impact of the *BCR/ABL* fusion gene in the molecular pathology of CML. However, we found that differences between leukaemia subtypes were dominated by stronger and consistent differences between cell lines and clinical samples. This observation indicates that the most important common denominator of cell lines at a molecular level are gene alterations linked to their immortalisation (an essential feature of any type of cancer cell line), which, in terms of gene expression, apparently overrule type-specific gene alterations, such as chromosomal translocations that define the respective clinical entities. The gene ontology analysis confirmed this hypothesis and showed that in cell lines, genes related to DNA or RNA metabolism and genes related to macromolecule synthesis are particularly active. In contrast, in clinical samples, genes related to immune or host response are overexpressed.

We believe that these observations must be taken into account when experimental data on the molecular pathology of leukaemia obtained from leukaemic cell lines are extrapolated to clinical samples, given the fundamental differences in gene expression profiles between the two groups.

## Supplementary material

The following supplementary material is available for this article online:

Table SI This Table lists all probe sets, ordered by decreasing evidence for differential expression between fresh samples (CML, AML M3, AML M5) and cell lines (K562, NB4, HL60). Positive log fold changes indicate upregulations in cell lines compared with fresh samples. When multiple probe sets were reporting for the same gene, only the most significant was kept.

Table SII GO analysis of top 1000 upregulated genes (overexpression in cell lines compared with clinical samples).

Table SIII GO analysis of top 1000 downregulated genes (underexpression in cell lines compared with clinical samples).

This material is available as part of the online article from http://www.blackwell-synergy.com
