# Supplementary material for: Gene expression profiling reveals consistent differences between clinical samples of human leukaemias and their model cell lines
Source: Br J Haematol. 2006 Nov;135(4):520–3. doi: 10.1111/j.1365-2141.2006.06342.x (PMC1654200; doi:10.1111/j.1365-2141.2006.06342.x)
Supplement: Table SI — This Table lists all probe sets, ordered by decreasing evidence for differential expression between fresh samples (CML, AML M3, AML M5) and cell lines (K562, NB4, HL60). Positive log fold changes indicate upregulations in cell lines compared with fresh samples. When multiple probe sets were reporting for the same gene, only the most significant was kept. [file bjh0135-0520-TableSI.html]

| rank | probeset ID | gene symbol | log 2 fold change | log 2 abundance | moderated t | unadjusted p-value | adjusted p-value | gene title | GO biological process |  |  |  |  |  |  |  |  |  |  |  |  |  |  |  |  |  |  |  |  |  |  |  |  |  |  |  |  |
| 1 | 203820\_s\_at | IMP-3 | 3.886233644 | 5.122345401 | 37.0598817 | 5.52E-020 | 1.23E-015 | IGF-II mRNA-binding protein 3 | RNA processing, morphogenesis, protein biosynthesis |  | | | | | | | | | | | | | | | | | | | | | | | | | | | |
| 2 | 209120\_at | NR2F2 | 3.340938105 | 4.813384345 | 29.3773182 | 5.43E-018 | 4.02E-014 | nuclear receptor subfamily 2, group F, member 2 | lipid metabolism, regulation of transcription from Pol II promoter, signal transduction |  | | | | | | | | | | | | | | | | | | | | | | | | | | | |
| 3 | 218976\_at | DNAJC12 | 3.288723256 | 6.732586351 | 25.4956283 | 8.74E-017 | 4.85E-013 | DnaJ (Hsp40) homolog, subfamily C, member 12 | protein folding |  | | | | | | | | | | | | | | | | | | | | | | | | | | | |
| 4 | 205194\_at | PSPH | 2.255786613 | 4.643839076 | 21.6969838 | 2.01E-015 | 8.92E-012 | phosphoserine phosphatase | L-serine biosynthesis, metabolism |  | | | | | | | | | | | | | | | | | | | | | | | | | | | |
| 5 | 219371\_s\_at | KLF2 | -3.898144121 | 9.329497052 | -20.796884 | 4.55E-015 | 1.68E-011 | Kruppel-like factor 2 (lung) | regulation of transcription, DNA-dependent |  | | | | | | | | | | | | | | | | | | | | | | | | | | | |
| 6 | 209434\_s\_at | PPAT | 2.148076532 | 6.02659989 | 20.4204758 | 6.46E-015 | 2.05E-011 | phosphoribosyl pyrophosphate amidotransferase | glutamine metabolism, metabolism, nucleoside metabolism, purine base biosynthesis, purine nucleotide biosynthesis |  | | | | | | | | | | | | | | | | | | | | | | | | | | | |
| 7 | 208961\_s\_at | COPEB | -3.537426122 | 10.15988705 | -19.9258256 | 1.03E-014 | 2.87E-011 | core promoter element binding protein | B-cell differentiation, cell growth, regulation of transcription, DNA-dependent |  | | | | | | | | | | | | | | | | | | | | | | | | | | | |
| 8 | 204228\_at | PPIH | 2.098563714 | 9.318600679 | 19.5495518 | 1.49E-014 | 3.68E-011 | peptidyl prolyl isomerase H (cyclophilin H) | nuclear mRNA splicing, via spliceosome, protein complex assembly, protein folding, snRNP protein-nucleus import |  | | | | | | | | | | | | | | | | | | | | | | | | | | | |
| 9 | 205394\_at | CHEK1 | 2.060130401 | 5.94086005 | 19.1031543 | 2.32E-014 | 5.15E-011 | CHK1 checkpoint homolog (S. pombe) | DNA damage checkpoint, cell cycle, gametogenesis, meiotic recombination, negative regulation of cell proliferation, protein amino acid phosphorylation, regulation of cyclin dependent protein kinase activity, response to DNA damage stimulus |  | | | | | | | | | | | | | | | | | | | | | | | | | | | |
| 10 | 214155\_s\_at | LOC113251 | 1.816351644 | 4.693722098 | 18.4811517 | 4.36E-014 | 8.81E-011 | c-Mpl binding protein | NA |  | | | | | | | | | | | | | | | | | | | | | | | | | | | |
| 11 | 213435\_at | SATB2 | 2.666472779 | 6.269920317 | 18.3957014 | 4.76E-014 | 8.82E-011 | SATB family member 2 | regulation of transcription, DNA-dependent |  | | | | | | | | | | | | | | | | | | | | | | | | | | | |
| 12 | 219006\_at | C6orf66 | 2.253559503 | 6.967349588 | 17.4733427 | 1.26E-013 | 2.16E-010 | chromosome 6 open reading frame 66 | NA |  | | | | | | | | | | | | | | | | | | | | | | | | | | | |
| 13 | 208763\_s\_at | DSIPI | -2.751465639 | 10.77006755 | -17.3615349 | 1.43E-013 | 2.27E-010 | delta sleep inducing peptide, immunoreactor | regulation of transcription, DNA-dependent |  | | | | | | | | | | | | | | | | | | | | | | | | | | | |
| 14 | 219479\_at | KDELC1 | 1.920866785 | 5.318408283 | 16.1641201 | 5.48E-013 | 7.21E-010 | KDEL (Lys-Asp-Glu-Leu) containing 1 | NA |  | | | | | | | | | | | | | | | | | | | | | | | | | | | |
| 15 | 203696\_s\_at | RFC2 | 1.493055374 | 7.863070527 | 16.158377 | 5.52E-013 | 7.21E-010 | replication factor C (activator 1) 2, 40kDa | DNA replication |  | | | | | | | | | | | | | | | | | | | | | | | | | | | |
| 16 | 209406\_at | BAG2 | 3.02020844 | 6.480705376 | 15.7461308 | 8.94E-013 | 1.10E-009 | BCL2-associated athanogene 2 | apoptosis, protein folding |  | | | | | | | | | | | | | | | | | | | | | | | | | | | |
| 17 | 209891\_at | Spc25 | 2.007218135 | 5.196900049 | 15.7053284 | 9.39E-013 | 1.10E-009 | kinetochore protein Spc25 | NA |  | | | | | | | | | | | | | | | | | | | | | | | | | | | |
| 18 | 203281\_s\_at | UBE1L | -1.395839613 | 8.500750722 | -15.3147403 | 1.50E-012 | 1.67E-009 | ubiquitin-activating enzyme E1-like | ubiquitin cycle |  | | | | | | | | | | | | | | | | | | | | | | | | | | | |
| 19 | 204795\_at | PRR3 | 1.269744362 | 7.777624211 | 14.9924413 | 2.23E-012 | 2.25E-009 | proline rich 3 | NA |  | | | | | | | | | | | | | | | | | | | | | | | | | | | |
| 20 | 209832\_s\_at | CDT1 | 2.981447902 | 7.816675483 | 14.9916021 | 2.23E-012 | 2.25E-009 | DNA replication factor | NA |  | | | | | | | | | | | | | | | | | | | | | | | | | | | |
| 21 | 222024\_s\_at | AKAP13 | -2.749057525 | 7.987525261 | -14.4068817 | 4.65E-012 | 4.34E-009 | A kinase (PRKA) anchor protein 13 | cell growth and/or maintenance, intracellular signaling cascade |  | | | | | | | | | | | | | | | | | | | | | | | | | | | |
| 22 | 209900\_s\_at | SLC16A1 | 2.678797251 | 5.819817726 | 14.3997445 | 4.69E-012 | 4.34E-009 | solute carrier family 16 (monocarboxylic acid transporters), member 1 | mevalonate transport, transport |  | | | | | | | | | | | | | | | | | | | | | | | | | | | |
| 23 | 203957\_at | E2F6 | 1.588083137 | 7.071117174 | 14.3475484 | 5.02E-012 | 4.46E-009 | E2F transcription factor 6 | negative regulation of transcription from Pol II promoter, regulation of cell cycle, regulation of transcription, DNA-dependent |  | | | | | | | | | | | | | | | | | | | | | | | | | | | |
| 24 | 213320\_at | HRMT1L3 | 1.793281109 | 6.276139048 | 14.3006137 | 5.33E-012 | 4.55E-009 | HMT1 hnRNP methyltransferase-like 3 (S. cerevisiae) | NA |  | | | | | | | | | | | | | | | | | | | | | | | | | | | |
| 25 | 213793\_s\_at | HOMER1 | 1.476893201 | 4.475935611 | 14.2074749 | 6.01E-012 | 4.94E-009 | homer homolog 1 (Drosophila) | NA |  | | | | | | | | | | | | | | | | | | | | | | | | | | | |
| 26 | 206643\_at | HAL | -3.23751484 | 7.240428522 | -14.1771982 | 6.25E-012 | 4.96E-009 | histidine ammonia-lyase | biosynthesis, histidine catabolism |  | | | | | | | | | | | | | | | | | | | | | | | | | | | |
| 27 | 203209\_at | RFC5 | 2.139320038 | 7.196036305 | 13.8835444 | 9.17E-012 | 6.39E-009 | replication factor C (activator 1) 5, 36.5kDa | DNA repair, DNA replication |  | | | | | | | | | | | | | | | | | | | | | | | | | | | |
| 28 | 201041\_s\_at | DUSP1 | -5.495922128 | 10.74386782 | -13.8807365 | 9.20E-012 | 6.39E-009 | dual specificity phosphatase 1 | cell cycle, protein amino acid dephosphorylation, response to oxidative stress |  | | | | | | | | | | | | | | | | | | | | | | | | | | | |
| 29 | 206103\_at | RAC3 | 1.232679561 | 6.301903781 | 13.7753229 | 1.06E-011 | 7.12E-009 | ras-related C3 botulinum toxin substrate 3 (rho family, small GTP binding protein Rac3) | protein transport, small GTPase mediated signal transduction |  | | | | | | | | | | | | | | | | | | | | | | | | | | | |
| 30 | 211528\_x\_at | HLA-G | -2.278649572 | 11.79004876 | -13.7336264 | 1.12E-011 | 7.24E-009 | HLA-G histocompatibility antigen, class I, G | antigen presentation, endogenous antigen, antigen processing, endogenous antigen via MHC class I, cellular defense response, detection of pest, pathogen or parasite |  | | | | | | | | | | | | | | | | | | | | | | | | | | | |
| 31 | 200797\_s\_at | MCL1 | -2.095791841 | 11.49204247 | -13.7182914 | 1.14E-011 | 7.24E-009 | myeloid cell leukemia sequence 1 (BCL2-related) | anti-apoptosis, apoptotic program, cell differentiation, cell fate determination, cell homeostasis, regulation of apoptosis |  | | | | | | | | | | | | | | | | | | | | | | | | | | | |
| 32 | 215313\_x\_at | HLA-A | -3.741747106 | 12.16879388 | -13.6409499 | 1.26E-011 | 7.81E-009 | major histocompatibility complex, class I, A | antigen presentation, endogenous antigen, antigen processing, endogenous antigen via MHC class I, immune response |  | | | | | | | | | | | | | | | | | | | | | | | | | | | |
| 33 | 219188\_s\_at | LRP16 | 1.855987357 | 6.516332975 | 13.5907854 | 1.35E-011 | 8.12E-009 | LRP16 protein | NA |  | | | | | | | | | | | | | | | | | | | | | | | | | | | |
| 34 | 212709\_at | NUP160 | 2.394883615 | 5.275389023 | 13.5484092 | 1.43E-011 | 8.37E-009 | nucleoporin 160kDa | mRNA-nucleus export, transport |  | | | | | | | | | | | | | | | | | | | | | | | | | | | |
| 35 | 202290\_at | PDAP1 | 1.498148431 | 8.462210596 | 13.4999909 | 1.53E-011 | 8.70E-009 | PDGFA associated protein 1 | cell proliferation, signal transduction |  | | | | | | | | | | | | | | | | | | | | | | | | | | | |
| 36 | 218597\_s\_at | C10orf70 | 2.221823544 | 7.07598772 | 13.4182494 | 1.71E-011 | 9.25E-009 | chromosome 10 open reading frame 70 | NA |  | | | | | | | | | | | | | | | | | | | | | | | | | | | |
| 37 | 210005\_at | GART | 1.507535049 | 5.765207534 | 13.380096 | 1.80E-011 | 9.51E-009 | phosphoribosylglycinamide formyltransferase, phosphoribosylglycinamide synthetase, phosphoribosylaminoimidazole synthetase | de novo' IMP biosynthesis, biosynthesis, purine base biosynthesis, purine nucleotide biosynthesis |  | | | | | | | | | | | | | | | | | | | | | | | | | | | |
| 38 | 213149\_at | DLAT | 2.097818597 | 6.438800414 | 13.1897618 | 2.33E-011 | 1.18E-008 | dihydrolipoamide S-acetyltransferase (E2 component of pyruvate dehydrogenase complex) | acetyl-CoA biosynthesis, glycolysis, metabolism |  | | | | | | | | | | | | | | | | | | | | | | | | | | | |
| 39 | 219119\_at | LSM8 | 1.934852129 | 8.557683708 | 13.1437359 | 2.48E-011 | 1.23E-008 | LSM8 homolog, U6 small nuclear RNA associated (S. cerevisiae) | nuclear mRNA splicing, via spliceosome |  | | | | | | | | | | | | | | | | | | | | | | | | | | | |
| 40 | 218397\_at | FANCL | 1.716702082 | 6.049880492 | 13.0752699 | 2.73E-011 | 1.32E-008 | Fanconi anemia, complementation group L | DNA repair, ubiquitin cycle |  | | | | | | | | | | | | | | | | | | | | | | | | | | | |
| 41 | 208336\_s\_at | GPSN2 | 1.337244179 | 8.611403501 | 13.0507078 | 2.82E-011 | 1.32E-008 | glycoprotein, synaptic 2 | NA |  | | | | | | | | | | | | | | | | | | | | | | | | | | | |
| 42 | 200995\_at | IPO7 | 2.42960498 | 6.409552488 | 13.0446434 | 2.85E-011 | 1.32E-008 | Importin 7 | protein transport, protein-nucleus import, docking, signal transduction |  | | | | | | | | | | | | | | | | | | | | | | | | | | | |
| 43 | 210172\_at | SF1 | -3.230122919 | 8.900867984 | -12.973314 | 3.14E-011 | 1.43E-008 | splicing factor 1 | nuclear mRNA splicing, via spliceosome, regulation of transcription, DNA-dependent |  | | | | | | | | | | | | | | | | | | | | | | | | | | | |
| 44 | 217436\_x\_at | --- | -2.281722038 | 10.37945712 | -12.8981607 | 3.49E-011 | 1.55E-008 | --- | NA |  | | | | | | | | | | | | | | | | | | | | | | | | | | | |
| 45 | 200903\_s\_at | AHCY | 2.302013189 | 9.027930584 | 12.7609767 | 4.23E-011 | 1.72E-008 | S-adenosylhomocysteine hydrolase | one-carbon compound metabolism |  | | | | | | | | | | | | | | | | | | | | | | | | | | | |
| 46 | 205085\_at | ORC1L | 2.210205022 | 6.566685151 | 12.7565667 | 4.26E-011 | 1.72E-008 | origin recognition complex, subunit 1-like (yeast) | DNA replication, DNA replication initiation |  | | | | | | | | | | | | | | | | | | | | | | | | | | | |
| 47 | 214452\_at | BCAT1 | 3.830474226 | 5.752525951 | 12.6395405 | 5.03E-011 | 1.99E-008 | branched chain aminotransferase 1, cytosolic | G1/S transition of mitotic cell cycle, branched chain family amino acid biosynthesis, cell proliferation, metabolism |  | | | | | | | | | | | | | | | | | | | | | | | | | | | |
| 48 | 221606\_s\_at | NSBP1 | 1.628390207 | 4.910158003 | 12.5485902 | 5.72E-011 | 2.23E-008 | nucleosomal binding protein 1 | regulation of transcription, DNA-dependent |  | | | | | | | | | | | | | | | | | | | | | | | | | | | |
| 49 | 208445\_s\_at | BAZ1B | 1.209662546 | 8.011774865 | 12.5370682 | 5.82E-011 | 2.23E-008 | bromodomain adjacent to zinc finger domain, 1B | protein ubiquitination, regulation of transcription, DNA-dependent, transcription |  | | | | | | | | | | | | | | | | | | | | | | | | | | | |
| 50 | 204353\_s\_at | POT1 | 1.379851197 | 7.043247232 | 12.306332 | 8.11E-011 | 3.05E-008 | POT1 protection of telomeres 1 homolog (S. pombe) | telomerase-dependent telomere maintenance |  | | | | | | | | | | | | | | | | | | | | | | | | | | | |
| 51 | 218073\_s\_at | FLJ10407 | 2.855177153 | 6.155789295 | 12.2933386 | 8.27E-011 | 3.06E-008 | hypothetical protein FLJ10407 | NA |  | | | | | | | | | | | | | | | | | | | | | | | | | | | |
| 52 | 220011\_at | MGC2603 | 1.330997765 | 7.016400805 | 12.2394935 | 8.94E-011 | 3.26E-008 | hypothetical protein MGC2603 | NA |  | | | | | | | | | | | | | | | | | | | | | | | | | | | |
| 53 | 214550\_s\_at | TNPO3 | 1.080100076 | 8.682365728 | 12.1642664 | 9.98E-011 | 3.57E-008 | transportin 3 | protein transport |  | | | | | | | | | | | | | | | | | | | | | | | | | | | |
| 54 | 211754\_s\_at | SLC25A17 | 1.695051472 | 6.571731714 | 12.1380293 | 1.04E-010 | 3.57E-008 | solute carrier family 25 (mitochondrial carrier; peroxisomal membrane protein, 34kDa), member 17 /// solute carrier family 25 (mitochondrial carrier; peroxisomal membrane protein, 34kDa), member 17 | mitochondrial transport, transport |  | | | | | | | | | | | | | | | | | | | | | | | | | | | |
| 55 | 209681\_at | SLC19A2 | 2.059955479 | 5.053813072 | 12.1335066 | 1.04E-010 | 3.57E-008 | solute carrier family 19 (thiamine transporter), member 2 | DNA replication, perception of sound, thiamin transport, transport |  | | | | | | | | | | | | | | | | | | | | | | | | | | | |
| 56 | 219162\_s\_at | MRPL11 | 1.386021576 | 8.631645226 | 12.1193914 | 1.07E-010 | 3.58E-008 | mitochondrial ribosomal protein L11 | protein biosynthesis |  | | | | | | | | | | | | | | | | | | | | | | | | | | | |
| 57 | 52285\_f\_at | C18orf9 | 2.068366531 | 5.024521062 | 12.1097679 | 1.08E-010 | 3.58E-008 | chromosome 18 open reading frame 9 | NA |  | | | | | | | | | | | | | | | | | | | | | | | | | | | |
| 58 | 205004\_at | NKRF | 1.013541255 | 6.759968552 | 12.040401 | 1.20E-010 | 3.91E-008 | NF-kappa B repressing factor | regulation of transcription, DNA-dependent |  | | | | | | | | | | | | | | | | | | | | | | | | | | | |
| 59 | 212282\_at | MAC30 | 3.215729368 | 8.437208609 | 12.0288207 | 1.22E-010 | 3.92E-008 | hypothetical protein MAC30 | regulation of cell growth |  | | | | | | | | | | | | | | | | | | | | | | | | | | | |
| 60 | 203967\_at | CDC6 | 2.162831566 | 5.731041027 | 12.0184479 | 1.24E-010 | 3.92E-008 | CDC6 cell division cycle 6 homolog (S. cerevisiae) | DNA replication, DNA replication checkpoint, cell cycle, cytokinesis, mitosis, negative regulation of DNA replication, negative regulation of cell proliferation, regulation of cyclin dependent protein kinase activity, traversing start control point of mitotic cell cycle |  | | | | | | | | | | | | | | | | | | | | | | | | | | | |
| 61 | 202737\_s\_at | LSM4 | 1.416920255 | 8.74890258 | 11.9975132 | 1.28E-010 | 3.99E-008 | LSM4 homolog, U6 small nuclear RNA associated (S. cerevisiae) | RNA splicing, nuclear mRNA splicing, via spliceosome |  | | | | | | | | | | | | | | | | | | | | | | | | | | | |
| 62 | 209189\_at | FOS | -4.875165142 | 9.934595224 | -11.9775636 | 1.31E-010 | 4.03E-008 | v-fos FBJ murine osteosarcoma viral oncogene homolog | DNA methylation, cell growth and/or maintenance, inflammatory response, regulation of transcription from Pol II promoter |  | | | | | | | | | | | | | | | | | | | | | | | | | | | |
| 63 | 202904\_s\_at | LSM5 | 2.009973933 | 4.428742512 | 11.9715917 | 1.33E-010 | 4.03E-008 | LSM5 homolog, U6 small nuclear RNA associated (S. cerevisiae) | nuclear mRNA splicing, via spliceosome |  | | | | | | | | | | | | | | | | | | | | | | | | | | | |
| 64 | 217829\_s\_at | USP39 | 1.062827177 | 8.232450529 | 11.9617262 | 1.34E-010 | 4.04E-008 | ubiquitin specific protease 39 | RNA splicing, mRNA processing, spliceosome assembly, ubiquitin-dependent protein catabolism |  | | | | | | | | | | | | | | | | | | | | | | | | | | | |
| 65 | 201121\_s\_at | PGRMC1 | 1.078772356 | 8.503045424 | 11.9300301 | 1.41E-010 | 4.18E-008 | progesterone receptor membrane component 1 | NA |  | | | | | | | | | | | | | | | | | | | | | | | | | | | |
| 66 | 203362\_s\_at | MAD2L1 | 3.000927458 | 6.797677416 | 11.8825765 | 1.51E-010 | 4.42E-008 | MAD2 mitotic arrest deficient-like 1 (yeast) | cell cycle, mitosis, mitotic checkpoint |  | | | | | | | | | | | | | | | | | | | | | | | | | | | |
| 67 | 204806\_x\_at | HLA-F | -2.839484189 | 10.72977784 | -11.8111393 | 1.68E-010 | 4.86E-008 | major histocompatibility complex, class I, F | antigen presentation, endogenous antigen, antigen processing, endogenous antigen via MHC class I, immune response |  | | | | | | | | | | | | | | | | | | | | | | | | | | | |
| 68 | 203092\_at | TIMM44 | 1.250242909 | 7.151213736 | 11.7958828 | 1.72E-010 | 4.90E-008 | translocase of inner mitochondrial membrane 44 homolog (yeast) | protein-mitochondrial targeting, regulation of transcription, DNA-dependent |  | | | | | | | | | | | | | | | | | | | | | | | | | | | |
| 69 | 212099\_at | RHOB | -3.832814089 | 9.290433165 | -11.7792514 | 1.77E-010 | 4.93E-008 | ras homolog gene family, member B | angiogenesis, cell adhesion, cell cycle, endosome to lysosome transport, negative regulation of cell cycle, positive regulation of angiogenesis, programmed cell death, transformed cells, protein transport |  | | | | | | | | | | | | | | | | | | | | | | | | | | | |
| 70 | 203380\_x\_at | SFRS5 | -1.616092678 | 10.34233396 | -11.7752415 | 1.78E-010 | 4.93E-008 | splicing factor, arginine/serine-rich 5 | mRNA splice site selection, nuclear mRNA splicing, via spliceosome |  | | | | | | | | | | | | | | | | | | | | | | | | | | | |
| 71 | 202710\_at | BET1 | 1.490482234 | 6.752367364 | 11.7181432 | 1.94E-010 | 5.31E-008 | BET1 homolog (S. cerevisiae) | ER to Golgi transport, protein transport |  | | | | | | | | | | | | | | | | | | | | | | | | | | | |
| 72 | 203416\_at | CD53 | -1.889504755 | 10.83956211 | -11.6953794 | 2.00E-010 | 5.43E-008 | CD53 antigen | antimicrobial humoral response (sensu Vertebrata), signal transduction |  | | | | | | | | | | | | | | | | | | | | | | | | | | | |
| 73 | 218903\_s\_at | MGC2731 | 1.162516682 | 6.962339798 | 11.667109 | 2.09E-010 | 5.60E-008 | hypothetical protein MGC2731 | NA |  | | | | | | | | | | | | | | | | | | | | | | | | | | | |
| 74 | 208660\_at | CS | 1.043730997 | 10.1425355 | 11.6342203 | 2.20E-010 | 5.74E-008 | citrate synthase | tricarboxylic acid cycle |  | | | | | | | | | | | | | | | | | | | | | | | | | | | |
| 75 | 212247\_at | NUP205 | 1.953076219 | 7.478990554 | 11.5535018 | 2.48E-010 | 6.42E-008 | nucleoporin 205kDa | nucleocytoplasmic transport, protein-nucleus import, docking, transport |  | | | | | | | | | | | | | | | | | | | | | | | | | | | |
| 76 | 217942\_at | MRPS35 | 2.282789038 | 8.891856168 | 11.5320382 | 2.57E-010 | 6.53E-008 | mitochondrial ribosomal protein S35 | NA |  | | | | | | | | | | | | | | | | | | | | | | | | | | | |
| 77 | 220417\_s\_at | THAP4 | 1.182457745 | 7.802556538 | 11.5265966 | 2.59E-010 | 6.53E-008 | THAP domain containing 4 | NA |  | | | | | | | | | | | | | | | | | | | | | | | | | | | |
| 78 | 211799\_x\_at | HLA-A /// HLA-C | -3.207335177 | 10.4819074 | -11.4822023 | 2.77E-010 | 6.84E-008 | major histocompatibility complex, class I, A /// major histocompatibility complex, class I, C | antigen presentation, endogenous antigen, antigen processing, endogenous antigen via MHC class I, immune response, immune response |  | | | | | | | | | | | | | | | | | | | | | | | | | | | |
| 79 | 201996\_s\_at | SHARP | -1.312666277 | 8.550522383 | -11.4354153 | 2.98E-010 | 7.21E-008 | SMART/HDAC1 associated repressor protein | regulation of transcription, DNA-dependent |  | | | | | | | | | | | | | | | | | | | | | | | | | | | |
| 80 | 203867\_s\_at | FLJ10458 | 1.701452724 | 7.745451918 | 11.4328822 | 2.99E-010 | 7.21E-008 | Notchless gene homolog (Drosophila) | G-protein coupled receptor protein signaling pathway |  | | | | | | | | | | | | | | | | | | | | | | | | | | | |
| 81 | 220083\_x\_at | UCHL5 | 1.829365386 | 5.298075712 | 11.4140294 | 3.07E-010 | 7.34E-008 | ubiquitin carboxyl-terminal hydrolase L5 | ubiquitin cycle, ubiquitin-dependent protein catabolism |  | | | | | | | | | | | | | | | | | | | | | | | | | | | |
| 82 | 208424\_s\_at | CIAPIN1 | 1.481964041 | 7.323183636 | 11.3710546 | 3.28E-010 | 7.76E-008 | cytokine induced apoptosis inhibitor 1 | NA |  | | | | | | | | | | | | | | | | | | | | | | | | | | | |
| 83 | 213590\_at | SLC16A5 | -0.877522689 | 7.158806282 | -11.3637928 | 3.32E-010 | 7.77E-008 | solute carrier family 16 (monocarboxylic acid transporters), member 5 | monocarboxylic acid transport, transport |  | | | | | | | | | | | | | | | | | | | | | | | | | | | |
| 84 | 204957\_at | ORC5L | 2.19315415 | 6.990331371 | 11.3190318 | 3.56E-010 | 8.24E-008 | origin recognition complex, subunit 5-like (yeast) | DNA replication, DNA replication initiation |  | | | | | | | | | | | | | | | | | | | | | | | | | | | |
| 85 | 204717\_s\_at | SLC29A2 | 1.498459344 | 5.625937929 | 11.3049663 | 3.64E-010 | 8.25E-008 | solute carrier family 29 (nucleoside transporters), member 2 | cell proliferation, nucleobase, nucleoside, nucleotide and nucleic acid metabolism, nucleoside transport, transport |  | | | | | | | | | | | | | | | | | | | | | | | | | | | |
| 86 | 218104\_at | TEX10 | 1.408312321 | 8.050265541 | 11.205543 | 4.24E-010 | 9.52E-008 | testis expressed sequence 10 | NA |  | | | | | | | | | | | | | | | | | | | | | | | | | | | |
| 87 | 210093\_s\_at | MAGOH | 1.47477429 | 6.969886897 | 11.1927913 | 4.33E-010 | 9.58E-008 | mago-nashi homolog, proliferation-associated (Drosophila) | sex determination |  | | | | | | | | | | | | | | | | | | | | | | | | | | | |
| 88 | 219000\_s\_at | DCC1 | 1.087432267 | 5.819652021 | 11.1687637 | 4.50E-010 | 9.58E-008 | defective in sister chromatid cohesion homolog 1 (S. cerevisiae) | NA |  | | | | | | | | | | | | | | | | | | | | | | | | | | | |
| 89 | 218154\_at | GSDMDC1 | -1.031454914 | 8.921552122 | -11.1661805 | 4.51E-010 | 9.58E-008 | gasdermin domain containing 1 | NA |  | | | | | | | | | | | | | | | | | | | | | | | | | | | |
| 90 | 218757\_s\_at | UPF3B | 1.700288588 | 8.049201359 | 11.1653668 | 4.52E-010 | 9.58E-008 | UPF3 regulator of nonsense transcripts homolog B (yeast) | mRNA catabolism, nonsense-mediated decay |  | | | | | | | | | | | | | | | | | | | | | | | | | | | |
| 91 | 220060\_s\_at | FLJ20641 | 1.484386457 | 5.662442454 | 11.1643288 | 4.53E-010 | 9.58E-008 | hypothetical protein FLJ20641 | NA |  | | | | | | | | | | | | | | | | | | | | | | | | | | | |
| 92 | 213578\_at | BMPR1A | 1.163422236 | 4.225489696 | 11.1145901 | 4.89E-010 | 1.03E-007 | bone morphogenetic protein receptor, type IA | protein amino acid phosphorylation, transforming growth factor beta receptor signaling pathway |  | | | | | | | | | | | | | | | | | | | | | | | | | | | |
| 93 | 203517\_at | MTX2 | 2.183983971 | 7.201528324 | 11.0654172 | 5.29E-010 | 1.09E-007 | metaxin 2 | mitochondrial transport, protein transport |  | | | | | | | | | | | | | | | | | | | | | | | | | | | |
| 94 | 210715\_s\_at | SPINT2 | -2.53060389 | 8.400346621 | -11.0358117 | 5.54E-010 | 1.13E-007 | serine protease inhibitor, Kunitz type, 2 | cell motility |  | | | | | | | | | | | | | | | | | | | | | | | | | | | |
| 95 | 202423\_at | MYST3 | -0.886320326 | 9.169292099 | -10.9934837 | 5.92E-010 | 1.19E-007 | MYST histone acetyltransferase (monocytic leukemia) 3 | DNA packaging, cell growth and/or maintenance, nucleosome assembly, protein ubiquitination, regulation of transcription, DNA-dependent |  | | | | | | | | | | | | | | | | | | | | | | | | | | | |
| 96 | 214662\_at | WDR43 | 1.847847546 | 6.155760896 | 10.9914756 | 5.94E-010 | 1.19E-007 | WD repeat domain 43 | NA |  | | | | | | | | | | | | | | | | | | | | | | | | | | | |
| 97 | 201436\_at | EIF4E | 1.258479939 | 4.767700384 | 10.9738661 | 6.11E-010 | 1.21E-007 | eukaryotic translation initiation factor 4E | regulation of translation, translational initiation |  | | | | | | | | | | | | | | | | | | | | | | | | | | | |
| 98 | 211953\_s\_at | RANBP5 | 2.719232025 | 6.69392399 | 10.9533035 | 6.31E-010 | 1.24E-007 | RAN binding protein 5 | NLS-bearing substrate-nucleus import, protein transport, protein-nucleus import, docking |  | | | | | | | | | | | | | | | | | | | | | | | | | | | |
| 99 | 210314\_x\_at | TNFSF13 /// TNFSF12-TNFSF13 | -3.26075649 | 8.51924098 | -10.9378635 | 6.46E-010 | 1.26E-007 | tumor necrosis factor (ligand) superfamily, member 13 /// tumor necrosis factor (ligand) superfamily, member 12-member 13 | immune response, positive regulation of cell proliferation, signal transduction |  | | | | | | | | | | | | | | | | | | | | | | | | | | | |
| 100 | 215509\_s\_at | BUB1 | 2.168444498 | 6.189065268 | 10.9287957 | 6.56E-010 | 1.26E-007 | BUB1 budding uninhibited by benzimidazoles 1 homolog (yeast) | cell cycle, mitosis, mitotic spindle checkpoint, protein amino acid phosphorylation |  | | | | | | | | | | | | | | | | | | | | | | | | | | | |
| 101 | 218889\_at | C10orf117 | 2.504821645 | 6.82675753 | 10.9248213 | 6.60E-010 | 1.26E-007 | chromosome 10 open reading frame 117 | NA |  | | | | | | | | | | | | | | | | | | | | | | | | | | | |
| 102 | 205063\_at | SIP1 | 1.295598437 | 4.314030524 | 10.8962346 | 6.91E-010 | 1.31E-007 | survival of motor neuron protein interacting protein 1 | spliceosome assembly |  | | | | | | | | | | | | | | | | | | | | | | | | | | | |
| 103 | 206613\_s\_at | TAF1A | 1.249136087 | 4.587609683 | 10.8805583 | 7.08E-010 | 1.32E-007 | TATA box binding protein (TBP)-associated factor, RNA polymerase I, A, 48kDa | transcription from Pol I promoter, transcription from Pol II promoter |  | | | | | | | | | | | | | | | | | | | | | | | | | | | |
| 104 | 209440\_at | PRPS1 | 1.433772487 | 8.745175981 | 10.8654603 | 7.25E-010 | 1.34E-007 | phosphoribosyl pyrophosphate synthetase 1 | nucleoside metabolism, nucleotide biosynthesis, ribonucleoside monophosphate biosynthesis |  | | | | | | | | | | | | | | | | | | | | | | | | | | | |
| 105 | 204446\_s\_at | ALOX5 | -4.963110064 | 8.903928177 | -10.8356984 | 7.61E-010 | 1.40E-007 | arachidonate 5-lipoxygenase | electron transport, inflammatory response, leukotriene biosynthesis |  | | | | | | | | | | | | | | | | | | | | | | | | | | | |
| 106 | 220942\_x\_at | E2IG5 | 1.035640084 | 9.239335382 | 10.8181293 | 7.82E-010 | 1.42E-007 | growth and transformation-dependent protein | NA |  | | | | | | | | | | | | | | | | | | | | | | | | | | | |
| 107 | 218225\_at | SITPEC | 1.204880619 | 7.10180145 | 10.7914434 | 8.16E-010 | 1.47E-007 | likely ortholog of mouse signaling intermediate in Toll pathway-evolutionarily conserved | NA |  | | | | | | | | | | | | | | | | | | | | | | | | | | | |
| 108 | 203436\_at | RPP30 | 0.932604253 | 8.205874091 | 10.7436764 | 8.81E-010 | 1.54E-007 | ribonuclease P/MRP 30kDa subunit | tRNA processing |  | | | | | | | | | | | | | | | | | | | | | | | | | | | |
| 109 | 201955\_at | CCNC | 2.272347606 | 8.642817345 | 10.7410282 | 8.85E-010 | 1.54E-007 | cyclin C | cytokinesis, regulation of cell cycle, regulation of transcription, DNA-dependent |  | | | | | | | | | | | | | | | | | | | | | | | | | | | |
| 110 | 211708\_s\_at | SCD | 2.030017461 | 7.777955329 | 10.7403616 | 8.86E-010 | 1.54E-007 | stearoyl-CoA desaturase (delta-9-desaturase) /// stearoyl-CoA desaturase (delta-9-desaturase) | fatty acid biosynthesis |  | | | | | | | | | | | | | | | | | | | | | | | | | | | |
| 111 | 203405\_at | DSCR2 | 2.349207617 | 7.783754351 | 10.7278604 | 9.04E-010 | 1.54E-007 | Down syndrome critical region gene 2 | NA |  | | | | | | | | | | | | | | | | | | | | | | | | | | | |
| 112 | 202181\_at | KIAA0247 | -1.856424673 | 8.579575693 | -10.7057955 | 9.37E-010 | 1.58E-007 | KIAA0247 | NA |  | | | | | | | | | | | | | | | | | | | | | | | | | | | |
| 113 | 212291\_at | HIPK1 | -1.872364072 | 8.976034191 | -10.7012357 | 9.44E-010 | 1.58E-007 | homeodomain interacting protein kinase 1 | protein amino acid phosphorylation |  | | | | | | | | | | | | | | | | | | | | | | | | | | | |
| 114 | 205963\_s\_at | DNAJA3 | 1.217915737 | 8.467562732 | 10.7000736 | 9.45E-010 | 1.58E-007 | DnaJ (Hsp40) homolog, subfamily A, member 3 | cell growth and/or maintenance, protein folding, regulation of apoptosis |  | | | | | | | | | | | | | | | | | | | | | | | | | | | |
| 115 | 217783\_s\_at | YPEL5 | -2.418526852 | 9.446415511 | -10.6836374 | 9.71E-010 | 1.60E-007 | yippee-like 5 (Drosophila) | transcription |  | | | | | | | | | | | | | | | | | | | | | | | | | | | |
| 116 | 200708\_at | GOT2 | 1.474681958 | 9.259987868 | 10.6792801 | 9.78E-010 | 1.60E-007 | glutamic-oxaloacetic transaminase 2, mitochondrial (aspartate aminotransferase 2) | amino acid metabolism, aspartate catabolism, biosynthesis |  | | | | | | | | | | | | | | | | | | | | | | | | | | | |
| 117 | 202378\_s\_at | OBRGRP | -1.751580362 | 8.783682959 | -10.6766679 | 9.82E-010 | 1.60E-007 | leptin receptor gene-related protein | NA |  | | | | | | | | | | | | | | | | | | | | | | | | | | | |
| 118 | 203612\_at | BYSL | 1.329320473 | 7.917364835 | 10.6748507 | 9.85E-010 | 1.60E-007 | bystin-like | cell adhesion, pregnancy |  | | | | | | | | | | | | | | | | | | | | | | | | | | | |
| 119 | 204025\_s\_at | PDCD2 | 1.925189516 | 6.904967137 | 10.6561233 | 1.01E-009 | 1.63E-007 | programmed cell death 2 | apoptosis |  | | | | | | | | | | | | | | | | | | | | | | | | | | | |
| 120 | 215708\_s\_at | PRIM2A | 1.697870595 | 6.019701759 | 10.6395827 | 1.04E-009 | 1.67E-007 | primase, polypeptide 2A, 58kDa | DNA replication, DNA replication, synthesis of RNA primer |  | | | | | | | | | | | | | | | | | | | | | | | | | | | |
| 121 | 203342\_at | TIMM17B | 0.579529767 | 8.848709583 | 10.5970951 | 1.12E-009 | 1.77E-007 | translocase of inner mitochondrial membrane 17 homolog B (yeast) | protein-mitochondrial targeting |  | | | | | | | | | | | | | | | | | | | | | | | | | | | |
| 122 | 212533\_at | WEE1 | 1.812251229 | 7.731820224 | 10.5925263 | 1.12E-009 | 1.77E-007 | WEE1 homolog (S. pombe) | mitosis, protein amino acid phosphorylation, regulation of cell cycle |  | | | | | | | | | | | | | | | | | | | | | | | | | | | |
| 123 | 218521\_s\_at | FLJ11011 | -1.156096242 | 5.809626289 | -10.5556677 | 1.19E-009 | 1.87E-007 | hypothetical protein FLJ11011 | regulation of transcription, DNA-dependent, ubiquitin cycle |  | | | | | | | | | | | | | | | | | | | | | | | | | | | |
| 124 | 210943\_s\_at | CHS1 | -2.918050222 | 9.683361979 | -10.5251243 | 1.26E-009 | 1.95E-007 | Chediak-Higashi syndrome 1 | cellular defense response, endosome to lysosome transport, protein transport, signal transduction |  | | | | | | | | | | | | | | | | | | | | | | | | | | | |
| 125 | 218145\_at | TRIB3 | 2.516865857 | 8.988875365 | 10.5124256 | 1.28E-009 | 1.98E-007 | tribbles homolog 3 (Drosophila) | apoptosis, protein amino acid phosphorylation |  | | | | | | | | | | | | | | | | | | | | | | | | | | | |
| 126 | 202314\_at | CYP51A1 | 2.17059711 | 6.414360823 | 10.5021565 | 1.30E-009 | 2.00E-007 | cytochrome P450, family 51, subfamily A, polypeptide 1 | cholesterol biosynthesis, electron transport |  | | | | | | | | | | | | | | | | | | | | | | | | | | | |
| 127 | 211911\_x\_at | HLA-C /// HLA-B | -3.456994497 | 12.2310677 | -10.4979173 | 1.31E-009 | 2.00E-007 | major histocompatibility complex, class I, C /// major histocompatibility complex, class I, C /// major histocompatibility complex, class I, B /// major histocompatibility complex, class I, B | antigen presentation, endogenous antigen, antigen processing, endogenous antigen via MHC class I, immune response, immune response |  | | | | | | | | | | | | | | | | | | | | | | | | | | | |
| 128 | 202117\_at | ARHGAP1 | -1.232403987 | 9.683390286 | -10.4795423 | 1.35E-009 | 2.04E-007 | Rho GTPase activating protein 1 | Rho protein signal transduction, cytoskeleton organization and biogenesis, signal transduction |  | | | | | | | | | | | | | | | | | | | | | | | | | | | |
| 129 | 208729\_x\_at | HLA-B | -3.629544141 | 11.84768419 | -10.4743391 | 1.36E-009 | 2.05E-007 | major histocompatibility complex, class I, B | antigen presentation, endogenous antigen, antigen processing, endogenous antigen via MHC class I, immune response |  | | | | | | | | | | | | | | | | | | | | | | | | | | | |
| 130 | 203109\_at | UBE2M | 1.399714472 | 8.815619875 | 10.4493941 | 1.42E-009 | 2.09E-007 | ubiquitin-conjugating enzyme E2M (UBC12 homolog, yeast) | ubiquitin cycle |  | | | | | | | | | | | | | | | | | | | | | | | | | | | |
| 131 | 208907\_s\_at | MRPS18B | 1.772776315 | 8.393047224 | 10.4465974 | 1.43E-009 | 2.09E-007 | mitochondrial ribosomal protein S18B | protein biosynthesis |  | | | | | | | | | | | | | | | | | | | | | | | | | | | |
| 132 | 212544\_at | TRIP3 | 1.66257215 | 8.19893804 | 10.4344698 | 1.46E-009 | 2.11E-007 | thyroid hormone receptor interactor 3 | intracellular protein transport, regulation of transcription, DNA-dependent |  | | | | | | | | | | | | | | | | | | | | | | | | | | | |
| 133 | 201662\_s\_at | ACSL3 | 1.939055656 | 7.431365007 | 10.4243419 | 1.48E-009 | 2.12E-007 | acyl-CoA synthetase long-chain family member 3 | fatty acid metabolism, metabolism |  | | | | | | | | | | | | | | | | | | | | | | | | | | | |
| 134 | 213226\_at | CCNA2 | 2.2293733 | 7.564692278 | 10.4237538 | 1.48E-009 | 2.12E-007 | cyclin A2 | cytokinesis, mitosis, mitotic G2 checkpoint, regulation of cyclin dependent protein kinase activity |  | | | | | | | | | | | | | | | | | | | | | | | | | | | |
| 135 | 212458\_at | SPRED2 | 2.308019443 | 6.586537471 | 10.3918955 | 1.56E-009 | 2.22E-007 | sprouty-related, EVH1 domain containing 2 | development, regulation of signal transduction |  | | | | | | | | | | | | | | | | | | | | | | | | | | | |
| 136 | 217865\_at | RNF130 | -1.441917258 | 9.811577931 | -10.3702728 | 1.62E-009 | 2.29E-007 | ring finger protein 130 | protein ubiquitination, proteolysis and peptidolysis |  | | | | | | | | | | | | | | | | | | | | | | | | | | | |
| 137 | 202240\_at | PLK1 | 1.884341812 | 7.281038346 | 10.3570298 | 1.65E-009 | 2.31E-007 | polo-like kinase 1 (Drosophila) | mitosis, protein amino acid phosphorylation, regulation of cell cycle |  | | | | | | | | | | | | | | | | | | | | | | | | | | | |
| 138 | 212215\_at | KIAA0436 | 1.330452635 | 7.146010014 | 10.3169335 | 1.77E-009 | 2.44E-007 | putative prolyl oligopeptidase | proteolysis and peptidolysis |  | | | | | | | | | | | | | | | | | | | | | | | | | | | |
| 139 | 205761\_s\_at | PP35 | 1.549075722 | 6.175243225 | 10.3119976 | 1.78E-009 | 2.44E-007 | protein similar to E.coli yhdg and R. capsulatus nifR3 | tRNA processing |  | | | | | | | | | | | | | | | | | | | | | | | | | | | |
| 140 | 205429\_s\_at | MPP6 | 1.068985195 | 4.794408614 | 10.3032819 | 1.81E-009 | 2.46E-007 | membrane protein, palmitoylated 6 (MAGUK p55 subfamily member 6) | protein complex assembly |  | | | | | | | | | | | | | | | | | | | | | | | | | | | |
| 141 | 218712\_at | FLJ20508 | 1.816115842 | 4.847675639 | 10.2991235 | 1.82E-009 | 2.46E-007 | hypothetical protein FLJ20508 | NA |  | | | | | | | | | | | | | | | | | | | | | | | | | | | |
| 142 | 218860\_at | MGC3162 | 0.942089245 | 7.67082048 | 10.2965854 | 1.83E-009 | 2.46E-007 | hypothetical protein MGC3162 | NA |  | | | | | | | | | | | | | | | | | | | | | | | | | | | |
| 143 | 209165\_at | AATF | 0.92454295 | 8.679280728 | 10.2910376 | 1.84E-009 | 2.47E-007 | apoptosis antagonizing transcription factor | anti-apoptosis |  | | | | | | | | | | | | | | | | | | | | | | | | | | | |
| 144 | 209248\_at | GHITM | 1.607864792 | 10.04149901 | 10.2723333 | 1.90E-009 | 2.50E-007 | growth hormone inducible transmembrane protein | NA |  | | | | | | | | | | | | | | | | | | | | | | | | | | | |
| 145 | 204831\_at | CDK8 | 1.931186435 | 5.864052743 | 10.2610309 | 1.94E-009 | 2.53E-007 | Cyclin-dependent kinase 8 | cytokinesis, protein amino acid phosphorylation, regulation of cell cycle, regulation of transcription, DNA-dependent |  | | | | | | | | | | | | | | | | | | | | | | | | | | | |
| 146 | 204122\_at | TYROBP | -4.853509876 | 9.888560408 | -10.2346043 | 2.02E-009 | 2.63E-007 | TYRO protein tyrosine kinase binding protein | cellular defense response, intracellular signaling cascade |  | | | | | | | | | | | | | | | | | | | | | | | | | | | |
| 147 | 218622\_at | NUP37 | 2.249078252 | 8.519577851 | 10.2286652 | 2.04E-009 | 2.64E-007 | nucleoporin 37kDa | protein transport |  | | | | | | | | | | | | | | | | | | | | | | | | | | | |
| 148 | 218209\_s\_at | P15RS | 2.104695945 | 6.725275282 | 10.2109121 | 2.11E-009 | 2.67E-007 | hypothetical protein FLJ10656 | NA |  | | | | | | | | | | | | | | | | | | | | | | | | | | | |
| 149 | 206380\_s\_at | PFC | -3.478308201 | 9.107162458 | -10.2056787 | 2.12E-009 | 2.68E-007 | properdin P factor, complement | complement activation, alternative pathway, defense response to bacteria, immune response |  | | | | | | | | | | | | | | | | | | | | | | | | | | | |
| 150 | 208813\_at | GOT1 | 2.310764898 | 7.679381346 | 10.1890787 | 2.18E-009 | 2.72E-007 | glutamic-oxaloacetic transaminase 1, soluble (aspartate aminotransferase 1) | amino acid metabolism, aspartate catabolism, biosynthesis |  | | | | | | | | | | | | | | | | | | | | | | | | | | | |
| 151 | 221090\_s\_at | FLJ10826 | 1.349411104 | 7.660432901 | 10.1862043 | 2.19E-009 | 2.72E-007 | hypothetical protein FLJ10826 | protein metabolism |  | | | | | | | | | | | | | | | | | | | | | | | | | | | |
| 152 | 201391\_at | TRAP1 | 1.968828436 | 9.112560472 | 10.1833341 | 2.21E-009 | 2.72E-007 | TNF receptor-associated protein 1 | protein folding |  | | | | | | | | | | | | | | | | | | | | | | | | | | | |
| 153 | 201531\_at | ZFP36 | -2.884518217 | 10.20809526 | -10.1743006 | 2.24E-009 | 2.73E-007 | zinc finger protein 36, C3H type, homolog (mouse) | mRNA catabolism |  | | | | | | | | | | | | | | | | | | | | | | | | | | | |
| 154 | 204162\_at | KNTC2 | 2.797168155 | 6.477678828 | 10.1722626 | 2.25E-009 | 2.73E-007 | kinetochore associated 2 | mitosis, mitotic sister chromatid segregation |  | | | | | | | | | | | | | | | | | | | | | | | | | | | |
| 155 | 220235\_s\_at | RIF1 | 2.479316018 | 6.21332196 | 10.1716454 | 2.25E-009 | 2.73E-007 | receptor-interacting factor 1 | NA |  | | | | | | | | | | | | | | | | | | | | | | | | | | | |
| 156 | 208916\_at | SLC1A5 | 1.494890957 | 8.673147553 | 10.1335027 | 2.40E-009 | 2.89E-007 | solute carrier family 1 (neutral amino acid transporter), member 5 | dicarboxylic acid transport, neutral amino acid transport, transport |  | | | | | | | | | | | | | | | | | | | | | | | | | | | |
| 157 | 219505\_at | CECR1 | -3.539197636 | 9.649492447 | -10.1278934 | 2.42E-009 | 2.89E-007 | cat eye syndrome chromosome region, candidate 1 | development, purine ribonucleoside monophosphate biosynthesis |  | | | | | | | | | | | | | | | | | | | | | | | | | | | |
| 158 | 201897\_s\_at | CKS1B | 2.189007833 | 9.118746823 | 10.1134691 | 2.48E-009 | 2.93E-007 | CDC28 protein kinase regulatory subunit 1B | cell cycle, cytokinesis |  | | | | | | | | | | | | | | | | | | | | | | | | | | | |
| 159 | 218281\_at | MRPL48 | 1.483373665 | 8.256930614 | 10.0609771 | 2.71E-009 | 3.18E-007 | mitochondrial ribosomal protein L48 | NA |  | | | | | | | | | | | | | | | | | | | | | | | | | | | |
| 160 | 203025\_at | ARD1 | 1.519089149 | 7.867827919 | 10.0371595 | 2.82E-009 | 3.28E-007 | ARD1 homolog, N-acetyltransferase (S. cerevisiae) | DNA packaging, chromosome organization and biogenesis (sensu Eukaryota), internal protein amino acid acetylation |  | | | | | | | | | | | | | | | | | | | | | | | | | | | |
| 161 | 218949\_s\_at | QRSL1 | 1.689480249 | 7.130878171 | 10.0362872 | 2.82E-009 | 3.28E-007 | glutaminyl-tRNA synthase (glutamine-hydrolyzing)-like 1 | protein biosynthesis |  | | | | | | | | | | | | | | | | | | | | | | | | | | | |
| 162 | 206715\_at | TFEC | -2.745622839 | 6.444940595 | -10.0226483 | 2.89E-009 | 3.34E-007 | transcription factor EC | NA |  | | | | | | | | | | | | | | | | | | | | | | | | | | | |
| 163 | 211569\_s\_at | HADHSC | 1.842254276 | 7.417461797 | 9.99249597 | 3.04E-009 | 3.50E-007 | L-3-hydroxyacyl-Coenzyme A dehydrogenase, short chain | fatty acid metabolism |  | | | | | | | | | | | | | | | | | | | | | | | | | | | |
| 164 | 208975\_s\_at | KPNB1 | 1.571984442 | 7.781334662 | 9.96967283 | 3.16E-009 | 3.62E-007 | karyopherin (importin) beta 1 | NLS-bearing substrate-nucleus import, protein transport, protein-nucleus import, docking, protein-nucleus import, translocation |  | | | | | | | | | | | | | | | | | | | | | | | | | | | |
| 165 | 200913\_at | PPM1G | 1.33095935 | 8.340721011 | 9.96172368 | 3.20E-009 | 3.65E-007 | protein phosphatase 1G (formerly 2C), magnesium-dependent, gamma isoform | cell cycle arrest, protein amino acid dephosphorylation |  | | | | | | | | | | | | | | | | | | | | | | | | | | | |
| 166 | 201342\_at | SNRPC | 1.065934683 | 9.106093905 | 9.95045568 | 3.26E-009 | 3.68E-007 | small nuclear ribonucleoprotein polypeptide C | RNA splicing |  | | | | | | | | | | | | | | | | | | | | | | | | | | | |
| 167 | 210115\_at | RPL39L | 1.450121977 | 5.189295526 | 9.9429697 | 3.30E-009 | 3.71E-007 | ribosomal protein L39-like | protein biosynthesis |  | | | | | | | | | | | | | | | | | | | | | | | | | | | |
| 168 | 205220\_at | GPR109B | -3.477261665 | 8.007538326 | -9.93146348 | 3.37E-009 | 3.74E-007 | G protein-coupled receptor 109B /// G protein-coupled receptor 109B | G-protein coupled receptor protein signaling pathway |  | | | | | | | | | | | | | | | | | | | | | | | | | | | |
| 169 | 201264\_at | COPE | 0.741712534 | 9.602837362 | 9.91469482 | 3.47E-009 | 3.80E-007 | coatomer protein complex, subunit epsilon | antimicrobial humoral response (sensu Vertebrata), intracellular protein transport |  | | | | | | | | | | | | | | | | | | | | | | | | | | | |
| 170 | 205339\_at | SIL | 1.146918795 | 6.232864877 | 9.91349636 | 3.47E-009 | 3.80E-007 | TAL1 (SCL) interrupting locus | cell proliferation |  | | | | | | | | | | | | | | | | | | | | | | | | | | | |
| 171 | 219229\_at | SLCO3A1 | -2.414669413 | 6.848418111 | -9.91305723 | 3.48E-009 | 3.80E-007 | solute carrier organic anion transporter family, member 3A1 | ion transport |  | | | | | | | | | | | | | | | | | | | | | | | | | | | |
| 172 | 221750\_at | HMGCS1 | 2.236851414 | 6.843049429 | 9.90176411 | 3.54E-009 | 3.85E-007 | 3-hydroxy-3-methylglutaryl-Coenzyme A synthase 1 (soluble) | acetyl-CoA metabolism, cholesterol biosynthesis, lipid metabolism |  | | | | | | | | | | | | | | | | | | | | | | | | | | | |
| 173 | 220789\_s\_at | TBRG4 | 1.433417475 | 7.69294759 | 9.90030182 | 3.55E-009 | 3.85E-007 | transforming growth factor beta regulator 4 | G1 phase of mitotic cell cycle, cell cycle arrest, positive regulation of cell proliferation |  | | | | | | | | | | | | | | | | | | | | | | | | | | | |
| 174 | 217336\_at | RPS10 | 1.448703698 | 7.495716832 | 9.88986005 | 3.62E-009 | 3.90E-007 | ribosomal protein S10 | protein biosynthesis |  | | | | | | | | | | | | | | | | | | | | | | | | | | | |
| 175 | 212891\_s\_at | GADD45GIP1 | 1.980976 | 6.350230832 | 9.88277086 | 3.66E-009 | 3.93E-007 | growth arrest and DNA-damage-inducible, gamma interacting protein 1 | NA |  | | | | | | | | | | | | | | | | | | | | | | | | | | | |
| 176 | 204221\_x\_at | HRB2 | -2.461440539 | 7.47038364 | -9.85719739 | 3.82E-009 | 4.06E-007 | HIV-1 rev binding protein 2 | NA |  | | | | | | | | | | | | | | | | | | | | | | | | | | | |
| 177 | 203493\_s\_at | KIAA0092 | 2.193294201 | 6.667588545 | 9.82817823 | 4.02E-009 | 4.25E-007 | translokin | NA |  | | | | | | | | | | | | | | | | | | | | | | | | | | | |
| 178 | 201397\_at | PHGDH | 2.835868787 | 7.683391278 | 9.82142242 | 4.06E-009 | 4.28E-007 | phosphoglycerate dehydrogenase | L-serine biosynthesis, brain development |  | | | | | | | | | | | | | | | | | | | | | | | | | | | |
| 179 | 202785\_at | NDUFA7 | 1.494920721 | 7.702224825 | 9.80159513 | 4.20E-009 | 4.38E-007 | NADH dehydrogenase (ubiquinone) 1 alpha subcomplex, 7, 14.5kDa | NA |  | | | | | | | | | | | | | | | | | | | | | | | | | | | |
| 180 | 201782\_s\_at | AIP | 0.851379559 | 9.144027785 | 9.79737821 | 4.23E-009 | 4.40E-007 | aryl hydrocarbon receptor interacting protein | protein folding |  | | | | | | | | | | | | | | | | | | | | | | | | | | | |
| 181 | 208829\_at | TAPBP | -0.983379925 | 10.09996782 | -9.79042206 | 4.29E-009 | 4.43E-007 | TAP binding protein (tapasin) | antigen processing, endogenous antigen via MHC class I, immune response, peptide antigen stabilization, protein complex assembly, retrograde transport, Golgi to ER |  | | | | | | | | | | | | | | | | | | | | | | | | | | | |
| 182 | 221513\_s\_at | UTP14A /// UTP14C | 1.47803072 | 7.394245112 | 9.78453459 | 4.33E-009 | 4.45E-007 | UTP14, U3 small nucleolar ribonucleoprotein, homolog A (yeast) /// UTP14, U3 small nucleolar ribonucleoprotein, homolog C (yeast) | NA |  | | | | | | | | | | | | | | | | | | | | | | | | | | | |
| 183 | 203100\_s\_at | CDYL | 0.988168812 | 6.903100827 | 9.76877384 | 4.45E-009 | 4.55E-007 | chromodomain protein, Y-like | chromatin assembly or disassembly, metabolism, spermatogenesis |  | | | | | | | | | | | | | | | | | | | | | | | | | | | |
| 184 | 212894\_at | SUPV3L1 | 1.621163331 | 7.453997582 | 9.75064201 | 4.59E-009 | 4.67E-007 | suppressor of var1, 3-like 1 (S. cerevisiae) | NA |  | | | | | | | | | | | | | | | | | | | | | | | | | | | |
| 185 | 206550\_s\_at | NUP155 | 1.737197931 | 6.581104409 | 9.74896919 | 4.60E-009 | 4.67E-007 | nucleoporin 155kDa | nucleocytoplasmic transport, transport |  | | | | | | | | | | | | | | | | | | | | | | | | | | | |
| 186 | 213326\_at | VAMP1 | -2.065026065 | 7.611319136 | -9.74160369 | 4.66E-009 | 4.71E-007 | vesicle-associated membrane protein 1 (synaptobrevin 1) | NA |  | | | | | | | | | | | | | | | | | | | | | | | | | | | |
| 187 | 221479\_s\_at | BNIP3L | -1.541240313 | 9.25434777 | -9.73856044 | 4.68E-009 | 4.71E-007 | BCL2/adenovirus E1B 19kDa interacting protein 3-like /// BCL2/adenovirus E1B 19kDa interacting protein 3-like | apoptosis, induction of apoptosis, negative regulation of survival gene product activity |  | | | | | | | | | | | | | | | | | | | | | | | | | | | |
| 188 | 202020\_s\_at | LANCL1 | 1.420630697 | 7.819797946 | 9.7093768 | 4.93E-009 | 4.93E-007 | LanC lantibiotic synthetase component C-like 1 (bacterial) | G-protein coupled receptor protein signaling pathway |  | | | | | | | | | | | | | | | | | | | | | | | | | | | |
| 189 | 212072\_s\_at | CSNK2A1 | 1.237900053 | 8.366531482 | 9.69621024 | 5.04E-009 | 5.02E-007 | casein kinase 2, alpha 1 polypeptide | Wnt receptor signaling pathway, protein amino acid phosphorylation, signal transduction |  | | | | | | | | | | | | | | | | | | | | | | | | | | | |
| 190 | 216997\_x\_at | TLE4 | -2.596000819 | 6.64930605 | -9.66186351 | 5.35E-009 | 5.26E-007 | transducin-like enhancer of split 4 (E(sp1) homolog, Drosophila) | frizzled signaling pathway, regulation of transcription, DNA-dependent |  | | | | | | | | | | | | | | | | | | | | | | | | | | | |
| 191 | 205543\_at | APG-1 | 1.640985548 | 5.226370112 | 9.6574992 | 5.39E-009 | 5.27E-007 | heat shock protein (hsp110 family) | protein folding, response to unfolded protein |  | | | | | | | | | | | | | | | | | | | | | | | | | | | |
| 192 | 203023\_at | HSPC111 | 2.060507965 | 6.441878331 | 9.63855855 | 5.57E-009 | 5.42E-007 | hypothetical protein HSPC111 | NA |  | | | | | | | | | | | | | | | | | | | | | | | | | | | |
| 193 | 203110\_at | PTK2B | -1.589686692 | 8.28712341 | -9.63674303 | 5.58E-009 | 5.42E-007 | PTK2B protein tyrosine kinase 2 beta | apoptosis, cell adhesion, positive regulation of cell proliferation, protein amino acid phosphorylation, protein complex assembly, response to stress, signal complex formation, signal transduction |  | | | | | | | | | | | | | | | | | | | | | | | | | | | |
| 194 | 203591\_s\_at | CSF3R | -3.494369159 | 9.046507684 | -9.63029387 | 5.65E-009 | 5.45E-007 | colony stimulating factor 3 receptor (granulocyte) /// colony stimulating factor 3 receptor (granulocyte) | cell adhesion, defense response, signal transduction |  | | | | | | | | | | | | | | | | | | | | | | | | | | | |
| 195 | 214095\_at | SHMT2 | 2.40322338 | 6.686435026 | 9.61096878 | 5.84E-009 | 5.59E-007 | serine hydroxymethyltransferase 2 (mitochondrial) | L-serine metabolism, glycine metabolism, one-carbon compound metabolism |  | | | | | | | | | | | | | | | | | | | | | | | | | | | |
| 196 | 211696\_x\_at | HBB | -5.645945677 | 11.19154059 | -9.60770336 | 5.87E-009 | 5.60E-007 | hemoglobin, beta /// hemoglobin, beta | oxygen transport, transport |  | | | | | | | | | | | | | | | | | | | | | | | | | | | |
| 197 | 215380\_s\_at | C7orf24 | 1.698978319 | 8.965269776 | 9.6029489 | 5.92E-009 | 5.62E-007 | chromosome 7 open reading frame 24 | NA |  | | | | | | | | | | | | | | | | | | | | | | | | | | | |
| 198 | 204076\_at | ENTPD4 | -1.224821669 | 7.588755753 | -9.59645184 | 5.99E-009 | 5.66E-007 | ectonucleoside triphosphate diphosphohydrolase 4 | UDP catabolism |  | | | | | | | | | | | | | | | | | | | | | | | | | | | |
| 199 | 213704\_at | RABGGTB | 1.421347798 | 4.977302939 | 9.59420525 | 6.01E-009 | 5.66E-007 | Rab geranylgeranyltransferase, beta subunit | protein modification, visual perception |  | | | | | | | | | | | | | | | | | | | | | | | | | | | |
| 200 | 31807\_at | DDX49 | 0.755210036 | 8.783204005 | 9.59136584 | 6.04E-009 | 5.66E-007 | DEAD (Asp-Glu-Ala-Asp) box polypeptide 49 | NA |  | | | | | | | | | | | | | | | | | | | | | | | | | | | |
| 201 | 212359\_s\_at | KIAA0913 | -1.178541458 | 8.15996098 | -9.57264918 | 6.24E-009 | 5.83E-007 | KIAA0913 | NA |  | | | | | | | | | | | | | | | | | | | | | | | | | | | |
| 202 | 210457\_x\_at | HMGA1 | 2.255980365 | 7.731787577 | 9.55885903 | 6.39E-009 | 5.94E-007 | high mobility group AT-hook 1 | DNA unwinding, chromosome organization and biogenesis (sensu Eukaryota), loss of chromatin silencing, nucleosome disassembly, positive regulation of transcription, protein complex assembly, regulation of transcription, DNA-dependent |  | | | | | | | | | | | | | | | | | | | | | | | | | | | |
| 203 | 212946\_at | KIAA0564 | 1.088350901 | 7.230673825 | 9.55261166 | 6.46E-009 | 5.98E-007 | KIAA0564 protein | NA |  | | | | | | | | | | | | | | | | | | | | | | | | | | | |
| 204 | 201027\_s\_at | EIF5B | 1.364284751 | 7.589244 | 9.5469641 | 6.53E-009 | 6.02E-007 | eukaryotic translation initiation factor 5B | protein biosynthesis, regulation of translational initiation |  | | | | | | | | | | | | | | | | | | | | | | | | | | | |
| 205 | 218768\_at | NUP107 | 1.579274053 | 8.763340136 | 9.52227097 | 6.81E-009 | 6.23E-007 | nucleoporin 107kDa | mRNA-nucleus export, protein transport |  | | | | | | | | | | | | | | | | | | | | | | | | | | | |
| 206 | 203150\_at | RAB9P40 | 0.992060011 | 8.085762556 | 9.51741036 | 6.87E-009 | 6.23E-007 | Rab9 effector p40 | receptor mediated endocytosis, vesicle docking during exocytosis |  | | | | | | | | | | | | | | | | | | | | | | | | | | | |
| 207 | 208693\_s\_at | GARS | 2.336999107 | 9.937926701 | 9.5041853 | 7.03E-009 | 6.33E-007 | glycyl-tRNA synthetase | glycyl-tRNA aminoacylation, protein biosynthesis |  | | | | | | | | | | | | | | | | | | | | | | | | | | | |
| 208 | 219494\_at | RAD54B | 1.079258417 | 5.977697766 | 9.50392266 | 7.04E-009 | 6.33E-007 | RAD54 homolog B (S. cerevisiae) | DNA repair, cell growth and/or maintenance, meiotic recombination, mitotic recombination |  | | | | | | | | | | | | | | | | | | | | | | | | | | | |
| 209 | 221685\_s\_at | FLJ20364 | 1.319233075 | 6.212972475 | 9.49591514 | 7.13E-009 | 6.39E-007 | hypothetical protein FLJ20364 | NA |  | | | | | | | | | | | | | | | | | | | | | | | | | | | |
| 210 | 203284\_s\_at | HS2ST1 | 1.702268622 | 6.466777182 | 9.49047304 | 7.20E-009 | 6.43E-007 | heparan sulfate 2-O-sulfotransferase 1 | NA |  | | | | | | | | | | | | | | | | | | | | | | | | | | | |
| 211 | 203664\_s\_at | POLR2D | 1.167606941 | 7.371212448 | 9.4747629 | 7.40E-009 | 6.58E-007 | polymerase (RNA) II (DNA directed) polypeptide D | transcription, transcription from Pol II promoter |  | | | | | | | | | | | | | | | | | | | | | | | | | | | |
| 212 | 214459\_x\_at | HLA-C | -2.503020626 | 12.55217876 | -9.46621384 | 7.52E-009 | 6.65E-007 | major histocompatibility complex, class I, C | antigen presentation, endogenous antigen, antigen processing, endogenous antigen via MHC class I, immune response |  | | | | | | | | | | | | | | | | | | | | | | | | | | | |
| 213 | 202697\_at | CPSF5 | 1.47132321 | 8.319988684 | 9.45315923 | 7.69E-009 | 6.78E-007 | cleavage and polyadenylation specific factor 5, 25 kDa | mRNA processing |  | | | | | | | | | | | | | | | | | | | | | | | | | | | |
| 214 | 214661\_s\_at | C4orf9 | 1.663224532 | 8.227350122 | 9.44719514 | 7.77E-009 | 6.82E-007 | chromosome 4 open reading frame 9 | NA |  | | | | | | | | | | | | | | | | | | | | | | | | | | | |
| 215 | 203177\_x\_at | TFAM | 1.87094085 | 6.960110578 | 9.44161126 | 7.85E-009 | 6.86E-007 | transcription factor A, mitochondrial | DNA-dependent DNA replication, regulation of transcription from Pol I promoter, transcription from mitochondrial promoter |  | | | | | | | | | | | | | | | | | | | | | | | | | | | |
| 216 | 216228\_s\_at | WDHD1 | 0.801974133 | 4.347638689 | 9.43623259 | 7.92E-009 | 6.87E-007 | WD repeat and HMG-box DNA binding protein 1 | main pathways of carbohydrate metabolism, regulation of transcription, DNA-dependent |  | | | | | | | | | | | | | | | | | | | | | | | | | | | |
| 217 | 213333\_at | MDH2 | 1.421497822 | 7.523000849 | 9.43229297 | 7.98E-009 | 6.89E-007 | malate dehydrogenase 2, NAD (mitochondrial) | tricarboxylic acid cycle |  | | | | | | | | | | | | | | | | | | | | | | | | | | | |
| 218 | 209507\_at | RPA3 | 1.253990185 | 8.520017417 | 9.40823404 | 8.32E-009 | 7.16E-007 | replication protein A3, 14kDa | DNA repair, DNA replication |  | | | | | | | | | | | | | | | | | | | | | | | | | | | |
| 219 | 211080\_s\_at | NEK2 | 1.40711817 | 5.949781787 | 9.39058411 | 8.58E-009 | 7.36E-007 | NIMA (never in mitosis gene a)-related kinase 2 /// NIMA (never in mitosis gene a)-related kinase 2 | cytokinesis, meiosis, protein amino acid phosphorylation, regulation of mitosis |  | | | | | | | | | | | | | | | | | | | | | | | | | | | |
| 220 | 213626\_at | CBR4 | 1.083312353 | 5.994218133 | 9.36132972 | 9.04E-009 | 7.72E-007 | carbonic reductase 4 | metabolism |  | | | | | | | | | | | | | | | | | | | | | | | | | | | |
| 221 | 212474\_at | KIAA0241 | 1.245474532 | 7.114782551 | 9.35616728 | 9.12E-009 | 7.76E-007 | KIAA0241 protein | NA |  | | | | | | | | | | | | | | | | | | | | | | | | | | | |
| 222 | 210097\_s\_at | NOL7 | 1.764213435 | 8.996224582 | 9.29546833 | 1.02E-008 | 8.48E-007 | nucleolar protein 7, 27kDa | NA |  | | | | | | | | | | | | | | | | | | | | | | | | | | | |
| 223 | 215905\_s\_at | HPRP8BP | 1.001283811 | 8.501922331 | 9.29108093 | 1.02E-008 | 8.51E-007 | U5 snRNP-specific 40 kDa protein (hPrp8-binding) | RNA splicing, nuclear mRNA splicing, via spliceosome |  | | | | | | | | | | | | | | | | | | | | | | | | | | | |
| 224 | 210023\_s\_at | NSPC1 | 1.418049638 | 8.322577004 | 9.27888074 | 1.05E-008 | 8.64E-007 | likely ortholog of mouse nervous system polycomb 1 | protein ubiquitination |  | | | | | | | | | | | | | | | | | | | | | | | | | | | |
| 225 | 221712\_s\_at | FLJ10439 | 1.631446418 | 7.470425398 | 9.26831685 | 1.07E-008 | 8.77E-007 | hypothetical protein FLJ10439 /// hypothetical protein FLJ10439 | NA |  | | | | | | | | | | | | | | | | | | | | | | | | | | | |
| 226 | 218882\_s\_at | WDR3 | 1.510150893 | 7.528103665 | 9.26464105 | 1.07E-008 | 8.77E-007 | WD repeat domain 3 | NA |  | | | | | | | | | | | | | | | | | | | | | | | | | | | |
| 227 | 209408\_at | KIF2C | 2.115957747 | 8.333130233 | 9.26400123 | 1.07E-008 | 8.77E-007 | kinesin family member 2C | cell proliferation, mitosis |  | | | | | | | | | | | | | | | | | | | | | | | | | | | |
| 228 | 218376\_s\_at | NICAL | -2.093884661 | 8.419386887 | -9.25617173 | 1.09E-008 | 8.86E-007 | NEDD9 interacting protein with calponin homology and LIM domains | cytoskeleton organization and biogenesis, signal transduction |  | | | | | | | | | | | | | | | | | | | | | | | | | | | |
| 229 | 209085\_x\_at | RFC1 | 1.320931035 | 7.355487214 | 9.24190679 | 1.12E-008 | 9.05E-007 | replication factor C (activator 1) 1, 145kDa | DNA-dependent DNA replication, regulation of transcription, DNA-dependent, telomerase-dependent telomere maintenance |  | | | | | | | | | | | | | | | | | | | | | | | | | | | |
| 230 | 205053\_at | PRIM1 | 1.816213422 | 8.667816177 | 9.23902022 | 1.12E-008 | 9.07E-007 | primase, polypeptide 1, 49kDa | DNA replication, DNA replication, synthesis of RNA primer |  | | | | | | | | | | | | | | | | | | | | | | | | | | | |
| 231 | 202880\_s\_at | PSCD1 | -1.994849842 | 7.688857098 | -9.22546879 | 1.15E-008 | 9.26E-007 | pleckstrin homology, Sec7 and coiled-coil domains 1(cytohesin 1) | vesicle-mediated transport |  | | | | | | | | | | | | | | | | | | | | | | | | | | | |
| 232 | 207891\_s\_at | TREX2 | 1.229490955 | 6.591989367 | 9.20869315 | 1.18E-008 | 9.50E-007 | three prime repair exonuclease 2 | NA |  | | | | | | | | | | | | | | | | | | | | | | | | | | | |
| 233 | 211795\_s\_at | FYB | -3.077138371 | 7.821493116 | -9.18901318 | 1.23E-008 | 9.79E-007 | FYN binding protein (FYB-120/130) | NLS-bearing substrate-nucleus import, immune response, protein amino acid phosphorylation, protein kinase cascade, signal transduction |  | | | | | | | | | | | | | | | | | | | | | | | | | | | |
| 234 | 217913\_at | VPS4A | 0.841511027 | 8.150852703 | 9.18760554 | 1.23E-008 | 9.79E-007 | vacuolar protein sorting 4A (yeast) | NA |  | | | | | | | | | | | | | | | | | | | | | | | | | | | |
| 235 | 217611\_at | LOC157697 | -0.653988993 | 6.660474573 | -9.18570362 | 1.23E-008 | 9.79E-007 | hypothetical protein LOC157697 | NA |  | | | | | | | | | | | | | | | | | | | | | | | | | | | |
| 236 | 218772\_x\_at | C9orf87 | 2.370954225 | 5.743003264 | 9.1558322 | 1.30E-008 | 1.03E-006 | chromosome 9 open reading frame 87 | NA |  | | | | | | | | | | | | | | | | | | | | | | | | | | | |
| 237 | 209129\_at | TRIP6 | 2.386036961 | 7.477291837 | 9.14837384 | 1.32E-008 | 1.04E-006 | thyroid hormone receptor interactor 6 | NA |  | | | | | | | | | | | | | | | | | | | | | | | | | | | |
| 238 | 221746\_at | UBL4 | 0.908240506 | 8.293806951 | 9.14409365 | 1.33E-008 | 1.04E-006 | ubiquitin-like 4 | protein modification |  | | | | | | | | | | | | | | | | | | | | | | | | | | | |
| 239 | 203228\_at | PAFAH1B3 | 1.276701308 | 7.368565728 | 9.13515649 | 1.35E-008 | 1.05E-006 | platelet-activating factor acetylhydrolase, isoform Ib, gamma subunit 29kDa | lipid catabolism, neurogenesis |  | | | | | | | | | | | | | | | | | | | | | | | | | | | |
| 240 | 201475\_x\_at | MARS | 1.586999845 | 9.219467841 | 9.12313071 | 1.38E-008 | 1.06E-006 | methionine-tRNA synthetase | methionyl-tRNA aminoacylation, protein biosynthesis |  | | | | | | | | | | | | | | | | | | | | | | | | | | | |
| 241 | 221923\_s\_at | NPM1 | 2.100387377 | 10.27216796 | 9.12260824 | 1.38E-008 | 1.06E-006 | nucleophosmin (nucleolar phosphoprotein B23, numatrin) | activation of NF-kappaB transcription factor, cell aging, cell growth and/or maintenance, centrosome cycle, intracellular protein transport, negative regulation of cell proliferation, nucleocytoplasmic transport, response to stress, ribosome assembly |  | | | | | | | | | | | | | | | | | | | | | | | | | | | |
| 242 | 202191\_s\_at | GAS7 | -1.730921844 | 7.839081487 | -9.10531907 | 1.43E-008 | 1.09E-006 | growth arrest-specific 7 | cell cycle arrest, cell growth and/or maintenance, development, neurogenesis |  | | | | | | | | | | | | | | | | | | | | | | | | | | | |
| 243 | 34764\_at | LARS2 | 1.26247951 | 6.056446861 | 9.09694867 | 1.45E-008 | 1.10E-006 | leucyl-tRNA synthetase 2, mitochondrial | leucyl-tRNA aminoacylation, protein biosynthesis |  | | | | | | | | | | | | | | | | | | | | | | | | | | | |
| 244 | 210028\_s\_at | ORC3L | 1.515442015 | 6.274442988 | 9.09430061 | 1.45E-008 | 1.10E-006 | origin recognition complex, subunit 3-like (yeast) | DNA replication |  | | | | | | | | | | | | | | | | | | | | | | | | | | | |
| 245 | 212087\_s\_at | ERAL1 | 1.166814027 | 7.198408655 | 9.09402812 | 1.45E-008 | 1.10E-006 | Era G-protein-like 1 (E. coli) | cell growth and/or maintenance |  | | | | | | | | | | | | | | | | | | | | | | | | | | | |
| 246 | 213280\_at | GARNL4 | -1.757576973 | 6.7914366 | -9.09352491 | 1.46E-008 | 1.10E-006 | GTPase activating Rap/RanGAP domain-like 4 | NA |  | | | | | | | | | | | | | | | | | | | | | | | | | | | |
| 247 | 213998\_s\_at | DDX17 | -2.40314132 | 8.299333154 | -9.07474894 | 1.51E-008 | 1.13E-006 | DEAD (Asp-Glu-Ala-Asp) box polypeptide 17 | RNA processing |  | | | | | | | | | | | | | | | | | | | | | | | | | | | |
| 248 | 203395\_s\_at | HES1 | 1.041613599 | 5.483547071 | 9.06711876 | 1.53E-008 | 1.14E-006 | hairy and enhancer of split 1, (Drosophila) | neurogenesis, regulation of transcription, DNA-dependent |  | | | | | | | | | | | | | | | | | | | | | | | | | | | |
| 249 | 207480\_s\_at | MEIS2 | 1.489907585 | 4.371753513 | 9.06168555 | 1.54E-008 | 1.15E-006 | Meis1, myeloid ecotropic viral integration site 1 homolog 2 (mouse) | negative regulation of transcription from Pol II promoter, regulation of transcription, DNA-dependent |  | | | | | | | | | | | | | | | | | | | | | | | | | | | |
| 250 | 200957\_s\_at | SSRP1 | 1.778485172 | 8.640343104 | 9.06012374 | 1.55E-008 | 1.15E-006 | structure specific recognition protein 1 | regulation of transcription, DNA-dependent |  | | | | | | | | | | | | | | | | | | | | | | | | | | | |
| 251 | 209817\_at | PPP3CB | 1.189876928 | 6.238122921 | 9.04726551 | 1.58E-008 | 1.17E-006 | protein phosphatase 3 (formerly 2B), catalytic subunit, beta isoform (calcineurin A beta) | protein amino acid dephosphorylation, regulation of cell cycle, signal transduction, transcription, DNA-dependent |  | | | | | | | | | | | | | | | | | | | | | | | | | | | |
| 252 | 217851\_s\_at | C20orf45 | 2.069323287 | 5.187766846 | 9.03390498 | 1.62E-008 | 1.19E-006 | chromosome 20 open reading frame 45 | NA |  | | | | | | | | | | | | | | | | | | | | | | | | | | | |
| 253 | 204244\_s\_at | ASK | 2.087540811 | 6.610733616 | 9.03122877 | 1.63E-008 | 1.19E-006 | activator of S phase kinase | NA |  | | | | | | | | | | | | | | | | | | | | | | | | | | | |
| 254 | 203775\_at | SLC25A13 | 1.512886347 | 5.991087228 | 9.00901117 | 1.70E-008 | 1.23E-006 | solute carrier family 25, member 13 (citrin) | transport |  | | | | | | | | | | | | | | | | | | | | | | | | | | | |
| 255 | 207396\_s\_at | ALG3 | 0.996204167 | 8.749399365 | 9.0078707 | 1.70E-008 | 1.23E-006 | asparagine-linked glycosylation 3 homolog (yeast, alpha-1,3-mannosyltransferase) | protein amino acid glycosylation |  | | | | | | | | | | | | | | | | | | | | | | | | | | | |
| 256 | 222192\_s\_at | FLJ21820 | 1.113144268 | 7.044964146 | 9.00725352 | 1.70E-008 | 1.23E-006 | hypothetical protein FLJ21820 | NA |  | | | | | | | | | | | | | | | | | | | | | | | | | | | |
| 257 | 212222\_at | PSME4 | 0.807901871 | 6.579751349 | 8.98636269 | 1.77E-008 | 1.27E-006 | proteasome (prosome, macropain) activator subunit 4 | NA |  | | | | | | | | | | | | | | | | | | | | | | | | | | | |
| 258 | 203740\_at | MPHOSPH6 | 0.847640956 | 8.613510492 | 8.98423629 | 1.77E-008 | 1.27E-006 | M-phase phosphoprotein 6 | M phase of mitotic cell cycle, regulation of cell cycle |  | | | | | | | | | | | | | | | | | | | | | | | | | | | |
| 259 | 205195\_at | AP1S1 | 0.988611169 | 6.402508987 | 8.97297858 | 1.81E-008 | 1.28E-006 | adaptor-related protein complex 1, sigma 1 subunit | intracellular protein transport, receptor mediated endocytosis |  | | | | | | | | | | | | | | | | | | | | | | | | | | | |
| 260 | 204905\_s\_at | EEF1E1 | 2.173996843 | 8.397742319 | 8.97274561 | 1.81E-008 | 1.28E-006 | eukaryotic translation elongation factor 1 epsilon 1 | protein biosynthesis |  | | | | | | | | | | | | | | | | | | | | | | | | | | | |
| 261 | 207845\_s\_at | ANAPC10 | 2.005403109 | 5.831781978 | 8.95731123 | 1.86E-008 | 1.31E-006 | anaphase promoting complex subunit 10 | cell cycle, mitosis, regulation of mitotic metaphase/anaphase transition, ubiquitin cycle |  | | | | | | | | | | | | | | | | | | | | | | | | | | | |
| 262 | 210416\_s\_at | CHEK2 | 0.735174244 | 7.437589621 | 8.94314374 | 1.91E-008 | 1.34E-006 | CHK2 checkpoint homolog (S. pombe) | DNA damage checkpoint, cell cycle, cell growth and/or maintenance, protein amino acid phosphorylation, response to DNA damage stimulus |  | | | | | | | | | | | | | | | | | | | | | | | | | | | |
| 263 | 213975\_s\_at | LYZ /// LILRB1 | -4.260672256 | 10.42212146 | -8.9420453 | 1.91E-008 | 1.34E-006 | lysozyme (renal amyloidosis) /// leukocyte immunoglobulin-like receptor, subfamily B (with TM and ITIM domains), member 1 | carbohydrate metabolism, cell wall catabolism, cytolysis, defense response to bacteria, immune response |  | | | | | | | | | | | | | | | | | | | | | | | | | | | |
| 264 | 209006\_s\_at | NPD014 | -1.872043375 | 7.360780518 | -8.93905365 | 1.93E-008 | 1.34E-006 | NPD014 protein | NA |  | | | | | | | | | | | | | | | | | | | | | | | | | | | |
| 265 | 218663\_at | HCAP-G | 2.55825826 | 6.405151742 | 8.93781939 | 1.93E-008 | 1.34E-006 | chromosome condensation protein G | cell cycle, mitosis, mitotic chromosome condensation |  | | | | | | | | | | | | | | | | | | | | | | | | | | | |
| 266 | 208964\_s\_at | FADS1 | 1.603441491 | 8.206607073 | 8.93089849 | 1.95E-008 | 1.35E-006 | fatty acid desaturase 1 | fatty acid biosynthesis, fatty acid desaturation |  | | | | | | | | | | | | | | | | | | | | | | | | | | | |
| 267 | 205677\_s\_at | DLEU1 | 1.340007481 | 7.556386792 | 8.91321547 | 2.02E-008 | 1.39E-006 | deleted in lymphocytic leukemia, 1 | negative regulation of cell cycle |  | | | | | | | | | | | | | | | | | | | | | | | | | | | |
| 268 | 210113\_s\_at | NALP1 | -1.344093641 | 7.421200145 | -8.90966854 | 2.03E-008 | 1.39E-006 | NACHT, leucine rich repeat and PYD containing 1 | caspase activation, defense response to pathogen, induction of apoptosis, regulation of apoptosis |  | | | | | | | | | | | | | | | | | | | | | | | | | | | |
| 269 | 202613\_at | CTPS | 2.056002135 | 7.551251153 | 8.90919294 | 2.03E-008 | 1.39E-006 | CTP synthase | glutamine metabolism, nucleobase, nucleoside, nucleotide and nucleic acid metabolism, pyrimidine nucleotide biosynthesis, response to drug |  | | | | | | | | | | | | | | | | | | | | | | | | | | | |
| 270 | 202591\_s\_at | SSBP1 | 2.128325184 | 9.541554226 | 8.90374156 | 2.05E-008 | 1.40E-006 | single-stranded DNA binding protein 1 | DNA replication |  | | | | | | | | | | | | | | | | | | | | | | | | | | | |
| 271 | 209482\_at | POP7 | 1.517369686 | 8.409511689 | 8.856135 | 2.24E-008 | 1.52E-006 | processing of precursor 7, ribonuclease P subunit (S. cerevisiae) | tRNA processing |  | | | | | | | | | | | | | | | | | | | | | | | | | | | |
| 272 | 213372\_at | PAQR3 | 1.852757985 | 4.686614081 | 8.8487544 | 2.27E-008 | 1.53E-006 | progestin and adipoQ receptor family member III | NA |  | | | | | | | | | | | | | | | | | | | | | | | | | | | |
| 273 | 220840\_s\_at | FLJ10706 | 1.506483751 | 5.648405098 | 8.84389372 | 2.29E-008 | 1.54E-006 | hypothetical protein FLJ10706 | NA |  | | | | | | | | | | | | | | | | | | | | | | | | | | | |
| 274 | 218981\_at | ACN9 | 1.049935771 | 4.892890361 | 8.84136162 | 2.30E-008 | 1.54E-006 | ACN9 homolog (S. cerevisiae) | NA |  | | | | | | | | | | | | | | | | | | | | | | | | | | | |
| 275 | 218984\_at | FLJ20485 | 1.961970231 | 7.204681992 | 8.83842067 | 2.31E-008 | 1.54E-006 | hypothetical protein FLJ20485 | tRNA processing |  | | | | | | | | | | | | | | | | | | | | | | | | | | | |
| 276 | 209110\_s\_at | RGL2 | -0.87676865 | 8.889291431 | -8.83108839 | 2.34E-008 | 1.55E-006 | ral guanine nucleotide dissociation stimulator-like 2 | Ras protein signal transduction, small GTPase mediated signal transduction |  | | | | | | | | | | | | | | | | | | | | | | | | | | | |
| 277 | 202268\_s\_at | APPBP1 | 1.763446074 | 8.220364518 | 8.82217327 | 2.38E-008 | 1.57E-006 | amyloid beta precursor protein binding protein 1, 59kDa | apoptosis, cell cycle, signal transduction, ubiquitin cycle |  | | | | | | | | | | | | | | | | | | | | | | | | | | | |
| 278 | 218512\_at | WDR12 | 2.051047995 | 8.371346661 | 8.81359725 | 2.42E-008 | 1.59E-006 | WD repeat domain 12 | NA |  | | | | | | | | | | | | | | | | | | | | | | | | | | | |
| 279 | 206499\_s\_at | CHC1 | 1.643658218 | 8.255499503 | 8.80848149 | 2.44E-008 | 1.59E-006 | chromosome condensation 1 | DNA packaging, G1/S transition of mitotic cell cycle, cell cycle, mitotic spindle assembly, regulation of S phase of mitotic cell cycle, regulation of mitosis |  | | | | | | | | | | | | | | | | | | | | | | | | | | | |
| 280 | 211558\_s\_at | DHPS | 1.224047662 | 8.523986059 | 8.80749413 | 2.45E-008 | 1.59E-006 | deoxyhypusine synthase | hypusine biosynthesis from peptidyl-lysine, positive regulation of cell proliferation, protein biosynthesis, spermidine catabolism to deoxyhypusine, using deoxyhypusine synthase |  | | | | | | | | | | | | | | | | | | | | | | | | | | | |
| 281 | 203167\_at | TIMP2 | -1.525389022 | 8.30832372 | -8.8014322 | 2.48E-008 | 1.61E-006 | tissue inhibitor of metalloproteinase 2 | NA |  | | | | | | | | | | | | | | | | | | | | | | | | | | | |
| 282 | 201756\_at | RPA2 | 1.397676493 | 8.824061072 | 8.79504339 | 2.50E-008 | 1.62E-006 | replication protein A2, 32kDa | DNA-dependent DNA replication |  | | | | | | | | | | | | | | | | | | | | | | | | | | | |
| 283 | 219177\_at | BRIX | 1.202225404 | 5.276034488 | 8.79208054 | 2.52E-008 | 1.62E-006 | BRIX | ribosome biogenesis |  | | | | | | | | | | | | | | | | | | | | | | | | | | | |
| 284 | 219539\_at | GEMIN6 | 1.045836953 | 7.367337862 | 8.78994926 | 2.53E-008 | 1.62E-006 | gem (nuclear organelle) associated protein 6 | spliceosomal snRNP biogenesis, spliceosome assembly |  | | | | | | | | | | | | | | | | | | | | | | | | | | | |
| 285 | 201563\_at | SORD | 2.360239641 | 7.912602144 | 8.78488041 | 2.55E-008 | 1.63E-006 | sorbitol dehydrogenase | sorbitol metabolism, visual perception |  | | | | | | | | | | | | | | | | | | | | | | | | | | | |
| 286 | 213211\_s\_at | TAF6L | 0.883925211 | 5.358653316 | 8.77802187 | 2.58E-008 | 1.65E-006 | TAF6-like RNA polymerase II, p300/CBP-associated factor (PCAF)-associated factor, 65kDa | chromatin remodeling, regulation of transcription from Pol II promoter |  | | | | | | | | | | | | | | | | | | | | | | | | | | | |
| 287 | 218339\_at | MRPL22 | 1.500273484 | 7.59561623 | 8.7663126 | 2.64E-008 | 1.68E-006 | mitochondrial ribosomal protein L22 | protein biosynthesis |  | | | | | | | | | | | | | | | | | | | | | | | | | | | |
| 288 | 219420\_s\_at | FLJ12439 | 0.594432043 | 7.932003937 | 8.76321629 | 2.66E-008 | 1.69E-006 | hypothetical protein FLJ12439 | NA |  | | | | | | | | | | | | | | | | | | | | | | | | | | | |
| 289 | 202233\_s\_at | UQCRH | 1.682501613 | 10.51376381 | 8.76026172 | 2.67E-008 | 1.69E-006 | ubiquinol-cytochrome c reductase hinge protein | aerobic respiration, electron transport, mitochondrial electron transport, ubiquinol to cytochrome c, oxidative phosphorylation |  | | | | | | | | | | | | | | | | | | | | | | | | | | | |
| 290 | 203830\_at | NJMU-R1 | 1.043784713 | 4.444072351 | 8.75193965 | 2.71E-008 | 1.71E-006 | protein kinase Njmu-R1 | spermatogenesis |  | | | | | | | | | | | | | | | | | | | | | | | | | | | |
| 291 | 218467\_at | TNFSF5IP1 | 1.453496231 | 9.756787152 | 8.73870041 | 2.78E-008 | 1.74E-006 | tumor necrosis factor superfamily, member 5-induced protein 1 | NA |  | | | | | | | | | | | | | | | | | | | | | | | | | | | |
| 292 | 208969\_at | NDUFA9 | 0.867744888 | 9.048986422 | 8.73130966 | 2.82E-008 | 1.76E-006 | NADH dehydrogenase (ubiquinone) 1 alpha subcomplex, 9, 39kDa | sodium ion transport |  | | | | | | | | | | | | | | | | | | | | | | | | | | | |
| 293 | 218799\_at | FLJ10349 | 1.18535589 | 7.139891623 | 8.72862232 | 2.83E-008 | 1.77E-006 | hypothetical protein FLJ10349 | NA |  | | | | | | | | | | | | | | | | | | | | | | | | | | | |
| 294 | 204207\_s\_at | RNGTT | 0.669301991 | 7.161396811 | 8.72488798 | 2.85E-008 | 1.77E-006 | RNA guanylyltransferase and 5'-phosphatase | mRNA capping, protein amino acid dephosphorylation |  | | | | | | | | | | | | | | | | | | | | | | | | | | | |
| 295 | 203316\_s\_at | SNRPE | 1.784093966 | 9.644542985 | 8.72201622 | 2.86E-008 | 1.78E-006 | small nuclear ribonucleoprotein polypeptide E | NA |  | | | | | | | | | | | | | | | | | | | | | | | | | | | |
| 296 | 201000\_at | AARS | 1.951985623 | 9.463359443 | 8.71261763 | 2.91E-008 | 1.80E-006 | alanyl-tRNA synthetase | alanyl-tRNA aminoacylation, protein biosynthesis, tRNA processing |  | | | | | | | | | | | | | | | | | | | | | | | | | | | |
| 297 | 221851\_at | LOC90379 | 0.864670366 | 7.264024247 | 8.70643417 | 2.95E-008 | 1.81E-006 | Hypothetical protein BC002926 | NA |  | | | | | | | | | | | | | | | | | | | | | | | | | | | |
| 298 | 217926\_at | HSPC023 | 1.113979781 | 8.793825761 | 8.7057685 | 2.95E-008 | 1.81E-006 | HSPC023 protein | NA |  | | | | | | | | | | | | | | | | | | | | | | | | | | | |
| 299 | 213223\_at | RPL28 | 1.19072387 | 8.419663357 | 8.70402485 | 2.96E-008 | 1.81E-006 | ribosomal protein L28 | protein biosynthesis |  | | | | | | | | | | | | | | | | | | | | | | | | | | | |
| 300 | 218911\_at | YEATS4 | 1.75896413 | 4.683180475 | 8.70092458 | 2.98E-008 | 1.82E-006 | YEATS domain containing 4 | NA |  | | | | | | | | | | | | | | | | | | | | | | | | | | | |
| 301 | 222250\_s\_at | DKFZP434B168 | 1.027695901 | 6.463263518 | 8.6999284 | 2.98E-008 | 1.82E-006 | DKFZP434B168 protein | NA |  | | | | | | | | | | | | | | | | | | | | | | | | | | | |
| 302 | 218017\_s\_at | FLJ32731 | -1.728024127 | 8.490416717 | -8.69102888 | 3.03E-008 | 1.84E-006 | Hypothetical protein FLJ32731 | NA |  | | | | | | | | | | | | | | | | | | | | | | | | | | | |
| 303 | 209619\_at | CD74 | -4.762072646 | 9.573180983 | -8.6856276 | 3.06E-008 | 1.85E-006 | CD74 antigen (invariant polypeptide of major histocompatibility complex, class II antigen-associated) | immune response, protein folding |  | | | | | | | | | | | | | | | | | | | | | | | | | | | |
| 304 | 221693\_s\_at | MRPS18A | 1.288770115 | 7.562368186 | 8.68026263 | 3.09E-008 | 1.87E-006 | mitochondrial ribosomal protein S18A /// mitochondrial ribosomal protein S18A | protein biosynthesis |  | | | | | | | | | | | | | | | | | | | | | | | | | | | |
| 305 | 215728\_s\_at | BACH | 0.879972136 | 7.205442323 | 8.67015491 | 3.15E-008 | 1.89E-006 | brain acyl-CoA hydrolase | lipid metabolism |  | | | | | | | | | | | | | | | | | | | | | | | | | | | |
| 306 | 204031\_s\_at | PCBP2 | 0.859921745 | 11.66321256 | 8.66609302 | 3.18E-008 | 1.90E-006 | poly(rC) binding protein 2 | mRNA metabolism |  | | | | | | | | | | | | | | | | | | | | | | | | | | | |
| 307 | 209551\_at | MGC11061 | 1.001111861 | 4.054374003 | 8.65838211 | 3.22E-008 | 1.92E-006 | hypothetical protein MGC11061 | NA |  | | | | | | | | | | | | | | | | | | | | | | | | | | | |
| 308 | 203022\_at | RNASEH2A | 1.5744707 | 7.796334549 | 8.65570159 | 3.24E-008 | 1.92E-006 | ribonuclease H2, large subunit | DNA replication, RNA catabolism |  | | | | | | | | | | | | | | | | | | | | | | | | | | | |
| 309 | 203931\_s\_at | MRPL12 | 1.759366982 | 8.196025809 | 8.65389159 | 3.25E-008 | 1.92E-006 | mitochondrial ribosomal protein L12 | protein biosynthesis |  | | | | | | | | | | | | | | | | | | | | | | | | | | | |
| 310 | 201682\_at | PMPCB | 1.222805386 | 9.435473227 | 8.638656 | 3.34E-008 | 1.97E-006 | peptidase (mitochondrial processing) beta | proteolysis and peptidolysis |  | | | | | | | | | | | | | | | | | | | | | | | | | | | |
| 311 | 201657\_at | ARL1 | 0.82227158 | 4.380340385 | 8.6382735 | 3.34E-008 | 1.97E-006 | ADP-ribosylation factor-like 1 | small GTPase mediated signal transduction |  | | | | | | | | | | | | | | | | | | | | | | | | | | | |
| 312 | 204700\_x\_at | MGC29875 | 1.127755431 | 5.781369371 | 8.61744267 | 3.48E-008 | 2.04E-006 | Hypothetical protein MGC29875 | NA |  | | | | | | | | | | | | | | | | | | | | | | | | | | | |
| 313 | 209849\_s\_at | RAD51C | 1.507935397 | 8.00837159 | 8.61323069 | 3.50E-008 | 2.05E-006 | RAD51 homolog C (S. cerevisiae) | DNA recombination, DNA repair |  | | | | | | | | | | | | | | | | | | | | | | | | | | | |
| 314 | 201406\_at | RPL36A | 1.031021704 | 12.31520086 | 8.61074247 | 3.52E-008 | 2.06E-006 | ribosomal protein L36a | protein biosynthesis |  | | | | | | | | | | | | | | | | | | | | | | | | | | | |
| 315 | 211429\_s\_at | SERPINA1 | -4.336129746 | 9.411927095 | -8.60141355 | 3.58E-008 | 2.08E-006 | serine (or cysteine) proteinase inhibitor, clade A (alpha-1 antiproteinase, antitrypsin), member 1 | acute-phase response |  | | | | | | | | | | | | | | | | | | | | | | | | | | | |
| 316 | 218654\_s\_at | MRPS33 | 1.328725488 | 8.357776915 | 8.59538615 | 3.62E-008 | 2.10E-006 | mitochondrial ribosomal protein S33 | protein biosynthesis |  | | | | | | | | | | | | | | | | | | | | | | | | | | | |
| 317 | 218617\_at | TRIT1 | 1.39805336 | 7.493550238 | 8.58094165 | 3.72E-008 | 2.14E-006 | tRNA isopentenyltransferase 1 | tRNA processing |  | | | | | | | | | | | | | | | | | | | | | | | | | | | |
| 318 | 43977\_at | FLJ20422 | 0.634240767 | 9.1898931 | 8.57738377 | 3.75E-008 | 2.15E-006 | hypothetical protein FLJ20422 | NA |  | | | | | | | | | | | | | | | | | | | | | | | | | | | |
| 319 | 200632\_s\_at | NDRG1 | -1.115757846 | 8.640221799 | -8.57525681 | 3.76E-008 | 2.15E-006 | N-myc downstream regulated gene 1 | cell differentiation, response to metal ion |  | | | | | | | | | | | | | | | | | | | | | | | | | | | |
| 320 | 208904\_s\_at | RPS28 | 0.800040891 | 12.84309456 | 8.57525066 | 3.76E-008 | 2.15E-006 | ribosomal protein S28 | NA |  | | | | | | | | | | | | | | | | | | | | | | | | | | | |
| 321 | 219173\_at | MYO15B | -1.512326864 | 8.258999374 | -8.57288559 | 3.78E-008 | 2.15E-006 | myosin XVB, pseudogene | NA |  | | | | | | | | | | | | | | | | | | | | | | | | | | | |
| 322 | 218877\_s\_at | C6orf75 | 2.216949878 | 6.517687176 | 8.57117374 | 3.79E-008 | 2.15E-006 | chromosome 6 open reading frame 75 | NA |  | | | | | | | | | | | | | | | | | | | | | | | | | | | |
| 323 | 201597\_at | COX7A2 | 1.296563554 | 10.22613363 | 8.56917792 | 3.80E-008 | 2.16E-006 | cytochrome c oxidase subunit VIIa polypeptide 2 (liver) | electron transport |  | | | | | | | | | | | | | | | | | | | | | | | | | | | |
| 324 | 203567\_s\_at | TRIM38 | -1.216656157 | 8.311451766 | -8.53461086 | 4.06E-008 | 2.29E-006 | tripartite motif-containing 38 | positive regulation of I-kappaB kinase/NF-kappaB cascade, protein ubiquitination |  | | | | | | | | | | | | | | | | | | | | | | | | | | | |
| 325 | 201077\_s\_at | NHP2L1 | 1.532674879 | 9.800149014 | 8.52668021 | 4.12E-008 | 2.31E-006 | NHP2 non-histone chromosome protein 2-like 1 (S. cerevisiae) | protein biosynthesis, regulation of cell cycle |  | | | | | | | | | | | | | | | | | | | | | | | | | | | |
| 326 | 203212\_s\_at | MTMR2 | 1.082167879 | 6.909101145 | 8.52443686 | 4.13E-008 | 2.31E-006 | myotubularin related protein 2 | protein amino acid dephosphorylation |  | | | | | | | | | | | | | | | | | | | | | | | | | | | |
| 327 | 220892\_s\_at | PSAT1 | 2.668518517 | 7.941629238 | 8.52231435 | 4.15E-008 | 2.32E-006 | phosphoserine aminotransferase 1 | L-serine biosynthesis, metabolism, pyridoxine biosynthesis |  | | | | | | | | | | | | | | | | | | | | | | | | | | | |
| 328 | 203043\_at | ZBED1 | -0.668770057 | 8.101678903 | -8.51056076 | 4.24E-008 | 2.35E-006 | zinc finger, BED domain containing 1 | NA |  | | | | | | | | | | | | | | | | | | | | | | | | | | | |
| 329 | 200760\_s\_at | ARL6IP5 | -1.866152501 | 10.04706765 | -8.51005825 | 4.25E-008 | 2.35E-006 | ADP-ribosylation-like factor 6 interacting protein 5 | NA |  | | | | | | | | | | | | | | | | | | | | | | | | | | | |
| 330 | 213897\_s\_at | MRPL23 | 1.269323719 | 8.116133947 | 8.50156081 | 4.32E-008 | 2.38E-006 | mitochondrial ribosomal protein L23 | protein biosynthesis |  | | | | | | | | | | | | | | | | | | | | | | | | | | | |
| 331 | 204603\_at | EXO1 | 1.127379271 | 7.038876814 | 8.50038787 | 4.32E-008 | 2.38E-006 | exonuclease 1 | DNA recombination, DNA repair, mismatch repair |  | | | | | | | | | | | | | | | | | | | | | | | | | | | |
| 332 | 204133\_at | RNU3IP2 | 1.154891122 | 7.585810161 | 8.49893404 | 4.34E-008 | 2.38E-006 | RNA, U3 small nucleolar interacting protein 2 | rRNA processing |  | | | | | | | | | | | | | | | | | | | | | | | | | | | |
| 333 | 203625\_x\_at | SKP2 | 1.57036314 | 7.486364887 | 8.49563267 | 4.36E-008 | 2.38E-006 | S-phase kinase-associated protein 2 (p45) | G1/S transition of mitotic cell cycle, cell proliferation, regulation of cell cycle, ubiquitin cycle |  | | | | | | | | | | | | | | | | | | | | | | | | | | | |
| 334 | 203816\_at | DGUOK | 0.874568989 | 7.101556894 | 8.4770039 | 4.52E-008 | 2.45E-006 | deoxyguanosine kinase | guanosine metabolism, nucleobase, nucleoside, nucleotide and nucleic acid metabolism |  | | | | | | | | | | | | | | | | | | | | | | | | | | | |
| 335 | 200755\_s\_at | CALU | 1.185582255 | 8.078302905 | 8.46252331 | 4.64E-008 | 2.51E-006 | calumenin | NA |  | | | | | | | | | | | | | | | | | | | | | | | | | | | |
| 336 | 203367\_at | DUSP14 | 1.312908172 | 6.996552943 | 8.46246603 | 4.64E-008 | 2.51E-006 | dual specificity phosphatase 14 | protein amino acid dephosphorylation |  | | | | | | | | | | | | | | | | | | | | | | | | | | | |
| 337 | 204126\_s\_at | CDC45L | 2.16569965 | 7.988392499 | 8.45999784 | 4.67E-008 | 2.51E-006 | CDC45 cell division cycle 45-like (S. cerevisiae) | DNA replication, DNA replication checkpoint, DNA replication initiation, regulation of cell cycle |  | | | | | | | | | | | | | | | | | | | | | | | | | | | |
| 338 | 221520\_s\_at | CDCA8 | 1.360604438 | 7.93350535 | 8.4513547 | 4.74E-008 | 2.53E-006 | cell division cycle associated 8 | cytokinesis |  | | | | | | | | | | | | | | | | | | | | | | | | | | | |
| 339 | 203465\_at | MRPL19 | 1.587089629 | 6.141462298 | 8.45135281 | 4.74E-008 | 2.53E-006 | mitochondrial ribosomal protein L19 | protein biosynthesis |  | | | | | | | | | | | | | | | | | | | | | | | | | | | |
| 340 | 217754\_at | DDX56 | 1.247035971 | 7.941708258 | 8.45057 | 4.75E-008 | 2.53E-006 | DEAD (Asp-Glu-Ala-Asp) box polypeptide 56 | rRNA processing |  | | | | | | | | | | | | | | | | | | | | | | | | | | | |
| 341 | 212985\_at | CCNB1 | 1.024401847 | 5.417316068 | 8.4485288 | 4.77E-008 | 2.53E-006 | Cyclin B1 | G2/M transition of mitotic cell cycle, cytokinesis, mitosis, regulation of cell cycle |  | | | | | | | | | | | | | | | | | | | | | | | | | | | |
| 342 | 200920\_s\_at | BTG1 | -1.797732705 | 10.71793576 | -8.44596248 | 4.79E-008 | 2.54E-006 | B-cell translocation gene 1, anti-proliferative | NA |  | | | | | | | | | | | | | | | | | | | | | | | | | | | |
| 343 | 222217\_s\_at | SLC27A3 | -1.806862646 | 8.07183886 | -8.44554651 | 4.79E-008 | 2.54E-006 | solute carrier family 27 (fatty acid transporter), member 3 | metabolism |  | | | | | | | | | | | | | | | | | | | | | | | | | | | |
| 344 | 208887\_at | EIF3S4 | 1.635603446 | 10.48128691 | 8.44196319 | 4.83E-008 | 2.55E-006 | eukaryotic translation initiation factor 3, subunit 4 delta, 44kDa | protein biosynthesis, regulation of translational initiation |  | | | | | | | | | | | | | | | | | | | | | | | | | | | |
| 345 | 203091\_at | FUBP1 | 0.588358317 | 8.230936585 | 8.43893676 | 4.85E-008 | 2.56E-006 | far upstream element (FUSE) binding protein 1 | phosphate transport, regulation of transcription, DNA-dependent |  | | | | | | | | | | | | | | | | | | | | | | | | | | | |
| 346 | 203032\_s\_at | FH | 1.908381666 | 5.629111774 | 8.43401367 | 4.90E-008 | 2.57E-006 | fumarate hydratase | fumarate metabolism, negative regulation of cell cycle, tricarboxylic acid cycle |  | | | | | | | | | | | | | | | | | | | | | | | | | | | |
| 347 | 201139\_s\_at | SSB | 1.70083355 | 8.189329913 | 8.43201414 | 4.92E-008 | 2.58E-006 | Sjogren syndrome antigen B (autoantigen La) | RNA processing, RNA-nucleus export, histone mRNA metabolism, tRNA modification, transcription from Pol III promoter |  | | | | | | | | | | | | | | | | | | | | | | | | | | | |
| 348 | 202223\_at | ITM1 | 1.402693842 | 7.585253104 | 8.42610109 | 4.97E-008 | 2.60E-006 | integral membrane protein 1 | protein amino acid glycosylation |  | | | | | | | | | | | | | | | | | | | | | | | | | | | |
| 349 | 211284\_s\_at | GRN | -1.930555748 | 10.36647533 | -8.42559794 | 4.98E-008 | 2.60E-006 | granulin | cell proliferation, cell-cell signaling, positive regulation of cell proliferation, signal transduction |  | | | | | | | | | | | | | | | | | | | | | | | | | | | |
| 350 | 207153\_s\_at | GLMN | 0.797482485 | 6.28143247 | 8.42360488 | 5.00E-008 | 2.60E-006 | glomulin, FKBP associated protein | muscle cell differentiation, negative regulation of T-cell proliferation, positive regulation of cytokine secretion, positive regulation of interleukin-2 biosynthesis, positive regulation of phosphorylation, regulation of gene expression, epigenetic, vasculogenesis |  | | | | | | | | | | | | | | | | | | | | | | | | | | | |
| 351 | 203235\_at | THOP1 | 1.188958469 | 6.962525143 | 8.41814482 | 5.05E-008 | 2.62E-006 | thimet oligopeptidase 1 | proteolysis and peptidolysis |  | | | | | | | | | | | | | | | | | | | | | | | | | | | |
| 352 | 202386\_s\_at | LKAP | -1.198905149 | 7.750042065 | -8.41473762 | 5.08E-008 | 2.62E-006 | limkain b1 | NA |  | | | | | | | | | | | | | | | | | | | | | | | | | | | |
| 353 | 211383\_s\_at | WDR37 | -1.007224527 | 7.562558275 | -8.41409562 | 5.09E-008 | 2.62E-006 | WD repeat domain 37 | NA |  | | | | | | | | | | | | | | | | | | | | | | | | | | | |
| 354 | 221550\_at | COX15 | 0.788696767 | 6.260027599 | 8.41325427 | 5.09E-008 | 2.62E-006 | COX15 homolog, cytochrome c oxidase assembly protein (yeast) | NA |  | | | | | | | | | | | | | | | | | | | | | | | | | | | |
| 355 | 202388\_at | RGS2 | -4.739688992 | 10.12569026 | -8.41310905 | 5.10E-008 | 2.62E-006 | regulator of G-protein signalling 2, 24kDa | cell cycle, regulation of G-protein coupled receptor protein signaling pathway, signal transduction |  | | | | | | | | | | | | | | | | | | | | | | | | | | | |
| 356 | 222209\_s\_at | FLJ22104 | 2.584372594 | 6.372306405 | 8.41150039 | 5.11E-008 | 2.62E-006 | hypothetical protein FLJ22104 | NA |  | | | | | | | | | | | | | | | | | | | | | | | | | | | |
| 357 | 202690\_s\_at | SNRPD1 | 2.341037934 | 8.716950172 | 8.41024997 | 5.12E-008 | 2.62E-006 | small nuclear ribonucleoprotein D1 polypeptide 16kDa | NA |  | | | | | | | | | | | | | | | | | | | | | | | | | | | |
| 358 | 212975\_at | KIAA0870 | -1.573800127 | 8.676718734 | -8.40735526 | 5.15E-008 | 2.63E-006 | KIAA0870 protein | NA |  | | | | | | | | | | | | | | | | | | | | | | | | | | | |
| 359 | 208696\_at | CCT5 | 1.466514964 | 10.58965446 | 8.40599843 | 5.16E-008 | 2.63E-006 | chaperonin containing TCP1, subunit 5 (epsilon) | protein folding |  | | | | | | | | | | | | | | | | | | | | | | | | | | | |
| 360 | 220658\_s\_at | ARNTL2 | 1.081364706 | 5.899412451 | 8.40593156 | 5.16E-008 | 2.63E-006 | aryl hydrocarbon receptor nuclear translocator-like 2 | entrainment of circadian clock, regulation of transcription, DNA-dependent, signal transduction |  | | | | | | | | | | | | | | | | | | | | | | | | | | | |
| 361 | 200726\_at | PPP1CC | 1.722570117 | 10.15954299 | 8.40356356 | 5.19E-008 | 2.63E-006 | protein phosphatase 1, catalytic subunit, gamma isoform | cytokinesis, glycogen metabolism |  | | | | | | | | | | | | | | | | | | | | | | | | | | | |
| 362 | 221539\_at | EIF4EBP1 | 1.536856547 | 8.638049806 | 8.40164615 | 5.21E-008 | 2.63E-006 | eukaryotic translation initiation factor 4E binding protein 1 | negative regulation of protein biosynthesis, negative regulation of translational initiation, regulation of translation |  | | | | | | | | | | | | | | | | | | | | | | | | | | | |
| 363 | 34225\_at | WHSC2 | 0.763062569 | 7.466207026 | 8.39886633 | 5.23E-008 | 2.64E-006 | Wolf-Hirschhorn syndrome candidate 2 | regulation of transcription, DNA-dependent |  | | | | | | | | | | | | | | | | | | | | | | | | | | | |
| 364 | 203056\_s\_at | PRDM2 | -1.47496708 | 8.252976568 | -8.38477634 | 5.38E-008 | 2.70E-006 | PR domain containing 2, with ZNF domain | regulation of transcription, DNA-dependent |  | | | | | | | | | | | | | | | | | | | | | | | | | | | |
| 365 | 213365\_at | MGC16943 | 1.365276829 | 6.566343172 | 8.3835399 | 5.39E-008 | 2.70E-006 | similar to RIKEN cDNA 4933424N09 gene | NA |  | | | | | | | | | | | | | | | | | | | | | | | | | | | |
| 366 | 202483\_s\_at | RANBP1 | 2.577144644 | 9.491699884 | 8.3806612 | 5.42E-008 | 2.71E-006 | RAN binding protein 1 | signal transduction |  | | | | | | | | | | | | | | | | | | | | | | | | | | | |
| 367 | 218893\_at | FLJ23469 | 1.189323321 | 8.142443825 | 8.37785327 | 5.45E-008 | 2.72E-006 | hypothetical protein FLJ23469 | metabolism |  | | | | | | | | | | | | | | | | | | | | | | | | | | | |
| 368 | 208264\_s\_at | EIF3S1 | 1.46068659 | 6.549498005 | 8.37373679 | 5.49E-008 | 2.73E-006 | eukaryotic translation initiation factor 3, subunit 1 alpha, 35kDa | protein biosynthesis, regulation of translational initiation |  | | | | | | | | | | | | | | | | | | | | | | | | | | | |
| 369 | 206871\_at | ELA2 | -6.315896171 | 9.943910083 | -8.36781218 | 5.55E-008 | 2.76E-006 | elastase 2, neutrophil | proteolysis and peptidolysis |  | | | | | | | | | | | | | | | | | | | | | | | | | | | |
| 370 | 204977\_at | DDX10 | 1.591454808 | 8.025460129 | 8.36278541 | 5.60E-008 | 2.78E-006 | DEAD (Asp-Glu-Ala-Asp) box polypeptide 10 | NA |  | | | | | | | | | | | | | | | | | | | | | | | | | | | |
| 371 | 209953\_s\_at | CDC37 | 0.81812883 | 8.740503815 | 8.34889036 | 5.75E-008 | 2.84E-006 | CDC37 cell division cycle 37 homolog (S. cerevisiae) | protein folding, protein targeting, regulation of cyclin dependent protein kinase activity |  | | | | | | | | | | | | | | | | | | | | | | | | | | | |
| 372 | 204825\_at | MELK | 1.705781677 | 7.694327172 | 8.34797527 | 5.76E-008 | 2.84E-006 | maternal embryonic leucine zipper kinase | protein amino acid phosphorylation |  | | | | | | | | | | | | | | | | | | | | | | | | | | | |
| 373 | 214431\_at | GMPS | 1.58820664 | 9.089361451 | 8.33231871 | 5.94E-008 | 2.91E-006 | guanine monphosphate synthetase | GMP biosynthesis, biosynthesis, cell growth and/or maintenance, glutamine metabolism, purine base biosynthesis, purine nucleotide biosynthesis |  | | | | | | | | | | | | | | | | | | | | | | | | | | | |
| 374 | 202486\_at | AFG3L2 | 0.915327552 | 8.271520189 | 8.32709589 | 6.00E-008 | 2.93E-006 | AFG3 ATPase family gene 3-like 2 (yeast) | proteolysis and peptidolysis |  | | | | | | | | | | | | | | | | | | | | | | | | | | | |
| 375 | 212978\_at | TA-LRRP | 0.965517341 | 5.851323611 | 8.32127226 | 6.06E-008 | 2.95E-006 | T-cell activation leucine repeat-rich protein | NA |  | | | | | | | | | | | | | | | | | | | | | | | | | | | |
| 376 | 218350\_s\_at | GMNN | 2.274923006 | 7.633088047 | 8.31996738 | 6.08E-008 | 2.95E-006 | geminin, DNA replication inhibitor | cell cycle, cell cycle arrest, negative regulation of DNA replication |  | | | | | | | | | | | | | | | | | | | | | | | | | | | |
| 377 | 204441\_s\_at | POLA2 | 0.937675352 | 7.48357269 | 8.31576703 | 6.13E-008 | 2.97E-006 | polymerase (DNA-directed), alpha (70kD) | DNA replication |  | | | | | | | | | | | | | | | | | | | | | | | | | | | |
| 378 | 209080\_x\_at | TXNL2 | 1.984674094 | 8.556308564 | 8.31505374 | 6.13E-008 | 2.97E-006 | thioredoxin-like 2 | electron transport |  | | | | | | | | | | | | | | | | | | | | | | | | | | | |
| 379 | 202910\_s\_at | CD97 | -1.972755936 | 9.321353312 | -8.3083072 | 6.21E-008 | 2.99E-006 | CD97 antigen | cell adhesion, cell motility, cell-cell signaling, immune response, inflammatory response, neuropeptide signaling pathway |  | | | | | | | | | | | | | | | | | | | | | | | | | | | |
| 380 | 205159\_at | CSF2RB | -3.642886886 | 8.131835026 | -8.30589088 | 6.24E-008 | 2.99E-006 | colony stimulating factor 2 receptor, beta, low-affinity (granulocyte-macrophage) /// colony stimulating factor 2 receptor, beta, low-affinity (granulocyte-macrophage) | antimicrobial humoral response (sensu Vertebrata), cytokine and chemokine mediated signaling pathway, respiratory gaseous exchange, signal transduction |  | | | | | | | | | | | | | | | | | | | | | | | | | | | |
| 381 | 213647\_at | DNA2L | 0.761103241 | 6.052007674 | 8.30579316 | 6.24E-008 | 2.99E-006 | DNA2 DNA replication helicase 2-like (yeast) | NA |  | | | | | | | | | | | | | | | | | | | | | | | | | | | |
| 382 | 217984\_at | RNASET2 | -1.401771068 | 10.59712669 | -8.3051831 | 6.25E-008 | 2.99E-006 | ribonuclease T2 | RNA catabolism |  | | | | | | | | | | | | | | | | | | | | | | | | | | | |
| 383 | 221258\_s\_at | KIF18A | 1.106839496 | 5.03560847 | 8.30476489 | 6.25E-008 | 2.99E-006 | kinesin family member 18A /// kinesin family member 18A | NA |  | | | | | | | | | | | | | | | | | | | | | | | | | | | |
| 384 | 210401\_at | P2RX1 | -1.800418631 | 8.680507994 | -8.30457192 | 6.26E-008 | 2.99E-006 | purinergic receptor P2X, ligand-gated ion channel, 1 | apoptosis, energy pathways, ion transport, signal transduction, synaptic transmission |  | | | | | | | | | | | | | | | | | | | | | | | | | | | |
| 385 | 39402\_at | IL1B | -1.298950465 | 7.561638902 | -8.2963591 | 6.36E-008 | 3.03E-006 | interleukin 1, beta | antimicrobial humoral response (sensu Vertebrata), apoptosis, cell proliferation, cell-cell signaling, immune response, inflammatory response, negative regulation of cell proliferation, regulation of cell cycle, signal transduction |  | | | | | | | | | | | | | | | | | | | | | | | | | | | |
| 386 | 38157\_at | DOM3Z | 0.550345556 | 8.124203355 | 8.29012733 | 6.43E-008 | 3.06E-006 | dom-3 homolog Z (C. elegans) | NA |  | | | | | | | | | | | | | | | | | | | | | | | | | | | |
| 387 | 214045\_at | LIAS | 1.557151704 | 6.423016259 | 8.28621164 | 6.48E-008 | 3.08E-006 | lipoic acid synthetase | lipoate biosynthesis |  | | | | | | | | | | | | | | | | | | | | | | | | | | | |
| 388 | 209056\_s\_at | CDC5L | 1.427059019 | 8.021866232 | 8.27078861 | 6.67E-008 | 3.13E-006 | CDC5 cell division cycle 5-like (S. pombe) | cytokinesis, regulation of transcription, DNA-dependent |  | | | | | | | | | | | | | | | | | | | | | | | | | | | |
| 389 | 218797\_s\_at | SIRT7 | -0.844460254 | 7.928692156 | -8.26933538 | 6.69E-008 | 3.14E-006 | sirtuin (silent mating type information regulation 2 homolog) 7 (S. cerevisiae) | NA |  | | | | | | | | | | | | | | | | | | | | | | | | | | | |
| 390 | 218594\_at | FLJ10359 | 1.534826746 | 8.02384349 | 8.2667622 | 6.72E-008 | 3.14E-006 | Protein BAP28 | NA |  | | | | | | | | | | | | | | | | | | | | | | | | | | | |
| 391 | 204531\_s\_at | BRCA1 | 1.094843613 | 6.822166028 | 8.26620656 | 6.73E-008 | 3.14E-006 | breast cancer 1, early onset | DNA damage response, signal transduction by p53 class mediator resulting in transcription of p21 class mediator, negative regulation of cell cycle, negative regulation of centriole replication, positive regulation of DNA repair, protein ubiquitination, regulation of apoptosis, regulation of cell proliferation, regulation of transcription from Pol II promoter, regulation of transcription from Pol III promoter | | | | | | |  | | | | | | | | | | | | | | | | | | | | | |
| 392 | 204355\_at | DHX30 | 1.303108883 | 8.125976366 | 8.26384956 | 6.76E-008 | 3.15E-006 | DEAH (Asp-Glu-Ala-His) box polypeptide 30 | NA |  | | | | | | | | | | | | | | | | | | | | | | | | | | | |
| 393 | 221827\_at | C20orf18 | 1.382862844 | 9.127393111 | 8.26289955 | 6.77E-008 | 3.15E-006 | chromosome 20 open reading frame 18 | protein ubiquitination |  | | | | | | | | | | | | | | | | | | | | | | | | | | | |
| 394 | 216226\_at | TAF4B | 1.080558547 | 6.200918937 | 8.26028678 | 6.81E-008 | 3.16E-006 | TAF4b RNA polymerase II, TATA box binding protein (TBP)-associated factor, 105kDa | regulation of transcription, DNA-dependent, transcription initiation |  | | | | | | | | | | | | | | | | | | | | | | | | | | | |
| 395 | 204204\_at | SLC31A2 | -2.322287195 | 7.801428828 | -8.2565015 | 6.86E-008 | 3.17E-006 | solute carrier family 31 (copper transporters), member 2 | copper ion transport, transport |  | | | | | | | | | | | | | | | | | | | | | | | | | | | |
| 396 | 208973\_at | PRNPIP | 1.18638281 | 7.07103494 | 8.24693949 | 6.98E-008 | 3.22E-006 | Prion protein interacting protein | NA |  | | | | | | | | | | | | | | | | | | | | | | | | | | | |
| 397 | 213610\_s\_at | MGC22679 | 1.02193352 | 4.612000197 | 8.24142979 | 7.06E-008 | 3.25E-006 | hypothetical protein MGC22679 | metabolism |  | | | | | | | | | | | | | | | | | | | | | | | | | | | |
| 398 | 207076\_s\_at | ASS | 4.235065314 | 8.121772352 | 8.22642798 | 7.26E-008 | 3.31E-006 | argininosuccinate synthetase | arginine biosynthesis, urea cycle |  | | | | | | | | | | | | | | | | | | | | | | | | | | | |
| 399 | 204524\_at | PDPK1 | -1.026367569 | 7.861089704 | -8.22131129 | 7.33E-008 | 3.32E-006 | 3-phosphoinositide dependent protein kinase-1 | actin cytoskeleton organization and biogenesis, insulin receptor signaling pathway, protein amino acid phosphorylation |  | | | | | | | | | | | | | | | | | | | | | | | | | | | |
| 400 | 211615\_s\_at | LRPPRC | 2.482489721 | 8.168562622 | 8.21946627 | 7.36E-008 | 3.32E-006 | leucine-rich PPR-motif containing /// leucine-rich PPR-motif containing | NA |  | | | | | | | | | | | | | | | | | | | | | | | | | | | |
| 401 | 221620\_s\_at | MGC4825 | 1.495200416 | 7.462346442 | 8.21687012 | 7.39E-008 | 3.32E-006 | hypothetical protein MGC4825 | NA |  | | | | | | | | | | | | | | | | | | | | | | | | | | | |
| 402 | 218367\_x\_at | USP21 | 0.643625758 | 7.730061857 | 8.21605601 | 7.41E-008 | 3.32E-006 | ubiquitin specific protease 21 | ubiquitin cycle, ubiquitin-dependent protein catabolism |  | | | | | | | | | | | | | | | | | | | | | | | | | | | |
| 403 | 218698\_at | MMRP19 | 0.945655791 | 7.770452672 | 8.21517632 | 7.42E-008 | 3.32E-006 | likely ortholog of mouse monocyte macrophage 19 | NA |  | | | | | | | | | | | | | | | | | | | | | | | | | | | |
| 404 | 216969\_s\_at | KIF22 | 1.269623689 | 6.778737328 | 8.21471268 | 7.42E-008 | 3.32E-006 | kinesin family member 22 | mitosis |  | | | | | | | | | | | | | | | | | | | | | | | | | | | |
| 405 | 218405\_at | ABT1 | 0.714513424 | 8.18693607 | 8.21409083 | 7.43E-008 | 3.32E-006 | activator of basal transcription 1 | transcription from Pol II promoter |  | | | | | | | | | | | | | | | | | | | | | | | | | | | |
| 406 | 209449\_at | LSM2 | 1.703482711 | 8.541408163 | 8.20542041 | 7.56E-008 | 3.37E-006 | LSM2 homolog, U6 small nuclear RNA associated (S. cerevisiae) | nuclear mRNA splicing, via spliceosome |  | | | | | | | | | | | | | | | | | | | | | | | | | | | |
| 407 | 202810\_at | DRG1 | 1.314987758 | 9.802701991 | 8.1981386 | 7.66E-008 | 3.41E-006 | developmentally regulated GTP binding protein 1 | development, transcription |  | | | | | | | | | | | | | | | | | | | | | | | | | | | |
| 408 | 203135\_at | TBP | 0.769749106 | 7.208604316 | 8.19606368 | 7.69E-008 | 3.42E-006 | TATA box binding protein | regulation of transcription, DNA-dependent, transcription, transcription initiation from Pol II promoter |  | | | | | | | | | | | | | | | | | | | | | | | | | | | |
| 409 | 213021\_at | GOSR1 | 1.03644111 | 6.624434987 | 8.19176084 | 7.76E-008 | 3.44E-006 | golgi SNAP receptor complex member 1 | ER to Golgi transport, intra-Golgi transport, intracellular protein transport |  | | | | | | | | | | | | | | | | | | | | | | | | | | | |
| 410 | 218419\_s\_at | MGC3123 | -0.953336931 | 7.254506653 | -8.18965804 | 7.79E-008 | 3.44E-006 | hypothetical protein MGC3123 | NA |  | | | | | | | | | | | | | | | | | | | | | | | | | | | |
| 411 | 218723\_s\_at | RGC32 | -3.964354909 | 8.391172146 | -8.18892603 | 7.80E-008 | 3.44E-006 | response gene to complement 32 | regulation of cyclin dependent protein kinase activity |  | | | | | | | | | | | | | | | | | | | | | | | | | | | |
| 412 | 203349\_s\_at | ETV5 | 1.905812366 | 7.387145787 | 8.17778244 | 7.97E-008 | 3.51E-006 | ets variant gene 5 (ets-related molecule) | regulation of transcription, DNA-dependent |  | | | | | | | | | | | | | | | | | | | | | | | | | | | |
| 413 | 208922\_s\_at | NXF1 | -1.239788749 | 9.550812208 | -8.17652426 | 7.99E-008 | 3.51E-006 | nuclear RNA export factor 1 | mRNA processing, mRNA-nucleus export, protein-nucleus import, transport |  | | | | | | | | | | | | | | | | | | | | | | | | | | | |
| 414 | 217956\_s\_at | MASA | 1.373581043 | 7.404956719 | 8.16894228 | 8.10E-008 | 3.54E-006 | E-1 enzyme | metabolism |  | | | | | | | | | | | | | | | | | | | | | | | | | | | |
| 415 | 201607\_at | PWP1 | 0.942893928 | 7.310023863 | 8.16879846 | 8.11E-008 | 3.54E-006 | nuclear phosphoprotein similar to S. cerevisiae PWP1 | transcription |  | | | | | | | | | | | | | | | | | | | | | | | | | | | |
| 416 | 202917\_s\_at | S100A8 | -7.33768848 | 10.21795149 | -8.16176856 | 8.22E-008 | 3.57E-006 | S100 calcium binding protein A8 (calgranulin A) | inflammatory response |  | | | | | | | | | | | | | | | | | | | | | | | | | | | |
| 417 | 209063\_x\_at | PAIP1 | 1.43649936 | 7.834473409 | 8.15299037 | 8.36E-008 | 3.63E-006 | poly(A) binding protein interacting protein 1 | protein biosynthesis, regulation of translation |  | | | | | | | | | | | | | | | | | | | | | | | | | | | |
| 418 | 214805\_at | EIF4A1 | -2.64850359 | 8.711033866 | -8.14771254 | 8.44E-008 | 3.65E-006 | Eukaryotic translation initiation factor 4A, isoform 1 | protein biosynthesis |  | | | | | | | | | | | | | | | | | | | | | | | | | | | |
| 419 | 220147\_s\_at | C12orf14 | 1.879199668 | 9.614351066 | 8.13902155 | 8.58E-008 | 3.70E-006 | chromosome 12 open reading frame 14 | NA |  | | | | | | | | | | | | | | | | | | | | | | | | | | | |
| 420 | 219037\_at | CGI-115 | 1.470307033 | 5.652877202 | 8.12504889 | 8.82E-008 | 3.77E-006 | CGI-115 protein | NA |  | | | | | | | | | | | | | | | | | | | | | | | | | | | |
| 421 | 217972\_at | CHCHD3 | 2.097448428 | 8.989540477 | 8.12468094 | 8.82E-008 | 3.77E-006 | coiled-coil-helix-coiled-coil-helix domain containing 3 | NA |  | | | | | | | | | | | | | | | | | | | | | | | | | | | |
| 422 | 205190\_at | PLS1 | 1.134268701 | 6.179789359 | 8.10814996 | 9.11E-008 | 3.88E-006 | plastin 1 (I isoform) | NA |  | | | | | | | | | | | | | | | | | | | | | | | | | | | |
| 423 | 202825\_at | SLC25A4 | 1.24932928 | 7.016715311 | 8.10294381 | 9.20E-008 | 3.91E-006 | solute carrier family 25 (mitochondrial carrier; adenine nucleotide translocator), member 4 | energy pathways, mitochondrial genome maintenance, mitochondrial transport, transport |  | | | | | | | | | | | | | | | | | | | | | | | | | | | |
| 424 | 214657\_s\_at | TncRNA | -2.934775029 | 7.483688692 | -8.10144991 | 9.23E-008 | 3.91E-006 | Trophoblast-derived noncoding RNA | NA |  | | | | | | | | | | | | | | | | | | | | | | | | | | | |
| 425 | 218957\_s\_at | FLJ11848 | 1.362386056 | 7.654300652 | 8.09006679 | 9.43E-008 | 3.99E-006 | hypothetical protein FLJ11848 | NA |  | | | | | | | | | | | | | | | | | | | | | | | | | | | |
| 426 | 211026\_s\_at | MGLL | -1.759944836 | 8.248461249 | -8.08799061 | 9.47E-008 | 4.00E-006 | monoglyceride lipase /// monoglyceride lipase | aromatic compound metabolism, inflammatory response, lipid metabolism |  | | | | | | | | | | | | | | | | | | | | | | | | | | | |
| 427 | 201512\_s\_at | TOMM70A | 1.832872908 | 7.633937123 | 8.08396235 | 9.54E-008 | 4.02E-006 | translocase of outer mitochondrial membrane 70 homolog A (yeast) | NA |  | | | | | | | | | | | | | | | | | | | | | | | | | | | |
| 428 | 36129\_at | RUTBC1 | -0.733990752 | 9.618108808 | -8.07895877 | 9.64E-008 | 4.05E-006 | RUN and TBC1 domain containing 1 | NA |  | | | | | | | | | | | | | | | | | | | | | | | | | | | |
| 429 | 60528\_at | PLA2G4B | -0.915027905 | 8.754297767 | -8.07410931 | 9.73E-008 | 4.07E-006 | phospholipase A2, group IVB (cytosolic) | arachidonic acid metabolism, calcium-mediated signaling, glycerophospholipid catabolism, inflammatory response, parturition |  | | | | | | | | | | | | | | | | | | | | | | | | | | | |
| 430 | 218887\_at | MRPL2 | 1.142031482 | 7.799618132 | 8.07250377 | 9.76E-008 | 4.07E-006 | mitochondrial ribosomal protein L2 | protein biosynthesis |  | | | | | | | | | | | | | | | | | | | | | | | | | | | |
| 431 | 203244\_at | PEX5 | 0.810833653 | 7.77291254 | 8.07150713 | 9.77E-008 | 4.07E-006 | peroxisomal biogenesis factor 5 | protein transport |  | | | | | | | | | | | | | | | | | | | | | | | | | | | |
| 432 | 200071\_at | SMNDC1 | 1.448666805 | 7.918939867 | 8.07126416 | 9.78E-008 | 4.07E-006 | survival motor neuron domain containing 1 /// survival motor neuron domain containing 1 | RNA splicing, apoptosis, induction of apoptosis, nuclear mRNA splicing, via spliceosome |  | | | | | | | | | | | | | | | | | | | | | | | | | | | |
| 433 | 208756\_at | EIF3S2 | 1.599302034 | 9.606193516 | 8.07113512 | 9.78E-008 | 4.07E-006 | eukaryotic translation initiation factor 3, subunit 2 beta, 36kDa | protein biosynthesis, regulation of translational initiation |  | | | | | | | | | | | | | | | | | | | | | | | | | | | |
| 434 | 219418\_at | FLJ12610 | 0.746472572 | 8.111478668 | 8.07060535 | 9.79E-008 | 4.07E-006 | hypothetical protein FLJ12610 | NA |  | | | | | | | | | | | | | | | | | | | | | | | | | | | |
| 435 | 201629\_s\_at | ACP1 | 1.746845967 | 8.090266795 | 8.06886446 | 9.82E-008 | 4.07E-006 | acid phosphatase 1, soluble | protein amino acid dephosphorylation |  | | | | | | | | | | | | | | | | | | | | | | | | | | | |
| 436 | 210983\_s\_at | MCM7 | 2.114310677 | 9.101786788 | 8.06827529 | 9.84E-008 | 4.07E-006 | MCM7 minichromosome maintenance deficient 7 (S. cerevisiae) | DNA replication, DNA replication initiation, cell cycle, regulation of transcription, DNA-dependent |  | | | | | | | | | | | | | | | | | | | | | | | | | | | |
| 437 | 200045\_at | ABCF1 | 0.994304871 | 9.172440788 | 8.06610215 | 9.88E-008 | 4.08E-006 | ATP-binding cassette, sub-family F (GCN20), member 1 /// ATP-binding cassette, sub-family F (GCN20), member 1 | inflammatory response, protein biosynthesis, transport |  | | | | | | | | | | | | | | | | | | | | | | | | | | | |
| 438 | 212144\_at | UNC84B | -1.14505606 | 9.147240177 | -8.06337681 | 9.93E-008 | 4.09E-006 | unc-84 homolog B (C. elegans) | mitotic spindle assembly, nuclear migration |  | | | | | | | | | | | | | | | | | | | | | | | | | | | |
| 439 | 204222\_s\_at | GLIPR1 | -3.514055621 | 8.456201109 | -8.05810767 | 1.00E-007 | 4.13E-006 | GLI pathogenesis-related 1 (glioma) | NA |  | | | | | | | | | | | | | | | | | | | | | | | | | | | |
| 440 | 212443\_at | KIAA0540 | -2.214891086 | 8.337679101 | -8.05608922 | 1.01E-007 | 4.14E-006 | KIAA0540 protein | NA |  | | | | | | | | | | | | | | | | | | | | | | | | | | | |
| 441 | 221677\_s\_at | DONSON | 1.590048618 | 6.626298083 | 8.05344746 | 1.01E-007 | 4.15E-006 | downstream neighbor of SON | NA |  | | | | | | | | | | | | | | | | | | | | | | | | | | | |
| 442 | 201145\_at | HAX1 | 1.673321039 | 9.389179976 | 8.03385965 | 1.05E-007 | 4.29E-006 | HS1 binding protein | NA |  | | | | | | | | | | | | | | | | | | | | | | | | | | | |
| 443 | 213687\_s\_at | RPL35A | 1.236386392 | 11.41879874 | 8.0270935 | 1.07E-007 | 4.34E-006 | ribosomal protein L35a | protein biosynthesis |  | | | | | | | | | | | | | | | | | | | | | | | | | | | |
| 444 | 203358\_s\_at | EZH2 | 2.139072368 | 7.265669645 | 8.02614406 | 1.07E-007 | 4.34E-006 | enhancer of zeste homolog 2 (Drosophila) | G-protein coupled receptor protein signaling pathway, establishment and/or maintenance of chromatin architecture, regulation of transcription, DNA-dependent |  | | | | | | | | | | | | | | | | | | | | | | | | | | | |
| 445 | 200019\_s\_at | FAU | 0.668353217 | 12.19111775 | 8.01382493 | 1.09E-007 | 4.44E-006 | Finkel-Biskis-Reilly murine sarcoma virus (FBR-MuSV) ubiquitously expressed (fox derived); ribosomal protein S30 /// Finkel-Biskis-Reilly murine sarcoma virus (FBR-MuSV) ubiquitously expressed (fox derived); ribosomal protein S30 | NA |  | | | | | | | | | | | | | | | | | | | | | | | | | | | |
| 446 | 203119\_at | MGC2574 | 1.448292496 | 8.084630698 | 8.00363402 | 1.11E-007 | 4.51E-006 | hypothetical protein MGC2574 | NA |  | | | | | | | | | | | | | | | | | | | | | | | | | | | |
| 447 | 218801\_at | UGCGL2 | 1.054853261 | 5.5164494 | 8.0018989 | 1.12E-007 | 4.52E-006 | UDP-glucose ceramide glucosyltransferase-like 2 | protein amino acid glycosylation |  | | | | | | | | | | | | | | | | | | | | | | | | | | | |
| 448 | 202779\_s\_at | UBE2S | 2.190893943 | 9.28671984 | 7.99133666 | 1.14E-007 | 4.60E-006 | ubiquitin-conjugating enzyme E2S | ubiquitin cycle |  | | | | | | | | | | | | | | | | | | | | | | | | | | | |
| 449 | 219807\_x\_at | RAB4B | -0.746505411 | 8.355914013 | -7.97547579 | 1.18E-007 | 4.74E-006 | RAB4B, member RAS oncogene family | NA |  | | | | | | | | | | | | | | | | | | | | | | | | | | | |
| 450 | 221772\_s\_at | PPP2R2D | 0.877777205 | 7.805009291 | 7.97284371 | 1.18E-007 | 4.75E-006 | protein phosphatase 2, regulatory subunit B, delta isoform | signal transduction |  | | | | | | | | | | | | | | | | | | | | | | | | | | | |
| 451 | 221437\_s\_at | MRPS15 | 1.884474688 | 7.730602072 | 7.97059752 | 1.19E-007 | 4.77E-006 | mitochondrial ribosomal protein S15 /// mitochondrial ribosomal protein S15 | protein biosynthesis |  | | | | | | | | | | | | | | | | | | | | | | | | | | | |
| 452 | 219336\_s\_at | ASCC1 | 0.923514506 | 7.004958192 | 7.96502493 | 1.20E-007 | 4.81E-006 | activating signal cointegrator 1 complex subunit 1 | regulation of transcription, DNA-dependent |  | | | | | | | | | | | | | | | | | | | | | | | | | | | |
| 453 | 216251\_s\_at | KIAA0153 | 0.981129618 | 8.319803454 | 7.95676263 | 1.22E-007 | 4.88E-006 | KIAA0153 protein | protein modification |  | | | | | | | | | | | | | | | | | | | | | | | | | | | |
| 454 | 219350\_s\_at | DIABLO | 0.984660767 | 9.200961672 | 7.95015877 | 1.24E-007 | 4.92E-006 | diablo homolog (Drosophila) | apoptosis, caspase activation via cytochrome c, induction of apoptosis, induction of apoptosis via death domain receptors |  | | | | | | | | | | | | | | | | | | | | | | | | | | | |
| 455 | 212676\_at | NF1 | 1.123548307 | 6.747183721 | 7.94946925 | 1.24E-007 | 4.92E-006 | Neurofibromin 1 (neurofibromatosis, von Recklinghausen disease, Watson disease) | Ras protein signal transduction, cell growth and/or maintenance, negative regulation of cell cycle, negative regulation of cell proliferation |  | | | | | | | | | | | | | | | | | | | | | | | | | | | |
| 456 | 205545\_x\_at | DNAJC8 | 0.827100841 | 9.493795659 | 7.94028668 | 1.26E-007 | 4.99E-006 | DnaJ (Hsp40) homolog, subfamily C, member 8 | protein folding |  | | | | | | | | | | | | | | | | | | | | | | | | | | | |
| 457 | 201390\_s\_at | CSNK2B | 1.204298966 | 10.58902615 | 7.92918481 | 1.29E-007 | 5.09E-006 | casein kinase 2, beta polypeptide | I-kappaB kinase/NF-kappaB cascade, positive regulation of Wnt receptor signaling pathway, signal transduction |  | | | | | | | | | | | | | | | | | | | | | | | | | | | |
| 458 | 216422\_at | PA2G4 | 0.626299612 | 5.329116106 | 7.92758046 | 1.29E-007 | 5.10E-006 | proliferation-associated 2G4, 38kDa | cell cycle arrest, cell proliferation, proteolysis and peptidolysis |  | | | | | | | | | | | | | | | | | | | | | | | | | | | |
| 459 | 200862\_at | DHCR24 | 2.053620884 | 8.162269672 | 7.9221807 | 1.31E-007 | 5.13E-006 | 24-dehydrocholesterol reductase | cholesterol biosynthesis, electron transport |  | | | | | | | | | | | | | | | | | | | | | | | | | | | |
| 460 | 209354\_at | TNFRSF14 | -1.150716696 | 6.877246554 | -7.9219457 | 1.31E-007 | 5.13E-006 | tumor necrosis factor receptor superfamily, member 14 (herpesvirus entry mediator) | apoptosis, cell surface receptor linked signal transduction, immune response |  | | | | | | | | | | | | | | | | | | | | | | | | | | | |
| 461 | 201173\_x\_at | NUDC | 1.258485778 | 9.413518445 | 7.91421469 | 1.33E-007 | 5.20E-006 | nuclear distribution gene C homolog (A. nidulans) | cell proliferation, development, regulation of cell cycle |  | | | | | | | | | | | | | | | | | | | | | | | | | | | |
| 462 | 200888\_s\_at | RPL23 | 0.827182173 | 12.08072058 | 7.90416289 | 1.35E-007 | 5.29E-006 | ribosomal protein L23 | NA |  | | | | | | | | | | | | | | | | | | | | | | | | | | | |
| 463 | 219997\_s\_at | COPS7B | 0.758921847 | 7.87429239 | 7.90358411 | 1.35E-007 | 5.29E-006 | COP9 constitutive photomorphogenic homolog subunit 7B (Arabidopsis) | NA |  | | | | | | | | | | | | | | | | | | | | | | | | | | | |
| 464 | 211316\_x\_at | CFLAR | -1.980954237 | 8.659703644 | -7.90029562 | 1.36E-007 | 5.31E-006 | CASP8 and FADD-like apoptosis regulator | anti-apoptosis, induction of apoptosis by extracellular signals, positive regulation of I-kappaB kinase/NF-kappaB cascade, proteolysis and peptidolysis, regulation of apoptosis |  | | | | | | | | | | | | | | | | | | | | | | | | | | | |
| 465 | 211220\_s\_at | HSF2 | 0.689713238 | 4.500001152 | 7.89878738 | 1.37E-007 | 5.32E-006 | heat shock transcription factor 2 | protein folding, regulation of transcription, DNA-dependent, response to unfolded protein, transcription from Pol II promoter |  | | | | | | | | | | | | | | | | | | | | | | | | | | | |
| 466 | 217790\_s\_at | SSR3 | 1.601204771 | 5.99847628 | 7.89673385 | 1.37E-007 | 5.33E-006 | signal sequence receptor, gamma (translocon-associated protein gamma) | cotranslational protein-membrane targeting |  | | | | | | | | | | | | | | | | | | | | | | | | | | | |
| 467 | 217427\_s\_at | HIRA | 0.993496499 | 8.088653937 | 7.88718494 | 1.40E-007 | 5.42E-006 | HIR histone cell cycle regulation defective homolog A (S. cerevisiae) | morphogenesis, regulation of transcription from Pol II promoter |  | | | | | | | | | | | | | | | | | | | | | | | | | | | |
| 468 | 213124\_at | ZNF473 | 0.641588743 | 6.018449224 | 7.86691108 | 1.45E-007 | 5.62E-006 | zinc finger protein 473 | NA |  | | | | | | | | | | | | | | | | | | | | | | | | | | | |
| 469 | 204294\_at | AMT | -0.774886442 | 7.648541385 | -7.84685221 | 1.51E-007 | 5.81E-006 | aminomethyltransferase (glycine cleavage system protein T) | glycine catabolism |  | | | | | | | | | | | | | | | | | | | | | | | | | | | |
| 470 | 202022\_at | ALDOC | 1.182184788 | 7.53409615 | 7.83804014 | 1.54E-007 | 5.91E-006 | aldolase C, fructose-bisphosphate | fructose metabolism, glycolysis |  | | | | | | | | | | | | | | | | | | | | | | | | | | | |
| 471 | 202983\_at | SMARCA3 | 2.401094765 | 7.460726961 | 7.83458349 | 1.55E-007 | 5.93E-006 | SWI/SNF related, matrix associated, actin dependent regulator of chromatin, subfamily a, member 3 | protein ubiquitination, regulation of transcription, DNA-dependent |  | | | | | | | | | | | | | | | | | | | | | | | | | | | |
| 472 | 210691\_s\_at | SIP | 1.99479942 | 6.343086293 | 7.83204403 | 1.56E-007 | 5.94E-006 | Siah-interacting protein | NA |  | | | | | | | | | | | | | | | | | | | | | | | | | | | |
| 473 | 210580\_x\_at | SULT1A3 | -1.399307822 | 9.002889988 | -7.82943888 | 1.57E-007 | 5.97E-006 | sulfotransferase family, cytosolic, 1A, phenol-preferring, member 3 | catecholamine metabolism, steroid metabolism, synaptic transmission |  | | | | | | | | | | | | | | | | | | | | | | | | | | | |
| 474 | 204853\_at | ORC2L | 0.995162524 | 7.000307842 | 7.82462909 | 1.58E-007 | 5.99E-006 | origin recognition complex, subunit 2-like (yeast) | DNA replication, DNA replication initiation, negative regulation of transcription from Pol II promoter |  | | | | | | | | | | | | | | | | | | | | | | | | | | | |
| 475 | 203195\_s\_at | NUP98 | 0.614408788 | 7.567066137 | 7.81554455 | 1.61E-007 | 6.08E-006 | nucleoporin 98kDa | DNA replication, nuclear pore organization and biogenesis, nucleocytoplasmic transport, protein transport, protein-nucleus import, docking |  | | | | | | | | | | | | | | | | | | | | | | | | | | | |
| 476 | 221480\_at | HNRPD | 0.62720793 | 4.934983624 | 7.81284574 | 1.62E-007 | 6.08E-006 | heterogeneous nuclear ribonucleoprotein D (AU-rich element RNA binding protein 1, 37kDa) | RNA catabolism, RNA processing, mRNA catabolism, regulation of transcription, DNA-dependent, telomerase-dependent telomere maintenance |  | | | | | | | | | | | | | | | | | | | | | | | | | | | |
| 477 | 205690\_s\_at | G10 | 1.273682452 | 8.925876628 | 7.81284179 | 1.62E-007 | 6.08E-006 | maternal G10 transcript | regulation of transcription from Pol II promoter |  | | | | | | | | | | | | | | | | | | | | | | | | | | | |
| 478 | 212610\_at | PTPN11 | 1.453784507 | 8.664792419 | 7.79876958 | 1.66E-007 | 6.23E-006 | protein tyrosine phosphatase, non-receptor type 11 (Noonan syndrome 1) | intracellular signaling cascade, perception of sound, protein amino acid dephosphorylation |  | | | | | | | | | | | | | | | | | | | | | | | | | | | |
| 479 | 213305\_s\_at | PPP2R5C | 1.130616312 | 7.057969965 | 7.79477421 | 1.68E-007 | 6.27E-006 | protein phosphatase 2, regulatory subunit B (B56), gamma isoform | signal transduction |  | | | | | | | | | | | | | | | | | | | | | | | | | | | |
| 480 | 201614\_s\_at | RUVBL1 | 1.709216937 | 8.120370392 | 7.7930185 | 1.68E-007 | 6.28E-006 | RuvB-like 1 (E. coli) | DNA recombination, regulation of transcription from Pol II promoter, spermatogenesis, transcription |  | | | | | | | | | | | | | | | | | | | | | | | | | | | |
| 481 | 205644\_s\_at | SNRPG | 1.58406974 | 9.358710982 | 7.78368251 | 1.71E-007 | 6.38E-006 | small nuclear ribonucleoprotein polypeptide G | NA |  | | | | | | | | | | | | | | | | | | | | | | | | | | | |
| 482 | 219258\_at | FLJ20516 | 1.278024449 | 6.06483123 | 7.77537962 | 1.74E-007 | 6.48E-006 | timeless-interacting protein | NA |  | | | | | | | | | | | | | | | | | | | | | | | | | | | |
| 483 | 215127\_s\_at | RBMS1 | -1.329676836 | 8.354586377 | -7.7715788 | 1.75E-007 | 6.50E-006 | RNA binding motif, single stranded interacting protein 1 | DNA replication, RNA processing, regulation of translation |  | | | | | | | | | | | | | | | | | | | | | | | | | | | |
| 484 | 212626\_x\_at | HNRPC | 0.771361013 | 10.2546722 | 7.77095233 | 1.76E-007 | 6.50E-006 | heterogeneous nuclear ribonucleoprotein C (C1/C2) | RNA splicing |  | | | | | | | | | | | | | | | | | | | | | | | | | | | |
| 485 | 217988\_at | CCNB1IP1 | 2.568804291 | 8.80861584 | 7.77075978 | 1.76E-007 | 6.50E-006 | cyclin B1 interacting protein 1 | ubiquitin cycle |  | | | | | | | | | | | | | | | | | | | | | | | | | | | |
| 486 | 213046\_at | PABPN1 | -1.719648126 | 8.284137674 | -7.76837541 | 1.77E-007 | 6.52E-006 | poly(A) binding protein, nuclear 1 | NA |  | | | | | | | | | | | | | | | | | | | | | | | | | | | |
| 487 | 211603\_s\_at | ETV4 | 0.846957497 | 7.37332008 | 7.76442561 | 1.78E-007 | 6.55E-006 | ets variant gene 4 (E1A enhancer binding protein, E1AF) /// ets variant gene 4 (E1A enhancer binding protein, E1AF) | regulation of transcription, DNA-dependent |  | | | | | | | | | | | | | | | | | | | | | | | | | | | |
| 488 | 201458\_s\_at | BUB3 | 1.477336305 | 8.388168382 | 7.74859317 | 1.84E-007 | 6.73E-006 | BUB3 budding uninhibited by benzimidazoles 3 homolog (yeast) | cell proliferation, mitosis, mitotic spindle checkpoint |  | | | | | | | | | | | | | | | | | | | | | | | | | | | |
| 489 | 200659\_s\_at | PHB | 0.812598264 | 8.343069134 | 7.74686079 | 1.84E-007 | 6.74E-006 | prohibitin | DNA metabolism, cell growth and/or maintenance, histone deacetylation, negative regulation of cell proliferation, negative regulation of transcription, protein biosynthesis, regulation of cell cycle |  | | | | | | | | | | | | | | | | | | | | | | | | | | | |
| 490 | 201267\_s\_at | PSMC3 | 1.355658529 | 8.972724242 | 7.73657714 | 1.88E-007 | 6.87E-006 | proteasome (prosome, macropain) 26S subunit, ATPase, 3 | protein catabolism |  | | | | | | | | | | | | | | | | | | | | | | | | | | | |
| 491 | 201177\_s\_at | UBA2 | 1.991444564 | 8.031644066 | 7.73350025 | 1.89E-007 | 6.89E-006 | SUMO-1 activating enzyme subunit 2 | ubiquitin cycle |  | | | | | | | | | | | | | | | | | | | | | | | | | | | |
| 492 | 203067\_at | PDHX | 1.345763912 | 7.56358527 | 7.73178835 | 1.90E-007 | 6.90E-006 | pyruvate dehydrogenase complex, component X | metabolism |  | | | | | | | | | | | | | | | | | | | | | | | | | | | |
| 493 | 218152\_at | HMG20A | 1.387315635 | 6.246666976 | 7.72918917 | 1.91E-007 | 6.91E-006 | high-mobility group 20A | establishment and/or maintenance of chromatin architecture, regulation of transcription, DNA-dependent |  | | | | | | | | | | | | | | | | | | | | | | | | | | | |
| 494 | 218393\_s\_at | SMU1 | 0.682470562 | 7.013552853 | 7.72394996 | 1.93E-007 | 6.97E-006 | smu-1 suppressor of mec-8 and unc-52 homolog (C. elegans) | NA |  | | | | | | | | | | | | | | | | | | | | | | | | | | | |
| 495 | 220990\_s\_at | VMP1 | -1.469994163 | 9.546673957 | -7.71187984 | 1.97E-007 | 7.13E-006 | likely ortholog of rat vacuole membrane protein 1 /// likely ortholog of rat vacuole membrane protein 1 | NA |  | | | | | | | | | | | | | | | | | | | | | | | | | | | |
| 496 | 201796\_s\_at | VARS2 | 0.858246507 | 8.5083505 | 7.70979866 | 1.98E-007 | 7.15E-006 | valyl-tRNA synthetase 2 | protein biosynthesis, translational elongation, valyl-tRNA aminoacylation |  | | | | | | | | | | | | | | | | | | | | | | | | | | | |
| 497 | 218682\_s\_at | SLC4A1AP | 1.05722157 | 7.238810394 | 7.70421879 | 2.00E-007 | 7.21E-006 | solute carrier family 4 (anion exchanger), member 1, adaptor protein | NA |  | | | | | | | | | | | | | | | | | | | | | | | | | | | |
| 498 | 201609\_x\_at | ICMT | 0.806178886 | 8.213184381 | 7.70147585 | 2.01E-007 | 7.24E-006 | isoprenylcysteine carboxyl methyltransferase | C-terminal protein amino acid methylation, protein modification, protein-membrane targeting |  | | | | | | | | | | | | | | | | | | | | | | | | | | | |
| 499 | 208949\_s\_at | GALIG /// LGALS3 | -3.183118414 | 9.565748442 | -7.70085085 | 2.02E-007 | 7.24E-006 | galectin-3 internal gene /// lectin, galactoside-binding, soluble, 3 (galectin 3) | NA |  | | | | | | | | | | | | | | | | | | | | | | | | | | | |
| 500 | 222163\_s\_at | SPATA5L1 | 1.538591263 | 6.846833588 | 7.7000927 | 2.02E-007 | 7.24E-006 | spermatogenesis associated 5-like 1 | cytokinesis |  | | | | | | | | | | | | | | | | | | | | | | | | | | | |
| 501 | 218561\_s\_at | C6orf149 | 2.215337783 | 8.044252929 | 7.69808162 | 2.03E-007 | 7.24E-006 | chromosome 6 open reading frame 149 | electron transport |  | | | | | | | | | | | | | | | | | | | | | | | | | | | |
| 502 | 208610\_s\_at | SRRM2 | -1.471171748 | 9.614099974 | -7.68639428 | 2.08E-007 | 7.38E-006 | serine/arginine repetitive matrix 2 | NA |  | | | | | | | | | | | | | | | | | | | | | | | | | | | |
| 503 | 218235\_s\_at | CGI-94 | 1.509063344 | 8.648385153 | 7.68456124 | 2.08E-007 | 7.39E-006 | comparative gene identification transcript 94 | rRNA processing |  | | | | | | | | | | | | | | | | | | | | | | | | | | | |
| 504 | 218826\_at | SLC35F2 | 1.701977738 | 8.05275994 | 7.68162033 | 2.10E-007 | 7.42E-006 | solute carrier family 35, member F2 | NA |  | | | | | | | | | | | | | | | | | | | | | | | | | | | |
| 505 | 218080\_x\_at | FAF1 | 1.367427629 | 8.608458585 | 7.68127141 | 2.10E-007 | 7.42E-006 | Fas (TNFRSF6) associated factor 1 | apoptosis |  | | | | | | | | | | | | | | | | | | | | | | | | | | | |
| 506 | 204087\_s\_at | SLC5A6 | 1.008743582 | 8.249781579 | 7.67293503 | 2.13E-007 | 7.52E-006 | solute carrier family 5 (sodium-dependent vitamin transporter), member 6 | sodium ion transport, transport |  | | | | | | | | | | | | | | | | | | | | | | | | | | | |
| 507 | 217947\_at | CKLFSF6 | -1.32519452 | 10.46278355 | -7.66960834 | 2.15E-007 | 7.55E-006 | chemokine-like factor super family 6 | chemotaxis |  | | | | | | | | | | | | | | | | | | | | | | | | | | | |
| 508 | 202721\_s\_at | GFPT1 | 1.136073008 | 7.38667506 | 7.65935511 | 2.19E-007 | 7.69E-006 | glutamine-fructose-6-phosphate transaminase 1 | carbohydrate biosynthesis, energy reserve metabolism, fructose 6-phosphate metabolism, glutamine metabolism, metabolism |  | | | | | | | | | | | | | | | | | | | | | | | | | | | |
| 509 | 201833\_at | HDAC2 | 1.757450833 | 7.971675649 | 7.65919521 | 2.19E-007 | 7.69E-006 | histone deacetylase 2 | NA |  | | | | | | | | | | | | | | | | | | | | | | | | | | | |
| 510 | 212018\_s\_at | DKFZP564M182 | 2.108537805 | 8.790615278 | 7.65026483 | 2.23E-007 | 7.81E-006 | DKFZP564M182 protein | protein biosynthesis |  | | | | | | | | | | | | | | | | | | | | | | | | | | | |
| 511 | 200691\_s\_at | HSPA9B | 2.07966316 | 9.316192192 | 7.6364021 | 2.29E-007 | 8.02E-006 | heat shock 70kDa protein 9B (mortalin-2) | protein folding |  | | | | | | | | | | | | | | | | | | | | | | | | | | | |
| 512 | 219244\_s\_at | MRPL46 | 1.20526583 | 7.915117019 | 7.62617876 | 2.34E-007 | 8.17E-006 | mitochondrial ribosomal protein L46 | NA |  | | | | | | | | | | | | | | | | | | | | | | | | | | | |
| 513 | 203160\_s\_at | RNF8 | 0.958811615 | 7.216441864 | 7.61988087 | 2.37E-007 | 8.23E-006 | ring finger protein (C3HC4 type) 8 | protein ubiquitination |  | | | | | | | | | | | | | | | | | | | | | | | | | | | |
| 514 | 204175\_at | ZNF593 | 1.957194612 | 7.878801739 | 7.6189526 | 2.37E-007 | 8.24E-006 | zinc finger protein 593 | negative regulation of transcription from Pol II promoter, regulation of transcription, DNA-dependent |  | | | | | | | | | | | | | | | | | | | | | | | | | | | |
| 515 | 218842\_at | FLJ21908 | 1.907933266 | 5.775776708 | 7.61519613 | 2.39E-007 | 8.29E-006 | hypothetical protein FLJ21908 | NA |  | | | | | | | | | | | | | | | | | | | | | | | | | | | |
| 516 | 220028\_at | ACVR2B | 0.925059482 | 6.80629773 | 7.61171175 | 2.41E-007 | 8.31E-006 | activin A receptor, type IIB | protein amino acid phosphorylation, transmembrane receptor protein serine/threonine kinase signaling pathway |  | | | | | | | | | | | | | | | | | | | | | | | | | | | |
| 517 | 52169\_at | LYK5 | -0.49253402 | 8.804692865 | -7.6113499 | 2.41E-007 | 8.31E-006 | protein kinase LYK5 | protein amino acid phosphorylation |  | | | | | | | | | | | | | | | | | | | | | | | | | | | |
| 518 | 207507\_s\_at | ATP5G3 | 1.802013502 | 9.95402512 | 7.60997706 | 2.42E-007 | 8.32E-006 | ATP synthase, H+ transporting, mitochondrial F0 complex, subunit c (subunit 9) isoform 3 | ATP synthesis coupled proton transport, energy pathways, proton transport |  | | | | | | | | | | | | | | | | | | | | | | | | | | | |
| 519 | 209157\_at | DNAJA2 | 1.482121314 | 7.51456404 | 7.60827089 | 2.42E-007 | 8.34E-006 | DnaJ (Hsp40) homolog, subfamily A, member 2 | G1 phase of mitotic cell cycle, positive regulation of cell proliferation, protein folding, regulation of cell cycle |  | | | | | | | | | | | | | | | | | | | | | | | | | | | |
| 520 | 212955\_s\_at | POLR2I | 1.225161987 | 8.187505858 | 7.60433454 | 2.44E-007 | 8.39E-006 | polymerase (RNA) II (DNA directed) polypeptide I, 14.5kDa | RNA elongation, regulation of transcription, DNA-dependent, transcription, transcription from Pol II promoter |  | | | | | | | | | | | | | | | | | | | | | | | | | | | |
| 521 | 213107\_at | TNIK | 0.823495117 | 6.867779351 | 7.6037127 | 2.45E-007 | 8.39E-006 | TRAF2 and NCK interacting kinase | protein amino acid phosphorylation, protein kinase cascade, regulation of translation, response to stress |  | | | | | | | | | | | | | | | | | | | | | | | | | | | |
| 522 | 216231\_s\_at | B2M | -1.101000062 | 12.99521613 | -7.59971622 | 2.47E-007 | 8.44E-006 | beta-2-microglobulin | antigen presentation, endogenous antigen, antigen processing, endogenous antigen via MHC class I, immune response |  | | | | | | | | | | | | | | | | | | | | | | | | | | | |
| 523 | 202105\_at | IGBP1 | 1.085442384 | 9.273637984 | 7.59838348 | 2.47E-007 | 8.45E-006 | immunoglobulin (CD79A) binding protein 1 | B-cell activation, regulation of signal transduction, response to biotic stimulus |  | | | | | | | | | | | | | | | | | | | | | | | | | | | |
| 524 | 200894\_s\_at | FKBP4 | 1.349591985 | 7.573912727 | 7.59551428 | 2.49E-007 | 8.48E-006 | FK506 binding protein 4, 59kDa | protein folding |  | | | | | | | | | | | | | | | | | | | | | | | | | | | |
| 525 | 212162\_at | KIDINS220 | -0.84047704 | 7.739164036 | -7.59446487 | 2.49E-007 | 8.48E-006 | Likely homolog of rat kinase D-interacting substance of 220 kDa | NA |  | | | | | | | | | | | | | | | | | | | | | | | | | | | |
| 526 | 206621\_s\_at | WBSCR1 | 0.856467797 | 10.20462252 | 7.59392695 | 2.49E-007 | 8.48E-006 | Williams-Beuren syndrome chromosome region 1 | protein biosynthesis, regulation of translational initiation |  | | | | | | | | | | | | | | | | | | | | | | | | | | | |
| 527 | 200925\_at | COX6A1 | 0.949258413 | 10.94927406 | 7.5911393 | 2.51E-007 | 8.52E-006 | cytochrome c oxidase subunit VIa polypeptide 1 | electron transport, energy pathways |  | | | | | | | | | | | | | | | | | | | | | | | | | | | |
| 528 | 203213\_at | CDC2 | 2.595716075 | 6.896043786 | 7.58828569 | 2.52E-007 | 8.55E-006 | cell division cycle 2, G1 to S and G2 to M | cytokinesis, mitosis, protein amino acid phosphorylation, traversing start control point of mitotic cell cycle |  | | | | | | | | | | | | | | | | | | | | | | | | | | | |
| 529 | 202468\_s\_at | CTNNAL1 | 1.592667203 | 7.640694041 | 7.57761687 | 2.58E-007 | 8.71E-006 | catenin (cadherin-associated protein), alpha-like 1 | apoptosis |  | | | | | | | | | | | | | | | | | | | | | | | | | | | |
| 530 | 200002\_at | RPL35 | 1.160883116 | 11.80935631 | 7.5752587 | 2.59E-007 | 8.73E-006 | ribosomal protein L35 /// ribosomal protein L35 | protein biosynthesis |  | | | | | | | | | | | | | | | | | | | | | | | | | | | |
| 531 | 218763\_at | STX18 | 0.653348299 | 7.871390526 | 7.57509845 | 2.59E-007 | 8.73E-006 | syntaxin 18 | ER to Golgi transport, protein transport, vesicle docking during exocytosis |  | | | | | | | | | | | | | | | | | | | | | | | | | | | |
| 532 | 205061\_s\_at | EXOSC9 | 0.88269221 | 8.161542685 | 7.57241795 | 2.60E-007 | 8.76E-006 | exosome component 9 | immune response, rRNA processing |  | | | | | | | | | | | | | | | | | | | | | | | | | | | |
| 533 | 201624\_at | DARS | 2.042268177 | 7.336233277 | 7.56955472 | 2.62E-007 | 8.77E-006 | aspartyl-tRNA synthetase | aspartyl-tRNA aminoacylation, protein biosynthesis, protein complex assembly |  | | | | | | | | | | | | | | | | | | | | | | | | | | | |
| 534 | 204744\_s\_at | IARS | 2.416745253 | 9.679748526 | 7.56916824 | 2.62E-007 | 8.77E-006 | isoleucine-tRNA synthetase | isoleucyl-tRNA aminoacylation, protein biosynthesis |  | | | | | | | | | | | | | | | | | | | | | | | | | | | |
| 535 | 208714\_at | NDUFV1 | 1.499007497 | 9.714917614 | 7.56543877 | 2.64E-007 | 8.78E-006 | NADH dehydrogenase (ubiquinone) flavoprotein 1, 51kDa | energy pathways, mitochondrial electron transport, NADH to ubiquinone |  | | | | | | | | | | | | | | | | | | | | | | | | | | | |
| 536 | 201873\_s\_at | ABCE1 | 1.994813194 | 7.295481182 | 7.55624873 | 2.69E-007 | 8.92E-006 | ATP-binding cassette, sub-family E (OABP), member 1 | NA |  | | | | | | | | | | | | | | | | | | | | | | | | | | | |
| 537 | 219555\_s\_at | BM039 | 1.900613129 | 7.411166274 | 7.5530692 | 2.71E-007 | 8.95E-006 | uncharacterized bone marrow protein BM039 | NA |  | | | | | | | | | | | | | | | | | | | | | | | | | | | |
| 538 | 201052\_s\_at | PSMF1 | 0.826720627 | 8.462422682 | 7.55200633 | 2.71E-007 | 8.95E-006 | proteasome (prosome, macropain) inhibitor subunit 1 (PI31) | ubiquitin-dependent protein catabolism |  | | | | | | | | | | | | | | | | | | | | | | | | | | | |
| 539 | 200661\_at | PPGB | -2.060331601 | 9.929608542 | -7.538621 | 2.79E-007 | 9.17E-006 | protective protein for beta-galactosidase (galactosialidosis) | intracellular protein transport, proteolysis and peptidolysis |  | | | | | | | | | | | | | | | | | | | | | | | | | | | |
| 540 | 200681\_at | GLO1 | 2.043959883 | 9.528079911 | 7.53616145 | 2.80E-007 | 9.20E-006 | glyoxalase I | carbohydrate metabolism |  | | | | | | | | | | | | | | | | | | | | | | | | | | | |
| 541 | 209247\_s\_at | ABCF2 | 1.701237761 | 8.050703767 | 7.52270543 | 2.88E-007 | 9.43E-006 | ATP-binding cassette, sub-family F (GCN20), member 2 | DNA recombination, DNA repair, DNA replication, transport |  | | | | | | | | | | | | | | | | | | | | | | | | | | | |
| 542 | 218314\_s\_at | FLJ10726 | 1.039341554 | 7.387737101 | 7.52097282 | 2.89E-007 | 9.44E-006 | hypothetical protein FLJ10726 | NA |  | | | | | | | | | | | | | | | | | | | | | | | | | | | |
| 543 | 210216\_x\_at | RAD1 | 0.97765021 | 7.643428535 | 7.51438816 | 2.92E-007 | 9.55E-006 | RAD1 homolog (S. pombe) | DNA repair, cell cycle checkpoint |  | | | | | | | | | | | | | | | | | | | | | | | | | | | |
| 544 | 210466\_s\_at | PAI-RBP1 | 1.303813819 | 9.655445037 | 7.50781099 | 2.96E-007 | 9.65E-006 | PAI-1 mRNA-binding protein | NA |  | | | | | | | | | | | | | | | | | | | | | | | | | | | |
| 545 | 212296\_at | PSMD14 | 1.760956054 | 9.128733476 | 7.50542963 | 2.98E-007 | 9.68E-006 | Proteasome (prosome, macropain) 26S subunit, non-ATPase, 14 | ubiquitin-dependent protein catabolism |  | | | | | | | | | | | | | | | | | | | | | | | | | | | |
| 546 | 203384\_s\_at | GOLGA1 | -0.691319092 | 6.271129245 | -7.49620621 | 3.03E-007 | 9.85E-006 | golgi autoantigen, golgin subfamily a, 1 | NA |  | | | | | | | | | | | | | | | | | | | | | | | | | | | |
| 547 | 202173\_s\_at | ZNF161 | -1.100406042 | 6.821723147 | -7.49482429 | 3.04E-007 | 9.86E-006 | zinc finger protein 161 | cellular defense response, regulation of transcription from Pol II promoter |  | | | | | | | | | | | | | | | | | | | | | | | | | | | |
| 548 | 221514\_at | UTP14A | 1.959863201 | 7.32160099 | 7.48848628 | 3.08E-007 | 9.95E-006 | UTP14, U3 small nucleolar ribonucleoprotein, homolog A (yeast) | NA |  | | | | | | | | | | | | | | | | | | | | | | | | | | | |
| 549 | 205395\_s\_at | MRE11A | 1.085839301 | 6.914000853 | 7.48823405 | 3.08E-007 | 9.95E-006 | MRE11 meiotic recombination 11 homolog A (S. cerevisiae) | double-strand break repair via nonhomologous end-joining, meiosis, meiotic recombination, regulation of mitotic recombination, telomerase-dependent telomere maintenance |  | | | | | | | | | | | | | | | | | | | | | | | | | | | |
| 550 | 208981\_at | PECAM1 | -2.981516636 | 8.129093654 | -7.4869496 | 3.09E-007 | 9.96E-006 | platelet/endothelial cell adhesion molecule (CD31 antigen) | cell motility, cell recognition, signal transduction |  | | | | | | | | | | | | | | | | | | | | | | | | | | | |
| 551 | 220688\_s\_at | C1orf33 | 1.738367526 | 8.14430132 | 7.48271656 | 3.12E-007 | 1.00E-005 | chromosome 1 open reading frame 33 | protein biosynthesis, ribosome biogenesis |  | | | | | | | | | | | | | | | | | | | | | | | | | | | |
| 552 | 208764\_s\_at | ATP5G2 | 1.364013373 | 10.75062059 | 7.47868272 | 3.14E-007 | 1.01E-005 | ATP synthase, H+ transporting, mitochondrial F0 complex, subunit c (subunit 9), isoform 2 | ATP synthesis coupled proton transport, proton transport |  | | | | | | | | | | | | | | | | | | | | | | | | | | | |
| 553 | 209804\_at | DCLRE1A | 0.69809398 | 6.913896511 | 7.47697029 | 3.15E-007 | 1.01E-005 | DNA cross-link repair 1A (PSO2 homolog, S. cerevisiae) | NA |  | | | | | | | | | | | | | | | | | | | | | | | | | | | |
| 554 | 220864\_s\_at | GRIM19 | 0.836498081 | 9.666776608 | 7.47632694 | 3.16E-007 | 1.01E-005 | cell death-regulatory protein GRIM19 | apoptosis, apoptotic nuclear changes, induction of apoptosis by extracellular signals, negative regulation of cell growth, negative regulation of protein biosynthesis, negative regulation of transcription, DNA-dependent, protein-nucleus import |  | | | | | | | | | | | | | | | | | | | | | | | | | | | |
| 555 | 201294\_s\_at | WSB1 | -1.601731587 | 7.834140687 | -7.47458016 | 3.17E-007 | 1.01E-005 | WD repeat and SOCS box-containing 1 | intracellular signaling cascade |  | | | | | | | | | | | | | | | | | | | | | | | | | | | |
| 556 | 212288\_at | FNBP1 | -1.63037911 | 9.469207181 | -7.47221583 | 3.18E-007 | 1.02E-005 | formin binding protein 1 | NA |  | | | | | | | | | | | | | | | | | | | | | | | | | | | |
| 557 | 218256\_s\_at | NUP54 | 1.928393199 | 7.261605412 | 7.47051177 | 3.19E-007 | 1.02E-005 | nucleoporin 54kDa | transport |  | | | | | | | | | | | | | | | | | | | | | | | | | | | |
| 558 | 203629\_s\_at | COG5 | 0.924152769 | 5.785555954 | 7.46828491 | 3.21E-007 | 1.02E-005 | component of oligomeric golgi complex 5 | intra-Golgi transport, protein transport |  | | | | | | | | | | | | | | | | | | | | | | | | | | | |
| 559 | 219452\_at | DPEP2 | -2.411773697 | 7.719881665 | -7.46183274 | 3.25E-007 | 1.03E-005 | dipeptidase 2 | proteolysis and peptidolysis |  | | | | | | | | | | | | | | | | | | | | | | | | | | | |
| 560 | 217720\_at | CHCHD2 | 1.257039167 | 11.62941378 | 7.45608385 | 3.29E-007 | 1.04E-005 | coiled-coil-helix-coiled-coil-helix domain containing 2 | NA |  | | | | | | | | | | | | | | | | | | | | | | | | | | | |
| 561 | 208799\_at | PSMB5 | 1.396280173 | 9.025790556 | 7.45418409 | 3.30E-007 | 1.04E-005 | proteasome (prosome, macropain) subunit, beta type, 5 | ubiquitin-dependent protein catabolism |  | | | | | | | | | | | | | | | | | | | | | | | | | | | |
| 562 | 209259\_s\_at | CSPG6 | 1.473998531 | 7.551251666 | 7.45395014 | 3.30E-007 | 1.04E-005 | chondroitin sulfate proteoglycan 6 (bamacan) | DNA repair, cell cycle, chromosome segregation, meiosis, mitotic spindle assembly, signal transduction, sister chromatid cohesion, transport |  | | | | | | | | | | | | | | | | | | | | | | | | | | | |
| 563 | 221586\_s\_at | E2F5 | 0.993872826 | 5.219859133 | 7.44567274 | 3.36E-007 | 1.06E-005 | E2F transcription factor 5, p130-binding | regulation of cell cycle, regulation of transcription, DNA-dependent |  | | | | | | | | | | | | | | | | | | | | | | | | | | | |
| 564 | 210620\_s\_at | GTF3C2 | 0.688018186 | 7.569977458 | 7.43808894 | 3.41E-007 | 1.07E-005 | general transcription factor IIIC, polypeptide 2, beta 110kDa | transcription |  | | | | | | | | | | | | | | | | | | | | | | | | | | | |
| 565 | 201509\_at | IDH3B | 0.747104892 | 8.287428509 | 7.42717898 | 3.48E-007 | 1.09E-005 | isocitrate dehydrogenase 3 (NAD+) beta | carbohydrate metabolism, isocitrate metabolism, metabolism, tricarboxylic acid cycle |  | | | | | | | | | | | | | | | | | | | | | | | | | | | |
| 566 | 210470\_x\_at | NONO | 1.34069833 | 10.55339178 | 7.42555389 | 3.50E-007 | 1.09E-005 | non-POU domain containing, octamer-binding | RNA splicing, mRNA processing |  | | | | | | | | | | | | | | | | | | | | | | | | | | | |
| 567 | 219293\_s\_at | PTD004 | 2.091538616 | 9.849471161 | 7.42236442 | 3.52E-007 | 1.10E-005 | hypothetical protein PTD004 | NA |  | | | | | | | | | | | | | | | | | | | | | | | | | | | |
| 568 | 208694\_at | PRKDC | 1.78119263 | 7.367272693 | 7.42217356 | 3.52E-007 | 1.10E-005 | protein kinase, DNA-activated, catalytic polypeptide | DNA recombination, double-strand break repair, protein modification |  | | | | | | | | | | | | | | | | | | | | | | | | | | | |
| 569 | 220034\_at | IRAK3 | -1.652139034 | 6.747940216 | -7.42051972 | 3.53E-007 | 1.10E-005 | interleukin-1 receptor-associated kinase 3 | cell surface receptor linked signal transduction, protein amino acid phosphorylation |  | | | | | | | | | | | | | | | | | | | | | | | | | | | |
| 570 | 208707\_at | EIF5 | -1.196853744 | 7.383990472 | -7.41941434 | 3.54E-007 | 1.10E-005 | eukaryotic translation initiation factor 5 | protein biosynthesis, regulation of translational initiation |  | | | | | | | | | | | | | | | | | | | | | | | | | | | |
| 571 | 205255\_x\_at | TCF7 | -0.927860388 | 8.364010421 | -7.41868174 | 3.54E-007 | 1.10E-005 | transcription factor 7 (T-cell specific, HMG-box) | Wnt receptor signaling pathway, immune response, regulation of transcription from Pol II promoter, regulation of transcription, DNA-dependent |  | | | | | | | | | | | | | | | | | | | | | | | | | | | |
| 572 | 207571\_x\_at | C1orf38 | -2.998426773 | 8.539456969 | -7.4175119 | 3.55E-007 | 1.10E-005 | chromosome 1 open reading frame 38 | cell adhesion |  | | | | | | | | | | | | | | | | | | | | | | | | | | | |
| 573 | 201502\_s\_at | NFKBIA | -2.546486371 | 10.42475312 | -7.40282357 | 3.66E-007 | 1.13E-005 | nuclear factor of kappa light polypeptide gene enhancer in B-cells inhibitor, alpha | apoptosis, cytoplasmic sequestering of NF-kappaB, response to pathogenic bacteria |  | | | | | | | | | | | | | | | | | | | | | | | | | | | |
| 574 | 218513\_at | FLJ11184 | 0.854624446 | 4.218264121 | 7.39814467 | 3.69E-007 | 1.14E-005 | hypothetical protein FLJ11184 | NA |  | | | | | | | | | | | | | | | | | | | | | | | | | | | |
| 575 | 212110\_at | SLC39A14 | 1.819380748 | 7.06575732 | 7.39705513 | 3.70E-007 | 1.14E-005 | solute carrier family 39 (zinc transporter), member 14 | metal ion transport |  | | | | | | | | | | | | | | | | | | | | | | | | | | | |
| 576 | 201209\_at | HDAC1 | 0.92834672 | 8.995358848 | 7.38792764 | 3.77E-007 | 1.16E-005 | histone deacetylase 1 | anti-apoptosis, chromatin modification, histone deacetylation, regulation of transcription, DNA-dependent |  | | | | | | | | | | | | | | | | | | | | | | | | | | | |
| 577 | 204232\_at | FCER1G | -3.578410015 | 9.257225922 | -7.38642705 | 3.78E-007 | 1.16E-005 | Fc fragment of IgE, high affinity I, receptor for; gamma polypeptide | cell surface receptor linked signal transduction, immune response |  | | | | | | | | | | | | | | | | | | | | | | | | | | | |
| 578 | 200017\_at | RPS27A | 0.899202785 | 11.94915777 | 7.37942702 | 3.84E-007 | 1.17E-005 | ribosomal protein S27a /// ribosomal protein S27a | protein biosynthesis |  | | | | | | | | | | | | | | | | | | | | | | | | | | | |
| 579 | 201555\_at | MCM3 | 1.525437966 | 9.369637619 | 7.37816909 | 3.85E-007 | 1.18E-005 | MCM3 minichromosome maintenance deficient 3 (S. cerevisiae) | DNA replication, DNA replication initiation, cell cycle, regulation of transcription, DNA-dependent |  | | | | | | | | | | | | | | | | | | | | | | | | | | | |
| 580 | 218894\_s\_at | FLJ10292 | 1.093456018 | 6.077574545 | 7.37659544 | 3.86E-007 | 1.18E-005 | mago-nashi homolog | sex determination |  | | | | | | | | | | | | | | | | | | | | | | | | | | | |
| 581 | 202959\_at | MUT | 0.795082203 | 6.082343996 | 7.37549008 | 3.87E-007 | 1.18E-005 | methylmalonyl Coenzyme A mutase | metabolism |  | | | | | | | | | | | | | | | | | | | | | | | | | | | |
| 582 | 200860\_s\_at | KIAA1007 | 0.712046329 | 9.989328274 | 7.37440866 | 3.88E-007 | 1.18E-005 | KIAA1007 protein | NA |  | | | | | | | | | | | | | | | | | | | | | | | | | | | |
| 583 | 213025\_at | THUMPD1 | 1.44527708 | 6.168376147 | 7.37421814 | 3.88E-007 | 1.18E-005 | THUMP domain containing 1 | NA |  | | | | | | | | | | | | | | | | | | | | | | | | | | | |
| 584 | 216863\_s\_at | ZCWCC1 | 0.970867604 | 7.302914675 | 7.37060472 | 3.91E-007 | 1.18E-005 | zinc finger, CW-type with coiled-coil domain 1 | NA |  | | | | | | | | | | | | | | | | | | | | | | | | | | | |
| 585 | 202852\_s\_at | FLJ11506 | 1.277032393 | 7.111631381 | 7.36568388 | 3.94E-007 | 1.19E-005 | hypothetical protein FLJ11506 | NA |  | | | | | | | | | | | | | | | | | | | | | | | | | | | |
| 586 | 205176\_s\_at | ITGB3BP | 1.856992758 | 7.649122179 | 7.3636302 | 3.96E-007 | 1.19E-005 | integrin beta 3 binding protein (beta3-endonexin) | apoptosis, cell adhesion, regulation of transcription, DNA-dependent, signal transduction |  | | | | | | | | | | | | | | | | | | | | | | | | | | | |
| 587 | 202615\_at | GNAQ | -1.352446352 | 9.889683046 | -7.36123851 | 3.98E-007 | 1.20E-005 | guanine nucleotide binding protein (G protein), q polypeptide | G-protein coupled receptor protein signaling pathway, blood coagulation, phospholipase C activation, protein amino acid ADP-ribosylation, signal transduction |  | | | | | | | | | | | | | | | | | | | | | | | | | | | |
| 588 | 209445\_x\_at | FLJ10803 | 1.109645993 | 8.12889122 | 7.35971482 | 3.99E-007 | 1.20E-005 | hypothetical protein FLJ10803 | NA |  | | | | | | | | | | | | | | | | | | | | | | | | | | | |
| 589 | 202039\_at | MYO18A /// TIAF1 | -0.820923883 | 7.957693816 | -7.35946421 | 3.99E-007 | 1.20E-005 | myosin XVIIIA /// TGFB1-induced anti-apoptotic factor 1 | DNA recombination, DNA repair, I-kappaB kinase/NF-kappaB cascade, anti-apoptosis, apoptosis |  | | | | | | | | | | | | | | | | | | | | | | | | | | | |
| 590 | 213850\_s\_at | SFRS2IP | -0.815638429 | 9.343605531 | -7.35826972 | 4.00E-007 | 1.20E-005 | splicing factor, arginine/serine-rich 2, interacting protein | RNA splicing, mRNA processing |  | | | | | | | | | | | | | | | | | | | | | | | | | | | |
| 591 | 202070\_s\_at | IDH3A | 1.087161984 | 7.32501605 | 7.35507743 | 4.03E-007 | 1.20E-005 | isocitrate dehydrogenase 3 (NAD+) alpha | carbohydrate metabolism, metabolism, tricarboxylic acid cycle |  | | | | | | | | | | | | | | | | | | | | | | | | | | | |
| 592 | 219240\_s\_at | C10orf88 | 0.669055793 | 5.791796363 | 7.35471876 | 4.03E-007 | 1.20E-005 | chromosome 10 open reading frame 88 | NA |  | | | | | | | | | | | | | | | | | | | | | | | | | | | |
| 593 | 201840\_at | NEDD8 | 0.581951688 | 9.566254601 | 7.35337266 | 4.04E-007 | 1.20E-005 | neural precursor cell expressed, developmentally down-regulated 8 | morphogenesis, proteolysis and peptidolysis, ubiquitin cycle, ubiquitin-dependent protein catabolism |  | | | | | | | | | | | | | | | | | | | | | | | | | | | |
| 594 | 210249\_s\_at | NCOA1 | -1.038405251 | 8.730823688 | -7.35212738 | 4.05E-007 | 1.21E-005 | nuclear receptor coactivator 1 | transcription |  | | | | | | | | | | | | | | | | | | | | | | | | | | | |
| 595 | 218395\_at | ACTR6 | 1.510236585 | 5.613072693 | 7.33870271 | 4.17E-007 | 1.24E-005 | ARP6 actin-related protein 6 homolog (yeast) | NA |  | | | | | | | | | | | | | | | | | | | | | | | | | | | |
| 596 | 200056\_s\_at | C1D | 1.052813655 | 7.210407551 | 7.33723892 | 4.18E-007 | 1.24E-005 | nuclear DNA-binding protein /// nuclear DNA-binding protein | NA |  | | | | | | | | | | | | | | | | | | | | | | | | | | | |
| 597 | 208726\_s\_at | EIF2S2 | 1.788545434 | 10.00734283 | 7.33345444 | 4.21E-007 | 1.25E-005 | eukaryotic translation initiation factor 2, subunit 2 beta, 38kDa | protein biosynthesis, translational initiation |  | | | | | | | | | | | | | | | | | | | | | | | | | | | |
| 598 | 204334\_at | KLF7 | -1.862319232 | 8.374563676 | -7.32982864 | 4.24E-007 | 1.25E-005 | Kruppel-like factor 7 (ubiquitous) | regulation of transcription from Pol II promoter |  | | | | | | | | | | | | | | | | | | | | | | | | | | | |
| 599 | 202184\_s\_at | NUP133 | 1.610554403 | 8.050875517 | 7.32710479 | 4.27E-007 | 1.26E-005 | nucleoporin 133kDa | RNA transport, mRNA-nucleus export, protein transport |  | | | | | | | | | | | | | | | | | | | | | | | | | | | |
| 600 | 214575\_s\_at | AZU1 | -4.220875595 | 10.54482698 | -7.32363869 | 4.30E-007 | 1.27E-005 | azurocidin 1 (cationic antimicrobial protein 37) | cellular extravasation, chemotaxis, defense response to Gram-negative bacteria, glia cell migration, induction of positive chemotaxis, inhibition of caspase activation, macrophage chemotaxis, microglial cell activation, monocyte activation, positive regulation of MHC class II biosynthesis, positive regulation of cell adhesion, positive regulation of fractalkine biosynthesis, positive regulation of interleukin-1 beta biosynthesis, positive regulation of phagocytosis, positive regulation of tumor necrosis factor-alpha biosynthesis, protein kinase C activation, proteolysis and peptidolysis, regulation of vascular permeability | | | | | | | | | | | | | | | | | | | | | | |  | | | | | |
| 601 | 211433\_x\_at | KIAA1539 | -0.977850538 | 8.247003455 | -7.32301557 | 4.30E-007 | 1.27E-005 | KIAA1539 | NA |  | | | | | | | | | | | | | | | | | | | | | | | | | | | |
| 602 | 207515\_s\_at | POLR1C | 0.97926554 | 7.561939785 | 7.32080541 | 4.32E-007 | 1.27E-005 | polymerase (RNA) I polypeptide C, 30kDa | transcription, transcription from Pol I promoter |  | | | | | | | | | | | | | | | | | | | | | | | | | | | |
| 603 | 201492\_s\_at | RPL41 | 0.338065244 | 13.81632513 | 7.3120781 | 4.40E-007 | 1.29E-005 | ribosomal protein L41 | NA |  | | | | | | | | | | | | | | | | | | | | | | | | | | | |
| 604 | 200091\_s\_at | RPS25 | 1.118023462 | 11.37030709 | 7.30576894 | 4.45E-007 | 1.30E-005 | ribosomal protein S25 /// ribosomal protein S25 | NA |  | | | | | | | | | | | | | | | | | | | | | | | | | | | |
| 605 | 209219\_at | RDBP | 0.957211246 | 9.060335343 | 7.30543274 | 4.46E-007 | 1.30E-005 | RD RNA binding protein | regulation of transcription, DNA-dependent |  | | | | | | | | | | | | | | | | | | | | | | | | | | | |
| 606 | 212534\_at | ZNF24 | 1.273771971 | 8.130982791 | 7.30511854 | 4.46E-007 | 1.30E-005 | Zinc finger protein 24 (KOX 17) | negative regulation of transcription, regulation of transcription, DNA-dependent |  | | | | | | | | | | | | | | | | | | | | | | | | | | | |
| 607 | 202453\_s\_at | GTF2H1 | 1.032611617 | 7.35346143 | 7.3025851 | 4.48E-007 | 1.30E-005 | general transcription factor IIH, polypeptide 1, 62kDa | DNA repair, regulation of cyclin dependent protein kinase activity, regulation of transcription, DNA-dependent, transcription from Pol II promoter |  | | | | | | | | | | | | | | | | | | | | | | | | | | | |
| 608 | 200022\_at | RPL18 | 1.367103163 | 12.31536917 | 7.30200769 | 4.49E-007 | 1.30E-005 | ribosomal protein L18 /// ribosomal protein L18 | protein biosynthesis |  | | | | | | | | | | | | | | | | | | | | | | | | | | | |
| 609 | 218315\_s\_at | CDK5RAP1 | 0.695632639 | 8.046101922 | 7.29691379 | 4.54E-007 | 1.32E-005 | CDK5 regulatory subunit associated protein 1 | brain development, cell proliferation, negative regulation of cyclin dependent protein kinase activity, regulation of neuron differentiation |  | | | | | | | | | | | | | | | | | | | | | | | | | | | |
| 610 | 212653\_s\_at | EHBP1 | 1.39964904 | 6.72924166 | 7.29239033 | 4.58E-007 | 1.32E-005 | EH domain binding protein 1 | ubiquitin cycle |  | | | | | | | | | | | | | | | | | | | | | | | | | | | |
| 611 | 211698\_at | CRI1 | 0.930662643 | 5.143298107 | 7.29217978 | 4.58E-007 | 1.32E-005 | CREBBP/EP300 inhibitor 1 /// CREBBP/EP300 inhibitor 1 | NA |  | | | | | | | | | | | | | | | | | | | | | | | | | | | |
| 612 | 217774\_s\_at | HSPC152 | 1.003456005 | 9.917083321 | 7.28751562 | 4.62E-007 | 1.33E-005 | hypothetical protein HSPC152 | NA |  | | | | | | | | | | | | | | | | | | | | | | | | | | | |
| 613 | 201577\_at | NME1 | 2.441212141 | 9.641851995 | 7.28429025 | 4.65E-007 | 1.34E-005 | non-metastatic cells 1, protein (NM23A) expressed in | CTP biosynthesis, GTP biosynthesis, UTP biosynthesis, negative regulation of cell cycle, negative regulation of cell proliferation, nucleoside triphosphate biosynthesis |  | | | | | | | | | | | | | | | | | | | | | | | | | | | |
| 614 | 218414\_s\_at | NDE1 | -1.086057383 | 8.542432201 | -7.28371149 | 4.66E-007 | 1.34E-005 | nudE nuclear distribution gene E homolog 1 (A. nidulans) | NA |  | | | | | | | | | | | | | | | | | | | | | | | | | | | |
| 615 | 204563\_at | SELL | -4.283732493 | 8.829396186 | -7.28034927 | 4.69E-007 | 1.34E-005 | selectin L (lymphocyte adhesion molecule 1) | cell adhesion, cell motility |  | | | | | | | | | | | | | | | | | | | | | | | | | | | |
| 616 | 209002\_s\_at | KIAA1536 | -0.909679817 | 8.241484789 | -7.27576622 | 4.73E-007 | 1.35E-005 | KIAA1536 protein | NA |  | | | | | | | | | | | | | | | | | | | | | | | | | | | |
| 617 | 201482\_at | QSCN6 | -1.192638517 | 9.198420292 | -7.27508426 | 4.74E-007 | 1.35E-005 | quiescin Q6 | cell growth and/or maintenance, electron transport, negative regulation of cell proliferation, regulation of cell cycle, regulation of cell growth |  | | | | | | | | | | | | | | | | | | | | | | | | | | | |
| 618 | 202666\_s\_at | ACTL6A | 1.420138557 | 5.538178244 | 7.27251319 | 4.77E-007 | 1.36E-005 | actin-like 6A | chromatin remodeling, response to pest, pathogen or parasite, signal transduction |  | | | | | | | | | | | | | | | | | | | | | | | | | | | |
| 619 | 221652\_s\_at | FLJ10637 | 2.602292752 | 7.792909257 | 7.26970013 | 4.79E-007 | 1.37E-005 | hypothetical protein FLJ10637 | NA |  | | | | | | | | | | | | | | | | | | | | | | | | | | | |
| 620 | 221080\_s\_at | FAM31C | -1.086932901 | 8.033146864 | -7.26899274 | 4.80E-007 | 1.37E-005 | family with sequence similarity 31, member C | NA |  | | | | | | | | | | | | | | | | | | | | | | | | | | | |
| 621 | 204220\_at | GMFG | -1.978323076 | 9.985995197 | -7.26818041 | 4.81E-007 | 1.37E-005 | glia maturation factor, gamma | protein amino acid phosphorylation |  | | | | | | | | | | | | | | | | | | | | | | | | | | | |
| 622 | 207830\_s\_at | PPP1R8 | 1.050062794 | 8.740780204 | 7.26618043 | 4.83E-007 | 1.37E-005 | protein phosphatase 1, regulatory (inhibitor) subunit 8 | RNA catabolism, nuclear mRNA splicing, via spliceosome |  | | | | | | | | | | | | | | | | | | | | | | | | | | | |
| 623 | 214263\_x\_at | POLR2C | 0.517760442 | 8.031515728 | 7.25285647 | 4.96E-007 | 1.40E-005 | polymerase (RNA) II (DNA directed) polypeptide C, 33kDa | transcription, transcription from Pol II promoter |  | | | | | | | | | | | | | | | | | | | | | | | | | | | |
| 624 | 217185\_s\_at | ZNF259 | 1.363103459 | 7.434505501 | 7.25012481 | 4.99E-007 | 1.41E-005 | zinc finger protein 259 | cell proliferation, signal transduction |  | | | | | | | | | | | | | | | | | | | | | | | | | | | |
| 625 | 203752\_s\_at | JUND | -1.081027419 | 11.1461529 | -7.249453 | 5.00E-007 | 1.41E-005 | jun D proto-oncogene | regulation of transcription from Pol II promoter |  | | | | | | | | | | | | | | | | | | | | | | | | | | | |
| 626 | 202576\_s\_at | DDX19 /// FLJ11126 | 0.789631032 | 7.917900633 | 7.24668075 | 5.02E-007 | 1.41E-005 | DEAD (Asp-Glu-Ala-As) box polypeptide 19 /// FLJ11126 protein | mRNA-nucleus export |  | | | | | | | | | | | | | | | | | | | | | | | | | | | |
| 627 | 209017\_s\_at | PRSS15 | 2.000205972 | 8.488221277 | 7.23791917 | 5.11E-007 | 1.44E-005 | protease, serine, 15 | ATP-dependent proteolysis |  | | | | | | | | | | | | | | | | | | | | | | | | | | | |
| 628 | 218001\_at | MRPS2 | 2.246815537 | 7.545239848 | 7.23496779 | 5.15E-007 | 1.44E-005 | mitochondrial ribosomal protein S2 | protein biosynthesis |  | | | | | | | | | | | | | | | | | | | | | | | | | | | |
| 629 | 219220\_x\_at | MRPS22 | 0.952042625 | 8.452674177 | 7.22424056 | 5.26E-007 | 1.47E-005 | mitochondrial ribosomal protein S22 | NA |  | | | | | | | | | | | | | | | | | | | | | | | | | | | |
| 630 | 217935\_s\_at | C20orf44 | 0.849625279 | 7.592555548 | 7.2197835 | 5.31E-007 | 1.48E-005 | chromosome 20 open reading frame 44 | NA |  | | | | | | | | | | | | | | | | | | | | | | | | | | | |
| 631 | 205252\_at | ZNF174 | 0.525749637 | 6.241779772 | 7.21696987 | 5.34E-007 | 1.48E-005 | zinc finger protein 174 | negative regulation of transcription from Pol II promoter, regulation of transcription, DNA-dependent |  | | | | | | | | | | | | | | | | | | | | | | | | | | | |
| 632 | 201152\_s\_at | MBNL1 | -1.011924779 | 10.36394013 | -7.21619731 | 5.35E-007 | 1.48E-005 | muscleblind-like (Drosophila) | embryonic development (sensu Mammalia), embryonic limb morphogenesis, muscle development, myoblast differentiation, neurogenesis |  | | | | | | | | | | | | | | | | | | | | | | | | | | | |
| 633 | 218408\_at | TIMM10 | 1.059498895 | 8.028552328 | 7.21398125 | 5.37E-007 | 1.49E-005 | translocase of inner mitochondrial membrane 10 homolog (yeast) | mitochondrial inner membrane protein import, protein transport |  | | | | | | | | | | | | | | | | | | | | | | | | | | | |
| 634 | 208838\_at | TIP120A | 1.209005312 | 6.635134277 | 7.21320651 | 5.38E-007 | 1.49E-005 | TBP-interacting protein | cell differentiation, ubiquitin cycle |  | | | | | | | | | | | | | | | | | | | | | | | | | | | |
| 635 | 201871\_s\_at | LOC51035 | 1.042199605 | 9.075442003 | 7.21190318 | 5.39E-007 | 1.49E-005 | ORF | NA |  | | | | | | | | | | | | | | | | | | | | | | | | | | | |
| 636 | 208758\_at | ATIC | 2.541638542 | 9.05390097 | 7.21177523 | 5.40E-007 | 1.49E-005 | 5-aminoimidazole-4-carboxamide ribonucleotide formyltransferase/IMP cyclohydrolase | nucleobase, nucleoside, nucleotide and nucleic acid metabolism, purine nucleotide biosynthesis |  | | | | | | | | | | | | | | | | | | | | | | | | | | | |
| 637 | 219889\_at | FRAT1 | -1.850189866 | 7.921849195 | -7.21104991 | 5.40E-007 | 1.49E-005 | frequently rearranged in advanced T-cell lymphomas | Wnt receptor signaling pathway, cell growth and/or maintenance |  | | | | | | | | | | | | | | | | | | | | | | | | | | | |
| 638 | 205167\_s\_at | CDC25C | 1.016070771 | 5.684539386 | 7.21071251 | 5.41E-007 | 1.49E-005 | cell division cycle 25C | cytokinesis, protein amino acid dephosphorylation, regulation of cyclin dependent protein kinase activity, regulation of mitosis, traversing start control point of mitotic cell cycle |  | | | | | | | | | | | | | | | | | | | | | | | | | | | |
| 639 | 215136\_s\_at | EXOSC8 | 2.111444215 | 7.357002243 | 7.2090651 | 5.43E-007 | 1.49E-005 | exosome component 8 | rRNA processing |  | | | | | | | | | | | | | | | | | | | | | | | | | | | |
| 640 | 203654\_s\_at | COIL | 1.053104425 | 7.27447235 | 7.20586754 | 5.46E-007 | 1.50E-005 | coilin | NA |  | | | | | | | | | | | | | | | | | | | | | | | | | | | |
| 641 | 202550\_s\_at | VAPB | 0.780824488 | 7.930254235 | 7.20230039 | 5.50E-007 | 1.51E-005 | VAMP (vesicle-associated membrane protein)-associated protein B and C | protein complex assembly |  | | | | | | | | | | | | | | | | | | | | | | | | | | | |
| 642 | 200698\_at | KDELR2 | 1.041154008 | 7.145853421 | 7.20058189 | 5.52E-007 | 1.51E-005 | KDEL (Lys-Asp-Glu-Leu) endoplasmic reticulum protein retention receptor 2 | intracellular protein transport |  | | | | | | | | | | | | | | | | | | | | | | | | | | | |
| 643 | 200826\_at | SNRPD2 | 1.497617358 | 10.4048547 | 7.19498923 | 5.58E-007 | 1.52E-005 | small nuclear ribonucleoprotein D2 polypeptide 16.5kDa | nuclear mRNA splicing, via spliceosome |  | | | | | | | | | | | | | | | | | | | | | | | | | | | |
| 644 | 213119\_at | SLC36A1 | -0.854507202 | 7.731254208 | -7.19327037 | 5.60E-007 | 1.52E-005 | solute carrier family 36 (proton/amino acid symporter), member 1 | amino acid transport, transport |  | | | | | | | | | | | | | | | | | | | | | | | | | | | |
| 645 | 51192\_at | SSH3 | -0.814657009 | 7.899558771 | -7.18637214 | 5.68E-007 | 1.54E-005 | slingshot homolog 3 (Drosophila) | protein amino acid dephosphorylation |  | | | | | | | | | | | | | | | | | | | | | | | | | | | |
| 646 | 201009\_s\_at | TXNIP | -2.715137373 | 11.26879727 | -7.1849377 | 5.70E-007 | 1.55E-005 | thioredoxin interacting protein | NA |  | | | | | | | | | | | | | | | | | | | | | | | | | | | |
| 647 | 210820\_x\_at | COQ7 | 0.586367133 | 6.594730942 | 7.18277956 | 5.73E-007 | 1.55E-005 | coenzyme Q7 homolog, ubiquinone (yeast) | gluconeogenesis, ubiquinone biosynthesis |  | | | | | | | | | | | | | | | | | | | | | | | | | | | |
| 648 | 200619\_at | SF3B2 | 0.64341359 | 9.524457023 | 7.17611191 | 5.80E-007 | 1.57E-005 | Splicing factor 3b, subunit 2, 145kDa | RNA splicing, mRNA processing, nuclear mRNA splicing, via spliceosome |  | | | | | | | | | | | | | | | | | | | | | | | | | | | |
| 649 | 204781\_s\_at | TNFRSF6 | -1.244151941 | 7.254172455 | -7.17525928 | 5.81E-007 | 1.57E-005 | tumor necrosis factor receptor superfamily, member 6 | anti-apoptosis, apoptosis, immune response, induction of apoptosis, protein complex assembly, regulation of apoptosis, signal transduction |  | | | | | | | | | | | | | | | | | | | | | | | | | | | |
| 650 | 203572\_s\_at | TAF6 | 0.658633908 | 7.964428288 | 7.17312063 | 5.84E-007 | 1.58E-005 | TAF6 RNA polymerase II, TATA box binding protein (TBP)-associated factor, 80kDa | regulation of transcription, DNA-dependent, transcription initiation |  | | | | | | | | | | | | | | | | | | | | | | | | | | | |
| 651 | 216295\_s\_at | CLTA | 1.161050698 | 10.08414138 | 7.17284214 | 5.84E-007 | 1.58E-005 | clathrin, light polypeptide (Lca) | intracellular protein transport |  | | | | | | | | | | | | | | | | | | | | | | | | | | | |
| 652 | 212673\_at | METAP1 | 1.017886787 | 8.138484957 | 7.17042387 | 5.87E-007 | 1.58E-005 | methionyl aminopeptidase 1 | proteolysis and peptidolysis |  | | | | | | | | | | | | | | | | | | | | | | | | | | | |
| 653 | 210644\_s\_at | LAIR1 | -2.056554812 | 9.591537177 | -7.16423881 | 5.95E-007 | 1.60E-005 | leukocyte-associated Ig-like receptor 1 | NA |  | | | | | | | | | | | | | | | | | | | | | | | | | | | |
| 654 | 220239\_at | KLHL7 | 1.43311314 | 6.390309775 | 7.15833682 | 6.02E-007 | 1.62E-005 | kelch-like 7 (Drosophila) | NA |  | | | | | | | | | | | | | | | | | | | | | | | | | | | |
| 655 | 200743\_s\_at | CLN2 | -1.319105086 | 10.35381825 | -7.15280306 | 6.09E-007 | 1.63E-005 | ceroid-lipofuscinosis, neuronal 2, late infantile (Jansky-Bielschowsky disease) | lipid metabolism, neurogenesis, proteolysis and peptidolysis |  | | | | | | | | | | | | | | | | | | | | | | | | | | | |
| 656 | 201273\_s\_at | SRP9 | 1.015912254 | 9.883402029 | 7.15250427 | 6.09E-007 | 1.63E-005 | signal recognition particle 9kDa | negative regulation of translational elongation, protein targeting |  | | | | | | | | | | | | | | | | | | | | | | | | | | | |
| 657 | 203341\_at | CEBPZ | 1.454188115 | 6.859622318 | 7.15065134 | 6.12E-007 | 1.63E-005 | CCAAT/enhancer binding protein zeta | regulation of transcription, DNA-dependent, transcription from Pol II promoter |  | | | | | | | | | | | | | | | | | | | | | | | | | | | |
| 658 | 220255\_at | FANCE | 1.198828715 | 7.16220748 | 7.14986952 | 6.13E-007 | 1.63E-005 | Fanconi anemia, complementation group E | DNA repair |  | | | | | | | | | | | | | | | | | | | | | | | | | | | |
| 659 | 220642\_x\_at | GPR89 | 1.086696626 | 7.435751153 | 7.14960219 | 6.13E-007 | 1.63E-005 | G protein-coupled receptor 89 | positive regulation of I-kappaB kinase/NF-kappaB cascade |  | | | | | | | | | | | | | | | | | | | | | | | | | | | |
| 660 | 205135\_s\_at | NUFIP1 | 1.481737618 | 5.866702717 | 7.14801193 | 6.15E-007 | 1.63E-005 | nuclear fragile X mental retardation protein interacting protein 1 | RNA processing |  | | | | | | | | | | | | | | | | | | | | | | | | | | | |
| 661 | 40148\_at | APBB2 | 0.480816499 | 4.418531596 | 7.14641321 | 6.17E-007 | 1.63E-005 | amyloid beta (A4) precursor protein-binding, family B, member 2 (Fe65-like) | actin filament-based movement, axonogenesis, cell cycle arrest, intracellular signaling cascade, negative regulation of S phase of mitotic cell cycle, negative regulation of cell growth, protein stabilization, regulation of transcription |  | | | | | | | | | | | | | | | | | | | | | | | | | | | |
| 662 | 209421\_at | MSH2 | 2.121570058 | 7.089387387 | 7.14070083 | 6.24E-007 | 1.65E-005 | mutS homolog 2, colon cancer, nonpolyposis type 1 (E. coli) | mismatch repair, negative regulation of cell cycle, postreplication repair |  | | | | | | | | | | | | | | | | | | | | | | | | | | | |
| 663 | 208079\_s\_at | STK6 | 2.373657072 | 7.38550593 | 7.13928623 | 6.26E-007 | 1.65E-005 | serine/threonine kinase 6 | cell cycle, mitosis, protein amino acid phosphorylation |  | | | | | | | | | | | | | | | | | | | | | | | | | | | |
| 664 | 220773\_s\_at | GPHN | 1.187792008 | 6.641215217 | 7.13840374 | 6.27E-007 | 1.65E-005 | gephyrin | Mo-molybdopterin cofactor biosynthesis |  | | | | | | | | | | | | | | | | | | | | | | | | | | | |
| 665 | 202212\_at | PES1 | 1.112342138 | 8.247258006 | 7.13779324 | 6.28E-007 | 1.65E-005 | pescadillo homolog 1, containing BRCT domain (zebrafish) | morphogenesis |  | | | | | | | | | | | | | | | | | | | | | | | | | | | |
| 666 | 215275\_at | T3JAM | -1.219406265 | 7.137150532 | -7.13778348 | 6.28E-007 | 1.65E-005 | TRAF3-interacting Jun N-terminal kinase (JNK)-activating modulator | NA |  | | | | | | | | | | | | | | | | | | | | | | | | | | | |
| 667 | 208645\_s\_at | RPS14 | 0.475243423 | 13.07308567 | 7.12949152 | 6.39E-007 | 1.68E-005 | ribosomal protein S14 | protein biosynthesis |  | | | | | | | | | | | | | | | | | | | | | | | | | | | |
| 668 | 210502\_s\_at | PPIE | 1.30363146 | 8.251119047 | 7.1288411 | 6.40E-007 | 1.68E-005 | peptidylprolyl isomerase E (cyclophilin E) | protein folding |  | | | | | | | | | | | | | | | | | | | | | | | | | | | |
| 669 | 203062\_s\_at | MDC1 | 1.307263601 | 7.929637142 | 7.12212434 | 6.48E-007 | 1.70E-005 | Mediator of DNA damage checkpoint 1 | NA |  | | | | | | | | | | | | | | | | | | | | | | | | | | | |
| 670 | 201775\_s\_at | KIAA0494 | -0.676224863 | 8.054297468 | -7.12122915 | 6.50E-007 | 1.70E-005 | KIAA0494 gene product | NA |  | | | | | | | | | | | | | | | | | | | | | | | | | | | |
| 671 | 221568\_s\_at | LIN7C | 0.884738134 | 5.901528295 | 7.12119457 | 6.50E-007 | 1.70E-005 | lin-7 homolog C (C. elegans) | NA |  | | | | | | | | | | | | | | | | | | | | | | | | | | | |
| 672 | 203694\_s\_at | DHX16 | 0.800993922 | 8.198186349 | 7.11906288 | 6.53E-007 | 1.70E-005 | DEAH (Asp-Glu-Ala-His) box polypeptide 16 | RNA splicing, nuclear mRNA splicing, via spliceosome, regulation of cell cycle |  | | | | | | | | | | | | | | | | | | | | | | | | | | | |
| 673 | 204173\_at | MLC1SA | 2.189599507 | 8.112933675 | 7.11502356 | 6.58E-007 | 1.71E-005 | myosin light chain 1 slow a | muscle development |  | | | | | | | | | | | | | | | | | | | | | | | | | | | |
| 674 | 213527\_s\_at | LOC146542 | -0.81632143 | 6.82612131 | -7.11168447 | 6.63E-007 | 1.72E-005 | similar to hypothetical protein MGC13138 | regulation of transcription, DNA-dependent |  | | | | | | | | | | | | | | | | | | | | | | | | | | | |
| 675 | 218719\_s\_at | FLJ13912 | 0.761659104 | 6.657186644 | 7.10636795 | 6.70E-007 | 1.74E-005 | hypothetical protein FLJ13912 | NA |  | | | | | | | | | | | | | | | | | | | | | | | | | | | |
| 676 | 212139\_at | GCN1L1 | 1.049201112 | 7.491457434 | 7.09290134 | 6.89E-007 | 1.79E-005 | GCN1 general control of amino-acid synthesis 1-like 1 (yeast) | regulation of translation |  | | | | | | | | | | | | | | | | | | | | | | | | | | | |
| 677 | 203189\_s\_at | NDUFS8 | 1.802759131 | 7.482915614 | 7.08937434 | 6.94E-007 | 1.80E-005 | NADH dehydrogenase (ubiquinone) Fe-S protein 8, 23kDa (NADH-coenzyme Q reductase) | electron transport, mitochondrial electron transport, NADH to ubiquinone |  | | | | | | | | | | | | | | | | | | | | | | | | | | | |
| 678 | 212332\_at | RBL2 | -1.287369198 | 8.368881467 | -7.08806312 | 6.96E-007 | 1.80E-005 | retinoblastoma-like 2 (p130) | cell cycle, negative regulation of cell cycle, regulation of transcription, DNA-dependent |  | | | | | | | | | | | | | | | | | | | | | | | | | | | |
| 679 | 219994\_at | APBB1IP | -1.796610211 | 7.663151483 | -7.08508809 | 7.00E-007 | 1.81E-005 | amyloid beta (A4) precursor protein-binding, family B, member 1 interacting protein | signal transduction |  | | | | | | | | | | | | | | | | | | | | | | | | | | | |
| 680 | 220587\_s\_at | GBL | 1.654560793 | 7.829753101 | 7.08378984 | 7.02E-007 | 1.81E-005 | G protein beta subunit-like | NA |  | | | | | | | | | | | | | | | | | | | | | | | | | | | |
| 681 | 221903\_s\_at | CYLD | -1.999393875 | 8.177447809 | -7.0705851 | 7.21E-007 | 1.86E-005 | cylindromatosis (turban tumor syndrome) | negative regulation of cell cycle, ubiquitin cycle, ubiquitin-dependent protein catabolism |  | | | | | | | | | | | | | | | | | | | | | | | | | | | |
| 682 | 209964\_s\_at | ATXN7 | -1.367796697 | 7.074614274 | -7.06942819 | 7.23E-007 | 1.86E-005 | ataxin 7 | nuclear organization and biogenesis, visual perception |  | | | | | | | | | | | | | | | | | | | | | | | | | | | |
| 683 | 202279\_at | C14orf2 | 1.39713023 | 8.875303999 | 7.06884262 | 7.24E-007 | 1.86E-005 | chromosome 14 open reading frame 2 | NA |  | | | | | | | | | | | | | | | | | | | | | | | | | | | |
| 684 | 211951\_at | NOLC1 | 1.778652388 | 9.254575926 | 7.06750973 | 7.26E-007 | 1.86E-005 | nucleolar and coiled-body phosphoprotein 1 | cell cycle, mitosis, rRNA processing |  | | | | | | | | | | | | | | | | | | | | | | | | | | | |
| 685 | 218645\_at | ZNF277 | 2.061108759 | 7.371174312 | 7.06733949 | 7.26E-007 | 1.86E-005 | zinc finger protein (C2H2 type) 277 | regulation of transcription, DNA-dependent |  | | | | | | | | | | | | | | | | | | | | | | | | | | | |
| 686 | 206707\_x\_at | C6orf32 | -3.318594618 | 8.568415609 | -7.06569612 | 7.28E-007 | 1.87E-005 | chromosome 6 open reading frame 32 | NA |  | | | | | | | | | | | | | | | | | | | | | | | | | | | |
| 687 | 221263\_s\_at | SF3B5 | 1.105829929 | 9.869525249 | 7.0641425 | 7.31E-007 | 1.87E-005 | splicing factor 3b, subunit 5, 10kDa /// splicing factor 3b, subunit 5, 10kDa | nuclear mRNA splicing, via spliceosome |  | | | | | | | | | | | | | | | | | | | | | | | | | | | |
| 688 | 210371\_s\_at | RBBP4 | 1.716614161 | 8.700532061 | 7.06058326 | 7.36E-007 | 1.88E-005 | retinoblastoma binding protein 4 | DNA repair, DNA replication, cell cycle, negative regulation of cell proliferation, regulation of transcription, DNA-dependent |  | | | | | | | | | | | | | | | | | | | | | | | | | | | |
| 689 | 221510\_s\_at | GLS | 1.248769347 | 6.942309962 | 7.06002821 | 7.37E-007 | 1.88E-005 | glutaminase | glutamine catabolism |  | | | | | | | | | | | | | | | | | | | | | | | | | | | |
| 690 | 202377\_at | OBRGRP /// LEPR | -1.639464451 | 8.496948296 | -7.05998025 | 7.37E-007 | 1.88E-005 | leptin receptor gene-related protein /// leptin receptor | cell surface receptor linked signal transduction, development, energy reserve metabolism |  | | | | | | | | | | | | | | | | | | | | | | | | | | | |
| 691 | 218061\_at | MEA | 0.695440107 | 9.287583252 | 7.05922502 | 7.38E-007 | 1.88E-005 | male-enhanced antigen | development, male gonad development, spermatogenesis |  | | | | | | | | | | | | | | | | | | | | | | | | | | | |
| 692 | 217782\_s\_at | GPS1 | 0.929456033 | 7.873570962 | 7.05872117 | 7.39E-007 | 1.88E-005 | G protein pathway suppressor 1 | JNK cascade, cell cycle, inactivation of MAPK |  | | | | | | | | | | | | | | | | | | | | | | | | | | | |
| 693 | 209945\_s\_at | GSK3B | -0.914001492 | 7.568758019 | -7.05791195 | 7.40E-007 | 1.88E-005 | glycogen synthase kinase 3 beta | Wnt receptor signaling pathway, glycogen metabolism, protein amino acid phosphorylation |  | | | | | | | | | | | | | | | | | | | | | | | | | | | |
| 694 | 203575\_at | CSNK2A2 | 2.149340458 | 7.49936084 | 7.0568215 | 7.42E-007 | 1.88E-005 | casein kinase 2, alpha prime polypeptide | Wnt receptor signaling pathway, protein amino acid phosphorylation, signal transduction, spermatid development |  | | | | | | | | | | | | | | | | | | | | | | | | | | | |
| 695 | 217980\_s\_at | MRPL16 | 1.198162603 | 8.791236985 | 7.05110109 | 7.51E-007 | 1.90E-005 | mitochondrial ribosomal protein L16 | protein biosynthesis |  | | | | | | | | | | | | | | | | | | | | | | | | | | | |
| 696 | 217841\_s\_at | PME-1 | 0.936050273 | 7.725764424 | 7.04974045 | 7.53E-007 | 1.91E-005 | protein phosphatase methylesterase-1 | protein amino acid demethylation |  | | | | | | | | | | | | | | | | | | | | | | | | | | | |
| 697 | 218646\_at | FLJ20534 | 0.799526371 | 8.088994859 | 7.04812911 | 7.55E-007 | 1.91E-005 | hypothetical protein FLJ20534 | NA |  | | | | | | | | | | | | | | | | | | | | | | | | | | | |
| 698 | 209337\_at | PSIP1 | 1.4661553 | 8.151137267 | 7.04736627 | 7.57E-007 | 1.91E-005 | PC4 and SFRS1 interacting protein 1 | NA |  | | | | | | | | | | | | | | | | | | | | | | | | | | | |
| 699 | 218069\_at | XTP3TPA | 0.932647154 | 9.52284477 | 7.04458742 | 7.61E-007 | 1.92E-005 | XTP3-transactivated protein A | NA |  | | | | | | | | | | | | | | | | | | | | | | | | | | | |
| 700 | 204331\_s\_at | MRPS12 | 1.514579574 | 7.916367209 | 7.04372351 | 7.62E-007 | 1.92E-005 | mitochondrial ribosomal protein S12 | protein biosynthesis |  | | | | | | | | | | | | | | | | | | | | | | | | | | | |
| 701 | 213951\_s\_at | HUMGT198A | 0.916661642 | 5.381072064 | 7.04242499 | 7.64E-007 | 1.92E-005 | GT198, complete ORF | NA |  | | | | | | | | | | | | | | | | | | | | | | | | | | | |
| 702 | 221860\_at | HNRPL | -2.14178773 | 7.998741022 | -7.04215841 | 7.65E-007 | 1.92E-005 | Heterogeneous nuclear ribonucleoprotein L | mRNA processing |  | | | | | | | | | | | | | | | | | | | | | | | | | | | |
| 703 | 218866\_s\_at | POLR3K | 1.106189122 | 7.72141573 | 7.04109858 | 7.66E-007 | 1.92E-005 | polymerase (RNA) III (DNA directed) polypeptide K, 12.3 kDa | RNA elongation, regulation of transcription, DNA-dependent, transcription, transcription from Pol III promoter |  | | | | | | | | | | | | | | | | | | | | | | | | | | | |
| 704 | 218564\_at | FLJ10520 | 1.336812584 | 5.85379245 | 7.03939531 | 7.69E-007 | 1.93E-005 | hypothetical protein FLJ10520 | NA |  | | | | | | | | | | | | | | | | | | | | | | | | | | | |
| 705 | 208753\_s\_at | NAP1L1 | 2.085868792 | 7.937086904 | 7.03871523 | 7.70E-007 | 1.93E-005 | nucleosome assembly protein 1-like 1 | DNA replication, nucleosome assembly, positive regulation of cell proliferation |  | | | | | | | | | | | | | | | | | | | | | | | | | | | |
| 706 | 201013\_s\_at | PAICS | 2.994226888 | 8.77904299 | 7.03122806 | 7.82E-007 | 1.96E-005 | phosphoribosylaminoimidazole carboxylase, phosphoribosylaminoimidazole succinocarboxamide synthetase | de novo' IMP biosynthesis, purine base biosynthesis, purine nucleotide biosynthesis |  | | | | | | | | | | | | | | | | | | | | | | | | | | | |
| 707 | 203339\_at | SLC25A12 | 0.655265755 | 6.323094113 | 7.03105097 | 7.82E-007 | 1.96E-005 | solute carrier family 25 (mitochondrial carrier, Aralar), member 12 | transport |  | | | | | | | | | | | | | | | | | | | | | | | | | | | |
| 708 | 205519\_at | FLJ12973 | 0.680048754 | 5.513138175 | 7.02636649 | 7.90E-007 | 1.97E-005 | hypothetical protein FLJ12973 | NA |  | | | | | | | | | | | | | | | | | | | | | | | | | | | |
| 709 | 221570\_s\_at | HSPC133 | 1.833532234 | 8.98233102 | 7.02516095 | 7.92E-007 | 1.97E-005 | HSPC133 protein | NA |  | | | | | | | | | | | | | | | | | | | | | | | | | | | |
| 710 | 212601\_at | ZZEF1 | -0.765116292 | 7.420159806 | -7.01471631 | 8.09E-007 | 2.01E-005 | zinc finger, ZZ-type with EF hand domain 1 | NA |  | | | | | | | | | | | | | | | | | | | | | | | | | | | |
| 711 | 210396\_s\_at | LAT1-3TM | 1.556752103 | 7.952407834 | 7.01449933 | 8.10E-007 | 2.01E-005 | LAT1-3TM protein | NA |  | | | | | | | | | | | | | | | | | | | | | | | | | | | |
| 712 | 221069\_s\_at | LOC51204 | 0.631316244 | 7.215379336 | 7.01204098 | 8.14E-007 | 2.02E-005 | clone HQ0477 PRO0477p | NA |  | | | | | | | | | | | | | | | | | | | | | | | | | | | |
| 713 | 219207\_at | FLJ21128 | 0.904255215 | 6.862944934 | 7.00963678 | 8.18E-007 | 2.03E-005 | hypothetical protein FLJ21128 | NA |  | | | | | | | | | | | | | | | | | | | | | | | | | | | |
| 714 | 202169\_s\_at | AASDHPPT | 1.853605281 | 8.100843844 | 7.0048467 | 8.26E-007 | 2.05E-005 | aminoadipate-semialdehyde dehydrogenase-phosphopantetheinyl transferase | fatty acid biosynthesis |  | | | | | | | | | | | | | | | | | | | | | | | | | | | |
| 715 | 213039\_at | ARHGEF18 | -1.157505049 | 9.811842871 | -7.00357187 | 8.28E-007 | 2.05E-005 | rho/rac guanine nucleotide exchange factor (GEF) 18 | NA |  | | | | | | | | | | | | | | | | | | | | | | | | | | | |
| 716 | 218213\_s\_at | C11orf10 | 0.941502011 | 10.6347149 | 6.99739074 | 8.39E-007 | 2.07E-005 | chromosome 11 open reading frame 10 | NA |  | | | | | | | | | | | | | | | | | | | | | | | | | | | |
| 717 | 209059\_s\_at | EDF1 | 1.097146849 | 8.760886853 | 6.99246422 | 8.48E-007 | 2.09E-005 | endothelial differentiation-related factor 1 | cell growth and/or maintenance, development, regulation of transcription, DNA-dependent |  | | | | | | | | | | | | | | | | | | | | | | | | | | | |
| 718 | 203782\_s\_at | POLRMT | 1.349350694 | 7.642481244 | 6.99006058 | 8.52E-007 | 2.10E-005 | polymerase (RNA) mitochondrial (DNA directed) | DNA replication, synthesis of RNA primer, transcription |  | | | | | | | | | | | | | | | | | | | | | | | | | | | |
| 719 | 218316\_at | TIMM9 | 1.85977644 | 7.91844871 | 6.98747729 | 8.56E-007 | 2.11E-005 | translocase of inner mitochondrial membrane 9 homolog (yeast) | mitochondrial inner membrane protein import, perception of sound, protein transport |  | | | | | | | | | | | | | | | | | | | | | | | | | | | |
| 720 | 222041\_at | OVCA2 /// DPH2L1 | -0.535323946 | 6.394225074 | -6.98308663 | 8.64E-007 | 2.12E-005 | candidate tumor suppressor in ovarian cancer 2 /// DPH2-like 1 (S. cerevisiae) | protein biosynthesis |  | | | | | | | | | | | | | | | | | | | | | | | | | | | |
| 721 | 219259\_at | SEMA4A | -1.515030745 | 9.094998652 | -6.98095049 | 8.68E-007 | 2.12E-005 | sema domain, immunoglobulin domain (Ig), transmembrane domain (TM) and short cytoplasmic domain, (semaphorin) 4A | development, neurogenesis |  | | | | | | | | | | | | | | | | | | | | | | | | | | | |
| 722 | 201232\_s\_at | PSMD13 | 1.071950676 | 8.355349585 | 6.98065079 | 8.69E-007 | 2.12E-005 | proteasome (prosome, macropain) 26S subunit, non-ATPase, 13 | NA |  | | | | | | | | | | | | | | | | | | | | | | | | | | | |
| 723 | 201713\_s\_at | RANBP2 | 1.385771587 | 7.30728484 | 6.97710671 | 8.75E-007 | 2.13E-005 | RAN binding protein 2 | protein folding, protein-nucleus import, transport |  | | | | | | | | | | | | | | | | | | | | | | | | | | | |
| 724 | 220066\_at | CARD15 | -1.903857936 | 6.622918776 | -6.96905842 | 8.90E-007 | 2.16E-005 | caspase recruitment domain family, member 15 | regulation of apoptosis |  | | | | | | | | | | | | | | | | | | | | | | | | | | | |
| 725 | 202190\_at | CSTF1 | 0.979558864 | 7.210197967 | 6.96812922 | 8.92E-007 | 2.16E-005 | cleavage stimulation factor, 3' pre-RNA, subunit 1, 50kDa | RNA processing, mRNA cleavage, mRNA polyadenylylation |  | | | | | | | | | | | | | | | | | | | | | | | | | | | |
| 726 | 209461\_x\_at | WDR18 | 1.334647269 | 7.22918402 | 6.96038905 | 9.06E-007 | 2.20E-005 | WD repeat domain 18 | NA |  | | | | | | | | | | | | | | | | | | | | | | | | | | | |
| 727 | 204695\_at | CDC25A | 1.735320538 | 7.777283153 | 6.95116187 | 9.24E-007 | 2.24E-005 | cell division cycle 25A | cytokinesis, mitosis, protein amino acid dephosphorylation, regulation of cyclin dependent protein kinase activity |  | | | | | | | | | | | | | | | | | | | | | | | | | | | |
| 728 | 221587\_s\_at | C19orf24 | 0.628964796 | 7.890348639 | 6.94874677 | 9.28E-007 | 2.24E-005 | chromosome 19 open reading frame 24 | NA |  | | | | | | | | | | | | | | | | | | | | | | | | | | | |
| 729 | 202679\_at | NPC1 | 1.049890197 | 7.213302866 | 6.94851886 | 9.29E-007 | 2.24E-005 | Niemann-Pick disease, type C1 | cholesterol transport, intracellular protein transport |  | | | | | | | | | | | | | | | | | | | | | | | | | | | |
| 730 | 216713\_at | CCM1 | 0.98863233 | 5.2830037 | 6.94825598 | 9.29E-007 | 2.24E-005 | cerebral cavernous malformations 1 | small GTPase mediated signal transduction |  | | | | | | | | | | | | | | | | | | | | | | | | | | | |
| 731 | 221079\_s\_at | METTL2 /// FLJ12760 | 0.869644473 | 4.422773679 | 6.94380751 | 9.38E-007 | 2.26E-005 | methyltransferase like 2 /// hypothetical protein FLJ12760 | NA |  | | | | | | | | | | | | | | | | | | | | | | | | | | | |
| 732 | 218919\_at | FLJ14007 | 1.420997955 | 7.351182406 | 6.94079565 | 9.44E-007 | 2.27E-005 | hypothetical protein FLJ14007 | NA |  | | | | | | | | | | | | | | | | | | | | | | | | | | | |
| 733 | 201306\_s\_at | ANP32B | 0.906847028 | 11.82648502 | 6.93390682 | 9.57E-007 | 2.30E-005 | acidic (leucine-rich) nuclear phosphoprotein 32 family, member B | NA |  | | | | | | | | | | | | | | | | | | | | | | | | | | | |
| 734 | 202743\_at | PIK3R3 | 0.650298958 | 5.848964845 | 6.92747557 | 9.70E-007 | 2.32E-005 | phosphoinositide-3-kinase, regulatory subunit 3 (p55, gamma) | insulin receptor signaling pathway, intracellular signaling cascade |  | | | | | | | | | | | | | | | | | | | | | | | | | | | |
| 735 | 218831\_s\_at | FCGRT | -2.033972503 | 8.623157123 | -6.92669174 | 9.72E-007 | 2.33E-005 | Fc fragment of IgG, receptor, transporter, alpha | immune response, pregnancy |  | | | | | | | | | | | | | | | | | | | | | | | | | | | |
| 736 | 202758\_s\_at | RFXANK | 0.57165563 | 8.233850408 | 6.91994896 | 9.86E-007 | 2.35E-005 | regulatory factor X-associated ankyrin-containing protein | humoral immune response, regulation of transcription, DNA-dependent, transcription from Pol II promoter |  | | | | | | | | | | | | | | | | | | | | | | | | | | | |
| 737 | 212619\_at | KIAA0286 | 1.416775099 | 6.38288187 | 6.9177794 | 9.90E-007 | 2.36E-005 | KIAA0286 protein | NA |  | | | | | | | | | | | | | | | | | | | | | | | | | | | |
| 738 | 203095\_at | MTIF2 | 2.093255853 | 8.189634349 | 6.9154054 | 9.95E-007 | 2.36E-005 | mitochondrial translational initiation factor 2 | protein biosynthesis, regulation of translational initiation |  | | | | | | | | | | | | | | | | | | | | | | | | | | | |
| 739 | 208289\_s\_at | EI24 | 1.159789625 | 7.436962696 | 6.91528641 | 9.95E-007 | 2.36E-005 | etoposide induced 2.4 mRNA | induction of apoptosis |  | | | | | | | | | | | | | | | | | | | | | | | | | | | |
| 740 | 218504\_at | FAHD2A | 1.519237552 | 7.438569867 | 6.9149607 | 9.96E-007 | 2.36E-005 | fumarylacetoacetate hydrolase domain containing 2A | metabolism |  | | | | | | | | | | | | | | | | | | | | | | | | | | | |
| 741 | 213943\_at | TWIST1 | 2.096156326 | 5.032935522 | 6.91176156 | 1.00E-006 | 2.38E-005 | twist homolog 1 (acrocephalosyndactyly 3; Saethre-Chotzen syndrome) (Drosophila) | cell differentiation, chromosome organization and biogenesis (sensu Eukaryota), morphogenesis, negative regulation of transcription from Pol II promoter, regulation of transcription, DNA-dependent, skeletal development |  | | | | | | | | | | | | | | | | | | | | | | | | | | | |
| 742 | 208666\_s\_at | ST13 | 1.766325093 | 6.639758878 | 6.91063556 | 1.00E-006 | 2.38E-005 | suppression of tumorigenicity 13 (colon carcinoma) (Hsp70 interacting protein) | protein folding |  | | | | | | | | | | | | | | | | | | | | | | | | | | | |
| 743 | 46256\_at | SSB3 | -0.658673859 | 9.551396331 | -6.91035176 | 1.01E-006 | 2.38E-005 | SPRY domain-containing SOCS box protein SSB-3 | intracellular signaling cascade |  | | | | | | | | | | | | | | | | | | | | | | | | | | | |
| 744 | 209674\_at | CRY1 | 1.345330288 | 5.217507827 | 6.90609343 | 1.01E-006 | 2.40E-005 | cryptochrome 1 (photolyase-like) | DNA repair, circadian rhythm, visual perception |  | | | | | | | | | | | | | | | | | | | | | | | | | | | |
| 745 | 218575\_at | ANAPC1 | 1.208164335 | 8.195463326 | 6.90217813 | 1.02E-006 | 2.41E-005 | anaphase promoting complex subunit 1 | cytokinesis, mitosis, regulation of cell cycle, ubiquitin cycle |  | | | | | | | | | | | | | | | | | | | | | | | | | | | |
| 746 | 218491\_s\_at | THY28 | 0.966109265 | 8.286843888 | 6.89835292 | 1.03E-006 | 2.43E-005 | thymocyte protein thy28 | NA |  | | | | | | | | | | | | | | | | | | | | | | | | | | | |
| 747 | 212286\_at | ANKRD12 | -1.077932442 | 7.092329606 | -6.89831659 | 1.03E-006 | 2.43E-005 | ankyrin repeat domain 12 | NA |  | | | | | | | | | | | | | | | | | | | | | | | | | | | |
| 748 | 214711\_at | 15E1.2 | 0.678316486 | 6.158961331 | 6.89779581 | 1.03E-006 | 2.43E-005 | hypothetical protein 15E1.2 | protein biosynthesis, regulation of translational fidelity |  | | | | | | | | | | | | | | | | | | | | | | | | | | | |
| 749 | 221692\_s\_at | MRPL34 | 1.289327472 | 7.621182626 | 6.89201049 | 1.04E-006 | 2.45E-005 | mitochondrial ribosomal protein L34 /// mitochondrial ribosomal protein L34 | protein biosynthesis |  | | | | | | | | | | | | | | | | | | | | | | | | | | | |
| 750 | 221512\_at | DKFZP564D0478 | 0.740241673 | 7.969032995 | 6.88305895 | 1.06E-006 | 2.49E-005 | hypothetical protein DKFZp564D0478 | NA |  | | | | | | | | | | | | | | | | | | | | | | | | | | | |
| 751 | 202043\_s\_at | SMS | 2.125063382 | 7.083190739 | 6.88252145 | 1.07E-006 | 2.49E-005 | spermine synthase | methionine metabolism, polyamine metabolism |  | | | | | | | | | | | | | | | | | | | | | | | | | | | |
| 752 | 204072\_s\_at | 13CDNA73 | -2.327740035 | 7.26617859 | -6.87717519 | 1.08E-006 | 2.51E-005 | hypothetical protein CG003 | NA |  | | | | | | | | | | | | | | | | | | | | | | | | | | | |
| 753 | 213175\_s\_at | SNRPB | 1.451704592 | 10.59886056 | 6.87664893 | 1.08E-006 | 2.51E-005 | small nuclear ribonucleoprotein polypeptides B and B1 | RNA splicing, mRNA processing, nuclear mRNA splicing, via spliceosome |  | | | | | | | | | | | | | | | | | | | | | | | | | | | |
| 754 | 212717\_at | PLEKHM1 | -1.262697841 | 8.104654168 | -6.87354289 | 1.09E-006 | 2.53E-005 | pleckstrin homology domain containing, family M (with RUN domain) member 1 | intracellular signaling cascade |  | | | | | | | | | | | | | | | | | | | | | | | | | | | |
| 755 | 218896\_s\_at | HSA277841 | -0.987964267 | 7.569098372 | -6.87200506 | 1.09E-006 | 2.53E-005 | ELG protein | NA |  | | | | | | | | | | | | | | | | | | | | | | | | | | | |
| 756 | 204127\_at | RFC3 | 1.978459632 | 7.537396693 | 6.86889898 | 1.10E-006 | 2.54E-005 | replication factor C (activator 1) 3, 38kDa | DNA replication, DNA strand elongation |  | | | | | | | | | | | | | | | | | | | | | | | | | | | |
| 757 | 207165\_at | HMMR | 2.153054416 | 7.003131026 | 6.86658656 | 1.10E-006 | 2.55E-005 | hyaluronan-mediated motility receptor (RHAMM) | cell motility |  | | | | | | | | | | | | | | | | | | | | | | | | | | | |
| 758 | 200786\_at | PSMB7 | 1.062105871 | 10.07111528 | 6.86534942 | 1.10E-006 | 2.56E-005 | proteasome (prosome, macropain) subunit, beta type, 7 | ubiquitin-dependent protein catabolism |  | | | | | | | | | | | | | | | | | | | | | | | | | | | |
| 759 | 202614\_at | SLC30A9 | 1.453187806 | 7.295146781 | 6.85982765 | 1.12E-006 | 2.58E-005 | solute carrier family 30 (zinc transporter), member 9 | cation transport |  | | | | | | | | | | | | | | | | | | | | | | | | | | | |
| 760 | 201113\_at | TUFM | 1.457850617 | 10.33123626 | 6.85778167 | 1.12E-006 | 2.59E-005 | Tu translation elongation factor, mitochondrial | protein biosynthesis, translational elongation |  | | | | | | | | | | | | | | | | | | | | | | | | | | | |
| 761 | 221834\_at | LONP | -1.201308582 | 6.859840213 | -6.84133067 | 1.16E-006 | 2.68E-005 | Peroxisomal lon protease | ATP-dependent proteolysis |  | | | | | | | | | | | | | | | | | | | | | | | | | | | |
| 762 | 219182\_at | CHST5 | 0.673393217 | 6.641959255 | 6.83996624 | 1.16E-006 | 2.68E-005 | carbohydrate (N-acetylglucosamine 6-O) sulfotransferase 5 | NA |  | | | | | | | | | | | | | | | | | | | | | | | | | | | |
| 763 | 205382\_s\_at | DF | -3.817510456 | 9.638384781 | -6.83983116 | 1.17E-006 | 2.68E-005 | D component of complement (adipsin) | complement activation, alternative pathway, proteolysis and peptidolysis |  | | | | | | | | | | | | | | | | | | | | | | | | | | | |
| 764 | 212623\_at | KIAA0033 | 0.854901571 | 7.399051434 | 6.83426394 | 1.18E-006 | 2.71E-005 | KIAA0033 protein | NA |  | | | | | | | | | | | | | | | | | | | | | | | | | | | |
| 765 | 204224\_s\_at | GCH1 | -3.174649843 | 7.403751559 | -6.83077284 | 1.19E-006 | 2.73E-005 | GTP cyclohydrolase 1 (dopa-responsive dystonia) | L-phenylalanine catabolism, neurotransmitter metabolism, nitric oxide biosynthesis, tetrahydrobiopterin biosynthesis |  | | | | | | | | | | | | | | | | | | | | | | | | | | | |
| 766 | 201472\_at | VBP1 | 1.293982044 | 9.57562638 | 6.82928453 | 1.19E-006 | 2.73E-005 | von Hippel-Lindau binding protein 1 | NA |  | | | | | | | | | | | | | | | | | | | | | | | | | | | |
| 767 | 218935\_at | EHD3 | -0.516196498 | 5.16781803 | -6.82547395 | 1.20E-006 | 2.75E-005 | EH-domain containing 3 | NA |  | | | | | | | | | | | | | | | | | | | | | | | | | | | |
| 768 | 212635\_at | TNPO1 | 0.848504565 | 7.245954286 | 6.82447266 | 1.20E-006 | 2.75E-005 | Transportin 1 | protein transport, protein-nucleus import, docking, protein-nucleus import, translocation |  | | | | | | | | | | | | | | | | | | | | | | | | | | | |
| 769 | 209879\_at | SELPLG | -2.238778874 | 9.134377066 | -6.82382524 | 1.20E-006 | 2.75E-005 | selectin P ligand | cell adhesion |  | | | | | | | | | | | | | | | | | | | | | | | | | | | |
| 770 | 211662\_s\_at | VDAC2 | 1.755729343 | 10.34024213 | 6.82331129 | 1.21E-006 | 2.75E-005 | voltage-dependent anion channel 2 /// voltage-dependent anion channel 2 | anion transport |  | | | | | | | | | | | | | | | | | | | | | | | | | | | |
| 771 | 208825\_x\_at | RPL23A | 0.437436329 | 13.72419561 | 6.82011474 | 1.21E-006 | 2.77E-005 | ribosomal protein L23a | NA |  | | | | | | | | | | | | | | | | | | | | | | | | | | | |
| 772 | 221931\_s\_at | SEH1L | 2.809779038 | 6.920018004 | 6.81141372 | 1.24E-006 | 2.81E-005 | SEH1-like (S. cerevisiae) | protein transport |  | | | | | | | | | | | | | | | | | | | | | | | | | | | |
| 773 | 210802\_s\_at | HSA9761 | 1.710085559 | 7.102603472 | 6.80971674 | 1.24E-006 | 2.82E-005 | putative dimethyladenosine transferase | rRNA modification, rRNA processing |  | | | | | | | | | | | | | | | | | | | | | | | | | | | |
| 774 | 218438\_s\_at | EG1 | 1.34894706 | 8.33307006 | 6.8088512 | 1.24E-006 | 2.82E-005 | endothelial-derived gene 1 | NA |  | | | | | | | | | | | | | | | | | | | | | | | | | | | |
| 775 | 219294\_at | C6orf139 | 0.815259319 | 4.248988734 | 6.80494874 | 1.25E-006 | 2.84E-005 | chromosome 6 open reading frame 139 | NA |  | | | | | | | | | | | | | | | | | | | | | | | | | | | |
| 776 | 218074\_at | CGI-128 | 1.118185547 | 9.116283827 | 6.80411229 | 1.26E-006 | 2.84E-005 | CGI-128 protein | NA |  | | | | | | | | | | | | | | | | | | | | | | | | | | | |
| 777 | 213338\_at | RIS1 | 1.995514429 | 6.676655171 | 6.80184309 | 1.26E-006 | 2.85E-005 | Ras-induced senescence 1 | NA |  | | | | | | | | | | | | | | | | | | | | | | | | | | | |
| 778 | 208117\_s\_at | FLJ12525 | 0.881590792 | 8.528724493 | 6.79921908 | 1.27E-006 | 2.87E-005 | hypothetical protein FLJ12525 /// hypothetical protein FLJ12525 | NA |  | | | | | | | | | | | | | | | | | | | | | | | | | | | |
| 779 | 219665\_at | FLJ22494 | -0.560822104 | 6.703619295 | -6.79904729 | 1.27E-006 | 2.87E-005 | hypothetical protein FLJ22494 | NA |  | | | | | | | | | | | | | | | | | | | | | | | | | | | |
| 780 | 217919\_s\_at | MRPL42 | 1.701649692 | 8.600788509 | 6.79821673 | 1.27E-006 | 2.87E-005 | mitochondrial ribosomal protein L42 | protein biosynthesis |  | | | | | | | | | | | | | | | | | | | | | | | | | | | |
| 781 | 202097\_at | NUP153 | 1.379133146 | 8.305637907 | 6.79724275 | 1.27E-006 | 2.87E-005 | nucleoporin 153kDa | transport |  | | | | | | | | | | | | | | | | | | | | | | | | | | | |
| 782 | 204192\_at | CD37 | -2.59368561 | 8.908861739 | -6.78948408 | 1.29E-006 | 2.91E-005 | CD37 antigen | N-linked glycosylation |  | | | | | | | | | | | | | | | | | | | | | | | | | | | |
| 783 | 210982\_s\_at | HLA-DRA | -4.859887216 | 9.207175457 | -6.78924098 | 1.30E-006 | 2.91E-005 | major histocompatibility complex, class II, DR alpha | antigen presentation, exogenous antigen, antigen processing, exogenous antigen via MHC class II, immune response |  | | | | | | | | | | | | | | | | | | | | | | | | | | | |
| 784 | 200039\_s\_at | PSMB2 | 1.231884518 | 9.896548029 | 6.7846354 | 1.31E-006 | 2.93E-005 | proteasome (prosome, macropain) subunit, beta type, 2 /// proteasome (prosome, macropain) subunit, beta type, 2 | ubiquitin-dependent protein catabolism |  | | | | | | | | | | | | | | | | | | | | | | | | | | | |
| 785 | 218602\_s\_at | FAM29A | 0.537286289 | 3.70468852 | 6.78437051 | 1.31E-006 | 2.93E-005 | family with sequence similarity 29, member A | NA |  | | | | | | | | | | | | | | | | | | | | | | | | | | | |
| 786 | 209095\_at | DLD | 1.526013533 | 9.283156118 | 6.78325158 | 1.31E-006 | 2.93E-005 | dihydrolipoamide dehydrogenase (E3 component of pyruvate dehydrogenase complex, 2-oxo-glutarate complex, branched chain keto acid dehydrogenase complex) | electron transport, energy pathways, glycolysis |  | | | | | | | | | | | | | | | | | | | | | | | | | | | |
| 787 | 214177\_s\_at | PBXIP1 | -1.370068961 | 8.518070336 | -6.78301796 | 1.31E-006 | 2.93E-005 | pre-B-cell leukemia transcription factor interacting protein 1 | cell differentiation, negative regulation of transcription |  | | | | | | | | | | | | | | | | | | | | | | | | | | | |
| 788 | 201016\_at | EIF1AX | 1.713619529 | 6.77590028 | 6.78076824 | 1.32E-006 | 2.94E-005 | eukaryotic translation initiation factor 1A, X-linked | protein biosynthesis, translational initiation |  | | | | | | | | | | | | | | | | | | | | | | | | | | | |
| 789 | 221227\_x\_at | COQ3 | 0.859301912 | 7.0899193 | 6.78009684 | 1.32E-006 | 2.95E-005 | coenzyme Q3 homolog, methyltransferase (yeast) /// coenzyme Q3 homolog, methyltransferase (yeast) | ubiquinone biosynthesis |  | | | | | | | | | | | | | | | | | | | | | | | | | | | |
| 790 | 203973\_s\_at | CEBPD | -2.971328144 | 10.48971918 | -6.77767789 | 1.33E-006 | 2.96E-005 | CCAAT/enhancer binding protein (C/EBP), delta | regulation of transcription, DNA-dependent, transcription from Pol II promoter |  | | | | | | | | | | | | | | | | | | | | | | | | | | | |
| 791 | 215991\_s\_at | KIAA0090 | 0.479630046 | 4.714667573 | 6.77744492 | 1.33E-006 | 2.96E-005 | KIAA0090 protein | NA |  | | | | | | | | | | | | | | | | | | | | | | | | | | | |
| 792 | 212249\_at | PIK3R1 | -1.833791597 | 7.425278824 | -6.76586303 | 1.36E-006 | 3.02E-005 | phosphoinositide-3-kinase, regulatory subunit 1 (p85 alpha) | intracellular signaling cascade |  | | | | | | | | | | | | | | | | | | | | | | | | | | | |
| 793 | 212789\_at | KIAA0056 | 1.230809483 | 7.415396772 | 6.76087954 | 1.38E-006 | 3.05E-005 | KIAA0056 protein | NA |  | | | | | | | | | | | | | | | | | | | | | | | | | | | |
| 794 | 209520\_s\_at | NCBP1 | 1.473929356 | 6.163845979 | 6.75859455 | 1.38E-006 | 3.06E-005 | nuclear cap binding protein subunit 1, 80kDa | RNA splicing, mRNA processing, mRNA-nucleus export, protein biosynthesis, transport |  | | | | | | | | | | | | | | | | | | | | | | | | | | | |
| 795 | 213518\_at | PRKCI | 1.768163109 | 6.400556563 | 6.75577416 | 1.39E-006 | 3.07E-005 | protein kinase C, iota | cytoskeleton organization and biogenesis, establishment and/or maintenance of epithelial cell polarity, intercellular junction assembly and/or maintenance, intracellular signaling cascade, membrane organization and biogenesis, protein amino acid phosphorylation, protein-membrane targeting, secretion, vesicle-mediated transport | | |  | | | | | | | | | | | | | | | | | | | | | | | | | |
| 796 | 202207\_at | ARL7 | -2.527132183 | 7.328686459 | -6.75565038 | 1.39E-006 | 3.07E-005 | ADP-ribosylation factor-like 7 | small GTPase mediated signal transduction |  | | | | | | | | | | | | | | | | | | | | | | | | | | | |
| 797 | 205668\_at | LY75 | -1.955037801 | 6.964366158 | -6.75166999 | 1.40E-006 | 3.09E-005 | lymphocyte antigen 75 | endocytosis, immune response, inflammatory response |  | | | | | | | | | | | | | | | | | | | | | | | | | | | |
| 798 | 200905\_x\_at | HLA-E | -1.975817041 | 11.30203602 | -6.7515417 | 1.40E-006 | 3.09E-005 | major histocompatibility complex, class I, E | antigen presentation, endogenous antigen, antigen processing, endogenous antigen via MHC class I, immune response |  | | | | | | | | | | | | | | | | | | | | | | | | | | | |
| 799 | 203723\_at | ITPKB | -1.021139332 | 7.280385438 | -6.74961859 | 1.41E-006 | 3.10E-005 | inositol 1,4,5-trisphosphate 3-kinase B | signal transduction |  | | | | | | | | | | | | | | | | | | | | | | | | | | | |
| 800 | 204033\_at | TRIP13 | 1.74590268 | 7.579279057 | 6.74753103 | 1.41E-006 | 3.11E-005 | thyroid hormone receptor interactor 13 | transcription from Pol II promoter |  | | | | | | | | | | | | | | | | | | | | | | | | | | | |
| 801 | 206342\_x\_at | IDS | -1.379920143 | 8.045331148 | -6.74720538 | 1.42E-006 | 3.11E-005 | iduronate 2-sulfatase (Hunter syndrome) | glycosaminoglycan metabolism, metabolism |  | | | | | | | | | | | | | | | | | | | | | | | | | | | |
| 802 | 203758\_at | CTSO | -1.3087072 | 6.83664488 | -6.74585299 | 1.42E-006 | 3.12E-005 | cathepsin O | proteolysis and peptidolysis |  | | | | | | | | | | | | | | | | | | | | | | | | | | | |
| 803 | 209312\_x\_at | HLA-DRB1 | -4.501995674 | 9.536309111 | -6.74383808 | 1.43E-006 | 3.13E-005 | major histocompatibility complex, class II, DR beta 1 /// major histocompatibility complex, class II, DR beta 1 | antigen presentation, exogenous antigen, antigen processing, exogenous antigen via MHC class II, immune response |  | | | | | | | | | | | | | | | | | | | | | | | | | | | |
| 804 | 209608\_s\_at | ACAT2 | 1.223618118 | 7.75319106 | 6.74164669 | 1.43E-006 | 3.14E-005 | acetyl-Coenzyme A acetyltransferase 2 (acetoacetyl Coenzyme A thiolase) | NA |  | | | | | | | | | | | | | | | | | | | | | | | | | | | |
| 805 | 209146\_at | SC4MOL | 2.529789357 | 6.253515006 | 6.73993333 | 1.44E-006 | 3.14E-005 | sterol-C4-methyl oxidase-like | fatty acid metabolism, metabolism, steroid metabolism, sterol biosynthesis |  | | | | | | | | | | | | | | | | | | | | | | | | | | | |
| 806 | 204510\_at | CDC7 | 1.188977901 | 6.822916555 | 6.73550081 | 1.45E-006 | 3.16E-005 | CDC7 cell division cycle 7 (S. cerevisiae) | DNA replication initiation, G1/S transition of mitotic cell cycle, cell cycle, cytokinesis, negative regulation of cell proliferation, protein amino acid phosphorylation, traversing start control point of mitotic cell cycle |  | | | | | | | | | | | | | | | | | | | | | | | | | | | |
| 807 | 201085\_s\_at | SON | -1.020336211 | 9.193592103 | -6.73499404 | 1.45E-006 | 3.16E-005 | SON DNA binding protein | anti-apoptosis |  | | | | | | | | | | | | | | | | | | | | | | | | | | | |
| 808 | 202040\_s\_at | JARID1A | -0.799621637 | 8.169316297 | -6.73396143 | 1.46E-006 | 3.17E-005 | Jumonji, AT rich interactive domain 1A (RBBP2-like) | regulation of transcription, DNA-dependent, transcription from Pol II promoter |  | | | | | | | | | | | | | | | | | | | | | | | | | | | |
| 809 | 202839\_s\_at | NDUFB7 | 1.216580243 | 7.103114962 | 6.73047574 | 1.47E-006 | 3.19E-005 | NADH dehydrogenase (ubiquinone) 1 beta subcomplex, 7, 18kDa | electron transport |  | | | | | | | | | | | | | | | | | | | | | | | | | | | |
| 810 | 212308\_at | CLASP2 | 0.656856828 | 4.29809845 | 6.72539656 | 1.48E-006 | 3.21E-005 | cytoplasmic linker associated protein 2 | NA |  | | | | | | | | | | | | | | | | | | | | | | | | | | | |
| 811 | 219148\_at | TOPK | 2.517211831 | 5.578662394 | 6.72474329 | 1.48E-006 | 3.21E-005 | T-LAK cell-originated protein kinase | mitosis, protein amino acid phosphorylation |  | | | | | | | | | | | | | | | | | | | | | | | | | | | |
| 812 | 204809\_at | CLPX | 1.27452684 | 6.703467816 | 6.71918416 | 1.50E-006 | 3.25E-005 | ClpX caseinolytic protease X homolog (E. coli) | protein folding, protein transport |  | | | | | | | | | | | | | | | | | | | | | | | | | | | |
| 813 | 212980\_at | AHSA2 | -1.47705658 | 6.102320214 | -6.71503875 | 1.51E-006 | 3.27E-005 | AHA1, activator of heat shock 90kDa protein ATPase homolog 2 (yeast) | NA |  | | | | | | | | | | | | | | | | | | | | | | | | | | | |
| 814 | 207186\_s\_at | FALZ | -1.10351903 | 8.458967177 | -6.71469269 | 1.52E-006 | 3.27E-005 | fetal Alzheimer antigen | electron transport, neurogenesis, regulation of transcription, DNA-dependent |  | | | | | | | | | | | | | | | | | | | | | | | | | | | |
| 815 | 202115\_s\_at | DKFZP564C186 | 1.013075821 | 7.954758262 | 6.71372294 | 1.52E-006 | 3.28E-005 | DKFZP564C186 protein | NA |  | | | | | | | | | | | | | | | | | | | | | | | | | | | |
| 816 | 201477\_s\_at | RRM1 | 1.430007995 | 8.701563324 | 6.71046245 | 1.53E-006 | 3.30E-005 | ribonucleotide reductase M1 polypeptide | DNA replication |  | | | | | | | | | | | | | | | | | | | | | | | | | | | |
| 817 | 201104\_x\_at | MGC8902 /// LOC376745 /// DJ328E19.C1.1 /// LOC200030 | -0.877745132 | 9.748470043 | -6.70333536 | 1.55E-006 | 3.34E-005 | hypothetical protein MGC8902 /// AG1 /// hypothetical protein DJ328E19.C1.1 /// hypothetical protein LOC200030 | NA |  | | | | | | | | | | | | | | | | | | | | | | | | | | | |
| 818 | 208802\_at | SRP72 | 1.943724421 | 7.075356015 | 6.69747449 | 1.57E-006 | 3.38E-005 | signal recognition particle 72kDa | protein amino acid phosphorylation, signal transduction |  | | | | | | | | | | | | | | | | | | | | | | | | | | | |
| 819 | 218110\_at | XAB2 | 0.901334908 | 6.988495709 | 6.69554863 | 1.58E-006 | 3.39E-005 | XPA binding protein 2 | DNA repair, RNA processing, regulation of transcription, DNA-dependent, transcription, transcription-coupled nucleotide-excision repair |  | | | | | | | | | | | | | | | | | | | | | | | | | | | |
| 820 | 202708\_s\_at | HIST2H2BE | -2.648011447 | 8.199387098 | -6.69490563 | 1.58E-006 | 3.39E-005 | histone 2, H2be | chromosome organization and biogenesis (sensu Eukaryota), nucleosome assembly |  | | | | | | | | | | | | | | | | | | | | | | | | | | | |
| 821 | 201708\_s\_at | NIPSNAP1 | 1.06182877 | 7.78254333 | 6.69415412 | 1.58E-006 | 3.39E-005 | nipsnap homolog 1 (C. elegans) | NA |  | | | | | | | | | | | | | | | | | | | | | | | | | | | |
| 822 | 204616\_at | UCHL3 | 1.254161603 | 8.395170659 | 6.69322097 | 1.59E-006 | 3.39E-005 | ubiquitin carboxyl-terminal esterase L3 (ubiquitin thiolesterase) | ubiquitin cycle, ubiquitin-dependent protein catabolism |  | | | | | | | | | | | | | | | | | | | | | | | | | | | |
| 823 | 200978\_at | MDH1 | 1.224383243 | 10.41379214 | 6.69117167 | 1.59E-006 | 3.40E-005 | malate dehydrogenase 1, NAD (soluble) | tricarboxylic acid cycle |  | | | | | | | | | | | | | | | | | | | | | | | | | | | |
| 824 | 219979\_s\_at | HSPC138 | 1.322913521 | 5.052502434 | 6.69108795 | 1.59E-006 | 3.40E-005 | hypothetical protein HSPC138 | NA |  | | | | | | | | | | | | | | | | | | | | | | | | | | | |
| 825 | 201478\_s\_at | DKC1 | 1.92855492 | 8.723900474 | 6.68922852 | 1.60E-006 | 3.40E-005 | dyskeratosis congenita 1, dyskerin | cell proliferation, rRNA processing, regulation of cell cycle, telomerase-dependent telomere maintenance |  | | | | | | | | | | | | | | | | | | | | | | | | | | | |
| 826 | 218236\_s\_at | PRKD3 | 2.019687005 | 6.383168935 | 6.68907559 | 1.60E-006 | 3.40E-005 | protein kinase D3 | intracellular signaling cascade, protein amino acid phosphorylation, protein kinase C activation |  | | | | | | | | | | | | | | | | | | | | | | | | | | | |
| 827 | 204775\_at | CHAF1B | 0.557223179 | 6.567626871 | 6.6852607 | 1.61E-006 | 3.43E-005 | chromatin assembly factor 1, subunit B (p60) | DNA repair, DNA replication, DNA replication-dependent nucleosome assembly, G-protein coupled receptor protein signaling pathway, cell cycle, protein complex assembly, regulation of transcription, DNA-dependent |  | | | | | | | | | | | | | | | | | | | | | | | | | | | |
| 828 | 212532\_s\_at | FLJ30656 | 0.800056035 | 8.416713451 | 6.68477626 | 1.61E-006 | 3.43E-005 | hypothetical protein FLJ30656 | NA |  | | | | | | | | | | | | | | | | | | | | | | | | | | | |
| 829 | 217901\_at | DSG2 | 1.712806941 | 5.499487155 | 6.68273651 | 1.62E-006 | 3.44E-005 | Desmoglein 2 | cell adhesion, homophilic cell adhesion |  | | | | | | | | | | | | | | | | | | | | | | | | | | | |
| 830 | 202564\_x\_at | ARL2 | 0.919441865 | 8.331731038 | 6.68258546 | 1.62E-006 | 3.44E-005 | ADP-ribosylation factor-like 2 | small GTPase mediated signal transduction, tubulin folding |  | | | | | | | | | | | | | | | | | | | | | | | | | | | |
| 831 | 209595\_at | GTF2F2 | 1.014434541 | 4.88012246 | 6.6813581 | 1.63E-006 | 3.44E-005 | general transcription factor IIF, polypeptide 2, 30kDa | RNA elongation from Pol II promoter, regulation of transcription, DNA-dependent, transcription initiation from Pol II promoter |  | | | | | | | | | | | | | | | | | | | | | | | | | | | |
| 832 | 201717\_at | MRPL49 | 0.782566623 | 9.030725217 | 6.68110443 | 1.63E-006 | 3.44E-005 | mitochondrial ribosomal protein L49 | cell growth and/or maintenance, protein biosynthesis, translational initiation |  | | | | | | | | | | | | | | | | | | | | | | | | | | | |
| 833 | 221700\_s\_at | UBA52 | 0.702054627 | 12.01529864 | 6.68079638 | 1.63E-006 | 3.44E-005 | ubiquitin A-52 residue ribosomal protein fusion product 1 /// ubiquitin A-52 residue ribosomal protein fusion product 1 | NA |  | | | | | | | | | | | | | | | | | | | | | | | | | | | |
| 834 | 217728\_at | S100A6 | -3.413543786 | 10.21042178 | -6.68049372 | 1.63E-006 | 3.44E-005 | S100 calcium binding protein A6 (calcyclin) | axonogenesis, cell-cell signaling, positive regulation of fibroblast proliferation, regulation of cell cycle |  | | | | | | | | | | | | | | | | | | | | | | | | | | | |
| 835 | 200040\_at | KHDRBS1 | 0.913635113 | 10.08093541 | 6.67871095 | 1.64E-006 | 3.45E-005 | KH domain containing, RNA binding, signal transduction associated 1 /// KH domain containing, RNA binding, signal transduction associated 1 | G1/S transition of mitotic cell cycle, Ras protein signal transduction, cell cycle arrest, cell proliferation, mRNA processing, signal transduction |  | | | | | | | | | | | | | | | | | | | | | | | | | | | |
| 836 | 212348\_s\_at | AOF2 | 1.26646233 | 8.487560871 | 6.67360511 | 1.65E-006 | 3.48E-005 | amine oxidase (flavin containing) domain 2 | electron transport, regulation of transcription, DNA-dependent |  | | | | | | | | | | | | | | | | | | | | | | | | | | | |
| 837 | 201317\_s\_at | PSMA2 | 1.331360742 | 10.27414703 | 6.67175448 | 1.66E-006 | 3.49E-005 | proteasome (prosome, macropain) subunit, alpha type, 2 | ubiquitin-dependent protein catabolism |  | | | | | | | | | | | | | | | | | | | | | | | | | | | |
| 838 | 208793\_x\_at | SMARCA4 | 0.849436503 | 8.006966809 | 6.66736921 | 1.68E-006 | 3.52E-005 | SWI/SNF related, matrix associated, actin dependent regulator of chromatin, subfamily a, member 4 | regulation of transcription from Pol II promoter |  | | | | | | | | | | | | | | | | | | | | | | | | | | | |
| 839 | 212411\_at | IMP4 | 1.280329715 | 8.718607737 | 6.66130052 | 1.70E-006 | 3.55E-005 | U3 snoRNP protein 4 homolog | NA |  | | | | | | | | | | | | | | | | | | | | | | | | | | | |
| 840 | 200790\_at | ODC1 | 2.1808628 | 9.790862941 | 6.65846778 | 1.71E-006 | 3.57E-005 | ornithine decarboxylase 1 | polyamine biosynthesis |  | | | | | | | | | | | | | | | | | | | | | | | | | | | |
| 841 | 216242\_x\_at | POLR2J2 | 0.729442049 | 8.372198508 | 6.65814145 | 1.71E-006 | 3.57E-005 | DNA directed RNA polymerase II polypeptide J-related gene | transcription |  | | | | | | | | | | | | | | | | | | | | | | | | | | | |
| 842 | 201648\_at | JAK1 | -1.054955855 | 9.205092913 | -6.65648313 | 1.71E-006 | 3.58E-005 | Janus kinase 1 (a protein tyrosine kinase) | intracellular signaling cascade, protein amino acid phosphorylation |  | | | | | | | | | | | | | | | | | | | | | | | | | | | |
| 843 | 221777\_at | FLJ14827 | 0.425102778 | 8.046615734 | 6.6556487 | 1.72E-006 | 3.58E-005 | hypothetical protein FLJ14827 | NA |  | | | | | | | | | | | | | | | | | | | | | | | | | | | |
| 844 | 209858\_x\_at | MPPE1 | -1.016448139 | 8.751938246 | -6.65445523 | 1.72E-006 | 3.58E-005 | metallophosphoesterase 1 | NA |  | | | | | | | | | | | | | | | | | | | | | | | | | | | |
| 845 | 212145\_at | MRPS27 | 1.629737792 | 8.600986104 | 6.65264974 | 1.73E-006 | 3.60E-005 | mitochondrial ribosomal protein S27 | NA |  | | | | | | | | | | | | | | | | | | | | | | | | | | | |
| 846 | 216305\_s\_at | C2orf3 | 1.167522593 | 5.760432202 | 6.64575579 | 1.75E-006 | 3.64E-005 | chromosome 2 open reading frame 3 | regulation of transcription, DNA-dependent |  | | | | | | | | | | | | | | | | | | | | | | | | | | | |
| 847 | 201112\_s\_at | CSE1L | 1.600955233 | 9.025954825 | 6.64454426 | 1.76E-006 | 3.64E-005 | CSE1 chromosome segregation 1-like (yeast) | apoptosis, cell proliferation, protein transport, protein-nucleus import, docking |  | | | | | | | | | | | | | | | | | | | | | | | | | | | |
| 848 | 207677\_s\_at | NCF4 | -3.077987863 | 8.904972951 | -6.64145392 | 1.77E-006 | 3.66E-005 | neutrophil cytosolic factor 4, 40kDa /// neutrophil cytosolic factor 4, 40kDa | defense response, electron transport, intracellular signaling cascade |  | | | | | | | | | | | | | | | | | | | | | | | | | | | |
| 849 | 209201\_x\_at | CXCR4 | -2.825492708 | 10.41137163 | -6.63427245 | 1.80E-006 | 3.70E-005 | chemokine (C-X-C motif) receptor 4 | G-protein coupled receptor protein signaling pathway |  | | | | | | | | | | | | | | | | | | | | | | | | | | | |
| 850 | 210250\_x\_at | ADSL | 1.514700636 | 9.169289179 | 6.63369704 | 1.80E-006 | 3.70E-005 | adenylosuccinate lyase | purine ribonucleotide biosynthesis |  | | | | | | | | | | | | | | | | | | | | | | | | | | | |
| 851 | 208093\_s\_at | NDEL1 | -1.31153034 | 8.261082372 | -6.63007212 | 1.81E-006 | 3.73E-005 | nudE nuclear distribution gene E homolog like 1 (A. nidulans) /// nudE nuclear distribution gene E homolog like 1 (A. nidulans) | NA |  | | | | | | | | | | | | | | | | | | | | | | | | | | | |
| 852 | 205086\_s\_at | 384D8-2 | 0.985848266 | 6.90221817 | 6.63003311 | 1.81E-006 | 3.73E-005 | hypothetical protein 384D8\_6 | NA |  | | | | | | | | | | | | | | | | | | | | | | | | | | | |
| 853 | 217871\_s\_at | MIF | 2.198668377 | 10.60774783 | 6.62820994 | 1.82E-006 | 3.74E-005 | macrophage migration inhibitory factor (glycosylation-inhibiting factor) | immune response, inflammatory response |  | | | | | | | | | | | | | | | | | | | | | | | | | | | |
| 854 | 211804\_s\_at | CDK2 | 0.863720626 | 7.48040375 | 6.62675603 | 1.83E-006 | 3.75E-005 | cyclin-dependent kinase 2 | G2/M transition of mitotic cell cycle, cell cycle, cytokinesis, mitosis, positive regulation of cell proliferation, protein amino acid phosphorylation, regulation of DNA replication, traversing start control point of mitotic cell cycle |  | | | | | | | | | | | | | | | | | | | | | | | | | | | |
| 855 | 204216\_s\_at | FLJ11806 | 0.600068466 | 8.226356503 | 6.62378075 | 1.84E-006 | 3.76E-005 | nuclear protein UKp68 | NA |  | | | | | | | | | | | | | | | | | | | | | | | | | | | |
| 856 | 217786\_at | SKB1 | 1.807974967 | 8.796461746 | 6.62074042 | 1.85E-006 | 3.78E-005 | SKB1 homolog (S. pombe) | cell proliferation, regulation of mitosis |  | | | | | | | | | | | | | | | | | | | | | | | | | | | |
| 857 | 208018\_s\_at | HCK | -3.858555992 | 8.783493405 | -6.61230777 | 1.88E-006 | 3.84E-005 | hemopoietic cell kinase | intracellular signaling cascade, mesoderm development, protein amino acid phosphorylation |  | | | | | | | | | | | | | | | | | | | | | | | | | | | |
| 858 | 217954\_s\_at | PHF3 | 0.97918478 | 7.165196155 | 6.61032494 | 1.89E-006 | 3.86E-005 | PHD finger protein 3 | development, regulation of transcription, DNA-dependent, transcription |  | | | | | | | | | | | | | | | | | | | | | | | | | | | |
| 859 | 212949\_at | BRRN1 | 1.232395377 | 7.799467064 | 6.60526203 | 1.91E-006 | 3.89E-005 | barren homolog (Drosophila) | mitosis, mitotic cell cycle, mitotic chromosome condensation |  | | | | | | | | | | | | | | | | | | | | | | | | | | | |
| 860 | 212685\_s\_at | TBL2 | 1.274296113 | 7.770325228 | 6.60525174 | 1.91E-006 | 3.89E-005 | transducin (beta)-like 2 | NA |  | | | | | | | | | | | | | | | | | | | | | | | | | | | |
| 861 | 201516\_at | SRM | 1.945236323 | 8.450483217 | 6.60470533 | 1.91E-006 | 3.89E-005 | spermidine synthase | spermidine biosynthesis |  | | | | | | | | | | | | | | | | | | | | | | | | | | | |
| 862 | 200043\_at | ERH | 0.837028822 | 9.789189745 | 6.60296674 | 1.92E-006 | 3.90E-005 | enhancer of rudimentary homolog (Drosophila) /// enhancer of rudimentary homolog (Drosophila) | nucleobase, nucleoside, nucleotide and nucleic acid metabolism, pyrimidine nucleoside metabolism, regulation of cell cycle |  | | | | | | | | | | | | | | | | | | | | | | | | | | | |
| 863 | 217900\_at | FLJ10326 | 1.24262146 | 8.541090441 | 6.60171463 | 1.93E-006 | 3.90E-005 | mitochondrial isoleucine tRNA synthetase | isoleucyl-tRNA aminoacylation |  | | | | | | | | | | | | | | | | | | | | | | | | | | | |
| 864 | 200924\_s\_at | SLC3A2 | 1.346025417 | 9.117985387 | 6.60152147 | 1.93E-006 | 3.90E-005 | solute carrier family 3 (activators of dibasic and neutral amino acid transport), member 2 | amino acid transport, calcium ion transport, carbohydrate metabolism, cell growth |  | | | | | | | | | | | | | | | | | | | | | | | | | | | |
| 865 | 210052\_s\_at | TPX2 | 1.8736644 | 8.250993914 | 6.60104065 | 1.93E-006 | 3.90E-005 | TPX2, microtubule-associated protein homolog (Xenopus laevis) | cell proliferation, mitosis |  | | | | | | | | | | | | | | | | | | | | | | | | | | | |
| 866 | 207700\_s\_at | NCOA3 | -1.167306377 | 7.528745409 | -6.59973671 | 1.93E-006 | 3.91E-005 | nuclear receptor coactivator 3 | regulation of transcription, DNA-dependent, signal transduction, transcription |  | | | | | | | | | | | | | | | | | | | | | | | | | | | |
| 867 | 213572\_s\_at | SERPINB1 | -2.637258729 | 9.480672885 | -6.59860764 | 1.94E-006 | 3.91E-005 | serine (or cysteine) proteinase inhibitor, clade B (ovalbumin), member 1 | NA |  | | | | | | | | | | | | | | | | | | | | | | | | | | | |
| 868 | 200869\_at | RPL18A | 0.994842113 | 12.38885507 | 6.59843941 | 1.94E-006 | 3.91E-005 | ribosomal protein L18a | protein biosynthesis |  | | | | | | | | | | | | | | | | | | | | | | | | | | | |
| 869 | 201705\_at | PSMD7 | 0.97794467 | 8.929426986 | 6.59774397 | 1.94E-006 | 3.92E-005 | proteasome (prosome, macropain) 26S subunit, non-ATPase, 7 (Mov34 homolog) | NA |  | | | | | | | | | | | | | | | | | | | | | | | | | | | |
| 870 | 202850\_at | ABCD3 | 0.875848404 | 7.096162381 | 6.59674582 | 1.95E-006 | 3.92E-005 | ATP-binding cassette, sub-family D (ALD), member 3 | peroxisomal long-chain fatty acid import, peroxisome organization and biogenesis, transport |  | | | | | | | | | | | | | | | | | | | | | | | | | | | |
| 871 | 218303\_x\_at | LOC51315 | -1.459479845 | 7.956606907 | -6.59397496 | 1.96E-006 | 3.94E-005 | hypothetical protein LOC51315 | NA |  | | | | | | | | | | | | | | | | | | | | | | | | | | | |
| 872 | 220046\_s\_at | CCNL1 | -1.809005814 | 9.231636146 | -6.5895146 | 1.98E-006 | 3.97E-005 | cyclin L1 | NA |  | | | | | | | | | | | | | | | | | | | | | | | | | | | |
| 873 | 218461\_at | MGC14560 | 1.466121377 | 7.13341826 | 6.58866535 | 1.98E-006 | 3.97E-005 | protein x 0004 | NA |  | | | | | | | | | | | | | | | | | | | | | | | | | | | |
| 874 | 214246\_x\_at | MINK | -1.018393459 | 8.637303555 | -6.58708804 | 1.99E-006 | 3.98E-005 | misshapen/NIK-related kinase | development, protein amino acid phosphorylation, protein kinase cascade, response to stress |  | | | | | | | | | | | | | | | | | | | | | | | | | | | |
| 875 | 221036\_s\_at | PSFL | -0.753790033 | 7.707778069 | -6.58688171 | 1.99E-006 | 3.98E-005 | anterior pharynx defective 1B-like /// anterior pharynx defective 1B-like | positive regulation of enzyme activity, protein processing |  | | | | | | | | | | | | | | | | | | | | | | | | | | | |
| 876 | 203565\_s\_at | MNAT1 | 0.948653787 | 7.075010407 | 6.57939045 | 2.02E-006 | 4.04E-005 | menage a trois 1 (CAK assembly factor) | DNA repair, cell cycle, protein complex assembly, protein ubiquitination, regulation of cyclin dependent protein kinase activity, regulation of transcription from Pol II promoter |  | | | | | | | | | | | | | | | | | | | | | | | | | | | |
| 877 | 205129\_at | NPM3 | 1.756880599 | 7.87180048 | 6.57877755 | 2.02E-006 | 4.04E-005 | nucleophosmin/nucleoplasmin, 3 | protein folding |  | | | | | | | | | | | | | | | | | | | | | | | | | | | |
| 878 | 201721\_s\_at | LAPTM5 | -2.102822341 | 11.55313812 | -6.57358518 | 2.04E-006 | 4.08E-005 | Lysosomal-associated multispanning membrane protein-5 | NA |  | | | | | | | | | | | | | | | | | | | | | | | | | | | |
| 879 | 200052\_s\_at | ILF2 | 1.874145095 | 8.845178962 | 6.57283549 | 2.05E-006 | 4.09E-005 | interleukin enhancer binding factor 2, 45kDa /// interleukin enhancer binding factor 2, 45kDa | immune response |  | | | | | | | | | | | | | | | | | | | | | | | | | | | |
| 880 | 203663\_s\_at | COX5A | 1.470335769 | 9.903810409 | 6.56785274 | 2.07E-006 | 4.12E-005 | cytochrome c oxidase subunit Va | electron transport |  | | | | | | | | | | | | | | | | | | | | | | | | | | | |
| 881 | 218231\_at | NAGK | -1.815645694 | 9.485451049 | -6.56775428 | 2.07E-006 | 4.12E-005 | N-acetylglucosamine kinase /// N-acetylglucosamine kinase | N-acetylglucosamine metabolism, N-acetylmannosamine metabolism |  | | | | | | | | | | | | | | | | | | | | | | | | | | | |
| 882 | 218702\_at | SARS2 | 0.86030778 | 6.995159205 | 6.56270108 | 2.09E-006 | 4.16E-005 | seryl-tRNA synthetase 2 | protein biosynthesis, seryl-tRNA aminoacylation |  | | | | | | | | | | | | | | | | | | | | | | | | | | | |
| 883 | 203836\_s\_at | MAP3K5 | -1.103349892 | 7.074636998 | -6.56247916 | 2.09E-006 | 4.16E-005 | mitogen-activated protein kinase kinase kinase 5 | MAPKKK cascade, activation of JUNK, apoptosis, induction of apoptosis by extracellular signals, protein amino acid phosphorylation, response to stress |  | | | | | | | | | | | | | | | | | | | | | | | | | | | |
| 884 | 201923\_at | PRDX4 | 3.402587397 | 7.671123554 | 6.56026018 | 2.10E-006 | 4.17E-005 | peroxiredoxin 4 | I-kappaB phosphorylation |  | | | | | | | | | | | | | | | | | | | | | | | | | | | |
| 885 | 213129\_s\_at | GCSH | 3.192657559 | 7.376397122 | 6.55841623 | 2.11E-006 | 4.18E-005 | glycine cleavage system protein H (aminomethyl carrier) | glycine catabolism |  | | | | | | | | | | | | | | | | | | | | | | | | | | | |
| 886 | 219434\_at | TREM1 | -2.18677183 | 8.485381617 | -6.55776188 | 2.11E-006 | 4.19E-005 | triggering receptor expressed on myeloid cells 1 | humoral immune response, intracellular signaling cascade |  | | | | | | | | | | | | | | | | | | | | | | | | | | | |
| 887 | 213239\_at | C13orf24 | 1.798227684 | 5.819733129 | 6.55431869 | 2.13E-006 | 4.21E-005 | chromosome 13 open reading frame 24 | NA |  | | | | | | | | | | | | | | | | | | | | | | | | | | | |
| 888 | 200031\_s\_at | RPS11 | 0.58936723 | 12.86187437 | 6.55036327 | 2.15E-006 | 4.24E-005 | ribosomal protein S11 /// ribosomal protein S11 | protein biosynthesis |  | | | | | | | | | | | | | | | | | | | | | | | | | | | |
| 889 | 201388\_at | PSMD3 | 1.406232783 | 8.168616405 | 6.54896327 | 2.15E-006 | 4.25E-005 | proteasome (prosome, macropain) 26S subunit, non-ATPase, 3 | NA |  | | | | | | | | | | | | | | | | | | | | | | | | | | | |
| 890 | 203615\_x\_at | SULT1A1 | -1.777750625 | 8.873746415 | -6.54883804 | 2.16E-006 | 4.25E-005 | sulfotransferase family, cytosolic, 1A, phenol-preferring, member 1 | amine metabolism, catecholamine metabolism, steroid metabolism |  | | | | | | | | | | | | | | | | | | | | | | | | | | | |
| 891 | 206102\_at | KIAA0186 | 1.835923876 | 6.780901596 | 6.5481723 | 2.16E-006 | 4.25E-005 | KIAA0186 gene product | NA |  | | | | | | | | | | | | | | | | | | | | | | | | | | | |
| 892 | 211249\_at | GPR68 | 0.568055802 | 5.223232657 | 6.54662928 | 2.17E-006 | 4.26E-005 | G protein-coupled receptor 68 | G-protein coupled receptor protein signaling pathway, inflammatory response |  | | | | | | | | | | | | | | | | | | | | | | | | | | | |
| 893 | 203410\_at | AP3M2 | 0.477328877 | 7.146906552 | 6.5437619 | 2.18E-006 | 4.28E-005 | adaptor-related protein complex 3, mu 2 subunit | intracellular protein transport |  | | | | | | | | | | | | | | | | | | | | | | | | | | | |
| 894 | 202740\_at | ACY1 | 1.061882416 | 8.017711807 | 6.53755819 | 2.21E-006 | 4.33E-005 | aminoacylase 1 | amino acid metabolism, proteolysis and peptidolysis |  | | | | | | | | | | | | | | | | | | | | | | | | | | | |
| 895 | 219531\_at | Cep72 | 0.701152078 | 6.929732302 | 6.53616023 | 2.21E-006 | 4.34E-005 | centrosomal protein 72 kDa | NA |  | | | | | | | | | | | | | | | | | | | | | | | | | | | |
| 896 | 219433\_at | BCOR | 0.898394663 | 5.032729779 | 6.53454582 | 2.22E-006 | 4.35E-005 | BCL6 co-repressor | NA |  | | | | | | | | | | | | | | | | | | | | | | | | | | | |
| 897 | 221816\_s\_at | PHF11 | -1.208841104 | 8.775482486 | -6.53448911 | 2.22E-006 | 4.35E-005 | PHD finger protein 11 | proteolysis and peptidolysis, regulation of transcription, DNA-dependent |  | | | | | | | | | | | | | | | | | | | | | | | | | | | |
| 898 | 203503\_s\_at | PEX14 | 1.251717051 | 7.332047574 | 6.53177387 | 2.24E-006 | 4.37E-005 | peroxisomal biogenesis factor 14 | NA |  | | | | | | | | | | | | | | | | | | | | | | | | | | | |
| 899 | 200792\_at | G22P1 | 1.155477411 | 10.79536188 | 6.52923214 | 2.25E-006 | 4.39E-005 | thyroid autoantigen 70kDa (Ku antigen) | DNA ligation, DNA recombination, DNA repair, double-strand break repair via nonhomologous end-joining |  | | | | | | | | | | | | | | | | | | | | | | | | | | | |
| 900 | 203294\_s\_at | LMAN1 | 1.312588961 | 5.916644162 | 6.5279254 | 2.25E-006 | 4.39E-005 | lectin, mannose-binding, 1 | ER to Golgi transport, blood coagulation, protein folding, protein transport |  | | | | | | | | | | | | | | | | | | | | | | | | | | | |
| 901 | 218830\_at | RPL26L1 | 1.124035602 | 8.462892335 | 6.52763829 | 2.26E-006 | 4.39E-005 | ribosomal protein L26-like 1 | protein biosynthesis |  | | | | | | | | | | | | | | | | | | | | | | | | | | | |
| 902 | 218112\_at | MRPS34 | 1.026562217 | 7.969582807 | 6.52762511 | 2.26E-006 | 4.39E-005 | mitochondrial ribosomal protein S34 | NA |  | | | | | | | | | | | | | | | | | | | | | | | | | | | |
| 903 | 218695\_at | EXOSC4 | 0.87717831 | 8.237687446 | 6.52489336 | 2.27E-006 | 4.41E-005 | exosome component 4 | rRNA processing |  | | | | | | | | | | | | | | | | | | | | | | | | | | | |
| 904 | 218747\_s\_at | TAPBPL | -1.233860527 | 6.956501689 | -6.52080501 | 2.29E-006 | 4.44E-005 | TAP binding protein-like | NA |  | | | | | | | | | | | | | | | | | | | | | | | | | | | |
| 905 | 212048\_s\_at | YARS | 1.961227409 | 9.783942929 | 6.51438201 | 2.32E-006 | 4.50E-005 | tyrosyl-tRNA synthetase | apoptosis, cell motility, protein biosynthesis, tyrosyl-tRNA aminoacylation |  | | | | | | | | | | | | | | | | | | | | | | | | | | | |
| 906 | 212864\_at | CDS2 | -0.808539621 | 7.390762598 | -6.51360277 | 2.32E-006 | 4.50E-005 | CDP-diacylglycerol synthase (phosphatidate cytidylyltransferase) 2 | phospholipid biosynthesis, transcription |  | | | | | | | | | | | | | | | | | | | | | | | | | | | |
| 907 | 200687\_s\_at | SF3B3 | 1.082665572 | 8.503779888 | 6.50895857 | 2.35E-006 | 4.54E-005 | splicing factor 3b, subunit 3, 130kDa | RNA splicing, nuclear mRNA splicing, via spliceosome, protein complex assembly |  | | | | | | | | | | | | | | | | | | | | | | | | | | | |
| 908 | 218333\_at | DERL2 | -0.975695286 | 7.86611577 | -6.50765074 | 2.35E-006 | 4.55E-005 | Der1-like domain family, member 2 | NA |  | | | | | | | | | | | | | | | | | | | | | | | | | | | |
| 909 | 221699\_s\_at | DDX50 | 1.332322942 | 9.046632361 | 6.50678951 | 2.36E-006 | 4.55E-005 | DEAD (Asp-Glu-Ala-Asp) box polypeptide 50 /// DEAD (Asp-Glu-Ala-Asp) box polypeptide 50 | NA |  | | | | | | | | | | | | | | | | | | | | | | | | | | | |
| 910 | 221214\_s\_at | NELF | 0.977102617 | 7.512970041 | 6.50530786 | 2.37E-006 | 4.56E-005 | nasal embryonic LHRH factor | NA |  | | | | | | | | | | | | | | | | | | | | | | | | | | | |
| 911 | 219146\_at | FLJ22729 | 0.95364085 | 5.757241926 | 6.50287802 | 2.38E-006 | 4.58E-005 | hypothetical protein FLJ22729 | NA |  | | | | | | | | | | | | | | | | | | | | | | | | | | | |
| 912 | 218766\_s\_at | WARS2 | 1.25811091 | 6.304642932 | 6.50156479 | 2.38E-006 | 4.59E-005 | tryptophanyl tRNA synthetase 2 (mitochondrial) | protein biosynthesis, tryptophanyl-tRNA aminoacylation |  | | | | | | | | | | | | | | | | | | | | | | | | | | | |
| 913 | 200806\_s\_at | HSPD1 | 2.258789784 | 10.80517332 | 6.50144891 | 2.38E-006 | 4.59E-005 | heat shock 60kDa protein 1 (chaperonin) | mitochondrial matrix protein import, protein folding, response to unfolded protein |  | | | | | | | | | | | | | | | | | | | | | | | | | | | |
| 914 | 200823\_x\_at | RPL29 | 1.16393408 | 12.36990135 | 6.4973461 | 2.41E-006 | 4.62E-005 | ribosomal protein L29 | embryo implantation, protein biosynthesis, small GTPase mediated signal transduction |  | | | | | | | | | | | | | | | | | | | | | | | | | | | |
| 915 | 221598\_s\_at | CRSP8 | 0.477021443 | 6.590459245 | 6.49672926 | 2.41E-006 | 4.63E-005 | cofactor required for Sp1 transcriptional activation, subunit 8, 34kDa | regulation of transcription from Pol II promoter, transcription initiation from Pol II promoter |  | | | | | | | | | | | | | | | | | | | | | | | | | | | |
| 916 | 213011\_s\_at | TPI1 | 0.868820998 | 10.85207289 | 6.4958209 | 2.41E-006 | 4.63E-005 | triosephosphate isomerase 1 | fatty acid biosynthesis, gluconeogenesis, glycolysis, metabolism, pentose-phosphate shunt |  | | | | | | | | | | | | | | | | | | | | | | | | | | | |
| 917 | 64900\_at | MGC15429 | 0.503516999 | 4.45459197 | 6.4953672 | 2.42E-006 | 4.63E-005 | hypothetical protein MGC15429 | NA |  | | | | | | | | | | | | | | | | | | | | | | | | | | | |
| 918 | 201070\_x\_at | SF3B1 | -0.831169787 | 10.13547906 | -6.4891422 | 2.45E-006 | 4.69E-005 | splicing factor 3b, subunit 1, 155kDa | nuclear mRNA splicing, via spliceosome |  | | | | | | | | | | | | | | | | | | | | | | | | | | | |
| 919 | 218460\_at | FLJ20397 | 1.129491633 | 8.282625653 | 6.48815082 | 2.45E-006 | 4.69E-005 | hypothetical protein FLJ20397 | NA |  | | | | | | | | | | | | | | | | | | | | | | | | | | | |
| 920 | 212997\_s\_at | TLK2 | 0.658164825 | 7.872636119 | 6.48384507 | 2.48E-006 | 4.73E-005 | tousled-like kinase 2 | cell cycle, chromatin modification, intracellular signaling cascade, protein amino acid phosphorylation, regulation of chromatin assembly or disassembly, response to DNA damage stimulus |  | | | | | | | | | | | | | | | | | | | | | | | | | | | |
| 921 | 214383\_x\_at | KLHDC3 | 0.739280832 | 8.755438746 | 6.48145231 | 2.49E-006 | 4.75E-005 | kelch domain containing 3 | meiotic recombination |  | | | | | | | | | | | | | | | | | | | | | | | | | | | |
| 922 | 201119\_s\_at | COX8A | 0.853557613 | 10.85811601 | 6.48069674 | 2.49E-006 | 4.75E-005 | cytochrome c oxidase subunit 8A (ubiquitous) | electron transport, energy pathways |  | | | | | | | | | | | | | | | | | | | | | | | | | | | |
| 923 | 219030\_at | CGI-121 | 1.207166336 | 8.211850901 | 6.47978929 | 2.50E-006 | 4.76E-005 | CGI-121 protein | protein catabolism |  | | | | | | | | | | | | | | | | | | | | | | | | | | | |
| 924 | 212214\_at | OPA1 | 0.797513034 | 7.166334971 | 6.47642764 | 2.52E-006 | 4.79E-005 | optic atrophy 1 (autosomal dominant) | axon transport of mitochondrion, inner mitochondrial membrane organization and biogenesis, mitochondrial fission, mitochondrial fusion, positive regulation of anti-apoptosis, visual perception |  | | | | | | | | | | | | | | | | | | | | | | | | | | | |
| 925 | 218047\_at | OSBPL9 | 1.144326314 | 9.028592579 | 6.47341716 | 2.53E-006 | 4.81E-005 | oxysterol binding protein-like 9 | lipid transport, steroid metabolism |  | | | | | | | | | | | | | | | | | | | | | | | | | | | |
| 926 | 1729\_at | TRADD | -0.588922229 | 7.662764999 | -6.47272828 | 2.54E-006 | 4.82E-005 | TNFRSF1A-associated via death domain | apoptosis, induction of apoptosis, positive regulation of I-kappaB kinase/NF-kappaB cascade, signal transduction |  | | | | | | | | | | | | | | | | | | | | | | | | | | | |
| 927 | 208897\_s\_at | DDX18 | 0.934731411 | 9.048916156 | 6.47100412 | 2.55E-006 | 4.83E-005 | DEAD (Asp-Glu-Ala-Asp) box polypeptide 18 | NA |  | | | | | | | | | | | | | | | | | | | | | | | | | | | |
| 928 | 215416\_s\_at | STOML2 | 2.108397389 | 8.557513766 | 6.46997399 | 2.55E-006 | 4.84E-005 | stomatin (EPB72)-like 2 | NA |  | | | | | | | | | | | | | | | | | | | | | | | | | | | |
| 929 | 204459\_at | CSTF2 | 0.787577085 | 7.241454453 | 6.4691147 | 2.56E-006 | 4.84E-005 | cleavage stimulation factor, 3' pre-RNA, subunit 2, 64kDa | mRNA cleavage, mRNA polyadenylylation |  | | | | | | | | | | | | | | | | | | | | | | | | | | | |
| 930 | 220052\_s\_at | TINF2 | -0.781239102 | 7.762696291 | -6.46794749 | 2.56E-006 | 4.84E-005 | TERF1 (TRF1)-interacting nuclear factor 2 | telomerase-dependent telomere maintenance |  | | | | | | | | | | | | | | | | | | | | | | | | | | | |
| 931 | 212702\_s\_at | BICD2 | -0.980069913 | 8.038087126 | -6.46171581 | 2.60E-006 | 4.90E-005 | bicaudal D homolog 2 (Drosophila) | NA |  | | | | | | | | | | | | | | | | | | | | | | | | | | | |
| 932 | 221488\_s\_at | C6orf82 | 1.34080458 | 10.14828527 | 6.45915654 | 2.61E-006 | 4.92E-005 | chromosome 6 open reading frame 82 | NA |  | | | | | | | | | | | | | | | | | | | | | | | | | | | |
| 933 | 202567\_at | SNRPD3 | 1.027092491 | 9.46461352 | 6.45834912 | 2.62E-006 | 4.92E-005 | small nuclear ribonucleoprotein D3 polypeptide 18kDa | nuclear mRNA splicing, via spliceosome |  | | | | | | | | | | | | | | | | | | | | | | | | | | | |
| 934 | 218884\_s\_at | FLJ13220 | 0.748685935 | 5.115469936 | 6.45515063 | 2.63E-006 | 4.95E-005 | hypothetical protein FLJ13220 | protein biosynthesis, translational elongation |  | | | | | | | | | | | | | | | | | | | | | | | | | | | |
| 935 | 212482\_at | FLJ13910 | 0.963015204 | 7.649858022 | 6.45422637 | 2.64E-006 | 4.96E-005 | hypothetical protein FLJ13910 | NA |  | | | | | | | | | | | | | | | | | | | | | | | | | | | |
| 936 | 201763\_s\_at | DAXX | 0.491516767 | 8.369766275 | 6.45143631 | 2.65E-006 | 4.98E-005 | death-associated protein 6 | apoptosis, regulation of transcription, DNA-dependent |  | | | | | | | | | | | | | | | | | | | | | | | | | | | |
| 937 | 213152\_s\_at | SRP46 | 1.33267762 | 6.479976086 | 6.45104358 | 2.66E-006 | 4.98E-005 | Splicing factor, arginine/serine-rich, 46kD | NA |  | | | | | | | | | | | | | | | | | | | | | | | | | | | |
| 938 | 209511\_at | POLR2F | 1.010609814 | 7.939864408 | 6.44847628 | 2.67E-006 | 5.00E-005 | polymerase (RNA) II (DNA directed) polypeptide F | transcription, DNA-dependent |  | | | | | | | | | | | | | | | | | | | | | | | | | | | |
| 939 | 212893\_at | ZZZ3 | 1.15263002 | 6.858433826 | 6.44803578 | 2.67E-006 | 5.00E-005 | zinc finger, ZZ domain containing 3 | NA |  | | | | | | | | | | | | | | | | | | | | | | | | | | | |
| 940 | 205361\_s\_at | PFDN4 | 2.049917677 | 7.744532845 | 6.44745444 | 2.68E-006 | 5.01E-005 | prefoldin 4 | chaperonin-mediated tubulin folding, protein folding |  | | | | | | | | | | | | | | | | | | | | | | | | | | | |
| 941 | 221531\_at | REC14 | 1.320926068 | 7.619115699 | 6.44621021 | 2.68E-006 | 5.02E-005 | recombination protein REC14 | NA |  | | | | | | | | | | | | | | | | | | | | | | | | | | | |
| 942 | 201948\_at | GNL2 | 1.874867664 | 6.939457827 | 6.44433476 | 2.70E-006 | 5.03E-005 | guanine nucleotide binding protein-like 2 (nucleolar) | ribosome biogenesis |  | | | | | | | | | | | | | | | | | | | | | | | | | | | |
| 943 | 203594\_at | RTCD1 | 2.155304898 | 8.106163733 | 6.44200252 | 2.71E-006 | 5.05E-005 | RNA terminal phosphate cyclase domain 1 | assembly of spliceosomal tri-snRNP |  | | | | | | | | | | | | | | | | | | | | | | | | | | | |
| 944 | 202416\_at | DNAJC7 | 0.83159111 | 8.705384024 | 6.44160952 | 2.71E-006 | 5.05E-005 | DnaJ (Hsp40) homolog, subfamily C, member 7 | protein folding |  | | | | | | | | | | | | | | | | | | | | | | | | | | | |
| 945 | 58780\_s\_at | FLJ10357 | -1.608479735 | 7.229838581 | -6.4388674 | 2.73E-006 | 5.07E-005 | hypothetical protein FLJ10357 | NA |  | | | | | | | | | | | | | | | | | | | | | | | | | | | |
| 946 | 202325\_s\_at | ATP5J | 1.136718142 | 9.728171424 | 6.43697272 | 2.74E-006 | 5.09E-005 | ATP synthase, H+ transporting, mitochondrial F0 complex, subunit F6 | ATP synthesis coupled proton transport, energy pathways, proton transport |  | | | | | | | | | | | | | | | | | | | | | | | | | | | |
| 947 | 202188\_at | NUP93 | 0.906498908 | 8.462442838 | 6.43656868 | 2.74E-006 | 5.09E-005 | nucleoporin 93kDa | transport |  | | | | | | | | | | | | | | | | | | | | | | | | | | | |
| 948 | 201236\_s\_at | BTG2 | -2.192658694 | 9.074324589 | -6.43261655 | 2.76E-006 | 5.13E-005 | BTG family, member 2 | DNA repair, negative regulation of cell proliferation, regulation of transcription, DNA-dependent |  | | | | | | | | | | | | | | | | | | | | | | | | | | | |
| 949 | 203103\_s\_at | PRP19 | 1.312467202 | 9.361492579 | 6.43039133 | 2.78E-006 | 5.15E-005 | PRP19/PSO4 homolog (S. cerevisiae) | protein ubiquitination |  | | | | | | | | | | | | | | | | | | | | | | | | | | | |
| 950 | 218627\_at | FLJ11259 | -1.571022858 | 7.35445784 | -6.42742963 | 2.80E-006 | 5.18E-005 | hypothetical protein FLJ11259 | NA |  | | | | | | | | | | | | | | | | | | | | | | | | | | | |
| 951 | 213784\_at | RABL4 | -0.458718474 | 7.191134401 | -6.4263474 | 2.80E-006 | 5.19E-005 | RAB, member of RAS oncogene family-like 4 | protein transport, small GTPase mediated signal transduction |  | | | | | | | | | | | | | | | | | | | | | | | | | | | |
| 952 | 207397\_s\_at | HOXD13 | 2.38643895 | 4.499755862 | 6.42422886 | 2.81E-006 | 5.20E-005 | homeo box D13 | development, regulation of transcription, DNA-dependent, skeletal development, transcription from Pol II promoter |  | | | | | | | | | | | | | | | | | | | | | | | | | | | |
| 953 | 213956\_at | CAP350 | -0.863580416 | 8.033931437 | -6.42347504 | 2.82E-006 | 5.20E-005 | centrosome-associated protein 350 | NA |  | | | | | | | | | | | | | | | | | | | | | | | | | | | |
| 954 | 218190\_s\_at | HSPC051 | 0.762921507 | 10.11385551 | 6.42271618 | 2.82E-006 | 5.20E-005 | ubiquinol-cytochrome c reductase complex (7.2 kD) | electron transport, mitochondrial electron transport, ubiquinol to cytochrome c |  | | | | | | | | | | | | | | | | | | | | | | | | | | | |
| 955 | 205909\_at | POLE2 | 1.220436592 | 7.481303316 | 6.41388047 | 2.88E-006 | 5.30E-005 | polymerase (DNA directed), epsilon 2 (p59 subunit) | DNA repair, DNA replication |  | | | | | | | | | | | | | | | | | | | | | | | | | | | |
| 956 | 209084\_s\_at | RAB28 | 1.140724395 | 5.928436042 | 6.41340967 | 2.88E-006 | 5.30E-005 | RAB28, member RAS oncogene family | small GTPase mediated signal transduction |  | | | | | | | | | | | | | | | | | | | | | | | | | | | |
| 957 | 202406\_s\_at | TIAL1 | 0.955186622 | 9.00191092 | 6.41080616 | 2.90E-006 | 5.32E-005 | TIA1 cytotoxic granule-associated RNA binding protein-like 1 | apoptosis, defense response, induction of apoptosis, regulation of transcription from Pol II promoter |  | | | | | | | | | | | | | | | | | | | | | | | | | | | |
| 958 | 203344\_s\_at | RBBP8 | 1.67987384 | 7.845947465 | 6.4106437 | 2.90E-006 | 5.32E-005 | retinoblastoma binding protein 8 | NA |  | | | | | | | | | | | | | | | | | | | | | | | | | | | |
| 959 | 211796\_s\_at | TRB@ | -2.300008253 | 8.086129924 | -6.4093794 | 2.91E-006 | 5.33E-005 | T cell receptor beta locus | immune response |  | | | | | | | | | | | | | | | | | | | | | | | | | | | |
| 960 | 203305\_at | F13A1 | -1.80931821 | 7.66940631 | -6.40850962 | 2.91E-006 | 5.33E-005 | coagulation factor XIII, A1 polypeptide | blood coagulation, peptide cross-linking |  | | | | | | | | | | | | | | | | | | | | | | | | | | | |
| 961 | 218936\_s\_at | HSPC128 | 1.420039769 | 7.051621409 | 6.40840218 | 2.91E-006 | 5.33E-005 | HSPC128 protein | NA |  | | | | | | | | | | | | | | | | | | | | | | | | | | | |
| 962 | 203577\_at | GTF2H4 | 1.24088574 | 6.787144906 | 6.40737342 | 2.92E-006 | 5.34E-005 | general transcription factor IIH, polypeptide 4, 52kDa | DNA repair, regulation of transcription, DNA-dependent, transcription from Pol II promoter |  | | | | | | | | | | | | | | | | | | | | | | | | | | | |
| 963 | 201231\_s\_at | ENO1 | 0.935430039 | 11.64210344 | 6.40422344 | 2.94E-006 | 5.36E-005 | enolase 1, (alpha) | glycolysis, negative regulation of cell growth, negative regulation of transcription from Pol II promoter |  | | | | | | | | | | | | | | | | | | | | | | | | | | | |
| 964 | 217915\_s\_at | C15orf15 | 1.658883188 | 9.888266553 | 6.40329047 | 2.94E-006 | 5.37E-005 | chromosome 15 open reading frame 15 | protein biosynthesis, ribosome biogenesis |  | | | | | | | | | | | | | | | | | | | | | | | | | | | |
| 965 | 217987\_at | NS3TP1 | 0.948517758 | 7.683977483 | 6.40051711 | 2.96E-006 | 5.40E-005 | HCV NS3-transactivated protein 1 | asparagine biosynthesis |  | | | | | | | | | | | | | | | | | | | | | | | | | | | |
| 966 | 221622\_s\_at | HT007 | 1.669053677 | 8.860006068 | 6.39817001 | 2.98E-006 | 5.42E-005 | uncharacterized hypothalamus protein HT007 | NA |  | | | | | | | | | | | | | | | | | | | | | | | | | | | |
| 967 | 200750\_s\_at | RAN | 2.076255329 | 10.5728108 | 6.39749577 | 2.98E-006 | 5.42E-005 | RAN, member RAS oncogene family | protein transport |  | | | | | | | | | | | | | | | | | | | | | | | | | | | |
| 968 | 201115\_at | POLD2 | 2.081564657 | 8.412653433 | 6.39261974 | 3.01E-006 | 5.47E-005 | polymerase (DNA directed), delta 2, regulatory subunit 50kDa | DNA replication |  | | | | | | | | | | | | | | | | | | | | | | | | | | | |
| 969 | 202431\_s\_at | MYC | 2.551000466 | 9.39851052 | 6.39231176 | 3.01E-006 | 5.47E-005 | v-myc myelocytomatosis viral oncogene homolog (avian) | cell cycle arrest, cell proliferation, iron ion homeostasis, regulation of transcription from Pol II promoter |  | | | | | | | | | | | | | | | | | | | | | | | | | | | |
| 970 | 202754\_at | R3HDM | 1.032555169 | 8.408622156 | 6.39143916 | 3.02E-006 | 5.47E-005 | R3H domain (binds single-stranded nucleic acids) containing | NA |  | | | | | | | | | | | | | | | | | | | | | | | | | | | |
| 971 | 205217\_at | TIMM8A | 0.766500341 | 5.063733556 | 6.39098172 | 3.02E-006 | 5.47E-005 | translocase of inner mitochondrial membrane 8 homolog A (yeast) | mitochondrial inner membrane protein import, neurogenesis, perception of sound, protein transport |  | | | | | | | | | | | | | | | | | | | | | | | | | | | |
| 972 | 209906\_at | C3AR1 | -2.233062724 | 9.217496866 | -6.39036259 | 3.03E-006 | 5.47E-005 | complement component 3a receptor 1 | G-protein coupled receptor protein signaling pathway, cell motility, cellular defense response, chemotaxis, circulation, cytosolic calcium ion concentration elevation, inflammatory response, smooth muscle contraction |  | | | | | | | | | | | | | | | | | | | | | | | | | | | |
| 973 | 208905\_at | CYCS | 2.053163556 | 10.54231387 | 6.39028752 | 3.03E-006 | 5.47E-005 | cytochrome c, somatic | DNA fragmentation during apoptosis, apoptosis, caspase activation via cytochrome c, cellular respiration, electron transport |  | | | | | | | | | | | | | | | | | | | | | | | | | | | |
| 974 | 200845\_s\_at | PRDX6 | 1.296455453 | 9.044054764 | 6.38962642 | 3.03E-006 | 5.48E-005 | peroxiredoxin 6 | lipid catabolism, phospholipid catabolism, response to oxidative stress |  | | | | | | | | | | | | | | | | | | | | | | | | | | | |
| 975 | 217353\_at | HNRPA1 | 0.618923734 | 4.782194147 | 6.38871664 | 3.04E-006 | 5.48E-005 | heterogeneous nuclear ribonucleoprotein A1 | mRNA processing, mRNA-nucleus export, transport |  | | | | | | | | | | | | | | | | | | | | | | | | | | | |
| 976 | 214752\_x\_at | FLNA | -1.128944163 | 9.999959787 | -6.38658652 | 3.05E-006 | 5.49E-005 | filamin A, alpha (actin binding protein 280) | actin cytoskeleton organization and biogenesis, cell motility, cell surface receptor linked signal transduction, neurogenesis, positive regulation of I-kappaB kinase/NF-kappaB cascade |  | | | | | | | | | | | | | | | | | | | | | | | | | | | |
| 977 | 203318\_s\_at | ZNF148 | -0.7209137 | 7.960832069 | -6.38654317 | 3.05E-006 | 5.49E-005 | zinc finger protein 148 (pHZ-52) | cellular defense response, negative regulation of transcription from Pol II promoter, regulation of transcription, DNA-dependent |  | | | | | | | | | | | | | | | | | | | | | | | | | | | |
| 978 | 209124\_at | MYD88 | -1.77635997 | 10.03276759 | -6.38635463 | 3.05E-006 | 5.49E-005 | myeloid differentiation primary response gene (88) | cell surface receptor linked signal transduction, immune response, inflammatory response, positive regulation of I-kappaB kinase/NF-kappaB cascade |  | | | | | | | | | | | | | | | | | | | | | | | | | | | |
| 979 | 205446\_s\_at | ATF2 | 0.910309227 | 7.156601389 | 6.38564414 | 3.06E-006 | 5.50E-005 | activating transcription factor 2 | regulation of transcription, DNA-dependent |  | | | | | | | | | | | | | | | | | | | | | | | | | | | |
| 980 | 202110\_at | COX7B | 1.174358056 | 9.911425598 | 6.38256111 | 3.08E-006 | 5.52E-005 | cytochrome c oxidase subunit VIIb | electron transport |  | | | | | | | | | | | | | | | | | | | | | | | | | | | |
| 981 | 217640\_x\_at | C18orf24 | 1.828392026 | 5.04876242 | 6.38010938 | 3.09E-006 | 5.55E-005 | chromosome 18 open reading frame 24 | NA |  | | | | | | | | | | | | | | | | | | | | | | | | | | | |
| 982 | 203622\_s\_at | LOC56902 | 1.24685973 | 7.953850192 | 6.37548722 | 3.13E-006 | 5.60E-005 | putatative 28 kDa protein | NA |  | | | | | | | | | | | | | | | | | | | | | | | | | | | |
| 983 | 209645\_s\_at | ALDH1B1 | 0.728438402 | 6.76680827 | 6.37546164 | 3.13E-006 | 5.60E-005 | aldehyde dehydrogenase 1 family, member B1 | carbohydrate metabolism, metabolism |  | | | | | | | | | | | | | | | | | | | | | | | | | | | |
| 984 | 218389\_s\_at | APH-1A | 0.620721524 | 8.357613294 | 6.37408513 | 3.14E-006 | 5.61E-005 | likely ortholog of C. elegans anterior pharynx defective 1A | Notch receptor processing, amyloid precursor protein catabolism, membrane protein ectodomain proteolysis, positive regulation of enzyme activity, protein processing |  | | | | | | | | | | | | | | | | | | | | | | | | | | | |
| 985 | 218398\_at | MRPS30 | 1.853745911 | 8.429598765 | 6.36698979 | 3.18E-006 | 5.68E-005 | mitochondrial ribosomal protein S30 | apoptosis, electron transport, protein biosynthesis |  | | | | | | | | | | | | | | | | | | | | | | | | | | | |
| 986 | 212256\_at | GALNT10 | -0.895624174 | 7.17418593 | -6.36654472 | 3.19E-006 | 5.68E-005 | UDP-N-acetyl-alpha-D-galactosamine:polypeptide N-acetylgalactosaminyltransferase 10 (GalNAc-T10) | NA |  | | | | | | | | | | | | | | | | | | | | | | | | | | | |
| 987 | 204023\_at | RFC4 | 1.674575514 | 8.179342683 | 6.36507874 | 3.20E-006 | 5.68E-005 | replication factor C (activator 1) 4, 37kDa | DNA replication, DNA strand elongation |  | | | | | | | | | | | | | | | | | | | | | | | | | | | |
| 988 | 218443\_s\_at | DAZAP1 | 1.044176534 | 7.381802545 | 6.36195443 | 3.22E-006 | 5.71E-005 | DAZ associated protein 1 | spermatogenesis |  | | | | | | | | | | | | | | | | | | | | | | | | | | | |
| 989 | 216241\_s\_at | TCEA1 | 1.601797427 | 9.573968413 | 6.36163034 | 3.22E-006 | 5.71E-005 | transcription elongation factor A (SII), 1 | RNA elongation, regulation of transcription from Pol II promoter, transcription |  | | | | | | | | | | | | | | | | | | | | | | | | | | | |
| 990 | 217025\_s\_at | DBN1 | 1.045042884 | 7.606020678 | 6.36120658 | 3.22E-006 | 5.72E-005 | drebrin 1 | actin filament organization, neurogenesis, regulation of dendrite morphogenesis, regulation of neuronal synaptic plasticity |  | | | | | | | | | | | | | | | | | | | | | | | | | | | |
| 991 | 200025\_s\_at | RPL27 | 0.556850861 | 12.64311337 | 6.35986133 | 3.23E-006 | 5.73E-005 | ribosomal protein L27 /// ribosomal protein L27 | protein biosynthesis |  | | | | | | | | | | | | | | | | | | | | | | | | | | | |
| 992 | 218580\_x\_at | AKIP | 0.915578463 | 9.607544971 | 6.35783711 | 3.25E-006 | 5.74E-005 | aurora-A kinase interacting protein | NA |  | | | | | | | | | | | | | | | | | | | | | | | | | | | |
| 993 | 205115\_s\_at | RBM19 | 0.619468587 | 6.840805563 | 6.35696047 | 3.25E-006 | 5.75E-005 | RNA binding motif protein 19 | NA |  | | | | | | | | | | | | | | | | | | | | | | | | | | | |
| 994 | 200843\_s\_at | EPRS | 1.64448011 | 9.004247673 | 6.35677949 | 3.25E-006 | 5.75E-005 | glutamyl-prolyl-tRNA synthetase | glutamyl-tRNA aminoacylation, prolyl-tRNA aminoacylation, protein biosynthesis, protein complex assembly |  | | | | | | | | | | | | | | | | | | | | | | | | | | | |
| 995 | 37226\_at | BNIP1 | 0.765936783 | 6.244845405 | 6.35466187 | 3.27E-006 | 5.76E-005 | BCL2/adenovirus E1B 19kDa interacting protein 1 | anti-apoptosis, apoptosis |  | | | | | | | | | | | | | | | | | | | | | | | | | | | |
| 996 | 219530\_at | FLJ21816 | 0.75794513 | 6.129513747 | 6.35461267 | 3.27E-006 | 5.76E-005 | hypothetical protein FLJ21816 | NA |  | | | | | | | | | | | | | | | | | | | | | | | | | | | |
| 997 | 217526\_at | FLJ14639 | -1.254273081 | 7.266222863 | -6.35412308 | 3.27E-006 | 5.77E-005 | nuclear factor of activated T-cells, cytoplasmic, calcineurin-dependent 2 interacting protein | NA |  | | | | | | | | | | | | | | | | | | | | | | | | | | | |
| 998 | 202527\_s\_at | SMAD4 | 1.248852699 | 6.486623549 | 6.35097655 | 3.30E-006 | 5.80E-005 | SMAD, mothers against DPP homolog 4 (Drosophila) | SMAD protein heteromerization, regulation of transcription, DNA-dependent |  | | | | | | | | | | | | | | | | | | | | | | | | | | | |
| 999 | 219122\_s\_at | ICF45 | 1.142163506 | 7.48670895 | 6.34895766 | 3.31E-006 | 5.82E-005 | interphase cyctoplasmic foci protein 45 | NA |  | | | | | | | | | | | | | | | | | | | | | | | | | | | |
| 1000 | 219762\_s\_at | RPL36 | 1.835445548 | 10.69219826 | 6.34776298 | 3.32E-006 | 5.83E-005 | ribosomal protein L36 | protein biosynthesis |  | | | | | | | | | | | | | | | | | | | | | | | | | | | |
| 1001 | 219933\_at | GLRX2 | 1.05048956 | 7.614963303 | 6.3364383 | 3.40E-006 | 5.95E-005 | glutaredoxin 2 | DNA protection, apoptosis, cell differentiation, electron transport, glutathione metabolism, protein thiol-disulfide exchange, redox signal response, regulation of cell redox homeostasis, regulation of signal transduction, regulation of transcription, response to hydrogen peroxide, response to organic substance, response to pH, response to temperature | | | |  | | | | | | | | | | | | | | | | | | | | | | | | |
| 1002 | 201274\_at | PSMA5 | 1.207479335 | 9.034124166 | 6.33447619 | 3.42E-006 | 5.97E-005 | proteasome (prosome, macropain) subunit, alpha type, 5 | ubiquitin-dependent protein catabolism |  | | | | | | | | | | | | | | | | | | | | | | | | | | | |
| 1003 | 200877\_at | CCT4 | 1.936821024 | 10.97990751 | 6.33353578 | 3.42E-006 | 5.98E-005 | chaperonin containing TCP1, subunit 4 (delta) | protein folding, regulation of cell cycle |  | | | | | | | | | | | | | | | | | | | | | | | | | | | |
| 1004 | 212108\_at | ETEA | 0.575551784 | 7.991718672 | 6.33189877 | 3.43E-006 | 6.00E-005 | expressed in T-cells and eosinophils in atopic dermatitis | NA |  | | | | | | | | | | | | | | | | | | | | | | | | | | | |
| 1005 | 209314\_s\_at | HBS1L | 0.745974478 | 4.607858298 | 6.32999087 | 3.45E-006 | 6.02E-005 | HBS1-like (S. cerevisiae) | protein biosynthesis, signal transduction |  | | | | | | | | | | | | | | | | | | | | | | | | | | | |
| 1006 | 209509\_s\_at | DPAGT1 | 1.166471241 | 7.764996552 | 6.32901297 | 3.46E-006 | 6.02E-005 | dolichyl-phosphate (UDP-N-acetylglucosamine) N-acetylglucosaminephosphotransferase 1 (GlcNAc-1-P transferase) | lipid metabolism |  | | | | | | | | | | | | | | | | | | | | | | | | | | | |
| 1007 | 219166\_at | C14orf104 | 1.314333952 | 5.057370037 | 6.3289196 | 3.46E-006 | 6.02E-005 | chromosome 14 open reading frame 104 | NA |  | | | | | | | | | | | | | | | | | | | | | | | | | | | |
| 1008 | 202941\_at | NDUFV2 | 1.265485081 | 9.493617047 | 6.32829049 | 3.46E-006 | 6.02E-005 | NADH dehydrogenase (ubiquinone) flavoprotein 2, 24kDa | mitochondrial electron transport, NADH to ubiquinone, neurogenesis |  | | | | | | | | | | | | | | | | | | | | | | | | | | | |
| 1009 | 216210\_x\_at | HRIHFB2122 | -1.153254054 | 8.60927023 | -6.32436657 | 3.49E-006 | 6.06E-005 | Tara-like protein | actin modification, barbed-end actin filament capping |  | | | | | | | | | | | | | | | | | | | | | | | | | | | |
| 1010 | 201747\_s\_at | SAFB | 0.759348915 | 6.479099734 | 6.31840602 | 3.54E-006 | 6.13E-005 | scaffold attachment factor B | establishment and/or maintenance of chromatin architecture, regulation of transcription, DNA-dependent |  | | | | | | | | | | | | | | | | | | | | | | | | | | | |
| 1011 | 211998\_at | H3F3B | -2.220077374 | 9.189356675 | -6.31510664 | 3.56E-006 | 6.16E-005 | H3 histone, family 3B (H3.3B) | cell adhesion, chromosome organization and biogenesis (sensu Eukaryota), nucleosome assembly, regulation of cell shape |  | | | | | | | | | | | | | | | | | | | | | | | | | | | |
| 1012 | 202683\_s\_at | RNMT | 1.252874787 | 7.501934369 | 6.31504459 | 3.56E-006 | 6.16E-005 | RNA (guanine-7-) methyltransferase | mRNA capping |  | | | | | | | | | | | | | | | | | | | | | | | | | | | |
| 1013 | 204868\_at | ICT1 | 1.425218663 | 7.529262364 | 6.3149792 | 3.56E-006 | 6.16E-005 | immature colon carcinoma transcript 1 | translational termination |  | | | | | | | | | | | | | | | | | | | | | | | | | | | |
| 1014 | 200989\_at | HIF1A | -1.702100684 | 9.332323275 | -6.31384844 | 3.57E-006 | 6.17E-005 | hypoxia-inducible factor 1, alpha subunit (basic helix-loop-helix transcription factor) | homeostasis, regulation of transcription, DNA-dependent, response to hypoxia, signal transduction |  | | | | | | | | | | | | | | | | | | | | | | | | | | | |
| 1015 | 219275\_at | PDCD5 | 1.084268588 | 6.961881666 | 6.31100833 | 3.59E-006 | 6.19E-005 | programmed cell death 5 | apoptosis, induction of apoptosis |  | | | | | | | | | | | | | | | | | | | | | | | | | | | |
| 1016 | 214322\_at | CAMK2G | -0.896019665 | 7.095919879 | -6.31079644 | 3.59E-006 | 6.19E-005 | calcium/calmodulin-dependent protein kinase (CaM kinase) II gamma | insulin secretion, protein amino acid phosphorylation, signal transduction |  | | | | | | | | | | | | | | | | | | | | | | | | | | | |
| 1017 | 218982\_s\_at | MRPS17 | 1.962359209 | 7.6210676 | 6.30080557 | 3.67E-006 | 6.32E-005 | mitochondrial ribosomal protein S17 | protein biosynthesis |  | | | | | | | | | | | | | | | | | | | | | | | | | | | |
| 1018 | 212420\_at | ELF1 | -1.162985628 | 10.62668758 | -6.30007543 | 3.68E-006 | 6.32E-005 | E74-like factor 1 (ets domain transcription factor) | regulation of transcription, DNA-dependent |  | | | | | | | | | | | | | | | | | | | | | | | | | | | |
| 1019 | 211088\_s\_at | PLK4 | 0.700540919 | 4.478555817 | 6.3000371 | 3.68E-006 | 6.32E-005 | polo-like kinase 4 (Drosophila) /// polo-like kinase 4 (Drosophila) | protein amino acid phosphorylation, regulation of cell cycle |  | | | | | | | | | | | | | | | | | | | | | | | | | | | |
| 1020 | 203574\_at | NFIL3 | -1.842756736 | 9.060182642 | -6.29481852 | 3.72E-006 | 6.38E-005 | nuclear factor, interleukin 3 regulated | immune response, regulation of transcription, DNA-dependent, transcription from Pol II promoter |  | | | | | | | | | | | | | | | | | | | | | | | | | | | |
| 1021 | 216996\_s\_at | KIAA0971 | 1.135898732 | 7.558642622 | 6.29363633 | 3.73E-006 | 6.39E-005 | KIAA0971 | NA |  | | | | | | | | | | | | | | | | | | | | | | | | | | | |
| 1022 | 201091\_s\_at | CBX3 | 1.289788666 | 8.293422739 | 6.28616829 | 3.79E-006 | 6.49E-005 | chromobox homolog 3 (HP1 gamma homolog, Drosophila) | chromatin assembly or disassembly, chromatin modification, regulation of transcription, DNA-dependent |  | | | | | | | | | | | | | | | | | | | | | | | | | | | |
| 1023 | 203186\_s\_at | S100A4 | -3.620675483 | 9.980310615 | -6.28441197 | 3.81E-006 | 6.51E-005 | S100 calcium binding protein A4 (calcium protein, calvasculin, metastasin, murine placental homolog) | NA |  | | | | | | | | | | | | | | | | | | | | | | | | | | | |
| 1024 | 218647\_s\_at | FLJ23476 | 0.957191373 | 7.540960639 | 6.28403268 | 3.81E-006 | 6.51E-005 | ischemia/reperfusion inducible protein | NA |  | | | | | | | | | | | | | | | | | | | | | | | | | | | |
| 1025 | 215068\_s\_at | FBXL18 | 0.613267225 | 5.283598377 | 6.28305084 | 3.82E-006 | 6.52E-005 | F-box and leucine-rich repeat protein 18 | NA |  | | | | | | | | | | | | | | | | | | | | | | | | | | | |
| 1026 | 203613\_s\_at | NDUFB6 | 1.282918778 | 8.707228746 | 6.28284903 | 3.82E-006 | 6.52E-005 | NADH dehydrogenase (ubiquinone) 1 beta subcomplex, 6, 17kDa | mitochondrial electron transport, NADH to ubiquinone |  | | | | | | | | | | | | | | | | | | | | | | | | | | | |
| 1027 | 218130\_at | MGC4368 | -1.309240759 | 7.429196043 | -6.28015213 | 3.84E-006 | 6.55E-005 | hypothetical protein MGC4368 | NA |  | | | | | | | | | | | | | | | | | | | | | | | | | | | |
| 1028 | 203738\_at | FLJ11193 | 1.239491364 | 6.633666141 | 6.27929921 | 3.85E-006 | 6.55E-005 | Hypothetical protein FLJ11193 | NA |  | | | | | | | | | | | | | | | | | | | | | | | | | | | |
| 1029 | 221509\_at | DENR | 1.396468551 | 8.302428297 | 6.27908137 | 3.85E-006 | 6.55E-005 | density-regulated protein | cell growth and/or maintenance, translational initiation |  | | | | | | | | | | | | | | | | | | | | | | | | | | | |
| 1030 | 202799\_at | CLPP | 1.202694945 | 8.337302774 | 6.27893377 | 3.85E-006 | 6.55E-005 | ClpP caseinolytic protease, ATP-dependent, proteolytic subunit homolog (E. coli) | proteolysis and peptidolysis |  | | | | | | | | | | | | | | | | | | | | | | | | | | | |
| 1031 | 207239\_s\_at | PCTK1 | 0.610021969 | 7.091881346 | 6.27624069 | 3.87E-006 | 6.58E-005 | PCTAIRE protein kinase 1 | protein amino acid phosphorylation, regulation of cell cycle |  | | | | | | | | | | | | | | | | | | | | | | | | | | | |
| 1032 | 208249\_s\_at | TGDS | 1.37400655 | 7.058981739 | 6.27595786 | 3.88E-006 | 6.58E-005 | TDP-glucose 4,6-dehydratase | nucleotide-sugar metabolism |  | | | | | | | | | | | | | | | | | | | | | | | | | | | |
| 1033 | 201327\_s\_at | CCT6A | 2.23820419 | 8.406282994 | 6.2755282 | 3.88E-006 | 6.58E-005 | chaperonin containing TCP1, subunit 6A (zeta 1) | protein folding |  | | | | | | | | | | | | | | | | | | | | | | | | | | | |
| 1034 | 210465\_s\_at | SNAPC3 | 1.04240767 | 5.937361405 | 6.2747634 | 3.89E-006 | 6.59E-005 | small nuclear RNA activating complex, polypeptide 3, 50kDa | regulation of transcription, DNA-dependent, snRNA transcription, transcription from Pol II promoter, transcription from Pol III promoter |  | | | | | | | | | | | | | | | | | | | | | | | | | | | |
| 1035 | 206036\_s\_at | REL | -1.401361915 | 6.702300222 | -6.27209849 | 3.91E-006 | 6.62E-005 | v-rel reticuloendotheliosis viral oncogene homolog (avian) | cell growth and/or maintenance, positive regulation of I-kappaB kinase/NF-kappaB cascade, regulation of transcription, DNA-dependent, transcription from Pol II promoter |  | | | | | | | | | | | | | | | | | | | | | | | | | | | |
| 1036 | 209861\_s\_at | METAP2 | 2.230584616 | 8.646953697 | 6.27161288 | 3.91E-006 | 6.62E-005 | methionyl aminopeptidase 2 | protein modification, proteolysis and peptidolysis, regulation of translation |  | | | | | | | | | | | | | | | | | | | | | | | | | | | |
| 1037 | 221081\_s\_at | FLJ22457 | -1.773487334 | 7.109311109 | -6.26812675 | 3.94E-006 | 6.66E-005 | hypothetical protein FLJ22457 | NA |  | | | | | | | | | | | | | | | | | | | | | | | | | | | |
| 1038 | 212890\_at | MGC15523 | -1.129552823 | 9.194021471 | -6.26781869 | 3.95E-006 | 6.66E-005 | hypothetical protein MGC15523 | amino acid transport |  | | | | | | | | | | | | | | | | | | | | | | | | | | | |
| 1039 | 203198\_at | CDK9 | 0.765623859 | 7.274237058 | 6.266659 | 3.96E-006 | 6.68E-005 | cyclin-dependent kinase 9 (CDC2-related kinase) | RNA elongation from Pol II promoter, cell proliferation, protein amino acid phosphorylation, regulation of cell cycle, transcription initiation from Pol II promoter |  | | | | | | | | | | | | | | | | | | | | | | | | | | | |
| 1040 | 218822\_s\_at | NPEPL1 | -0.763092413 | 7.064531367 | -6.26545522 | 3.97E-006 | 6.68E-005 | aminopeptidase-like 1 | protein metabolism, proteolysis and peptidolysis |  | | | | | | | | | | | | | | | | | | | | | | | | | | | |
| 1041 | 209418\_s\_at | C22orf19 | 1.004243746 | 7.854223323 | 6.26395706 | 3.98E-006 | 6.70E-005 | chromosome 22 open reading frame 19 | NA |  | | | | | | | | | | | | | | | | | | | | | | | | | | | |
| 1042 | 200645\_at | GABARAP | -1.176712685 | 11.12702154 | -6.2638773 | 3.98E-006 | 6.70E-005 | GABA(A) receptor-associated protein | protein targeting, protein transport, synaptic transmission |  | | | | | | | | | | | | | | | | | | | | | | | | | | | |
| 1043 | 222030\_at | SIVA | -0.877672281 | 7.011889068 | -6.26306756 | 3.99E-006 | 6.70E-005 | CD27-binding (Siva) protein | apoptosis, defense response, induction of apoptosis by extracellular signals, positive regulation of apoptosis |  | | | | | | | | | | | | | | | | | | | | | | | | | | | |
| 1044 | 203432\_at | TMPO | 1.77467556 | 8.062328189 | 6.26233483 | 3.99E-006 | 6.71E-005 | thymopoietin | NA |  | | | | | | | | | | | | | | | | | | | | | | | | | | | |
| 1045 | 208843\_s\_at | GORASP2 | 1.724887746 | 8.21546 | 6.26201054 | 4.00E-006 | 6.71E-005 | golgi reassembly stacking protein 2, 55kDa | NA |  | | | | | | | | | | | | | | | | | | | | | | | | | | | |
| 1046 | 205639\_at | AOAH | -1.852290742 | 8.325019993 | -6.25284638 | 4.08E-006 | 6.84E-005 | acyloxyacyl hydrolase (neutrophil) | inflammatory response, lipid metabolism |  | | | | | | | | | | | | | | | | | | | | | | | | | | | |
| 1047 | 221580\_s\_at | MGC5306 | 1.93131959 | 9.066401848 | 6.25100607 | 4.09E-006 | 6.86E-005 | hypothetical protein MGC5306 | NA |  | | | | | | | | | | | | | | | | | | | | | | | | | | | |
| 1048 | 213762\_x\_at | RBMX | 1.367015538 | 10.45831645 | 6.24488957 | 4.15E-006 | 6.95E-005 | RNA binding motif protein, X-linked | NA |  | | | | | | | | | | | | | | | | | | | | | | | | | | | |
| 1049 | 219459\_at | POLR3B | 0.526417551 | 7.197369496 | 6.24128913 | 4.18E-006 | 6.99E-005 | polymerase (RNA) III (DNA directed) polypeptide B | transcription |  | | | | | | | | | | | | | | | | | | | | | | | | | | | |
| 1050 | 200082\_s\_at | RPS7 | 1.07394744 | 12.33168423 | 6.24127073 | 4.18E-006 | 6.99E-005 | ribosomal protein S7 /// ribosomal protein S7 | protein biosynthesis |  | | | | | | | | | | | | | | | | | | | | | | | | | | | |
| 1051 | 205505\_at | GCNT1 | -1.671989314 | 7.468230893 | -6.23859126 | 4.20E-006 | 7.03E-005 | glucosaminyl (N-acetyl) transferase 1, core 2 (beta-1,6-N-acetylglucosaminyltransferase) | O-linked glycosylation |  | | | | | | | | | | | | | | | | | | | | | | | | | | | |
| 1052 | 212218\_s\_at | FASN | 1.634811776 | 8.366737998 | 6.23740288 | 4.21E-006 | 7.04E-005 | fatty acid synthase | biosynthesis, fatty acid biosynthesis |  | | | | | | | | | | | | | | | | | | | | | | | | | | | |
| 1053 | 218480\_at | FLJ21839 | 1.200515854 | 7.639380724 | 6.23659517 | 4.22E-006 | 7.04E-005 | hypothetical protein FLJ21839 | proteolysis and peptidolysis |  | | | | | | | | | | | | | | | | | | | | | | | | | | | |
| 1054 | 207616\_s\_at | TANK | -0.990566561 | 9.281141039 | -6.23654124 | 4.22E-006 | 7.04E-005 | TRAF family member-associated NFKB activator | signal transduction |  | | | | | | | | | | | | | | | | | | | | | | | | | | | |
| 1055 | 203308\_x\_at | HPS1 | -0.445602177 | 8.089929128 | -6.23502589 | 4.24E-006 | 7.06E-005 | Hermansky-Pudlak syndrome 1 | NA |  | | | | | | | | | | | | | | | | | | | | | | | | | | | |
| 1056 | 218106\_s\_at | MRPS10 | 1.477229674 | 7.274911111 | 6.23135683 | 4.27E-006 | 7.11E-005 | mitochondrial ribosomal protein S10 | protein biosynthesis |  | | | | | | | | | | | | | | | | | | | | | | | | | | | |
| 1057 | 204822\_at | TTK | 1.779998239 | 6.055048239 | 6.22996167 | 4.28E-006 | 7.12E-005 | TTK protein kinase | mitotic spindle assembly, mitotic spindle checkpoint, positive regulation of cell proliferation, protein amino acid phosphorylation, regulation of cell cycle |  | | | | | | | | | | | | | | | | | | | | | | | | | | | |
| 1058 | 205237\_at | FCN1 | -4.907041473 | 9.384115414 | -6.22706743 | 4.31E-006 | 7.16E-005 | ficolin (collagen/fibrinogen domain containing) 1 | opsonization, phosphate transport |  | | | | | | | | | | | | | | | | | | | | | | | | | | | |
| 1059 | 207824\_s\_at | MAZ | 1.364793386 | 7.734851156 | 6.21968172 | 4.38E-006 | 7.26E-005 | MYC-associated zinc finger protein (purine-binding transcription factor) | regulation of transcription, DNA-dependent, transcription initiation from Pol II promoter, transcription termination from Pol II promoter |  | | | | | | | | | | | | | | | | | | | | | | | | | | | |
| 1060 | 219169\_s\_at | TFB1M | 0.751454854 | 6.578234269 | 6.21878878 | 4.39E-006 | 7.26E-005 | transcription factor B1, mitochondrial | rRNA modification |  | | | | | | | | | | | | | | | | | | | | | | | | | | | |
| 1061 | 218212\_s\_at | MOCS2 | 0.743698336 | 5.484065062 | 6.21699879 | 4.41E-006 | 7.28E-005 | molybdenum cofactor synthesis 2 | Mo-molybdopterin cofactor biosynthesis, sulfur metabolism |  | | | | | | | | | | | | | | | | | | | | | | | | | | | |
| 1062 | 201222\_s\_at | RAD23B | 1.30642592 | 8.766953631 | 6.21689099 | 4.41E-006 | 7.28E-005 | RAD23 homolog B (S. cerevisiae) | nucleotide-excision repair |  | | | | | | | | | | | | | | | | | | | | | | | | | | | |
| 1063 | 200715\_x\_at | RPL13A | 1.026375663 | 12.19900498 | 6.21143016 | 4.46E-006 | 7.35E-005 | ribosomal protein L13a | protein biosynthesis |  | | | | | | | | | | | | | | | | | | | | | | | | | | | |
| 1064 | 204807\_at | TMEM5 | 0.443887761 | 4.204374374 | 6.21043312 | 4.47E-006 | 7.37E-005 | transmembrane protein 5 | NA |  | | | | | | | | | | | | | | | | | | | | | | | | | | | |
| 1065 | 203590\_at | DNCLI2 | -0.891305235 | 7.867087847 | -6.20953557 | 4.48E-006 | 7.37E-005 | dynein, cytoplasmic, light intermediate polypeptide 2 | NA |  | | | | | | | | | | | | | | | | | | | | | | | | | | | |
| 1066 | 219688\_at | BBS7 | 0.470646066 | 4.018608589 | 6.20903503 | 4.48E-006 | 7.38E-005 | Bardet-Biedl syndrome 7 | visual perception |  | | | | | | | | | | | | | | | | | | | | | | | | | | | |
| 1067 | 218046\_s\_at | MRPS16 | 1.23214412 | 7.837647771 | 6.20718878 | 4.50E-006 | 7.40E-005 | mitochondrial ribosomal protein S16 | protein biosynthesis |  | | | | | | | | | | | | | | | | | | | | | | | | | | | |
| 1068 | 204238\_s\_at | C6orf108 | 1.932266904 | 6.956458792 | 6.20262012 | 4.55E-006 | 7.46E-005 | chromosome 6 open reading frame 108 | cell proliferation |  | | | | | | | | | | | | | | | | | | | | | | | | | | | |
| 1069 | 208773\_s\_at | ANKHD1 /// MASK-BP3 | 0.634463525 | 8.531814617 | 6.20143255 | 4.56E-006 | 7.47E-005 | ankyrin repeat and KH domain containing 1 /// MASK-4E-BP3 alternate reading frame gene | NA |  | | | | | | | | | | | | | | | | | | | | | | | | | | | |
| 1070 | 214414\_x\_at | HBA2 | -5.135322669 | 11.11451757 | -6.20037798 | 4.57E-006 | 7.48E-005 | hemoglobin, alpha 2 /// hemoglobin, alpha 2 | NA |  | | | | | | | | | | | | | | | | | | | | | | | | | | | |
| 1071 | 204145\_at | FRG1 | 0.814302749 | 7.849088919 | 6.20008834 | 4.57E-006 | 7.48E-005 | FSHD region gene 1 | NA |  | | | | | | | | | | | | | | | | | | | | | | | | | | | |
| 1072 | 212371\_at | PNAS-4 | 1.131501457 | 7.695233934 | 6.1955968 | 4.62E-006 | 7.54E-005 | CGI-146 protein | NA |  | | | | | | | | | | | | | | | | | | | | | | | | | | | |
| 1073 | 220647\_s\_at | E2IG2 | 1.075748467 | 7.961870858 | 6.19280576 | 4.64E-006 | 7.58E-005 | E2IG2 protein | NA |  | | | | | | | | | | | | | | | | | | | | | | | | | | | |
| 1074 | 210275\_s\_at | ZA20D2 | -1.469514358 | 10.08381154 | -6.19236196 | 4.65E-006 | 7.58E-005 | zinc finger, A20 domain containing 2 | NA |  | | | | | | | | | | | | | | | | | | | | | | | | | | | |
| 1075 | 212693\_at | MDN1 | 1.082612052 | 7.684711041 | 6.19164076 | 4.66E-006 | 7.59E-005 | MDN1, midasin homolog (yeast) | protein complex assembly, protein folding |  | | | | | | | | | | | | | | | | | | | | | | | | | | | |
| 1076 | 200955\_at | IMMT | 1.57966012 | 9.036314722 | 6.19047268 | 4.67E-006 | 7.60E-005 | inner membrane protein, mitochondrial (mitofilin) | NA |  | | | | | | | | | | | | | | | | | | | | | | | | | | | |
| 1077 | 213298\_at | NFIC | -0.536496957 | 7.056896194 | -6.18984499 | 4.67E-006 | 7.60E-005 | nuclear factor I/C (CCAAT-binding transcription factor) | DNA replication, regulation of transcription, DNA-dependent, transcription from Pol II promoter |  | | | | | | | | | | | | | | | | | | | | | | | | | | | |
| 1078 | 209715\_at | CBX5 | 1.018909847 | 6.83445461 | 6.18795187 | 4.69E-006 | 7.63E-005 | chromobox homolog 5 (HP1 alpha homolog, Drosophila) | chromatin assembly or disassembly |  | | | | | | | | | | | | | | | | | | | | | | | | | | | |
| 1079 | 204527\_at | MYO5A | -1.299148914 | 5.627629346 | -6.18652877 | 4.71E-006 | 7.65E-005 | myosin VA (heavy polypeptide 12, myoxin) | actin filament-based movement, transport |  | | | | | | | | | | | | | | | | | | | | | | | | | | | |
| 1080 | 211937\_at | EIF4B | 1.134295932 | 10.85245462 | 6.18291726 | 4.75E-006 | 7.70E-005 | eukaryotic translation initiation factor 4B | protein biosynthesis, regulation of translational initiation |  | | | | | | | | | | | | | | | | | | | | | | | | | | | |
| 1081 | 213092\_x\_at | DNAJC9 | 1.8351864 | 5.60434052 | 6.18183428 | 4.76E-006 | 7.71E-005 | DnaJ (Hsp40) homolog, subfamily C, member 9 | protein folding |  | | | | | | | | | | | | | | | | | | | | | | | | | | | |
| 1082 | 201903\_at | UQCRC1 | 0.72109928 | 9.766598948 | 6.17749698 | 4.80E-006 | 7.77E-005 | ubiquinol-cytochrome c reductase core protein I | aerobic respiration, electron transport, oxidative phosphorylation, proteolysis and peptidolysis |  | | | | | | | | | | | | | | | | | | | | | | | | | | | |
| 1083 | 209561\_at | THBS3 | -0.660554146 | 6.922773283 | -6.17678677 | 4.81E-006 | 7.78E-005 | thrombospondin 3 | cell motility, cell-matrix adhesion |  | | | | | | | | | | | | | | | | | | | | | | | | | | | |
| 1084 | 219733\_s\_at | SLC27A5 | 0.992189581 | 7.045020099 | 6.17639538 | 4.81E-006 | 7.78E-005 | solute carrier family 27 (fatty acid transporter), member 5 | metabolism, very-long-chain fatty acid metabolism |  | | | | | | | | | | | | | | | | | | | | | | | | | | | |
| 1085 | 212125\_at | RANGAP1 | 1.11356047 | 7.966230504 | 6.1754482 | 4.82E-006 | 7.79E-005 | Ran GTPase activating protein 1 | signal transduction |  | | | | | | | | | | | | | | | | | | | | | | | | | | | |
| 1086 | 202606\_s\_at | TLK1 | 1.128857492 | 7.249267378 | 6.1738339 | 4.84E-006 | 7.81E-005 | tousled-like kinase 1 | cell cycle, chromatin modification, intracellular protein transport, intracellular signaling cascade, protein amino acid phosphorylation, regulation of chromatin assembly or disassembly, response to DNA damage stimulus |  | | | | | | | | | | | | | | | | | | | | | | | | | | | |
| 1087 | 200934\_at | DEK | 1.133606099 | 9.258717768 | 6.17263889 | 4.85E-006 | 7.83E-005 | DEK oncogene (DNA binding) | SRP-dependent cotranslational protein-membrane targeting, cell growth and/or maintenance, regulation of transcription from Pol II promoter, signal transduction, viral genome replication |  | | | | | | | | | | | | | | | | | | | | | | | | | | | |
| 1088 | 203259\_s\_at | C6orf74 | 1.741813317 | 8.61425718 | 6.17237443 | 4.86E-006 | 7.83E-005 | chromosome 6 open reading frame 74 | NA |  | | | | | | | | | | | | | | | | | | | | | | | | | | | |
| 1089 | 219120\_at | FLJ21945 | 0.620060052 | 6.025355573 | 6.17033059 | 4.88E-006 | 7.86E-005 | hypothetical protein FLJ21945 | NA |  | | | | | | | | | | | | | | | | | | | | | | | | | | | |
| 1090 | 32091\_at | KIAA0446 | -0.818527728 | 6.816396337 | -6.16943769 | 4.89E-006 | 7.87E-005 | KIAA0446 gene product | transport |  | | | | | | | | | | | | | | | | | | | | | | | | | | | |
| 1091 | 203077\_s\_at | SMAD2 | 0.96794237 | 6.097850039 | 6.16632783 | 4.92E-006 | 7.91E-005 | SMAD, mothers against DPP homolog 2 (Drosophila) | regulation of transcription, DNA-dependent, signal transduction |  | | | | | | | | | | | | | | | | | | | | | | | | | | | |
| 1092 | 202029\_x\_at | RPL38 | 0.537209661 | 12.56940097 | 6.16627611 | 4.92E-006 | 7.91E-005 | ribosomal protein L38 | protein biosynthesis |  | | | | | | | | | | | | | | | | | | | | | | | | | | | |
| 1093 | 211755\_s\_at | ATP5F1 | 1.13727568 | 10.92631693 | 6.16204274 | 4.97E-006 | 7.98E-005 | ATP synthase, H+ transporting, mitochondrial F0 complex, subunit b, isoform 1 /// ATP synthase, H+ transporting, mitochondrial F0 complex, subunit b, isoform 1 | ATP synthesis coupled proton transport, proton transport |  | | | | | | | | | | | | | | | | | | | | | | | | | | | |
| 1094 | 215761\_at | RC3 | -0.862823723 | 7.269336322 | -6.16132604 | 4.97E-006 | 7.98E-005 | rabconnectin-3 | NA |  | | | | | | | | | | | | | | | | | | | | | | | | | | | |
| 1095 | 218973\_at | EFTUD1 | 0.958189695 | 8.288325598 | 6.16036358 | 4.98E-006 | 7.99E-005 | elongation factor Tu GTP binding domain containing 1 | protein biosynthesis |  | | | | | | | | | | | | | | | | | | | | | | | | | | | |
| 1096 | 214290\_s\_at | HIST2H2AA | -2.943211209 | 11.26740469 | -6.15959604 | 4.99E-006 | 8.00E-005 | histone 2, H2aa | chromosome organization and biogenesis (sensu Eukaryota), nucleosome assembly |  | | | | | | | | | | | | | | | | | | | | | | | | | | | |
| 1097 | 219200\_at | MGC5297 | 1.401593404 | 6.447085788 | 6.15622102 | 5.03E-006 | 8.06E-005 | hypothetical protein MGC5297 | NA |  | | | | | | | | | | | | | | | | | | | | | | | | | | | |
| 1098 | 212245\_at | MCFD2 | 2.154141705 | 6.791134515 | 6.15551254 | 5.04E-006 | 8.06E-005 | multiple coagulation factor deficiency 2 | NA |  | | | | | | | | | | | | | | | | | | | | | | | | | | | |
| 1099 | 212104\_s\_at | RBM9 | 0.796607217 | 5.813371253 | 6.15210276 | 5.08E-006 | 8.10E-005 | RNA binding motif protein 9 | RNA metabolism, estrogen receptor signaling pathway, negative regulation of transcription, regulation of cell proliferation |  | | | | | | | | | | | | | | | | | | | | | | | | | | | |
| 1100 | 222029\_x\_at | HKE2 | 0.806267166 | 7.579878452 | 6.15198631 | 5.08E-006 | 8.10E-005 | HLA class II region expressed gene KE2 | protein folding |  | | | | | | | | | | | | | | | | | | | | | | | | | | | |
| 1101 | 203743\_s\_at | TDG | 1.323337163 | 7.133125862 | 6.15155496 | 5.08E-006 | 8.10E-005 | thymine-DNA glycosylase | base-excision repair, carbohydrate metabolism |  | | | | | | | | | | | | | | | | | | | | | | | | | | | |
| 1102 | 212160\_at | XPOT | 2.26642117 | 8.551105853 | 6.15147354 | 5.08E-006 | 8.10E-005 | exportin, tRNA (nuclear export receptor for tRNAs) | transport |  | | | | | | | | | | | | | | | | | | | | | | | | | | | |
| 1103 | 202370\_s\_at | CBFB | 1.178030655 | 9.346419502 | 6.14772228 | 5.12E-006 | 8.16E-005 | core-binding factor, beta subunit | cell growth and/or maintenance, transcription from Pol II promoter |  | | | | | | | | | | | | | | | | | | | | | | | | | | | |
| 1104 | 212138\_at | SCC-112 | 1.020799466 | 8.403462556 | 6.14666611 | 5.14E-006 | 8.17E-005 | SCC-112 protein | NA |  | | | | | | | | | | | | | | | | | | | | | | | | | | | |
| 1105 | 203246\_s\_at | TUSC4 | 0.625379756 | 8.375188118 | 6.1461426 | 5.14E-006 | 8.18E-005 | tumor suppressor candidate 4 | negative regulation of cell cycle |  | | | | | | | | | | | | | | | | | | | | | | | | | | | |
| 1106 | 202179\_at | BLMH | 1.803736257 | 8.019532466 | 6.13535215 | 5.26E-006 | 8.36E-005 | bleomycin hydrolase | proteolysis and peptidolysis |  | | | | | | | | | | | | | | | | | | | | | | | | | | | |
| 1107 | 55616\_at | PERLD1 | -0.406482269 | 7.937893686 | -6.13457367 | 5.27E-006 | 8.37E-005 | per1-like domain containing 1 | NA |  | | | | | | | | | | | | | | | | | | | | | | | | | | | |
| 1108 | 202961\_s\_at | ATP5J2 | 1.045673827 | 10.58186398 | 6.13113325 | 5.31E-006 | 8.41E-005 | ATP synthase, H+ transporting, mitochondrial F0 complex, subunit f, isoform 2 | ATP biosynthesis, proton transport |  | | | | | | | | | | | | | | | | | | | | | | | | | | | |
| 1109 | 220525\_s\_at | AUP1 | 1.002489834 | 8.682400923 | 6.12954957 | 5.33E-006 | 8.44E-005 | ancient ubiquitous protein 1 | NA |  | | | | | | | | | | | | | | | | | | | | | | | | | | | |
| 1110 | 201226\_at | NDUFB8 | 0.774517042 | 9.59723812 | 6.12867722 | 5.34E-006 | 8.44E-005 | NADH dehydrogenase (ubiquinone) 1 beta subcomplex, 8, 19kDa | electron transport, mitochondrial electron transport, NADH to ubiquinone |  | | | | | | | | | | | | | | | | | | | | | | | | | | | |
| 1111 | 202930\_s\_at | SUCLA2 | 1.944499624 | 7.103482063 | 6.12857616 | 5.34E-006 | 8.44E-005 | succinate-CoA ligase, ADP-forming, beta subunit | metabolism, succinyl-CoA pathway, tricarboxylic acid cycle |  | | | | | | | | | | | | | | | | | | | | | | | | | | | |
| 1112 | 218379\_at | RBM7 | 1.20305308 | 7.835738013 | 6.12576592 | 5.38E-006 | 8.48E-005 | RNA binding motif protein 7 | meiosis |  | | | | | | | | | | | | | | | | | | | | | | | | | | | |
| 1113 | 211686\_s\_at | LOC84549 | 1.332574909 | 7.93473637 | 6.12344222 | 5.40E-006 | 8.52E-005 | RNA binding protein /// RNA binding protein | NA |  | | | | | | | | | | | | | | | | | | | | | | | | | | | |
| 1114 | 200963\_x\_at | RPL31 | 0.654696952 | 12.96214258 | 6.12216866 | 5.42E-006 | 8.54E-005 | ribosomal protein L31 | NA |  | | | | | | | | | | | | | | | | | | | | | | | | | | | |
| 1115 | 203832\_at | SNRPF | 1.549138489 | 9.452460918 | 6.12163227 | 5.42E-006 | 8.54E-005 | small nuclear ribonucleoprotein polypeptide F | NA |  | | | | | | | | | | | | | | | | | | | | | | | | | | | |
| 1116 | 209820\_s\_at | TBL3 | 0.685066812 | 7.778299516 | 6.12134731 | 5.43E-006 | 8.54E-005 | transducin (beta)-like 3 | G-protein signaling, coupled to cGMP nucleotide second messenger |  | | | | | | | | | | | | | | | | | | | | | | | | | | | |
| 1117 | 201861\_s\_at | LRRFIP1 | -1.151095532 | 9.684667921 | -6.11998327 | 5.44E-006 | 8.55E-005 | leucine rich repeat (in FLII) interacting protein 1 | negative regulation of transcription, regulation of transcription from Pol II promoter |  | | | | | | | | | | | | | | | | | | | | | | | | | | | |
| 1118 | 214167\_s\_at | RPLP0 | 1.497074655 | 11.61275784 | 6.11769573 | 5.47E-006 | 8.59E-005 | ribosomal protein, large, P0 | protein biosynthesis, translational elongation |  | | | | | | | | | | | | | | | | | | | | | | | | | | | |
| 1119 | 201030\_x\_at | LDHB | 1.904229435 | 11.5059528 | 6.11564869 | 5.50E-006 | 8.62E-005 | lactate dehydrogenase B | glycolysis |  | | | | | | | | | | | | | | | | | | | | | | | | | | | |
| 1120 | 215084\_s\_at | MGC8974 | 1.085366703 | 6.077353932 | 6.11535967 | 5.50E-006 | 8.62E-005 | hypothetical protein MGC8974 | NA |  | | | | | | | | | | | | | | | | | | | | | | | | | | | |
| 1121 | 208152\_s\_at | DDX21 | 1.545070563 | 9.683163769 | 6.11465362 | 5.51E-006 | 8.63E-005 | DEAD (Asp-Glu-Ala-Asp) box polypeptide 21 /// DEAD (Asp-Glu-Ala-Asp) box polypeptide 21 | NA |  | | | | | | | | | | | | | | | | | | | | | | | | | | | |
| 1122 | 200657\_at | SLC25A5 | 1.408363242 | 11.6174514 | 6.11368749 | 5.52E-006 | 8.64E-005 | solute carrier family 25 (mitochondrial carrier; adenine nucleotide translocator), member 5 | mitochondrial transport, transport |  | | | | | | | | | | | | | | | | | | | | | | | | | | | |
| 1123 | 219267\_at | GLTP | -0.935348134 | 7.066213127 | -6.11230611 | 5.54E-006 | 8.66E-005 | glycolipid transfer protein | NA |  | | | | | | | | | | | | | | | | | | | | | | | | | | | |
| 1124 | 207979\_s\_at | CD8B1 | -0.611578323 | 7.335746756 | -6.1089658 | 5.58E-006 | 8.71E-005 | CD8 antigen, beta polypeptide 1 (p37) | T-cell activation, immune response, transmembrane receptor protein tyrosine kinase signaling pathway |  | | | | | | | | | | | | | | | | | | | | | | | | | | | |
| 1125 | 212360\_at | AMPD2 | -1.296975938 | 8.976964298 | -6.10615519 | 5.61E-006 | 8.75E-005 | adenosine monophosphate deaminase 2 (isoform L) | purine nucleotide metabolism, purine ribonucleoside monophosphate biosynthesis |  | | | | | | | | | | | | | | | | | | | | | | | | | | | |
| 1126 | 209313\_at | XAB1 | 0.973364765 | 8.867024672 | 6.1034762 | 5.64E-006 | 8.80E-005 | XPA binding protein 1 | small GTPase mediated signal transduction |  | | | | | | | | | | | | | | | | | | | | | | | | | | | |
| 1127 | 218558\_s\_at | MRPL39 | 1.163943239 | 7.777347476 | 6.10304357 | 5.65E-006 | 8.80E-005 | mitochondrial ribosomal protein L39 | NA |  | | | | | | | | | | | | | | | | | | | | | | | | | | | |
| 1128 | 203528\_at | SEMA4D | -1.870134909 | 8.608393683 | -6.10222513 | 5.66E-006 | 8.81E-005 | sema domain, immunoglobulin domain (Ig), transmembrane domain (TM) and short cytoplasmic domain, (semaphorin) 4D | anti-apoptosis, cell adhesion, development, immune response, neurogenesis, protein complex assembly, urea metabolism |  | | | | | | | | | | | | | | | | | | | | | | | | | | | |
| 1129 | 209936\_at | RBM5 | -1.405062367 | 7.248311263 | -6.10116864 | 5.67E-006 | 8.83E-005 | RNA binding motif protein 5 | RNA processing, negative regulation of cell cycle |  | | | | | | | | | | | | | | | | | | | | | | | | | | | |
| 1130 | 210334\_x\_at | BIRC5 | 1.1469789 | 8.448966361 | 6.09933121 | 5.70E-006 | 8.85E-005 | baculoviral IAP repeat-containing 5 (survivin) | G2/M transition of mitotic cell cycle, anti-apoptosis, apoptosis |  | | | | | | | | | | | | | | | | | | | | | | | | | | | |
| 1131 | 49485\_at | PRDM4 | 0.439321802 | 6.956543353 | 6.09551433 | 5.74E-006 | 8.92E-005 | PR domain containing 4 | cell proliferation, regulation of transcription, DNA-dependent, signal transduction, transcription from Pol II promoter |  | | | | | | | | | | | | | | | | | | | | | | | | | | | |
| 1132 | 201292\_at | TOP2A | 2.706080692 | 7.856672802 | 6.09211985 | 5.79E-006 | 8.96E-005 | topoisomerase (DNA) II alpha 170kDa | DNA topological change |  | | | | | | | | | | | | | | | | | | | | | | | | | | | |
| 1133 | 200676\_s\_at | UBE2L3 | 1.241648963 | 7.642952158 | 6.09142916 | 5.80E-006 | 8.97E-005 | ubiquitin-conjugating enzyme E2L 3 | protein modification, ubiquitin cycle, ubiquitin-dependent protein catabolism |  | | | | | | | | | | | | | | | | | | | | | | | | | | | |
| 1134 | 219437\_s\_at | ANKRD11 | -0.936176978 | 7.999059043 | -6.09048601 | 5.81E-006 | 8.98E-005 | ankyrin repeat domain 11 | NA |  | | | | | | | | | | | | | | | | | | | | | | | | | | | |
| 1135 | 217805\_at | ILF3 | 1.713209122 | 8.001986301 | 6.08883467 | 5.83E-006 | 9.01E-005 | interleukin enhancer binding factor 3, 90kDa | M phase, regulation of transcription, DNA-dependent |  | | | | | | | | | | | | | | | | | | | | | | | | | | | |
| 1136 | 212083\_at | TEX261 | 0.403291855 | 8.935850731 | 6.08773507 | 5.84E-006 | 9.03E-005 | testis expressed sequence 261 | NA |  | | | | | | | | | | | | | | | | | | | | | | | | | | | |
| 1137 | 218481\_at | EXOSC5 | 1.117694642 | 8.21008945 | 6.08714335 | 5.85E-006 | 9.03E-005 | exosome component 5 | rRNA processing |  | | | | | | | | | | | | | | | | | | | | | | | | | | | |
| 1138 | 205756\_s\_at | F8 | -0.595036303 | 6.1553832 | -6.08463517 | 5.88E-006 | 9.07E-005 | coagulation factor VIII, procoagulant component (hemophilia A) | acute-phase response, blood coagulation, cell adhesion |  | | | | | | | | | | | | | | | | | | | | | | | | | | | |
| 1139 | 217816\_s\_at | PCNP | 0.806823314 | 9.003837439 | 6.08272543 | 5.91E-006 | 9.11E-005 | PEST-containing nuclear protein | NA |  | | | | | | | | | | | | | | | | | | | | | | | | | | | |
| 1140 | 201584\_s\_at | DDX39 | 1.280496765 | 9.606764568 | 6.08040335 | 5.94E-006 | 9.15E-005 | DEAD (Asp-Glu-Ala-Asp) box polypeptide 39 | NA |  | | | | | | | | | | | | | | | | | | | | | | | | | | | |
| 1141 | 211013\_x\_at | PML | 0.602951617 | 7.829216602 | 6.07803036 | 5.97E-006 | 9.18E-005 | promyelocytic leukemia | cell growth and/or maintenance, protein ubiquitination, regulation of transcription, DNA-dependent |  | | | | | | | | | | | | | | | | | | | | | | | | | | | |
| 1142 | 208847\_s\_at | ADH5 | 1.002001809 | 8.197850169 | 6.07423678 | 6.02E-006 | 9.25E-005 | alcohol dehydrogenase 5 (class III), chi polypeptide | ethanol oxidation |  | | | | | | | | | | | | | | | | | | | | | | | | | | | |
| 1143 | 214931\_s\_at | SRPK2 | 0.531884157 | 6.771267957 | 6.06934232 | 6.08E-006 | 9.34E-005 | SFRS protein kinase 2 | protein amino acid phosphorylation |  | | | | | | | | | | | | | | | | | | | | | | | | | | | |
| 1144 | 211297\_s\_at | CDK7 | 1.333509535 | 7.153467139 | 6.0686019 | 6.09E-006 | 9.34E-005 | cyclin-dependent kinase 7 (MO15 homolog, Xenopus laevis, cdk-activating kinase) | DNA repair, cytokinesis, protein amino acid phosphorylation, regulation of cyclin dependent protein kinase activity, regulation of transcription, DNA-dependent, transcription initiation from Pol II promoter |  | | | | | | | | | | | | | | | | | | | | | | | | | | | |
| 1145 | 214693\_x\_at | MGC8902 /// LOC376745 /// DJ328E19.C1.1 /// LOC200030 /// LOC348482 | -1.010508642 | 9.712320619 | -6.06309175 | 6.17E-006 | 9.45E-005 | hypothetical protein MGC8902 /// AG1 /// hypothetical protein DJ328E19.C1.1 /// hypothetical protein LOC200030 /// hypothetical protein LOC348482 | NA |  | | | | | | | | | | | | | | | | | | | | | | | | | | | |
| 1146 | 218529\_at | 8D6A | 1.471867051 | 8.207880792 | 6.05122172 | 6.33E-006 | 9.66E-005 | 8D6 antigen | regulation of cell growth |  | | | | | | | | | | | | | | | | | | | | | | | | | | | |
| 1147 | 207170\_s\_at | LETMD1 | 1.654681546 | 8.780800036 | 6.04824745 | 6.37E-006 | 9.71E-005 | LETM1 domain containing 1 | NA |  | | | | | | | | | | | | | | | | | | | | | | | | | | | |
| 1148 | 210240\_s\_at | CDKN2D | -1.870128986 | 7.828396446 | -6.04578625 | 6.41E-006 | 9.76E-005 | cyclin-dependent kinase inhibitor 2D (p19, inhibits CDK4) | cell cycle, cell cycle arrest, negative regulation of cell cycle, negative regulation of cell proliferation, regulation of cyclin dependent protein kinase activity |  | | | | | | | | | | | | | | | | | | | | | | | | | | | |
| 1149 | 201001\_s\_at | UBE2V1 /// Kua-UEV | 1.029525092 | 8.229295894 | 6.04463252 | 6.42E-006 | 9.77E-005 | ubiquitin-conjugating enzyme E2 variant 1 /// ubiquitin-conjugating enzyme E2 variant 1 | regulation of transcription, DNA-dependent, ubiquitin cycle |  | | | | | | | | | | | | | | | | | | | | | | | | | | | |
| 1150 | 201189\_s\_at | ITPR3 | 1.071098795 | 6.876822389 | 6.04460446 | 6.42E-006 | 9.77E-005 | inositol 1,4,5-triphosphate receptor, type 3 | calcium ion transport, cation transport |  | | | | | | | | | | | | | | | | | | | | | | | | | | | |
| 1151 | 218258\_at | POLR1D | 1.617079274 | 9.497522431 | 6.04062497 | 6.48E-006 | 9.84E-005 | polymerase (RNA) I polypeptide D, 16kDa | transcription |  | | | | | | | | | | | | | | | | | | | | | | | | | | | |
| 1152 | 220346\_at | MGC72244 | 0.645438659 | 4.417160159 | 6.03639502 | 6.54E-006 | 9.92E-005 | similar to Bifunctional methylenetetrahydrofolate dehydrogenase/cyclohydrolase, mitochondrial precursor | NA |  | | | | | | | | | | | | | | | | | | | | | | | | | | | |
| 1153 | 40189\_at | SET | 1.325511311 | 9.41884229 | 6.03578998 | 6.55E-006 | 9.92E-005 | SET translocation (myeloid leukemia-associated) | DNA replication, cell growth and/or maintenance, negative regulation of histone acetylation, nucleocytoplasmic transport, nucleosome assembly, nucleosome disassembly |  | | | | | | | | | | | | | | | | | | | | | | | | | | | |
| 1154 | 203356\_at | CAPN7 | 0.918068274 | 5.67492626 | 6.03482458 | 6.56E-006 | 9.94E-005 | calpain 7 | proteolysis and peptidolysis |  | | | | | | | | | | | | | | | | | | | | | | | | | | | |
| 1155 | 212895\_s\_at | ABR | -0.905577292 | 8.736351296 | -6.03443804 | 6.57E-006 | 9.94E-005 | active BCR-related gene | small GTPase mediated signal transduction |  | | | | | | | | | | | | | | | | | | | | | | | | | | | |
| 1156 | 216574\_s\_at | RPE | 0.775135669 | 6.260396505 | 6.03283074 | 6.59E-006 | 9.97E-005 | ribulose-5-phosphate-3-epimerase | carbohydrate metabolism |  | | | | | | | | | | | | | | | | | | | | | | | | | | | |
| 1157 | 201400\_at | PSMB3 | 1.019311122 | 10.09484863 | 6.03204736 | 6.60E-006 | 9.97E-005 | proteasome (prosome, macropain) subunit, beta type, 3 | ubiquitin-dependent protein catabolism |  | | | | | | | | | | | | | | | | | | | | | | | | | | | |
| 1158 | 208941\_s\_at | SEPHS1 | 0.680958316 | 7.481385565 | 6.03169894 | 6.61E-006 | 9.97E-005 | selenophosphate synthetase 1 | protein modification |  | | | | | | | | | | | | | | | | | | | | | | | | | | | |
| 1159 | 217773\_s\_at | NDUFA4 | 1.132160818 | 10.13457862 | 6.03043441 | 6.63E-006 | 9.99E-005 | NADH dehydrogenase (ubiquinone) 1 alpha subcomplex, 4, 9kDa | NA |  | | | | | | | | | | | | | | | | | | | | | | | | | | | |
| 1160 | 208987\_s\_at | FBXL11 | -0.626107862 | 8.180354249 | -6.02929633 | 6.64E-006 | 0.00010004 | F-box and leucine-rich repeat protein 11 | regulation of transcription, DNA-dependent, ubiquitin cycle |  | | | | | | | | | | | | | | | | | | | | | | | | | | | |
| 1161 | 202397\_at | NUTF2 | 1.313233988 | 7.917676879 | 6.02474084 | 6.71E-006 | 0.000100842 | nuclear transport factor 2 | protein transport, protein-nucleus import |  | | | | | | | | | | | | | | | | | | | | | | | | | | | |
| 1162 | 203858\_s\_at | COX10 | 0.568076274 | 8.164523858 | 6.0221295 | 6.75E-006 | 0.000101354 | COX10 homolog, cytochrome c oxidase assembly protein, heme A: farnesyltransferase (yeast) | heme biosynthesis |  | | | | | | | | | | | | | | | | | | | | | | | | | | | |
| 1163 | 91816\_f\_at | RKHD1 | 0.903234428 | 5.392891294 | 6.02172424 | 6.75E-006 | 0.000101376 | ring finger and KH domain containing 1 | NA |  | | | | | | | | | | | | | | | | | | | | | | | | | | | |
| 1164 | 214877\_at | CDKAL1 | 0.682179113 | 6.667169899 | 6.0209433 | 6.77E-006 | 0.000101482 | CDK5 regulatory subunit associated protein 1-like 1 | NA |  | | | | | | | | | | | | | | | | | | | | | | | | | | | |
| 1165 | 37796\_at | LRCH4 | -0.775244842 | 8.215574094 | -6.01853121 | 6.80E-006 | 0.000101884 | leucine-rich repeats and calponin homology (CH) domain containing 4 | neurogenesis |  | | | | | | | | | | | | | | | | | | | | | | | | | | | |
| 1166 | 210320\_s\_at | DDX52 | 0.496111184 | 6.46337874 | 6.01780424 | 6.81E-006 | 0.000101978 | DEAD (Asp-Glu-Ala-Asp) box polypeptide 52 | NA |  | | | | | | | | | | | | | | | | | | | | | | | | | | | |
| 1167 | 200873\_s\_at | CCT8 | 1.890879327 | 10.36484225 | 6.01652984 | 6.83E-006 | 0.000102196 | chaperonin containing TCP1, subunit 8 (theta) | protein folding |  | | | | | | | | | | | | | | | | | | | | | | | | | | | |
| 1168 | 222231\_s\_at | PRO1855 | 1.305627819 | 9.565437361 | 6.01540036 | 6.85E-006 | 0.000102369 | hypothetical protein PRO1855 | NA |  | | | | | | | | | | | | | | | | | | | | | | | | | | | |
| 1169 | 207187\_at | JAK3 | -0.509118999 | 8.142841578 | -6.01291509 | 6.89E-006 | 0.000102772 | Janus kinase 3 (a protein tyrosine kinase, leukocyte) | cell growth and/or maintenance, intracellular signaling cascade, mesoderm development, protein amino acid phosphorylation |  | | | | | | | | | | | | | | | | | | | | | | | | | | | |
| 1170 | 218729\_at | LXN | -2.492070514 | 7.26623463 | -6.01275289 | 6.89E-006 | 0.000102772 | latexin | NA |  | | | | | | | | | | | | | | | | | | | | | | | | | | | |
| 1171 | 219849\_at | FLJ23506 | -0.531762012 | 7.045166833 | -6.01173196 | 6.90E-006 | 0.000102865 | hypothetical protein FLJ23506 | regulation of transcription, DNA-dependent |  | | | | | | | | | | | | | | | | | | | | | | | | | | | |
| 1172 | 209026\_x\_at | OK/SW-cl.56 | 1.42565657 | 11.51269155 | 6.01104182 | 6.91E-006 | 0.000102952 | beta 5-tubulin | microtubule polymerization, microtubule-based movement, natural killer cell mediated cytotoxicity |  | | | | | | | | | | | | | | | | | | | | | | | | | | | |
| 1173 | 209034\_at | PNRC1 | -1.262376139 | 8.594750535 | -6.00936141 | 6.94E-006 | 0.000103195 | proline-rich nuclear receptor coactivator 1 | NA |  | | | | | | | | | | | | | | | | | | | | | | | | | | | |
| 1174 | 209279\_s\_at | NSDHL | 1.217595224 | 6.451421311 | 6.00402445 | 7.02E-006 | 0.000104275 | NAD(P) dependent steroid dehydrogenase-like | cholesterol biosynthesis, steroid biosynthesis |  | | | | | | | | | | | | | | | | | | | | | | | | | | | |
| 1175 | 218349\_s\_at | FLJ10036 | 1.013019954 | 7.192611896 | 6.00096655 | 7.07E-006 | 0.000104793 | Zwilch | NA |  | | | | | | | | | | | | | | | | | | | | | | | | | | | |
| 1176 | 201600\_at | REA | 1.356360502 | 11.08249638 | 6.00086377 | 7.07E-006 | 0.000104793 | repressor of estrogen receptor activity | negative regulation of transcription |  | | | | | | | | | | | | | | | | | | | | | | | | | | | |
| 1177 | 201322\_at | ATP5B | 1.410121804 | 11.19330734 | 5.99918422 | 7.10E-006 | 0.000105112 | ATP synthase, H+ transporting, mitochondrial F1 complex, beta polypeptide | ATP synthesis coupled proton transport, energy pathways, proton transport |  | | | | | | | | | | | | | | | | | | | | | | | | | | | |
| 1178 | 217576\_x\_at | SOS2 | -1.040226819 | 7.441646947 | -5.99730937 | 7.13E-006 | 0.000105429 | son of sevenless homolog 2 (Drosophila) | small GTPase mediated signal transduction |  | | | | | | | | | | | | | | | | | | | | | | | | | | | |
| 1179 | 217777\_s\_at | HSPC121 | 1.557361512 | 7.121609771 | 5.99718073 | 7.13E-006 | 0.000105429 | butyrate-induced transcript 1 | I-kappaB kinase/NF-kappaB cascade, Rac protein signal transduction, activation of JUNK, small GTPase mediated signal transduction |  | | | | | | | | | | | | | | | | | | | | | | | | | | | |
| 1180 | 212167\_s\_at | SMARCB1 | 0.928080957 | 8.469354848 | 5.99689288 | 7.13E-006 | 0.000105429 | SWI/SNF related, matrix associated, actin dependent regulator of chromatin, subfamily b, member 1 | DNA integration, chromatin remodeling, negative regulation of cell cycle, regulation of transcription from Pol II promoter |  | | | | | | | | | | | | | | | | | | | | | | | | | | | |
| 1181 | 201088\_at | KPNA2 | 1.647584717 | 8.994749089 | 5.99660854 | 7.14E-006 | 0.000105429 | karyopherin alpha 2 (RAG cohort 1, importin alpha 1) | DNA metabolism, G2 phase of mitotic cell cycle, M phase specific microtubule process, NLS-bearing substrate-nucleus import, intracellular protein transport, regulation of DNA recombination |  | | | | | | | | | | | | | | | | | | | | | | | | | | | |
| 1182 | 200051\_at | SART1 | 0.732654666 | 7.749183242 | 5.99459805 | 7.17E-006 | 0.000105686 | squamous cell carcinoma antigen recognised by T cells /// squamous cell carcinoma antigen recognised by T cells | NA |  | | | | | | | | | | | | | | | | | | | | | | | | | | | |
| 1183 | 219007\_at | NUP43 | 1.569670964 | 7.945361424 | 5.99403287 | 7.18E-006 | 0.000105747 | nucleoporin 43kDa | protein transport |  | | | | | | | | | | | | | | | | | | | | | | | | | | | |
| 1184 | 208502\_s\_at | PITX1 | 1.707335436 | 5.349137931 | 5.9913228 | 7.22E-006 | 0.00010631 | paired-like homeodomain transcription factor 1 | morphogenesis, regulation of transcription, DNA-dependent, skeletal development |  | | | | | | | | | | | | | | | | | | | | | | | | | | | |
| 1185 | 202822\_at | LPP | -1.031289921 | 7.74028774 | -5.99101539 | 7.23E-006 | 0.000106312 | LIM domain containing preferred translocation partner in lipoma | NA |  | | | | | | | | | | | | | | | | | | | | | | | | | | | |
| 1186 | 218085\_at | SNF7DC2 | 1.394879585 | 7.27223438 | 5.98841855 | 7.27E-006 | 0.00010678 | SNF7 domain containing 2 | NA |  | | | | | | | | | | | | | | | | | | | | | | | | | | | |
| 1187 | 201405\_s\_at | COPS6 | 1.191513108 | 9.21858506 | 5.98798809 | 7.27E-006 | 0.000106811 | COP9 constitutive photomorphogenic homolog subunit 6 (Arabidopsis) | NA |  | | | | | | | | | | | | | | | | | | | | | | | | | | | |
| 1188 | 209262\_s\_at | NR2F6 | 1.056874677 | 7.442096588 | 5.98680417 | 7.29E-006 | 0.000107001 | nuclear receptor subfamily 2, group F, member 6 | regulation of transcription, DNA-dependent, signal transduction |  | | | | | | | | | | | | | | | | | | | | | | | | | | | |
| 1189 | 212892\_at | ZNF282 | 0.736598893 | 8.089132046 | 5.98635912 | 7.30E-006 | 0.000107001 | zinc finger protein 282 | regulation of transcription, DNA-dependent |  | | | | | | | | | | | | | | | | | | | | | | | | | | | |
| 1190 | 201699\_at | PSMC6 | 1.629421888 | 7.71516394 | 5.98628422 | 7.30E-006 | 0.000107001 | proteasome (prosome, macropain) 26S subunit, ATPase, 6 | protein catabolism |  | | | | | | | | | | | | | | | | | | | | | | | | | | | |
| 1191 | 204426\_at | RNP24 | 1.454091351 | 7.72555353 | 5.98352719 | 7.35E-006 | 0.000107511 | coated vesicle membrane protein | intracellular protein transport |  | | | | | | | | | | | | | | | | | | | | | | | | | | | |
| 1192 | 221548\_s\_at | ILKAP | 0.786786871 | 7.792388852 | 5.98237466 | 7.37E-006 | 0.000107714 | integrin-linked kinase-associated serine/threonine phosphatase 2C | integrin-mediated signaling pathway, protein amino acid dephosphorylation |  | | | | | | | | | | | | | | | | | | | | | | | | | | | |
| 1193 | 218472\_s\_at | PELO | 0.91480027 | 6.724344798 | 5.98179136 | 7.37E-006 | 0.000107781 | pelota homolog (Drosophila) | protein biosynthesis |  | | | | | | | | | | | | | | | | | | | | | | | | | | | |
| 1194 | 217860\_at | NDUFA10 | 1.329928332 | 9.20352536 | 5.98080684 | 7.39E-006 | 0.000107944 | NADH dehydrogenase (ubiquinone) 1 alpha subcomplex, 10, 42kDa | energy pathways, nucleobase, nucleoside, nucleotide and nucleic acid metabolism |  | | | | | | | | | | | | | | | | | | | | | | | | | | | |
| 1195 | 205060\_at | PARG | 0.517271993 | 6.418999101 | 5.9755672 | 7.48E-006 | 0.000108947 | poly (ADP-ribose) glycohydrolase | NA |  | | | | | | | | | | | | | | | | | | | | | | | | | | | |
| 1196 | 200922\_at | KDELR1 | 1.4030569 | 8.895719163 | 5.97529209 | 7.48E-006 | 0.000108947 | KDEL (Lys-Asp-Glu-Leu) endoplasmic reticulum protein retention receptor 1 | intracellular protein transport, protein transport |  | | | | | | | | | | | | | | | | | | | | | | | | | | | |
| 1197 | 213612\_x\_at | MGC8902 /// LOC376745 /// LOC348482 | -0.61955266 | 9.996256763 | -5.97512561 | 7.48E-006 | 0.000108947 | hypothetical protein MGC8902 /// AG1 /// hypothetical protein LOC348482 | NA |  | | | | | | | | | | | | | | | | | | | | | | | | | | | |
| 1198 | 218538\_s\_at | MRS2L | 1.571401966 | 5.514001059 | 5.97328819 | 7.51E-006 | 0.000109286 | MRS2-like, magnesium homeostasis factor (S. cerevisiae) | metal ion transport |  | | | | | | | | | | | | | | | | | | | | | | | | | | | |
| 1199 | 208947\_s\_at | RENT1 | 0.625990096 | 7.172595734 | 5.97312117 | 7.52E-006 | 0.000109286 | regulator of nonsense transcripts 1 | DNA restriction, mRNA catabolism, nonsense-mediated decay, regulation of translational termination |  | | | | | | | | | | | | | | | | | | | | | | | | | | | |
| 1200 | 208991\_at | STAT3 | -1.039926739 | 10.03434735 | -5.97089535 | 7.55E-006 | 0.000109752 | signal transducer and activator of transcription 3 (acute-phase response factor) | JAK-STAT cascade, acute-phase response, cell motility, intracellular signaling cascade, negative regulation of transcription from Pol II promoter, neurogenesis, regulation of transcription, DNA-dependent |  | | | | | | | | | | | | | | | | | | | | | | | | | | | |
| 1201 | 218488\_at | EIF2B3 | 1.453724478 | 7.954708607 | 5.96989729 | 7.57E-006 | 0.000109922 | eukaryotic translation initiation factor 2B, subunit 3 gamma, 58kDa | biosynthesis, protein biosynthesis, response to virus, translational initiation |  | | | | | | | | | | | | | | | | | | | | | | | | | | | |
| 1202 | 212040\_at | TGOLN2 | -0.980760651 | 8.881151022 | -5.96823547 | 7.60E-006 | 0.000110254 | trans-golgi network protein 2 | NA |  | | | | | | | | | | | | | | | | | | | | | | | | | | | |
| 1203 | 201043\_s\_at | ANP32A | 0.803668994 | 8.479952034 | 5.96703527 | 7.62E-006 | 0.000110474 | acidic (leucine-rich) nuclear phosphoprotein 32 family, member A | intracellular signaling cascade, nucleocytoplasmic transport |  | | | | | | | | | | | | | | | | | | | | | | | | | | | |
| 1204 | 202371\_at | TCEAL4 | -2.569599382 | 6.327475664 | -5.9665054 | 7.63E-006 | 0.000110531 | transcription elongation factor A (SII)-like 4 | NA |  | | | | | | | | | | | | | | | | | | | | | | | | | | | |
| 1205 | 218545\_at | FLJ11088 | 1.44141335 | 5.858317739 | 5.96389162 | 7.67E-006 | 0.000111098 | GGA binding partner | NA |  | | | | | | | | | | | | | | | | | | | | | | | | | | | |
| 1206 | 205692\_s\_at | CD38 | -0.988159451 | 7.60686204 | -5.96312974 | 7.68E-006 | 0.000111213 | CD38 antigen (p45) | energy pathways, induction of apoptosis by extracellular signals, signal transduction |  | | | | | | | | | | | | | | | | | | | | | | | | | | | |
| 1207 | 213911\_s\_at | H2AFZ | 0.891551979 | 11.08876451 | 5.96148853 | 7.71E-006 | 0.000111543 | H2A histone family, member Z | chromosome organization and biogenesis (sensu Eukaryota), nucleosome assembly |  | | | | | | | | | | | | | | | | | | | | | | | | | | | |
| 1208 | 201613\_s\_at | AP1G2 | -0.860127526 | 7.753307426 | -5.95978076 | 7.74E-006 | 0.000111839 | adaptor-related protein complex 1, gamma 2 subunit | endocytosis, intracellular protein transport, protein complex assembly, vesicle-mediated transport |  | | | | | | | | | | | | | | | | | | | | | | | | | | | |
| 1209 | 212208\_at | THRAP2 | -1.226703417 | 9.065063292 | -5.95970095 | 7.74E-006 | 0.000111839 | thyroid hormone receptor associated protein 2 | NA |  | | | | | | | | | | | | | | | | | | | | | | | | | | | |
| 1210 | 201216\_at | C12orf8 | 0.798445321 | 10.86574177 | 5.95914778 | 7.75E-006 | 0.000111903 | chromosome 12 open reading frame 8 | intracellular protein transport, protein folding |  | | | | | | | | | | | | | | | | | | | | | | | | | | | |
| 1211 | 218577\_at | FLJ20331 | 1.40981088 | 6.821737199 | 5.95866731 | 7.76E-006 | 0.000111915 | hypothetical protein FLJ20331 | NA |  | | | | | | | | | | | | | | | | | | | | | | | | | | | |
| 1212 | 204500\_s\_at | AGTPBP1 | -1.407268305 | 7.428327507 | -5.9585085 | 7.76E-006 | 0.000111915 | ATP/GTP binding protein 1 | proteolysis and peptidolysis |  | | | | | | | | | | | | | | | | | | | | | | | | | | | |
| 1213 | 214696\_at | MGC14376 | -1.954290843 | 6.760580889 | -5.95680009 | 7.79E-006 | 0.000112261 | hypothetical protein MGC14376 | NA |  | | | | | | | | | | | | | | | | | | | | | | | | | | | |
| 1214 | 212492\_s\_at | JMJD2B | -0.834278965 | 7.816122996 | -5.95652176 | 7.80E-006 | 0.000112261 | jumonji domain containing 2B | NA |  | | | | | | | | | | | | | | | | | | | | | | | | | | | |
| 1215 | 200940\_s\_at | RERE | -0.89131294 | 8.255954859 | -5.95615442 | 7.80E-006 | 0.00011228 | arginine-glutamic acid dipeptide (RE) repeats | regulation of transcription, DNA-dependent |  | | | | | | | | | | | | | | | | | | | | | | | | | | | |
| 1216 | 45749\_at | FLJ13725 | -0.55226677 | 9.665019602 | -5.95550044 | 7.82E-006 | 0.000112369 | hypothetical protein FLJ13725 | NA |  | | | | | | | | | | | | | | | | | | | | | | | | | | | |
| 1217 | 210482\_x\_at | MAP2K5 | 0.637415823 | 6.896247107 | 5.9536365 | 7.85E-006 | 0.00011276 | mitogen-activated protein kinase kinase 5 | protein amino acid phosphorylation, signal transduction |  | | | | | | | | | | | | | | | | | | | | | | | | | | | |
| 1218 | 209004\_s\_at | FBXL5 | -1.827904838 | 8.393103329 | -5.95325333 | 7.85E-006 | 0.000112782 | F-box and leucine-rich repeat protein 5 | transport, ubiquitin cycle |  | | | | | | | | | | | | | | | | | | | | | | | | | | | |
| 1219 | 217968\_at | TSSC1 | 0.849412016 | 7.903883403 | 5.95255833 | 7.87E-006 | 0.000112882 | tumor suppressing subtransferable candidate 1 | NA |  | | | | | | | | | | | | | | | | | | | | | | | | | | | |
| 1220 | 205321\_at | EIF2S3 | 1.844034038 | 9.465519085 | 5.95187666 | 7.88E-006 | 0.000112979 | eukaryotic translation initiation factor 2, subunit 3 gamma, 52kDa | protein biosynthesis |  | | | | | | | | | | | | | | | | | | | | | | | | | | | |
| 1221 | 202808\_at | C10orf26 | -0.77557187 | 8.981268199 | -5.95028557 | 7.91E-006 | 0.000113304 | chromosome 10 open reading frame 26 | electron transport |  | | | | | | | | | | | | | | | | | | | | | | | | | | | |
| 1222 | 215434\_x\_at | LOC376745 | -0.722877292 | 8.71868948 | -5.94916889 | 7.93E-006 | 0.00011351 | AG1 | NA |  | | | | | | | | | | | | | | | | | | | | | | | | | | | |
| 1223 | 217478\_s\_at | HLA-DMA | -3.382043447 | 8.038451314 | -5.94750228 | 7.95E-006 | 0.000113782 | major histocompatibility complex, class II, DM alpha | antigen presentation, exogenous antigen, antigen processing, exogenous antigen via MHC class II, detection of pest, pathogen or parasite, immune response |  | | | | | | | | | | | | | | | | | | | | | | | | | | | |
| 1224 | 218160\_at | NDUFA8 | 1.566482043 | 8.793203387 | 5.94683371 | 7.97E-006 | 0.000113877 | NADH dehydrogenase (ubiquinone) 1 alpha subcomplex, 8, 19kDa | NA |  | | | | | | | | | | | | | | | | | | | | | | | | | | | |
| 1225 | 219460\_s\_at | FLJ20507 | -0.891971146 | 7.91321789 | -5.9434995 | 8.02E-006 | 0.00011447 | hypothetical protein FLJ20507 | NA |  | | | | | | | | | | | | | | | | | | | | | | | | | | | |
| 1226 | 212024\_x\_at | FLII | -0.491663365 | 9.049242461 | -5.94341057 | 8.03E-006 | 0.00011447 | flightless I homolog (Drosophila) | development, metabolism, muscle contraction |  | | | | | | | | | | | | | | | | | | | | | | | | | | | |
| 1227 | 207628\_s\_at | WBSCR22 | 1.669663357 | 8.78014809 | 5.94331761 | 8.03E-006 | 0.00011447 | Williams Beuren syndrome chromosome region 22 | NA |  | | | | | | | | | | | | | | | | | | | | | | | | | | | |
| 1228 | 205748\_s\_at | RNF126 | 1.061796927 | 7.861456214 | 5.94189623 | 8.05E-006 | 0.000114739 | ring finger protein 126 | protein ubiquitination |  | | | | | | | | | | | | | | | | | | | | | | | | | | | |
| 1229 | 211990\_at | HLA-DPA1 | -4.71720143 | 8.532382851 | -5.94167577 | 8.06E-006 | 0.000114739 | major histocompatibility complex, class II, DP alpha 1 | antigen presentation, exogenous antigen, antigen processing, exogenous antigen via MHC class II, immune response |  | | | | | | | | | | | | | | | | | | | | | | | | | | | |
| 1230 | 220890\_s\_at | DDX47 | 1.170332842 | 8.909579062 | 5.93662667 | 8.15E-006 | 0.000115878 | DEAD (Asp-Glu-Ala-Asp) box polypeptide 47 | RNA metabolism |  | | | | | | | | | | | | | | | | | | | | | | | | | | | |
| 1231 | 212176\_at | C6orf111 | -0.929099533 | 7.870251469 | -5.9362779 | 8.15E-006 | 0.000115893 | chromosome 6 open reading frame 111 | NA |  | | | | | | | | | | | | | | | | | | | | | | | | | | | |
| 1232 | 217960\_s\_at | TOMM22 | 1.34049444 | 8.345314237 | 5.93545196 | 8.17E-006 | 0.00011602 | translocase of outer mitochondrial membrane 22 homolog (yeast) | protein transport, protein-mitochondrial targeting |  | | | | | | | | | | | | | | | | | | | | | | | | | | | |
| 1233 | 218619\_s\_at | SUV39H1 | 0.637128398 | 8.014121266 | 5.93520385 | 8.17E-006 | 0.00011602 | suppressor of variegation 3-9 homolog 1 (Drosophila) | DNA replication and chromosome cycle, chromatin assembly or disassembly, chromatin modification |  | | | | | | | | | | | | | | | | | | | | | | | | | | | |
| 1234 | 215152\_at | MYB | 0.527905499 | 4.704138958 | 5.92732747 | 8.32E-006 | 0.000117857 | v-myb myeloblastosis viral oncogene homolog (avian) | cell growth and/or maintenance, regulation of transcription, DNA-dependent |  | | | | | | | | | | | | | | | | | | | | | | | | | | | |
| 1235 | 214722\_at | N2N | -1.610848893 | 8.440120937 | -5.92653743 | 8.33E-006 | 0.000117962 | similar to NOTCH2 protein | NA |  | | | | | | | | | | | | | | | | | | | | | | | | | | | |
| 1236 | 209324\_s\_at | RGS16 | 1.196800615 | 7.177753465 | 5.92569958 | 8.35E-006 | 0.000118106 | regulator of G-protein signalling 16 | regulation of G-protein coupled receptor protein signaling pathway, signal transduction, visual perception |  | | | | | | | | | | | | | | | | | | | | | | | | | | | |
| 1237 | 200038\_s\_at | RPL17 | 0.827327124 | 12.40472056 | 5.92480834 | 8.36E-006 | 0.000118264 | ribosomal protein L17 /// ribosomal protein L17 | positive regulation of I-kappaB kinase/NF-kappaB cascade, protein biosynthesis |  | | | | | | | | | | | | | | | | | | | | | | | | | | | |
| 1238 | 200662\_s\_at | TOMM20 | 1.925398395 | 9.185515119 | 5.92412775 | 8.38E-006 | 0.000118366 | translocase of outer mitochondrial membrane 20 homolog (yeast) | protein-mitochondrial targeting |  | | | | | | | | | | | | | | | | | | | | | | | | | | | |
| 1239 | 213007\_at | FLJ10719 | 1.384641344 | 7.403348552 | 5.92345871 | 8.39E-006 | 0.000118466 | hypothetical protein FLJ10719 | NA |  | | | | | | | | | | | | | | | | | | | | | | | | | | | |
| 1240 | 210213\_s\_at | ITGB4BP | 0.869352533 | 9.288739695 | 5.92063946 | 8.44E-006 | 0.000119071 | integrin beta 4 binding protein | metabolism, protein biosynthesis, translational initiation |  | | | | | | | | | | | | | | | | | | | | | | | | | | | |
| 1241 | 214867\_at | NDST2 | -0.568833762 | 7.154355367 | -5.92058 | 8.44E-006 | 0.000119071 | N-deacetylase/N-sulfotransferase (heparan glucosaminyl) 2 | energy pathways |  | | | | | | | | | | | | | | | | | | | | | | | | | | | |
| 1242 | 210041\_s\_at | PGM3 | 1.148549217 | 5.668575703 | 5.9197653 | 8.46E-006 | 0.000119211 | phosphoglucomutase 3 | carbohydrate metabolism, glucosamine metabolism |  | | | | | | | | | | | | | | | | | | | | | | | | | | | |
| 1243 | 212186\_at | ACACA | 1.102950895 | 7.97775758 | 5.91843606 | 8.48E-006 | 0.000119486 | acetyl-Coenzyme A carboxylase alpha | fatty acid biosynthesis, metabolism |  | | | | | | | | | | | | | | | | | | | | | | | | | | | |
| 1244 | 207877\_s\_at | NVL | 0.884572858 | 7.466453695 | 5.91464268 | 8.55E-006 | 0.000120267 | nuclear VCP-like | NA |  | | | | | | | | | | | | | | | | | | | | | | | | | | | |
| 1245 | 201246\_s\_at | OTUB1 | 0.401945329 | 7.803207752 | 5.91462985 | 8.55E-006 | 0.000120267 | OTU domain, ubiquitin aldehyde binding 1 | NA |  | | | | | | | | | | | | | | | | | | | | | | | | | | | |
| 1246 | 201106\_at | GPX4 | 0.979006076 | 9.890331033 | 5.91075265 | 8.63E-006 | 0.000121074 | glutathione peroxidase 4 (phospholipid hydroperoxidase) | development, phospholipid metabolism, response to oxidative stress |  | | | | | | | | | | | | | | | | | | | | | | | | | | | |
| 1247 | 218997\_at | PAF53 | 1.403753769 | 7.963930225 | 5.91029019 | 8.64E-006 | 0.000121121 | RNA polymerase I associated factor 53 | transcription |  | | | | | | | | | | | | | | | | | | | | | | | | | | | |
| 1248 | 218163\_at | MCTS1 | 2.041996848 | 8.870745487 | 5.90810823 | 8.68E-006 | 0.000121554 | malignant T cell amplified sequence 1 | positive regulation of cell proliferation, regulation of cell cycle |  | | | | | | | | | | | | | | | | | | | | | | | | | | | |
| 1249 | 209927\_s\_at | DKFZP547E1010 | 0.568060056 | 7.374917803 | 5.90730108 | 8.69E-006 | 0.000121695 | DKFZP547E1010 protein | NA |  | | | | | | | | | | | | | | | | | | | | | | | | | | | |
| 1250 | 218845\_at | DUSP22 | -0.919891808 | 8.446630026 | -5.9061978 | 8.71E-006 | 0.000121915 | dual specificity phosphatase 22 | apoptosis, cell proliferation, development, inactivation of MAPK, protein amino acid dephosphorylation |  | | | | | | | | | | | | | | | | | | | | | | | | | | | |
| 1251 | 211025\_x\_at | COX5B | 1.066906166 | 10.26833198 | 5.90424826 | 8.75E-006 | 0.000122289 | cytochrome c oxidase subunit Vb /// cytochrome c oxidase subunit Vb | electron transport, respiratory gaseous exchange |  | | | | | | | | | | | | | | | | | | | | | | | | | | | |
| 1252 | 208886\_at | H1F0 | -2.011999248 | 8.287277663 | -5.90274247 | 8.78E-006 | 0.00012262 | H1 histone family, member 0 | chromosome organization and biogenesis (sensu Eukaryota), nucleosome assembly |  | | | | | | | | | | | | | | | | | | | | | | | | | | | |
| 1253 | 219598\_s\_at | RWDD1 | 1.433043424 | 8.184863307 | 5.90174091 | 8.80E-006 | 0.000122815 | RWD domain containing 1 | NA |  | | | | | | | | | | | | | | | | | | | | | | | | | | | |
| 1254 | 203922\_s\_at | CYBB | -3.337244911 | 8.828585484 | -5.90039253 | 8.83E-006 | 0.000123105 | cytochrome b-245, beta polypeptide (chronic granulomatous disease) | antimicrobial humoral response (sensu Vertebrata), electron transport, energy pathways, inflammatory response |  | | | | | | | | | | | | | | | | | | | | | | | | | | | |
| 1255 | 200092\_s\_at | RPL37 | 0.736410454 | 12.40791014 | 5.8992252 | 8.85E-006 | 0.000123346 | ribosomal protein L37 /// ribosomal protein L37 | NA |  | | | | | | | | | | | | | | | | | | | | | | | | | | | |
| 1256 | 63825\_at | ABHD2 | -1.268997284 | 7.979260457 | -5.89772331 | 8.88E-006 | 0.000123679 | Abhydrolase domain containing 2 | NA |  | | | | | | | | | | | | | | | | | | | | | | | | | | | |
| 1257 | 219110\_at | NOLA1 | 1.326111659 | 7.727447012 | 5.89683221 | 8.90E-006 | 0.000123809 | nucleolar protein family A, member 1 (H/ACA small nucleolar RNPs) | rRNA processing |  | | | | | | | | | | | | | | | | | | | | | | | | | | | |
| 1258 | 213701\_at | DKFZp434N2030 | 1.204278907 | 6.577935948 | 5.89152665 | 9.00E-006 | 0.000124896 | Hypothetical protein DKFZp434N2030 | NA |  | | | | | | | | | | | | | | | | | | | | | | | | | | | |
| 1259 | 202396\_at | TCERG1 | 2.004720749 | 7.762223952 | 5.89147263 | 9.00E-006 | 0.000124896 | transcription elongation regulator 1 | regulation of transcription, DNA-dependent, transcription, transcription from Pol II promoter |  | | | | | | | | | | | | | | | | | | | | | | | | | | | |
| 1260 | 218586\_at | C20orf20 | 1.006110191 | 6.967117989 | 5.89132687 | 9.01E-006 | 0.000124896 | chromosome 20 open reading frame 20 | NA |  | | | | | | | | | | | | | | | | | | | | | | | | | | | |
| 1261 | 201634\_s\_at | CYB5-M | 1.314314052 | 5.899729973 | 5.88749523 | 9.08E-006 | 0.000125605 | cytochrome b5 outer mitochondrial membrane precursor | electron transport |  | | | | | | | | | | | | | | | | | | | | | | | | | | | |
| 1262 | 215023\_s\_at | PEX1 | 0.670020407 | 6.254901213 | 5.88742802 | 9.08E-006 | 0.000125605 | peroxisome biogenesis factor 1 | peroxisome organization and biogenesis, protein transport |  | | | | | | | | | | | | | | | | | | | | | | | | | | | |
| 1263 | 205323\_s\_at | MTF1 | -1.052827063 | 8.094627644 | -5.88372861 | 9.16E-006 | 0.000126464 | metal-regulatory transcription factor 1 | regulation of transcription from Pol II promoter, response to metal ion |  | | | | | | | | | | | | | | | | | | | | | | | | | | | |
| 1264 | 218099\_at | HT008 | -0.783099615 | 8.133623033 | -5.87687231 | 9.30E-006 | 0.000128082 | uncharacterized hypothalamus protein HT008 | phosphoenolpyruvate-dependent sugar phosphotransferase system |  | | | | | | | | | | | | | | | | | | | | | | | | | | | |
| 1265 | 204824\_at | ENDOG | 0.993129478 | 7.309317985 | 5.87588986 | 9.32E-006 | 0.000128205 | endonuclease G | DNA metabolism |  | | | | | | | | | | | | | | | | | | | | | | | | | | | |
| 1266 | 208995\_s\_at | PPIG | 0.708628278 | 6.228104638 | 5.87587883 | 9.32E-006 | 0.000128205 | peptidyl-prolyl isomerase G (cyclophilin G) | RNA splicing, protein folding |  | | | | | | | | | | | | | | | | | | | | | | | | | | | |
| 1267 | 201710\_at | MYBL2 | 1.013854193 | 8.032532556 | 5.87469188 | 9.34E-006 | 0.000128464 | v-myb myeloblastosis viral oncogene homolog (avian)-like 2 | anti-apoptosis, development, regulation of cell cycle, regulation of transcription, DNA-dependent, transcription from Pol II promoter |  | | | | | | | | | | | | | | | | | | | | | | | | | | | |
| 1268 | 202870\_s\_at | CDC20 | 2.279134883 | 7.937643595 | 5.87316391 | 9.38E-006 | 0.00012874 | CDC20 cell division cycle 20 homolog (S. cerevisiae) | cytokinesis, mitosis, regulation of cell cycle, ubiquitin cycle, ubiquitin-dependent protein catabolism |  | | | | | | | | | | | | | | | | | | | | | | | | | | | |
| 1269 | 40149\_at | SH2B | -0.437615642 | 7.403921765 | -5.87234393 | 9.39E-006 | 0.000128895 | SH2-B homolog | intracellular signaling cascade |  | | | | | | | | | | | | | | | | | | | | | | | | | | | |
| 1270 | 212947\_at | SLC9A8 | -0.810269817 | 6.913182023 | -5.869898 | 9.44E-006 | 0.000129516 | solute carrier family 9 (sodium/hydrogen exchanger), isoform 8 | NA |  | | | | | | | | | | | | | | | | | | | | | | | | | | | |
| 1271 | 210845\_s\_at | PLAUR | -2.005905294 | 9.196125597 | -5.86943008 | 9.45E-006 | 0.000129571 | plasminogen activator, urokinase receptor | blood coagulation, cell surface receptor linked signal transduction, chemotaxis |  | | | | | | | | | | | | | | | | | | | | | | | | | | | |
| 1272 | 200701\_at | NPC2 | -1.433854477 | 9.598875414 | -5.86806573 | 9.48E-006 | 0.000129883 | Niemann-Pick disease, type C2 | NA |  | | | | | | | | | | | | | | | | | | | | | | | | | | | |
| 1273 | 218639\_s\_at | MGC11349 | -0.896800064 | 7.312618672 | -5.86652843 | 9.52E-006 | 0.000130246 | hypothetical protein MGC11349 | NA |  | | | | | | | | | | | | | | | | | | | | | | | | | | | |
| 1274 | 216326\_s\_at | HDAC3 | 0.586881485 | 8.572464693 | 5.85989669 | 9.66E-006 | 0.000131933 | histone deacetylase 3 | anti-apoptosis, chromatin modification, histone deacetylation, regulation of cell cycle, regulation of transcription, DNA-dependent |  | | | | | | | | | | | | | | | | | | | | | | | | | | | |
| 1275 | 202467\_s\_at | TRIP15 | 1.31742928 | 9.555414593 | 5.85730217 | 9.71E-006 | 0.00013245 | thyroid receptor interacting protein 15 | NA |  | | | | | | | | | | | | | | | | | | | | | | | | | | | |
| 1276 | 211159\_s\_at | PPP2R5D | 0.7968213 | 7.032342398 | 5.85202984 | 9.83E-006 | 0.000133845 | protein phosphatase 2, regulatory subunit B (B56), delta isoform | neurogenesis, signal transduction |  | | | | | | | | | | | | | | | | | | | | | | | | | | | |
| 1277 | 211745\_x\_at | HBA1 | -4.454989732 | 11.55443375 | -5.85080131 | 9.85E-006 | 0.000134129 | hemoglobin, alpha 1 /// hemoglobin, alpha 1 | oxygen transport, transport |  | | | | | | | | | | | | | | | | | | | | | | | | | | | |
| 1278 | 200910\_at | CCT3 | 2.239395048 | 9.262652565 | 5.85021487 | 9.87E-006 | 0.000134221 | chaperonin containing TCP1, subunit 3 (gamma) | protein folding |  | | | | | | | | | | | | | | | | | | | | | | | | | | | |
| 1279 | 205768\_s\_at | SLC27A2 | 2.071138347 | 7.457389583 | 5.84876033 | 9.90E-006 | 0.000134573 | solute carrier family 27 (fatty acid transporter), member 2 | metabolism, very-long-chain fatty acid metabolism |  | | | | | | | | | | | | | | | | | | | | | | | | | | | |
| 1280 | 202276\_at | SHFM1 | 1.018264484 | 9.028423739 | 5.84395614 | 1.00E-005 | 0.000135933 | split hand/foot malformation (ectrodactyly) type 1 | embryonic limb morphogenesis, proteolysis and peptidolysis |  | | | | | | | | | | | | | | | | | | | | | | | | | | | |
| 1281 | 213958\_at | CD6 | -0.613043811 | 8.125010628 | -5.83856477 | 1.01E-005 | 0.000137486 | CD6 antigen /// CD6 antigen | cell adhesion, immune response |  | | | | | | | | | | | | | | | | | | | | | | | | | | | |
| 1282 | 221046\_s\_at | HSPC135 | 0.837634575 | 7.491606797 | 5.83742278 | 1.02E-005 | 0.000137751 | HSPC135 protein | NA |  | | | | | | | | | | | | | | | | | | | | | | | | | | | |
| 1283 | 211727\_s\_at | COX11 | 1.550968174 | 7.726416176 | 5.83591066 | 1.02E-005 | 0.000138046 | COX11 homolog, cytochrome c oxidase assembly protein (yeast) /// COX11 homolog, cytochrome c oxidase assembly protein (yeast) | cytochrome c oxidase biogenesis, respiratory gaseous exchange |  | | | | | | | | | | | | | | | | | | | | | | | | | | | |
| 1284 | 203094\_at | MAD2L1BP | 0.766975566 | 7.668820533 | 5.83488837 | 1.02E-005 | 0.000138276 | MAD2L1 binding protein | regulation of exit from mitosis |  | | | | | | | | | | | | | | | | | | | | | | | | | | | |
| 1285 | 210010\_s\_at | SLC25A1 | 1.669221055 | 7.91870547 | 5.83407053 | 1.02E-005 | 0.000138367 | solute carrier family 25 (mitochondrial carrier; citrate transporter), member 1 | mitochondrial citrate transport, transport |  | | | | | | | | | | | | | | | | | | | | | | | | | | | |
| 1286 | 221538\_s\_at | PLXNA1 | 1.678485843 | 6.457072572 | 5.83157928 | 1.03E-005 | 0.000139038 | plexin A1 | development |  | | | | | | | | | | | | | | | | | | | | | | | | | | | |
| 1287 | 217883\_at | C2orf25 | 0.907748354 | 10.43378817 | 5.83131867 | 1.03E-005 | 0.000139038 | chromosome 2 open reading frame 25 | NA |  | | | | | | | | | | | | | | | | | | | | | | | | | | | |
| 1288 | 218848\_at | MGC2655 | 0.842184385 | 7.252052885 | 5.83055534 | 1.03E-005 | 0.000139083 | hypothetical protein MGC2655 | NA |  | | | | | | | | | | | | | | | | | | | | | | | | | | | |
| 1289 | 220934\_s\_at | MGC3196 | 0.646607507 | 8.351865769 | 5.82610337 | 1.04E-005 | 0.000140318 | hypothetical protein MGC3196 | NA |  | | | | | | | | | | | | | | | | | | | | | | | | | | | |
| 1290 | 219068\_x\_at | ATAD3A | 0.569814194 | 7.907807213 | 5.82351001 | 1.05E-005 | 0.000140958 | ATPase family, AAA domain containing 3A | NA |  | | | | | | | | | | | | | | | | | | | | | | | | | | | |
| 1291 | 204089\_x\_at | MAP3K4 | 0.848428526 | 8.089744544 | 5.82253957 | 1.05E-005 | 0.000141177 | mitogen-activated protein kinase kinase kinase 4 | activation of MAPKK, protein amino acid phosphorylation, response to stress |  | | | | | | | | | | | | | | | | | | | | | | | | | | | |
| 1292 | 212896\_at | KIAA0052 | 1.35182292 | 8.310781956 | 5.8219618 | 1.05E-005 | 0.000141223 | KIAA0052 | NA |  | | | | | | | | | | | | | | | | | | | | | | | | | | | |
| 1293 | 218195\_at | C6orf211 | 1.636407454 | 5.743419146 | 5.82172968 | 1.05E-005 | 0.000141223 | chromosome 6 open reading frame 211 | NA |  | | | | | | | | | | | | | | | | | | | | | | | | | | | |
| 1294 | 203667\_at | TBCA | 0.957721586 | 9.951636082 | 5.82157602 | 1.05E-005 | 0.000141223 | tubulin-specific chaperone a | beta-tubulin folding, chaperonin-mediated tubulin folding, post-chaperonin tubulin folding pathway, protein folding |  | | | | | | | | | | | | | | | | | | | | | | | | | | | |
| 1295 | 204959\_at | MNDA | -4.714022344 | 9.771047115 | -5.81568951 | 1.07E-005 | 0.000142997 | myeloid cell nuclear differentiation antigen /// myeloid cell nuclear differentiation antigen | cellular defense response, regulation of transcription, DNA-dependent |  | | | | | | | | | | | | | | | | | | | | | | | | | | | |
| 1296 | 209665\_at | CYB561D2 | -0.528805161 | 8.441115129 | -5.81390084 | 1.07E-005 | 0.000143481 | cytochrome b-561 domain containing 2 | cyclin catabolism, electron transport, regulation of exit from mitosis, ubiquitin-dependent protein catabolism |  | | | | | | | | | | | | | | | | | | | | | | | | | | | |
| 1297 | 201937\_s\_at | DNPEP | 0.829836602 | 8.152814363 | 5.8130489 | 1.07E-005 | 0.000143666 | aspartyl aminopeptidase | peptide metabolism, proteolysis and peptidolysis |  | | | | | | | | | | | | | | | | | | | | | | | | | | | |
| 1298 | 204093\_at | CCNH | 1.119091566 | 8.682236389 | 5.8121721 | 1.07E-005 | 0.000143773 | cyclin H | DNA repair, cell cycle, regulation of cyclin dependent protein kinase activity, regulation of transcription, DNA-dependent |  | | | | | | | | | | | | | | | | | | | | | | | | | | | |
| 1299 | 210759\_s\_at | PSMA1 | 1.109894321 | 10.46028674 | 5.81179425 | 1.07E-005 | 0.000143807 | proteasome (prosome, macropain) subunit, alpha type, 1 | ubiquitin-dependent protein catabolism |  | | | | | | | | | | | | | | | | | | | | | | | | | | | |
| 1300 | 208822\_s\_at | DAP3 | 1.469274326 | 9.614996267 | 5.81104787 | 1.08E-005 | 0.000143895 | death associated protein 3 | apoptosis, induction of apoptosis by extracellular signals |  | | | | | | | | | | | | | | | | | | | | | | | | | | | |
| 1301 | 213318\_s\_at | BAT3 | 0.839252518 | 10.03205187 | 5.81097916 | 1.08E-005 | 0.000143895 | HLA-B associated transcript 3 | NA |  | | | | | | | | | | | | | | | | | | | | | | | | | | | |
| 1302 | 216253\_s\_at | PARVB | 1.283189743 | 6.389200212 | 5.80945287 | 1.08E-005 | 0.000144297 | parvin, beta | cell adhesion |  | | | | | | | | | | | | | | | | | | | | | | | | | | | |
| 1303 | 218902\_at | NOTCH1 | -0.890146652 | 7.275155258 | -5.80853519 | 1.08E-005 | 0.000144428 | Notch homolog 1, translocation-associated (Drosophila) | cell differentiation, immune response, regulation of development, regulation of transcription, DNA-dependent |  | | | | | | | | | | | | | | | | | | | | | | | | | | | |
| 1304 | 203721\_s\_at | CGI-48 | 1.432628726 | 8.586307545 | 5.80850715 | 1.08E-005 | 0.000144428 | CGI-48 protein | NA |  | | | | | | | | | | | | | | | | | | | | | | | | | | | |
| 1305 | 218343\_s\_at | GTF3C3 | 1.721238441 | 7.763740388 | 5.80639219 | 1.09E-005 | 0.000144988 | general transcription factor IIIC, polypeptide 3, 102kDa | transcription, transcription from Pol III promoter |  | | | | | | | | | | | | | | | | | | | | | | | | | | | |
| 1306 | 201979\_s\_at | PPP5C | 1.005550794 | 7.559188067 | 5.80588116 | 1.09E-005 | 0.000144988 | protein phosphatase 5, catalytic subunit | mitosis, positive regulation of I-kappaB kinase/NF-kappaB cascade, protein amino acid dephosphorylation, transcription |  | | | | | | | | | | | | | | | | | | | | | | | | | | | |
| 1307 | 205133\_s\_at | HSPE1 | 2.298564866 | 8.446104299 | 5.80568837 | 1.09E-005 | 0.000144988 | heat shock 10kDa protein 1 (chaperonin 10) | NA |  | | | | | | | | | | | | | | | | | | | | | | | | | | | |
| 1308 | 203554\_x\_at | PTTG1 | 1.354854882 | 9.88697125 | 5.80202522 | 1.10E-005 | 0.000146053 | pituitary tumor-transforming 1 | DNA metabolism, DNA repair, DNA replication and chromosome cycle, cell growth and/or maintenance, chromosome segregation, mitosis, spermatogenesis, transcription from Pol II promoter |  | | | | | | | | | | | | | | | | | | | | | | | | | | | |
| 1309 | 211707\_s\_at | IQCB1 | 1.05117559 | 7.720863789 | 5.80186264 | 1.10E-005 | 0.000146053 | IQ motif containing B1 /// IQ motif containing B1 | NA |  | | | | | | | | | | | | | | | | | | | | | | | | | | | |
| 1310 | 211042\_x\_at | MCAM | 0.888338846 | 7.629786978 | 5.80047401 | 1.10E-005 | 0.000146417 | melanoma cell adhesion molecule /// melanoma cell adhesion molecule | cell adhesion, morphogenesis |  | | | | | | | | | | | | | | | | | | | | | | | | | | | |
| 1311 | 202379\_s\_at | NKTR | -1.406921771 | 9.603605693 | -5.7957399 | 1.11E-005 | 0.00014788 | natural killer-tumor recognition sequence | protein folding |  | | | | | | | | | | | | | | | | | | | | | | | | | | | |
| 1312 | 201263\_at | TARS | 2.297381631 | 9.1738726 | 5.79132512 | 1.12E-005 | 0.000149074 | threonyl-tRNA synthetase | protein biosynthesis, threonyl-tRNA aminoacylation |  | | | | | | | | | | | | | | | | | | | | | | | | | | | |
| 1313 | 201965\_s\_at | KIAA0625 | -0.829019057 | 8.353822883 | -5.79049958 | 1.13E-005 | 0.000149259 | senataxin | cell death |  | | | | | | | | | | | | | | | | | | | | | | | | | | | |
| 1314 | 202911\_at | MSH6 | 1.476381039 | 8.573124689 | 5.78679761 | 1.14E-005 | 0.000150315 | mutS homolog 6 (E. coli) | mismatch repair |  | | | | | | | | | | | | | | | | | | | | | | | | | | | |
| 1315 | 204246\_s\_at | DCTN3 | 0.684765486 | 9.384174172 | 5.78462291 | 1.14E-005 | 0.000150955 | dynactin 3 (p22) | cytokinesis, mitosis |  | | | | | | | | | | | | | | | | | | | | | | | | | | | |
| 1316 | 201687\_s\_at | API5 | 1.002427672 | 7.567929675 | 5.78380171 | 1.14E-005 | 0.000151142 | apoptosis inhibitor 5 | anti-apoptosis, transport |  | | | | | | | | | | | | | | | | | | | | | | | | | | | |
| 1317 | 212587\_s\_at | PTPRC | -2.06094271 | 9.348715391 | -5.780037 | 1.15E-005 | 0.000152256 | protein tyrosine phosphatase, receptor type, C | cell surface receptor linked signal transduction, protein amino acid dephosphorylation |  | | | | | | | | | | | | | | | | | | | | | | | | | | | |
| 1318 | 204018\_x\_at | HBA1 /// HBA2 | -4.176442745 | 11.67145211 | -5.77997012 | 1.15E-005 | 0.000152256 | hemoglobin, alpha 1 /// hemoglobin, alpha 1 /// hemoglobin, alpha 2 /// hemoglobin, alpha 2 | oxygen transport, transport |  | | | | | | | | | | | | | | | | | | | | | | | | | | | |
| 1319 | 213229\_at | DICER1 | -1.182839341 | 8.44151513 | -5.77942561 | 1.15E-005 | 0.000152351 | Dicer1, Dcr-1 homolog (Drosophila) | RNA interference, targeting of mRNA for destruction, RNA processing |  | | | | | | | | | | | | | | | | | | | | | | | | | | | |
| 1320 | 217457\_s\_at | RAP1GDS1 | 0.876646575 | 8.189546011 | 5.77787007 | 1.16E-005 | 0.000152714 | RAP1, GTP-GDP dissociation stimulator 1 | NA |  | | | | | | | | | | | | | | | | | | | | | | | | | | | |
| 1321 | 202682\_s\_at | USP4 | -1.041473503 | 8.634757501 | -5.77782298 | 1.16E-005 | 0.000152714 | ubiquitin specific protease 4 (proto-oncogene) | cell growth and/or maintenance, ubiquitin cycle, ubiquitin-dependent protein catabolism |  | | | | | | | | | | | | | | | | | | | | | | | | | | | |
| 1322 | 218097\_s\_at | CUEDC2 | 0.681733893 | 8.968399136 | 5.77706318 | 1.16E-005 | 0.000152883 | CUE domain containing 2 | NA |  | | | | | | | | | | | | | | | | | | | | | | | | | | | |
| 1323 | 218890\_x\_at | MRPL35 | 1.384172036 | 8.055414429 | 5.76905028 | 1.18E-005 | 0.000155546 | mitochondrial ribosomal protein L35 | NA |  | | | | | | | | | | | | | | | | | | | | | | | | | | | |
| 1324 | 201459\_at | RUVBL2 | 1.501823124 | 9.463486211 | 5.76559606 | 1.19E-005 | 0.000156655 | RuvB-like 2 (E. coli) | DNA recombination, DNA repair, protein complex assembly, multichaperone pathway, transcription |  | | | | | | | | | | | | | | | | | | | | | | | | | | | |
| 1325 | 210317\_s\_at | YWHAE | 2.002136022 | 8.19486056 | 5.76159382 | 1.20E-005 | 0.000157966 | tyrosine 3-monooxygenase/tryptophan 5-monooxygenase activation protein, epsilon polypeptide | NA |  | | | | | | | | | | | | | | | | | | | | | | | | | | | |
| 1326 | 209907\_s\_at | ITSN2 | -1.101357656 | 8.092555894 | -5.76042192 | 1.20E-005 | 0.000158286 | intersectin 2 | endocytosis |  | | | | | | | | | | | | | | | | | | | | | | | | | | | |
| 1327 | 221804\_s\_at | FAM45A /// FAM45B | -1.706268023 | 9.59422489 | -5.75769978 | 1.21E-005 | 0.000158968 | family with sequence similarity 45, member A /// family with sequence similarity 45, member B | NA |  | | | | | | | | | | | | | | | | | | | | | | | | | | | |
| 1328 | 218216\_x\_at | ARL6IP4 | 0.619990511 | 9.352531986 | 5.75682826 | 1.21E-005 | 0.000159184 | ADP-ribosylation-like factor 6 interacting protein 4 | RNA splicing |  | | | | | | | | | | | | | | | | | | | | | | | | | | | |
| 1329 | 217842\_at | LUC7L2 | 0.96597595 | 6.514944952 | 5.75589752 | 1.22E-005 | 0.000159326 | LUC7-like 2 (S. cerevisiae) | NA |  | | | | | | | | | | | | | | | | | | | | | | | | | | | |
| 1330 | 202138\_x\_at | JTV1 | 1.603174661 | 9.328757925 | 5.75129959 | 1.23E-005 | 0.00016078 | JTV1 gene | protein biosynthesis |  | | | | | | | | | | | | | | | | | | | | | | | | | | | |
| 1331 | 218828\_at | PLSCR3 | -0.570373567 | 8.47756042 | -5.74880463 | 1.24E-005 | 0.000161487 | phospholipid scramblase 3 | phospholipid scrambling |  | | | | | | | | | | | | | | | | | | | | | | | | | | | |
| 1332 | 209439\_s\_at | PHKA2 | -0.779170851 | 8.104187176 | -5.74668708 | 1.24E-005 | 0.000162069 | phosphorylase kinase, alpha 2 (liver) | carbohydrate metabolism, energy pathways, glycogen metabolism, protein modification |  | | | | | | | | | | | | | | | | | | | | | | | | | | | |
| 1333 | 220960\_x\_at | RPL22 | 0.64650838 | 12.69392598 | 5.74666683 | 1.24E-005 | 0.000162069 | ribosomal protein L22 | protein biosynthesis |  | | | | | | | | | | | | | | | | | | | | | | | | | | | |
| 1334 | 218133\_s\_at | NIF3L1 | 1.409097451 | 8.371386044 | 5.74399787 | 1.25E-005 | 0.000162656 | NIF3 NGG1 interacting factor 3-like 1 (S. pombe) | NA |  | | | | | | | | | | | | | | | | | | | | | | | | | | | |
| 1335 | 212945\_s\_at | MGA | 0.754240315 | 6.703769935 | 5.74276123 | 1.25E-005 | 0.000162927 | MAX gene associated | NA |  | | | | | | | | | | | | | | | | | | | | | | | | | | | |
| 1336 | 203467\_at | PMM1 | 1.266130553 | 6.842257707 | 5.739576 | 1.26E-005 | 0.000163981 | phosphomannomutase 1 | mannose biosynthesis, metabolism |  | | | | | | | | | | | | | | | | | | | | | | | | | | | |
| 1337 | 205055\_at | ITGAE | -0.954010947 | 8.647453827 | -5.73837636 | 1.27E-005 | 0.000164325 | integrin, alpha E (antigen CD103, human mucosal lymphocyte antigen 1; alpha polypeptide) | cell-matrix adhesion, integrin-mediated signaling pathway, leukocyte cell adhesion |  | | | | | | | | | | | | | | | | | | | | | | | | | | | |
| 1338 | 222077\_s\_at | RACGAP1 | 1.499771094 | 7.51881232 | 5.73627035 | 1.27E-005 | 0.000165003 | Rac GTPase activating protein 1 | electron transport, intracellular signaling cascade |  | | | | | | | | | | | | | | | | | | | | | | | | | | | |
| 1339 | 204924\_at | TLR2 | -1.838255405 | 8.407647889 | -5.73302942 | 1.28E-005 | 0.000166105 | toll-like receptor 2 | immune response, inflammatory response, signal transduction |  | | | | | | | | | | | | | | | | | | | | | | | | | | | |
| 1340 | 202854\_at | HPRT1 | 1.149540137 | 9.350724242 | 5.73050759 | 1.29E-005 | 0.000166857 | hypoxanthine phosphoribosyltransferase 1 (Lesch-Nyhan syndrome) | behavior, nucleoside metabolism, purine ribonucleoside salvage |  | | | | | | | | | | | | | | | | | | | | | | | | | | | |
| 1341 | 222037\_at | MCM4 | 1.595215465 | 7.42909229 | 5.73048503 | 1.29E-005 | 0.000166857 | MCM4 minichromosome maintenance deficient 4 (S. cerevisiae) | DNA replication, DNA replication initiation, regulation of transcription, DNA-dependent |  | | | | | | | | | | | | | | | | | | | | | | | | | | | |
| 1342 | 203013\_at | HSGT1 | 1.102659518 | 7.69349524 | 5.73006471 | 1.29E-005 | 0.000166882 | suppressor of S. cerevisiae gcr2 | regulation of glycolysis, transcription from Pol II promoter |  | | | | | | | | | | | | | | | | | | | | | | | | | | | |
| 1343 | 203145\_at | SPAG5 | 0.981942181 | 7.643650533 | 5.72739477 | 1.30E-005 | 0.000167622 | sperm associated antigen 5 | cell cycle, cytokinesis, mitosis |  | | | | | | | | | | | | | | | | | | | | | | | | | | | |
| 1344 | 200947\_s\_at | GLUD1 | 1.076156245 | 8.903797836 | 5.72564949 | 1.30E-005 | 0.000168081 | glutamate dehydrogenase 1 | amino acid metabolism, glutamate catabolism |  | | | | | | | | | | | | | | | | | | | | | | | | | | | |
| 1345 | 209759\_s\_at | DCI | 1.052068963 | 8.085121155 | 5.72538873 | 1.30E-005 | 0.000168081 | dodecenoyl-Coenzyme A delta isomerase (3,2 trans-enoyl-Coenzyme A isomerase) | fatty acid metabolism, metabolism |  | | | | | | | | | | | | | | | | | | | | | | | | | | | |
| 1346 | 203799\_at | DCL-1 | -2.428201462 | 8.622109624 | -5.72460033 | 1.31E-005 | 0.00016828 | type I transmembrane C-type lectin receptor DCL-1 | NA |  | | | | | | | | | | | | | | | | | | | | | | | | | | | |
| 1347 | 65635\_at | FLJ21865 | -0.502054517 | 8.637949964 | -5.7241464 | 1.31E-005 | 0.000168353 | endo-beta-N-acetylglucosaminidase | carbohydrate metabolism |  | | | | | | | | | | | | | | | | | | | | | | | | | | | |
| 1348 | 212995\_x\_at | FLJ14346 | 0.965259877 | 9.744426677 | 5.72256612 | 1.31E-005 | 0.000168851 | hypothetical protein FLJ14346 | NA |  | | | | | | | | | | | | | | | | | | | | | | | | | | | |
| 1349 | 212708\_at | LOC339287 | -0.868061286 | 9.92338902 | -5.72213511 | 1.31E-005 | 0.000168915 | hypothetical protein LOC339287 | NA |  | | | | | | | | | | | | | | | | | | | | | | | | | | | |
| 1350 | 210094\_s\_at | PARD3 | 0.618325719 | 6.378187565 | 5.7214217 | 1.31E-005 | 0.000169087 | par-3 partitioning defective 3 homolog (C. elegans) | axonogenesis, cell cycle, cytokinesis, establishment and/or maintenance of cell polarity, protein kinase C activation |  | | | | | | | | | | | | | | | | | | | | | | | | | | | |
| 1351 | 202715\_at | CAD | 1.418182408 | 8.320448271 | 5.7201069 | 1.32E-005 | 0.000169486 | carbamoyl-phosphate synthetase 2, aspartate transcarbamylase, and dihydroorotase | de novo' pyrimidine base biosynthesis, amino acid metabolism, arginine biosynthesis, biosynthesis, nitrogen metabolism, pyrimidine nucleotide biosynthesis |  | | | | | | | | | | | | | | | | | | | | | | | | | | | |
| 1352 | 200076\_s\_at | MGC2749 | 0.755902863 | 8.650037324 | 5.71872588 | 1.32E-005 | 0.000169912 | hypothetical protein MGC2749 /// hypothetical protein MGC2749 | NA |  | | | | | | | | | | | | | | | | | | | | | | | | | | | |
| 1353 | 217963\_s\_at | NGFRAP1 | -1.727506818 | 8.193518568 | -5.71833732 | 1.32E-005 | 0.000169961 | nerve growth factor receptor (TNFRSF16) associated protein 1 | apoptosis, development |  | | | | | | | | | | | | | | | | | | | | | | | | | | | |
| 1354 | 212872\_s\_at | USP49 | 0.599103593 | 7.159783122 | 5.71802713 | 1.32E-005 | 0.000169981 | ubiquitin specific protease 49 | regulation of transcription, DNA-dependent, transcription |  | | | | | | | | | | | | | | | | | | | | | | | | | | | |
| 1355 | 205945\_at | IL6R | -2.797654464 | 7.620591469 | -5.71386631 | 1.34E-005 | 0.00017147 | interleukin 6 receptor /// interleukin 6 receptor | cell proliferation, cell surface receptor linked signal transduction, development, immune response |  | | | | | | | | | | | | | | | | | | | | | | | | | | | |
| 1356 | 219588\_s\_at | MTB | 1.264859575 | 7.598853266 | 5.70898223 | 1.35E-005 | 0.000172961 | more than blood homolog | NA |  | | | | | | | | | | | | | | | | | | | | | | | | | | | |
| 1357 | 206831\_s\_at | ARSD | -0.848354751 | 6.550125458 | -5.70896521 | 1.35E-005 | 0.000172961 | arylsulfatase D | metabolism |  | | | | | | | | | | | | | | | | | | | | | | | | | | | |
| 1358 | 212180\_at | CRKL | 1.130026942 | 8.510851622 | 5.70557353 | 1.36E-005 | 0.000174018 | v-crk sarcoma virus CT10 oncogene homolog (avian)-like | JNK cascade, Ras protein signal transduction, cell motility, intracellular signaling cascade, protein amino acid phosphorylation |  | | | | | | | | | | | | | | | | | | | | | | | | | | | |
| 1359 | 200812\_at | CCT7 | 1.824132554 | 9.823746638 | 5.70535239 | 1.36E-005 | 0.000174018 | chaperonin containing TCP1, subunit 7 (eta) | protein folding, regulation of cell cycle |  | | | | | | | | | | | | | | | | | | | | | | | | | | | |
| 1360 | 201049\_s\_at | RPS18 | 0.662419977 | 13.54369169 | 5.70520979 | 1.36E-005 | 0.000174018 | ribosomal protein S18 | protein biosynthesis |  | | | | | | | | | | | | | | | | | | | | | | | | | | | |
| 1361 | 208074\_s\_at | AP2S1 | 1.303402177 | 9.389042614 | 5.70135685 | 1.37E-005 | 0.000175389 | adaptor-related protein complex 2, sigma 1 subunit | clathrin cage assembly, intracellular protein transport, regulation of endocytosis, transport |  | | | | | | | | | | | | | | | | | | | | | | | | | | | |
| 1362 | 214359\_s\_at | HSPCB | 2.223085884 | 10.43623046 | 5.7011878 | 1.38E-005 | 0.000175389 | heat shock 90kDa protein 1, beta | positive regulation of nitric oxide biosynthesis, protein folding, response to unfolded protein |  | | | | | | | | | | | | | | | | | | | | | | | | | | | |
| 1363 | 219581\_at | SEN2L | 0.681295886 | 5.504568348 | 5.69851032 | 1.38E-005 | 0.000176342 | likely homolog of yeast SEN2 | mRNA processing, tRNA splicing |  | | | | | | | | | | | | | | | | | | | | | | | | | | | |
| 1364 | 201899\_s\_at | UBE2A | 0.968031914 | 8.450996686 | 5.69724256 | 1.39E-005 | 0.000176742 | ubiquitin-conjugating enzyme E2A (RAD6 homolog) | postreplication repair, ubiquitin cycle, ubiquitin-dependent protein catabolism |  | | | | | | | | | | | | | | | | | | | | | | | | | | | |
| 1365 | 205180\_s\_at | ADAM8 | -1.749387672 | 8.906806881 | -5.69531291 | 1.39E-005 | 0.000177371 | a disintegrin and metalloproteinase domain 8 /// a disintegrin and metalloproteinase domain 8 | proteolysis and peptidolysis |  | | | | | | | | | | | | | | | | | | | | | | | | | | | |
| 1366 | 201588\_at | TXNL1 | 1.318849932 | 10.02954905 | 5.69514174 | 1.39E-005 | 0.000177371 | thioredoxin-like 1 | apoptosis, electron transport, signal transduction |  | | | | | | | | | | | | | | | | | | | | | | | | | | | |
| 1367 | 202459\_s\_at | LPIN2 | -1.040650032 | 8.087010243 | -5.69379728 | 1.40E-005 | 0.000177804 | lipin 2 | NA |  | | | | | | | | | | | | | | | | | | | | | | | | | | | |
| 1368 | 218394\_at | FLJ22386 | -1.260246767 | 8.506585986 | -5.69238459 | 1.40E-005 | 0.000178265 | leucine zipper domain protein | NA |  | | | | | | | | | | | | | | | | | | | | | | | | | | | |
| 1369 | 209473\_at | ENTPD1 | -1.755260628 | 8.475207207 | -5.69161653 | 1.41E-005 | 0.000178469 | ectonucleoside triphosphate diphosphohydrolase 1 | antimicrobial humoral response (sensu Vertebrata), blood coagulation, cell adhesion, cell-cell signaling |  | | | | | | | | | | | | | | | | | | | | | | | | | | | |
| 1370 | 205842\_s\_at | JAK2 | -1.475227197 | 7.51827966 | -5.69028466 | 1.41E-005 | 0.000178798 | Janus kinase 2 (a protein tyrosine kinase) | JAK-STAT cascade, cell growth and/or maintenance, cell motility, intracellular signaling cascade, mesoderm development, protein amino acid phosphorylation, regulation of cell cycle |  | | | | | | | | | | | | | | | | | | | | | | | | | | | |
| 1371 | 213596\_at | CASP4 | -0.654715917 | 5.517370397 | -5.68746777 | 1.42E-005 | 0.000179781 | caspase 4, apoptosis-related cysteine protease | induction of apoptosis, proteolysis and peptidolysis, regulation of apoptosis |  | | | | | | | | | | | | | | | | | | | | | | | | | | | |
| 1372 | 204860\_s\_at | BIRC1 | -1.928672057 | 6.861239777 | -5.68732379 | 1.42E-005 | 0.000179781 | baculoviral IAP repeat-containing 1 | anti-apoptosis, apoptosis, neurogenesis |  | | | | | | | | | | | | | | | | | | | | | | | | | | | |
| 1373 | 210053\_at | TAF5 | 1.246948923 | 6.83527127 | 5.68607882 | 1.42E-005 | 0.000180078 | TAF5 RNA polymerase II, TATA box binding protein (TBP)-associated factor, 100kDa | regulation of transcription, DNA-dependent |  | | | | | | | | | | | | | | | | | | | | | | | | | | | |
| 1374 | 216306\_x\_at | PTBP1 | 0.979236303 | 10.74236498 | 5.68435304 | 1.43E-005 | 0.000180604 | polypyrimidine tract binding protein 1 | RNA splicing, nuclear mRNA splicing, via spliceosome |  | | | | | | | | | | | | | | | | | | | | | | | | | | | |
| 1375 | 218732\_at | Bit1 | 0.973685402 | 8.467634978 | 5.68223954 | 1.43E-005 | 0.000181219 | Bcl-2 inhibitor of transcription | apoptosis |  | | | | | | | | | | | | | | | | | | | | | | | | | | | |
| 1376 | 208393\_s\_at | RAD50 | 1.26263755 | 7.205934518 | 5.67837814 | 1.45E-005 | 0.000182689 | RAD50 homolog (S. cerevisiae) | DNA repair, double-strand break repair, meiotic recombination, regulation of mitotic recombination, telomerase-dependent telomere maintenance, transport |  | | | | | | | | | | | | | | | | | | | | | | | | | | | |
| 1377 | 209128\_s\_at | SART3 | 0.776535972 | 8.049583807 | 5.67739273 | 1.45E-005 | 0.000182885 | squamous cell carcinoma antigen recognised by T cells 3 | RNA processing |  | | | | | | | | | | | | | | | | | | | | | | | | | | | |
| 1378 | 205512\_s\_at | PDCD8 | 1.504057406 | 8.024338765 | 5.67634422 | 1.45E-005 | 0.000182908 | programmed cell death 8 (apoptosis-inducing factor) | DNA damage response, signal transduction resulting in induction of apoptosis, DNA fragmentation during apoptosis, apoptosis, electron transport |  | | | | | | | | | | | | | | | | | | | | | | | | | | | |
| 1379 | 205068\_s\_at | ARHGAP26 | -1.492264056 | 8.019716888 | -5.67622939 | 1.45E-005 | 0.000182908 | Rho GTPase activating protein 26 | actin cytoskeleton organization and biogenesis, cell growth and/or maintenance, neurogenesis |  | | | | | | | | | | | | | | | | | | | | | | | | | | | |
| 1380 | 207122\_x\_at | SULT1A2 | -1.043349306 | 8.755583517 | -5.67614061 | 1.45E-005 | 0.000182908 | sulfotransferase family, cytosolic, 1A, phenol-preferring, member 2 | amine biosynthesis, catecholamine metabolism, steroid metabolism |  | | | | | | | | | | | | | | | | | | | | | | | | | | | |
| 1381 | 210609\_s\_at | TP53I3 | -1.087151159 | 7.798232529 | -5.67388188 | 1.46E-005 | 0.000183704 | tumor protein p53 inducible protein 3 | induction of apoptosis by oxidative stress |  | | | | | | | | | | | | | | | | | | | | | | | | | | | |
| 1382 | 218886\_at | PAK1IP1 | 1.395469243 | 6.465950021 | 5.67338776 | 1.46E-005 | 0.000183803 | PAK1 interacting protein 1 | NA |  | | | | | | | | | | | | | | | | | | | | | | | | | | | |
| 1383 | 200032\_s\_at | RPL9 | 0.629625655 | 12.73966868 | 5.66955112 | 1.48E-005 | 0.00018518 | ribosomal protein L9 /// ribosomal protein L9 | protein biosynthesis |  | | | | | | | | | | | | | | | | | | | | | | | | | | | |
| 1384 | 211978\_x\_at | PPIA | 0.709037843 | 13.07931337 | 5.66917796 | 1.48E-005 | 0.000185184 | peptidylprolyl isomerase A (cyclophilin A) | protein folding |  | | | | | | | | | | | | | | | | | | | | | | | | | | | |
| 1385 | 215781\_s\_at | TOP3B | 1.181002508 | 5.824002707 | 5.66903783 | 1.48E-005 | 0.000185184 | topoisomerase (DNA) III beta | DNA modification, DNA topological change, DNA unwinding, chromosome organization and biogenesis (sensu Eukaryota) |  | | | | | | | | | | | | | | | | | | | | | | | | | | | |
| 1386 | 222011\_s\_at | TCP1 | 1.365649795 | 7.107686411 | 5.66837946 | 1.48E-005 | 0.000185353 | t-complex 1 | tubulin folding |  | | | | | | | | | | | | | | | | | | | | | | | | | | | |
| 1387 | 205046\_at | CENPE | 1.099965936 | 6.874513149 | 5.6681178 | 1.48E-005 | 0.000185358 | centromere protein E, 312kDa | DNA replication and chromosome cycle, cytokinesis, mitotic chromosome movement, mitotic metaphase plate congression |  | | | | | | | | | | | | | | | | | | | | | | | | | | | |
| 1388 | 215631\_s\_at | BRMS1 | 0.49612072 | 8.032509505 | 5.66743356 | 1.48E-005 | 0.000185537 | breast cancer metastasis suppressor 1 | negative regulation of cell cycle |  | | | | | | | | | | | | | | | | | | | | | | | | | | | |
| 1389 | 217931\_at | TNRC5 | -0.612169427 | 8.781495464 | -5.66477235 | 1.49E-005 | 0.000186542 | trinucleotide repeat containing 5 | NA |  | | | | | | | | | | | | | | | | | | | | | | | | | | | |
| 1390 | 210210\_at | MPZL1 | -0.622637364 | 6.590127256 | -5.66445906 | 1.49E-005 | 0.000186568 | myelin protein zero-like 1 | cell-cell signaling, transmembrane receptor protein tyrosine kinase signaling pathway |  | | | | | | | | | | | | | | | | | | | | | | | | | | | |
| 1391 | 218105\_s\_at | MRPL4 | 1.827112705 | 8.776865081 | 5.66239416 | 1.50E-005 | 0.000187314 | mitochondrial ribosomal protein L4 | protein biosynthesis |  | | | | | | | | | | | | | | | | | | | | | | | | | | | |
| 1392 | 218189\_s\_at | NANS | 1.120895497 | 8.101487258 | 5.66217881 | 1.50E-005 | 0.000187314 | N-acetylneuraminic acid synthase (sialic acid synthase) | carbohydrate biosynthesis, cold acclimation, homoiothermy, lipopolysaccharide biosynthesis, response to freezing |  | | | | | | | | | | | | | | | | | | | | | | | | | | | |
| 1393 | 202402\_s\_at | CARS | 1.195707536 | 7.789959456 | 5.66160292 | 1.50E-005 | 0.00018745 | cysteinyl-tRNA synthetase | cysteinyl-tRNA aminoacylation, protein biosynthesis |  | | | | | | | | | | | | | | | | | | | | | | | | | | | |
| 1394 | 205526\_s\_at | KATNA1 | 0.941034266 | 7.696536906 | 5.65970283 | 1.51E-005 | 0.000187934 | katanin p60 (ATPase-containing) subunit A 1 | mitosis |  | | | | | | | | | | | | | | | | | | | | | | | | | | | |
| 1395 | 220172\_at | FLJ13096 | 0.5237745 | 4.905195998 | 5.65912955 | 1.51E-005 | 0.00018807 | hypothetical protein FLJ13096 | NA |  | | | | | | | | | | | | | | | | | | | | | | | | | | | |
| 1396 | 211594\_s\_at | MRPL9 | 1.086694727 | 8.120829768 | 5.65871327 | 1.51E-005 | 0.00018814 | mitochondrial ribosomal protein L9 /// mitochondrial ribosomal protein L9 | protein biosynthesis |  | | | | | | | | | | | | | | | | | | | | | | | | | | | |
| 1397 | 52731\_at | FLJ20294 | 0.699551139 | 4.840354106 | 5.65806257 | 1.51E-005 | 0.000188249 | hypothetical protein FLJ20294 | NA |  | | | | | | | | | | | | | | | | | | | | | | | | | | | |
| 1398 | 200027\_at | NARS | 1.66513189 | 10.0460399 | 5.65795641 | 1.52E-005 | 0.000188249 | asparaginyl-tRNA synthetase /// asparaginyl-tRNA synthetase | asparaginyl-tRNA aminoacylation, aspartyl-tRNA aminoacylation, protein biosynthesis |  | | | | | | | | | | | | | | | | | | | | | | | | | | | |
| 1399 | 218767\_at | XPMC2H | 0.961297809 | 7.975156358 | 5.65674544 | 1.52E-005 | 0.000188599 | XPMC2 prevents mitotic catastrophe 2 homolog (Xenopus laevis) | regulation of transcription, DNA-dependent |  | | | | | | | | | | | | | | | | | | | | | | | | | | | |
| 1400 | 201513\_at | TSN | 0.70354347 | 6.623058327 | 5.65661522 | 1.52E-005 | 0.000188599 | translin | DNA recombination |  | | | | | | | | | | | | | | | | | | | | | | | | | | | |
| 1401 | 204962\_s\_at | CENPA | 1.994209019 | 6.57862903 | 5.65637921 | 1.52E-005 | 0.000188599 | centromere protein A, 17kDa | chromosome organization and biogenesis (sensu Eukaryota), nucleosome assembly |  | | | | | | | | | | | | | | | | | | | | | | | | | | | |
| 1402 | 205372\_at | PLAG1 | 0.473307 | 4.358004526 | 5.65612309 | 1.52E-005 | 0.000188602 | pleiomorphic adenoma gene 1 | NA |  | | | | | | | | | | | | | | | | | | | | | | | | | | | |
| 1403 | 202703\_at | DUSP11 | 0.702928654 | 7.911298207 | 5.65488766 | 1.53E-005 | 0.00018902 | dual specificity phosphatase 11 (RNA/RNP complex 1-interacting) | RNA processing, protein amino acid dephosphorylation |  | | | | | | | | | | | | | | | | | | | | | | | | | | | |
| 1404 | 206205\_at | MPHOSPH9 | 0.776920781 | 6.190217351 | 5.64908138 | 1.55E-005 | 0.000191284 | M-phase phosphoprotein 9 | M phase of mitotic cell cycle, regulation of cell cycle |  | | | | | | | | | | | | | | | | | | | | | | | | | | | |
| 1405 | 202231\_at | GA17 | 1.813563069 | 9.833659453 | 5.64850031 | 1.55E-005 | 0.000191427 | dendritic cell protein | NA |  | | | | | | | | | | | | | | | | | | | | | | | | | | | |
| 1406 | 213097\_s\_at | ZRF1 | 1.489792688 | 8.566927171 | 5.64776907 | 1.55E-005 | 0.000191634 | zuotin related factor 1 | protein folding |  | | | | | | | | | | | | | | | | | | | | | | | | | | | |
| 1407 | 214086\_s\_at | PARP2 | 1.177772268 | 6.51573856 | 5.64256438 | 1.57E-005 | 0.00019367 | poly (ADP-ribose) polymerase family, member 2 | protein amino acid ADP-ribosylation |  | | | | | | | | | | | | | | | | | | | | | | | | | | | |
| 1408 | 220023\_at | APOB48R | -1.843851121 | 7.883542036 | -5.64199484 | 1.57E-005 | 0.00019381 | apolipoprotein B48 receptor | NA |  | | | | | | | | | | | | | | | | | | | | | | | | | | | |
| 1409 | 203624\_at | DXYS155E | -0.926663227 | 8.432604412 | -5.63847109 | 1.58E-005 | 0.000195223 | DNA segment on chromosome X and Y (unique) 155 expressed sequence | B-cell activation, regulation of transcription, DNA-dependent, signal transduction |  | | | | | | | | | | | | | | | | | | | | | | | | | | | |
| 1410 | 217995\_at | SQRDL | -2.001349035 | 9.652838431 | -5.63826061 | 1.58E-005 | 0.000195223 | sulfide quinone reductase-like (yeast) | electron transport |  | | | | | | | | | | | | | | | | | | | | | | | | | | | |
| 1411 | 218347\_at | FLJ10900 | 0.79468934 | 7.781790953 | 5.63793934 | 1.58E-005 | 0.000195256 | hypothetical protein FLJ10900 | electron transport |  | | | | | | | | | | | | | | | | | | | | | | | | | | | |
| 1412 | 201930\_at | MCM6 | 1.675542861 | 9.943119632 | 5.63767584 | 1.59E-005 | 0.000195263 | MCM6 minichromosome maintenance deficient 6 (MIS5 homolog, S. pombe) (S. cerevisiae) | DNA replication, DNA replication initiation, cell cycle, regulation of transcription, DNA-dependent |  | | | | | | | | | | | | | | | | | | | | | | | | | | | |
| 1413 | 203755\_at | BUB1B | 1.941037421 | 7.929077299 | 5.63622632 | 1.59E-005 | 0.000195791 | BUB1 budding uninhibited by benzimidazoles 1 homolog beta (yeast) | cell cycle, mitosis, mitotic checkpoint, protein amino acid phosphorylation |  | | | | | | | | | | | | | | | | | | | | | | | | | | | |
| 1414 | 201103\_x\_at | MGC8902 /// LOC376745 /// LOC200030 /// LOC348482 | -0.632802708 | 10.02304564 | -5.6308573 | 1.61E-005 | 0.000197727 | hypothetical protein MGC8902 /// AG1 /// hypothetical protein LOC200030 /// hypothetical protein LOC348482 | NA |  | | | | | | | | | | | | | | | | | | | | | | | | | | | |
| 1415 | 203763\_at | D2LIC | 0.852628223 | 5.646727696 | 5.62946876 | 1.62E-005 | 0.000198125 | dynein 2 light intermediate chain | NA |  | | | | | | | | | | | | | | | | | | | | | | | | | | | |
| 1416 | 213414\_s\_at | RPS19 | 0.735415575 | 12.76028175 | 5.62872814 | 1.62E-005 | 0.000198236 | ribosomal protein S19 | hemocyte development, protein biosynthesis |  | | | | | | | | | | | | | | | | | | | | | | | | | | | |
| 1417 | 219662\_at | MGC5509 | 0.94384267 | 6.052096464 | 5.62723244 | 1.62E-005 | 0.000198793 | hypothetical protein MGC5509 | NA |  | | | | | | | | | | | | | | | | | | | | | | | | | | | |
| 1418 | 210415\_s\_at | ODF2 | 0.852613231 | 6.796635277 | 5.62471072 | 1.63E-005 | 0.000199812 | outer dense fiber of sperm tails 2 | NA |  | | | | | | | | | | | | | | | | | | | | | | | | | | | |
| 1419 | 212627\_s\_at | EXOSC7 | 1.37246399 | 8.500244943 | 5.62090643 | 1.65E-005 | 0.000201193 | exosome component 7 | RNA catabolism, rRNA processing |  | | | | | | | | | | | | | | | | | | | | | | | | | | | |
| 1420 | 212118\_at | RFP | 0.555260856 | 8.559014603 | 5.61877643 | 1.65E-005 | 0.000202047 | ret finger protein | cell proliferation, protein ubiquitination, regulation of transcription, DNA-dependent, spermatogenesis, transcription |  | | | | | | | | | | | | | | | | | | | | | | | | | | | |
| 1421 | 219637\_at | FLJ12584 | 0.493362021 | 4.796527065 | 5.61801206 | 1.66E-005 | 0.000202283 | hypothetical protein FLJ12584 | NA |  | | | | | | | | | | | | | | | | | | | | | | | | | | | |
| 1422 | 212088\_at | PMPCA | 0.861808752 | 8.095217277 | 5.61632372 | 1.66E-005 | 0.000202857 | peptidase (mitochondrial processing) alpha | proteolysis and peptidolysis |  | | | | | | | | | | | | | | | | | | | | | | | | | | | |
| 1423 | 200079\_s\_at | KARS | 1.372680255 | 9.761985605 | 5.61584565 | 1.67E-005 | 0.000202936 | lysyl-tRNA synthetase /// lysyl-tRNA synthetase | aspartyl-tRNA aminoacylation, lysyl-tRNA aminoacylation, protein biosynthesis, tRNA processing |  | | | | | | | | | | | | | | | | | | | | | | | | | | | |
| 1424 | 219551\_at | EAF2 | -2.059610834 | 8.410836518 | -5.61547517 | 1.67E-005 | 0.000202993 | ELL associated factor 2 | NA |  | | | | | | | | | | | | | | | | | | | | | | | | | | | |
| 1425 | 218065\_s\_at | C11orf15 | -0.770455279 | 8.739865341 | -5.61461684 | 1.67E-005 | 0.000203274 | chromosome 11 open reading frame 15 | positive regulation of I-kappaB kinase/NF-kappaB cascade |  | | | | | | | | | | | | | | | | | | | | | | | | | | | |
| 1426 | 206653\_at | POLR3G | 0.660474626 | 4.768923556 | 5.61220635 | 1.68E-005 | 0.000204266 | Polymerase (RNA) III (DNA directed) polypeptide G (32kD) | regulation of transcription from Pol III promoter |  | | | | | | | | | | | | | | | | | | | | | | | | | | | |
| 1427 | 211423\_s\_at | SC5DL | 1.045185531 | 6.738984482 | 5.6100606 | 1.69E-005 | 0.000205031 | sterol-C5-desaturase (ERG3 delta-5-desaturase homolog, fungal)-like | lipid metabolism, sterol biosynthesis |  | | | | | | | | | | | | | | | | | | | | | | | | | | | |
| 1428 | 201813\_s\_at | TBC1D5 | -0.467648576 | 8.838576402 | -5.61005467 | 1.69E-005 | 0.000205031 | TBC1 domain family, member 5 | NA |  | | | | | | | | | | | | | | | | | | | | | | | | | | | |
| 1429 | 218722\_s\_at | FLJ12436 | 0.654339237 | 7.507160964 | 5.60917733 | 1.69E-005 | 0.000205311 | hypothetical protein FLJ12436 | NA |  | | | | | | | | | | | | | | | | | | | | | | | | | | | |
| 1430 | 206631\_at | PTGER2 | -1.715444059 | 7.31376444 | -5.60896179 | 1.69E-005 | 0.000205311 | prostaglandin E receptor 2 (subtype EP2), 53kDa | G-protein coupled receptor protein signaling pathway |  | | | | | | | | | | | | | | | | | | | | | | | | | | | |
| 1431 | 204247\_s\_at | CDK5 | 0.765523943 | 7.650875514 | 5.60436181 | 1.71E-005 | 0.000207218 | cyclin-dependent kinase 5 | cell cycle, cytokinesis, protein amino acid phosphorylation |  | | | | | | | | | | | | | | | | | | | | | | | | | | | |
| 1432 | 200074\_s\_at | RPL14 | 1.309577431 | 11.50603727 | 5.60206114 | 1.72E-005 | 0.000208179 | ribosomal protein L14 /// ribosomal protein L14 | protein biosynthesis |  | | | | | | | | | | | | | | | | | | | | | | | | | | | |
| 1433 | 209889\_at | SEC31L2 | -0.71557473 | 7.341300831 | -5.59617023 | 1.74E-005 | 0.000210839 | SEC31-like 2 (S. cerevisiae) | proteolysis and peptidolysis |  | | | | | | | | | | | | | | | | | | | | | | | | | | | |
| 1434 | 218465\_at | TMEM33 | 1.113126665 | 7.717575388 | 5.5951999 | 1.74E-005 | 0.000211185 | transmembrane protein 33 | NA |  | | | | | | | | | | | | | | | | | | | | | | | | | | | |
| 1435 | 214482\_at | ZNF46 | -0.446892141 | 6.343936238 | -5.59425065 | 1.75E-005 | 0.000211521 | zinc finger protein 46 (KUP) | regulation of transcription, DNA-dependent |  | | | | | | | | | | | | | | | | | | | | | | | | | | | |
| 1436 | 203778\_at | MANBA | -0.55069728 | 8.240542217 | -5.59124115 | 1.76E-005 | 0.00021261 | mannosidase, beta A, lysosomal | carbohydrate metabolism, protein modification |  | | | | | | | | | | | | | | | | | | | | | | | | | | | |
| 1437 | 209158\_s\_at | PSCD2 | 1.298471964 | 7.732137938 | 5.58963065 | 1.77E-005 | 0.000213266 | pleckstrin homology, Sec7 and coiled-coil domains 2 (cytohesin-2) | actin cytoskeleton organization and biogenesis, endocytosis |  | | | | | | | | | | | | | | | | | | | | | | | | | | | |
| 1438 | 205214\_at | STK17B | -2.048990628 | 8.304396573 | -5.589176 | 1.77E-005 | 0.000213368 | serine/threonine kinase 17b (apoptosis-inducing) | apoptosis, induction of apoptosis, protein amino acid phosphorylation, protein kinase cascade |  | | | | | | | | | | | | | | | | | | | | | | | | | | | |
| 1439 | 221751\_at | PANK3 | 1.126471073 | 7.112687684 | 5.58791225 | 1.77E-005 | 0.000213771 | Pantothenate kinase 3 | NA |  | | | | | | | | | | | | | | | | | | | | | | | | | | | |
| 1440 | 201595\_s\_at | LEREPO4 | 1.379388452 | 8.525926585 | 5.58761338 | 1.77E-005 | 0.000213771 | likely ortholog of mouse immediate early response, erythropoietin 4 | NA |  | | | | | | | | | | | | | | | | | | | | | | | | | | | |
| 1441 | 213859\_x\_at | SMARCA5 | 0.843270066 | 5.490944709 | 5.58713126 | 1.78E-005 | 0.000213887 | SWI/SNF related, matrix associated, actin dependent regulator of chromatin, subfamily a, member 5 | chromatin remodeling, regulation of transcription from Pol II promoter |  | | | | | | | | | | | | | | | | | | | | | | | | | | | |
| 1442 | 200046\_at | DAD1 | 0.67199693 | 9.225386304 | 5.58616532 | 1.78E-005 | 0.000214102 | defender against cell death 1 /// defender against cell death 1 | NA |  | | | | | | | | | | | | | | | | | | | | | | | | | | | |
| 1443 | 204565\_at | THEM2 | 1.194763523 | 7.448914404 | 5.58596127 | 1.78E-005 | 0.000214102 | thioesterase superfamily member 2 | NA |  | | | | | | | | | | | | | | | | | | | | | | | | | | | |
| 1444 | 217806\_s\_at | POLDIP2 | 0.520266208 | 8.286231249 | 5.5844388 | 1.79E-005 | 0.000214699 | polymerase (DNA-directed), delta interacting protein 2 | NA |  | | | | | | | | | | | | | | | | | | | | | | | | | | | |
| 1445 | 201376\_s\_at | HNRPF | 0.781985615 | 8.704644578 | 5.58413897 | 1.79E-005 | 0.000214699 | heterogeneous nuclear ribonucleoprotein F | RNA processing |  | | | | | | | | | | | | | | | | | | | | | | | | | | | |
| 1446 | 217884\_at | FLJ10774 | 1.341188837 | 8.843443178 | 5.58288411 | 1.79E-005 | 0.000215124 | N-acetyltransferase-like protein | NA |  | | | | | | | | | | | | | | | | | | | | | | | | | | | |
| 1447 | 200714\_x\_at | OS-9 | -0.387643473 | 10.22018803 | -5.58243968 | 1.80E-005 | 0.000215203 | amplified in osteosarcoma | cell growth and/or maintenance |  | | | | | | | | | | | | | | | | | | | | | | | | | | | |
| 1448 | 221776\_s\_at | BRD7 | 0.987535249 | 7.782033473 | 5.58224244 | 1.80E-005 | 0.000215203 | bromodomain containing 7 | NA |  | | | | | | | | | | | | | | | | | | | | | | | | | | | |
| 1449 | 219680\_at | NOD9 | -0.679950951 | 8.40646464 | -5.58146199 | 1.80E-005 | 0.000215465 | NOD9 protein | NA |  | | | | | | | | | | | | | | | | | | | | | | | | | | | |
| 1450 | 219242\_at | Cep63 | -0.859105387 | 7.551140258 | -5.57794053 | 1.81E-005 | 0.000217062 | centrosome protein Cep63 | NA |  | | | | | | | | | | | | | | | | | | | | | | | | | | | |
| 1451 | 219860\_at | LY6G5C | -0.446991142 | 6.047840601 | -5.57657675 | 1.82E-005 | 0.000217612 | lymphocyte antigen 6 complex, locus G5C | NA |  | | | | | | | | | | | | | | | | | | | | | | | | | | | |
| 1452 | 205740\_s\_at | MGC10433 | 0.732288495 | 8.293169225 | 5.57483313 | 1.83E-005 | 0.00021835 | hypothetical protein MGC10433 | NA |  | | | | | | | | | | | | | | | | | | | | | | | | | | | |
| 1453 | 218585\_s\_at | RAMP | 1.732418018 | 7.494988396 | 5.5727166 | 1.83E-005 | 0.000219275 | RA-regulated nuclear matrix-associated protein | NA |  | | | | | | | | | | | | | | | | | | | | | | | | | | | |
| 1454 | 217486\_s\_at | ZDHHC17 | -0.66786147 | 6.389757098 | -5.57152112 | 1.84E-005 | 0.000219748 | zinc finger, DHHC domain containing 17 | positive regulation of I-kappaB kinase/NF-kappaB cascade |  | | | | | | | | | | | | | | | | | | | | | | | | | | | |
| 1455 | 212904\_at | KIAA1185 | 1.192115297 | 8.855821056 | 5.57055495 | 1.84E-005 | 0.000219962 | KIAA1185 protein | NA |  | | | | | | | | | | | | | | | | | | | | | | | | | | | |
| 1456 | 208630\_at | HADHA | 1.091686229 | 9.469229277 | 5.57037272 | 1.84E-005 | 0.000219962 | hydroxyacyl-Coenzyme A dehydrogenase/3-ketoacyl-Coenzyme A thiolase/enoyl-Coenzyme A hydratase (trifunctional protein), alpha subunit | fatty acid metabolism, metabolism |  | | | | | | | | | | | | | | | | | | | | | | | | | | | |
| 1457 | 219999\_at | MAN2A2 | -0.977938842 | 7.565049109 | -5.56771304 | 1.86E-005 | 0.000221046 | mannosidase, alpha, class 2A, member 2 | carbohydrate metabolism |  | | | | | | | | | | | | | | | | | | | | | | | | | | | |
| 1458 | 202531\_at | IRF1 | -1.500376038 | 8.669318261 | -5.56684914 | 1.86E-005 | 0.000221358 | interferon regulatory factor 1 | immune response, negative regulation of cell cycle, regulation of transcription, DNA-dependent, transcription from Pol II promoter |  | | | | | | | | | | | | | | | | | | | | | | | | | | | |
| 1459 | 201074\_at | SMARCC1 | 1.296795495 | 8.753793652 | 5.56565031 | 1.86E-005 | 0.000221767 | SWI/SNF related, matrix associated, actin dependent regulator of chromatin, subfamily c, member 1 | chromatin assembly or disassembly, chromatin remodeling, regulation of transcription from Pol II promoter |  | | | | | | | | | | | | | | | | | | | | | | | | | | | |
| 1460 | 217990\_at | GMPR2 | 0.898611275 | 8.328710941 | 5.56545841 | 1.87E-005 | 0.000221767 | guanosine monophosphate reductase 2 | nucleotide metabolism |  | | | | | | | | | | | | | | | | | | | | | | | | | | | |
| 1461 | 221596\_s\_at | DKFZP564O0523 | 0.999146327 | 4.903631022 | 5.56531493 | 1.87E-005 | 0.000221767 | hypothetical protein DKFZp564O0523 | NA |  | | | | | | | | | | | | | | | | | | | | | | | | | | | |
| 1462 | 201622\_at | SND1 | 0.956144371 | 9.875856282 | 5.5603413 | 1.89E-005 | 0.000224146 | staphylococcal nuclease domain containing 1 | NA |  | | | | | | | | | | | | | | | | | | | | | | | | | | | |
| 1463 | 204097\_s\_at | RBMX2 | 1.226527097 | 6.960988638 | 5.55949085 | 1.89E-005 | 0.000224456 | RNA binding motif protein, X-linked 2 | NA |  | | | | | | | | | | | | | | | | | | | | | | | | | | | |
| 1464 | 209103\_s\_at | UFD1L | 1.269997796 | 8.777246107 | 5.55877902 | 1.89E-005 | 0.000224696 | ubiquitin fusion degradation 1-like | skeletal development, ubiquitin cycle, ubiquitin-dependent protein catabolism |  | | | | | | | | | | | | | | | | | | | | | | | | | | | |
| 1465 | 206364\_at | KIF14 | 1.514764048 | 6.38402383 | 5.55695874 | 1.90E-005 | 0.000225425 | Kinesin family member 14 | NA |  | | | | | | | | | | | | | | | | | | | | | | | | | | | |
| 1466 | 203677\_s\_at | TARBP2 | 0.714706962 | 7.615725175 | 5.55668792 | 1.90E-005 | 0.000225425 | TAR (HIV) RNA binding protein 2 | regulation of transcription from Pol II promoter |  | | | | | | | | | | | | | | | | | | | | | | | | | | | |
| 1467 | 212782\_x\_at | POLR2J | 0.7772181 | 9.817444578 | 5.55663005 | 1.90E-005 | 0.000225425 | polymerase (RNA) II (DNA directed) polypeptide J, 13.3kDa | transcription, transcription from Pol II promoter |  | | | | | | | | | | | | | | | | | | | | | | | | | | | |
| 1468 | 218528\_s\_at | RNF38 | -0.917187162 | 7.247470189 | -5.55449028 | 1.91E-005 | 0.000226178 | ring finger protein 38 | protein ubiquitination |  | | | | | | | | | | | | | | | | | | | | | | | | | | | |
| 1469 | 204709\_s\_at | KIF23 | 0.978239118 | 4.364843555 | 5.55403024 | 1.91E-005 | 0.000226267 | kinesin family member 23 | cell cycle, cytokinesis, mitosis, mitotic spindle elongation |  | | | | | | | | | | | | | | | | | | | | | | | | | | | |
| 1470 | 219683\_at | FZD3 | 0.490549275 | 4.105259423 | 5.55365689 | 1.92E-005 | 0.000226337 | frizzled homolog 3 (Drosophila) | G-protein coupled receptor protein signaling pathway, cell proliferation, development, frizzled signaling pathway |  | | | | | | | | | | | | | | | | | | | | | | | | | | | |
| 1471 | 213827\_at | SNX26 | -0.696285929 | 7.650969925 | -5.55164072 | 1.92E-005 | 0.000227246 | sorting nexin 26 | protein transport |  | | | | | | | | | | | | | | | | | | | | | | | | | | | |
| 1472 | 201260\_s\_at | SYPL | 0.974696947 | 8.568946583 | 5.54950495 | 1.93E-005 | 0.000228221 | synaptophysin-like protein | synaptic transmission, transport |  | | | | | | | | | | | | | | | | | | | | | | | | | | | |
| 1473 | 203291\_at | CNOT4 | 0.74809716 | 6.730005716 | 5.54857825 | 1.94E-005 | 0.000228577 | CCR4-NOT transcription complex, subunit 4 | protein ubiquitination |  | | | | | | | | | | | | | | | | | | | | | | | | | | | |
| 1474 | 201247\_at | SREBF2 | 1.059853493 | 8.092323302 | 5.5471558 | 1.94E-005 | 0.000229119 | sterol regulatory element binding transcription factor 2 | cholesterol metabolism, lipid metabolism, regulation of transcription from Pol II promoter |  | | | | | | | | | | | | | | | | | | | | | | | | | | | |
| 1475 | 213377\_x\_at | RPS12 | 0.670204259 | 13.08025342 | 5.54693299 | 1.94E-005 | 0.000229119 | ribosomal protein S12 | protein biosynthesis |  | | | | | | | | | | | | | | | | | | | | | | | | | | | |
| 1476 | 221597\_s\_at | HSPC171 | 0.591363666 | 7.666957316 | 5.54463458 | 1.95E-005 | 0.000230129 | HSPC171 protein | NA |  | | | | | | | | | | | | | | | | | | | | | | | | | | | |
| 1477 | 218535\_s\_at | RIOK2 | 1.001151334 | 7.217683837 | 5.54201204 | 1.97E-005 | 0.000231249 | RIO kinase 2 (yeast) | NA |  | | | | | | | | | | | | | | | | | | | | | | | | | | | |
| 1478 | 201357\_s\_at | SF3A1 | 0.946217288 | 8.218749627 | 5.53932799 | 1.98E-005 | 0.000232414 | splicing factor 3a, subunit 1, 120kDa | RNA splicing, nuclear mRNA splicing, via spliceosome |  | | | | | | | | | | | | | | | | | | | | | | | | | | | |
| 1479 | 204065\_at | CHST10 | 0.540922731 | 7.405251932 | 5.53931416 | 1.98E-005 | 0.000232414 | carbohydrate sulfotransferase 10 | cell adhesion |  | | | | | | | | | | | | | | | | | | | | | | | | | | | |
| 1480 | 212563\_at | BOP1 | 1.765024393 | 7.339828418 | 5.53775573 | 1.99E-005 | 0.000233108 | block of proliferation 1 | electron transport, rRNA processing |  | | | | | | | | | | | | | | | | | | | | | | | | | | | |
| 1481 | 221267\_s\_at | C19orf27 | -0.698402567 | 9.198228063 | -5.53629643 | 1.99E-005 | 0.000233753 | chromosome 19 open reading frame 27 /// chromosome 19 open reading frame 27 | NA |  | | | | | | | | | | | | | | | | | | | | | | | | | | | |
| 1482 | 216684\_s\_at | SS18 | 0.587968298 | 6.480494271 | 5.53508338 | 2.00E-005 | 0.000234146 | synovial sarcoma translocation, chromosome 18 | cell growth and/or maintenance |  | | | | | | | | | | | | | | | | | | | | | | | | | | | |
| 1483 | 221864\_at | MGC13024 | -0.826784843 | 9.03366724 | -5.53478101 | 2.00E-005 | 0.000234182 | hypothetical protein MGC13024 | NA |  | | | | | | | | | | | | | | | | | | | | | | | | | | | |
| 1484 | 209382\_at | POLR3C | 1.107664398 | 7.588468498 | 5.53281462 | 2.01E-005 | 0.000235099 | polymerase (RNA) III (DNA directed) polypeptide C (62kD) | transcription |  | | | | | | | | | | | | | | | | | | | | | | | | | | | |
| 1485 | 213915\_at | NKG7 | -2.777905769 | 9.034560592 | -5.53243847 | 2.01E-005 | 0.000235175 | natural killer cell group 7 sequence | NA |  | | | | | | | | | | | | | | | | | | | | | | | | | | | |
| 1486 | 213454\_at | CORT | 1.069284833 | 5.918006895 | 5.53041826 | 2.02E-005 | 0.000236 | cortistatin | G-protein signaling, adenylate cyclase inhibiting pathway, synaptic transmission |  | | | | | | | | | | | | | | | | | | | | | | | | | | | |
| 1487 | 212058\_at | SR140 | 1.066131784 | 8.52648679 | 5.52854674 | 2.03E-005 | 0.000236874 | U2-associated SR140 protein | NA |  | | | | | | | | | | | | | | | | | | | | | | | | | | | |
| 1488 | 204444\_at | KIF11 | 1.824028451 | 6.640161022 | 5.52413162 | 2.05E-005 | 0.000239027 | kinesin family member 11 | mitotic spindle assembly |  | | | | | | | | | | | | | | | | | | | | | | | | | | | |
| 1489 | 209015\_s\_at | DNAJB6 | 1.206296977 | 7.117996702 | 5.5240701 | 2.05E-005 | 0.000239027 | DnaJ (Hsp40) homolog, subfamily B, member 6 | protein folding, response to unfolded protein |  | | | | | | | | | | | | | | | | | | | | | | | | | | | |
| 1490 | 201770\_at | SNRPA | 1.167399294 | 9.604730453 | 5.52359368 | 2.05E-005 | 0.000239159 | small nuclear ribonucleoprotein polypeptide A | NA |  | | | | | | | | | | | | | | | | | | | | | | | | | | | |
| 1491 | 201175\_at | TMX2 | 1.365966274 | 8.715804641 | 5.51873377 | 2.07E-005 | 0.000241669 | thioredoxin-related transmembrane protein 2 | electron transport |  | | | | | | | | | | | | | | | | | | | | | | | | | | | |
| 1492 | 201541\_s\_at | ZNHIT1 | 1.115773961 | 8.606740311 | 5.5180042 | 2.08E-005 | 0.000241899 | zinc finger, HIT domain containing 1 | NA |  | | | | | | | | | | | | | | | | | | | | | | | | | | | |
| 1493 | 201241\_at | DDX1 | 1.362473859 | 8.869942212 | 5.51784738 | 2.08E-005 | 0.000241899 | DEAD (Asp-Glu-Ala-Asp) box polypeptide 1 | development, glycolysis, regulation of translational initiation, ribosome biogenesis, spliceosome assembly |  | | | | | | | | | | | | | | | | | | | | | | | | | | | |
| 1494 | 201587\_s\_at | IRAK1 | 1.029876557 | 9.426245173 | 5.51751941 | 2.08E-005 | 0.000241952 | interleukin-1 receptor-associated kinase 1 | activation of NF-kappaB-inducing kinase, autophosphorylation, defense response, positive regulation of transcription, protein amino acid phosphorylation, signal transduction, transmembrane receptor protein serine/threonine kinase signaling pathway |  | | | | | | | | | | | | | | | | | | | | | | | | | | | |
| 1495 | 222014\_x\_at | MTO1 | 1.39476022 | 6.943591703 | 5.51389531 | 2.10E-005 | 0.000243811 | mitochondrial translation optimization 1 homolog (S. cerevisiae) | electron transport |  | | | | | | | | | | | | | | | | | | | | | | | | | | | |
| 1496 | 217854\_s\_at | POLR2E | 1.093906222 | 9.26654663 | 5.51351387 | 2.10E-005 | 0.000243894 | polymerase (RNA) II (DNA directed) polypeptide E, 25kDa | transcription, transcription from Pol II promoter |  | | | | | | | | | | | | | | | | | | | | | | | | | | | |
| 1497 | 218743\_at | FLJ11749 | -0.513653876 | 7.324366546 | -5.51299101 | 2.10E-005 | 0.000244054 | hypothetical protein FLJ11749 | NA |  | | | | | | | | | | | | | | | | | | | | | | | | | | | |
| 1498 | 202339\_at | SYMPK | 0.445343994 | 7.311895654 | 5.51232358 | 2.10E-005 | 0.000244294 | symplekin | cell adhesion |  | | | | | | | | | | | | | | | | | | | | | | | | | | | |
| 1499 | 211769\_x\_at | TDE1 | -0.621523611 | 10.16234831 | -5.5117669 | 2.11E-005 | 0.000244473 | tumor differentially expressed 1 /// tumor differentially expressed 1 | NA |  | | | | | | | | | | | | | | | | | | | | | | | | | | | |
| 1500 | 203249\_at | EZH1 | -0.565770007 | 7.944583088 | -5.51126007 | 2.11E-005 | 0.000244625 | enhancer of zeste homolog 1 (Drosophila) | G-protein coupled receptor protein signaling pathway, morphogenesis, regulation of transcription, DNA-dependent |  | | | | | | | | | | | | | | | | | | | | | | | | | | | |
| 1501 | 202261\_at | TCFL1 | 0.692508891 | 8.636024045 | 5.50810011 | 2.12E-005 | 0.000245992 | transcription factor-like 1 | negative regulation of transcription from Pol II promoter, regulation of transcription, DNA-dependent |  | | | | | | | | | | | | | | | | | | | | | | | | | | | |
| 1502 | 201761\_at | MTHFD2 | 2.571950446 | 9.454899414 | 5.50708719 | 2.13E-005 | 0.000246308 | methylene tetrahydrofolate dehydrogenase (NAD+ dependent), methenyltetrahydrofolate cyclohydrolase | folic acid and derivative biosynthesis, one-carbon compound metabolism |  | | | | | | | | | | | | | | | | | | | | | | | | | | | |
| 1503 | 220367\_s\_at | SAP130 | 0.596154147 | 7.592698416 | 5.50706875 | 2.13E-005 | 0.000246308 | mSin3A-associated protein 130 | NA |  | | | | | | | | | | | | | | | | | | | | | | | | | | | |
| 1504 | 203545\_at | ALG8 | 1.394199128 | 8.193659662 | 5.50609729 | 2.13E-005 | 0.000246721 | asparagine-linked glycosylation 8 homolog (yeast, alpha-1,3-glucosyltransferase) | N-linked glycosylation, transport |  | | | | | | | | | | | | | | | | | | | | | | | | | | | |
| 1505 | 204167\_at | BTD | -0.470484776 | 6.962671955 | -5.50434562 | 2.14E-005 | 0.00024757 | biotinidase | biotin metabolism, central nervous system development, epidermis development, nitrogen metabolism |  | | | | | | | | | | | | | | | | | | | | | | | | | | | |
| 1506 | 212774\_at | ZNF238 | -1.524581349 | 7.798380946 | -5.50302022 | 2.15E-005 | 0.000248183 | zinc finger protein 238 | regulation of transcription, DNA-dependent, transport |  | | | | | | | | | | | | | | | | | | | | | | | | | | | |
| 1507 | 202905\_x\_at | NBS1 | -1.063900393 | 8.131958783 | -5.49847548 | 2.17E-005 | 0.000250613 | Nijmegen breakage syndrome 1 (nibrin) | cell cycle checkpoint, double-strand break repair |  | | | | | | | | | | | | | | | | | | | | | | | | | | | |
| 1508 | 212512\_s\_at | CARM1 | 1.35643393 | 7.225610146 | 5.49712751 | 2.18E-005 | 0.000251247 | coactivator-associated arginine methyltransferase 1 | NA |  | | | | | | | | | | | | | | | | | | | | | | | | | | | |
| 1509 | 216197\_at | ATF7IP | -0.599994013 | 4.927166758 | -5.49615227 | 2.18E-005 | 0.00025167 | activating transcription factor 7 interacting protein | DNA methylation, negative regulation of transcription, DNA-dependent, positive regulation of transcription, DNA-dependent, regulation of transcriptional preinitiation complex formation |  | | | | | | | | | | | | | | | | | | | | | | | | | | | |
| 1510 | 212441\_at | KIAA0232 | -0.875248973 | 8.238184969 | -5.49586393 | 2.18E-005 | 0.000251704 | KIAA0232 gene product | NA |  | | | | | | | | | | | | | | | | | | | | | | | | | | | |
| 1511 | 206314\_at | ZNF167 | -0.597504757 | 4.624750681 | -5.49452367 | 2.19E-005 | 0.000251944 | zinc finger protein 167 | regulation of transcription, DNA-dependent |  | | | | | | | | | | | | | | | | | | | | | | | | | | | |
| 1512 | 213041\_s\_at | ATP5D | 1.319761514 | 8.666233235 | 5.49196782 | 2.20E-005 | 0.000252849 | ATP synthase, H+ transporting, mitochondrial F1 complex, delta subunit | ATP synthesis coupled proton transport, proton transport |  | | | | | | | | | | | | | | | | | | | | | | | | | | | |
| 1513 | 214700\_x\_at | Rif1 | 0.733879986 | 5.931308318 | 5.49159124 | 2.20E-005 | 0.000252849 | telomere-associated protein RIF1 homolog | NA |  | | | | | | | | | | | | | | | | | | | | | | | | | | | |
| 1514 | 212794\_s\_at | KIAA1033 | -1.198850775 | 8.447211789 | -5.49156335 | 2.20E-005 | 0.000252849 | KIAA1033 protein | NA |  | | | | | | | | | | | | | | | | | | | | | | | | | | | |
| 1515 | 209104\_s\_at | NOLA2 | 1.437238394 | 9.729013917 | 5.49085409 | 2.21E-005 | 0.000253123 | nucleolar protein family A, member 2 (H/ACA small nucleolar RNPs) | protein biosynthesis |  | | | | | | | | | | | | | | | | | | | | | | | | | | | |
| 1516 | 201068\_s\_at | PSMC2 | 1.287052903 | 9.713208812 | 5.4895906 | 2.21E-005 | 0.000253716 | proteasome (prosome, macropain) 26S subunit, ATPase, 2 | proteolysis and peptidolysis |  | | | | | | | | | | | | | | | | | | | | | | | | | | | |
| 1517 | 201275\_at | FDPS | 1.110217573 | 9.096736738 | 5.48918003 | 2.22E-005 | 0.000253821 | farnesyl diphosphate synthase (farnesyl pyrophosphate synthetase, dimethylallyltranstransferase, geranyltranstransferase) | cholesterol biosynthesis, isoprenoid biosynthesis |  | | | | | | | | | | | | | | | | | | | | | | | | | | | |
| 1518 | 218032\_at | SNN | -1.730209032 | 7.627943492 | -5.48855967 | 2.22E-005 | 0.000254046 | stannin | response to abiotic stimulus, response to stress |  | | | | | | | | | | | | | | | | | | | | | | | | | | | |
| 1519 | 204206\_at | MNT | -0.820664003 | 7.899487791 | -5.48587171 | 2.23E-005 | 0.000255361 | MAX binding protein | development, negative regulation of cell proliferation, regulation of cell cycle, regulation of transcription, DNA-dependent, transcription from Pol II promoter |  | | | | | | | | | | | | | | | | | | | | | | | | | | | |
| 1520 | 206686\_at | PDK1 | 0.705957762 | 7.057567964 | 5.48574954 | 2.23E-005 | 0.000255361 | pyruvate dehydrogenase kinase, isoenzyme 1 | glucose metabolism, protein amino acid phosphorylation, small GTPase mediated signal transduction |  | | | | | | | | | | | | | | | | | | | | | | | | | | | |
| 1521 | 203234\_at | UPP1 | -1.393546157 | 7.046748746 | -5.4855913 | 2.23E-005 | 0.000255361 | uridine phosphorylase 1 | nucleoside metabolism |  | | | | | | | | | | | | | | | | | | | | | | | | | | | |
| 1522 | 207769\_s\_at | PQBP1 | 1.084800664 | 7.674050658 | 5.48396403 | 2.24E-005 | 0.000256171 | polyglutamine binding protein 1 | regulation of transcription, DNA-dependent |  | | | | | | | | | | | | | | | | | | | | | | | | | | | |
| 1523 | 213599\_at | OIP5 | 1.380376381 | 6.832565752 | 5.47999442 | 2.26E-005 | 0.000258347 | Opa-interacting protein 5 | cell communication |  | | | | | | | | | | | | | | | | | | | | | | | | | | | |
| 1524 | 203374\_s\_at | TPP2 | 1.192793227 | 8.405437961 | 5.4786002 | 2.27E-005 | 0.000258904 | tripeptidyl peptidase II | proteolysis and peptidolysis |  | | | | | | | | | | | | | | | | | | | | | | | | | | | |
| 1525 | 212629\_s\_at | PKN2 | -1.174493397 | 7.122475367 | -5.47695743 | 2.28E-005 | 0.000259727 | protein kinase N2 | protein amino acid phosphorylation, signal transduction |  | | | | | | | | | | | | | | | | | | | | | | | | | | | |
| 1526 | 201285\_at | MKRN1 | -0.682850145 | 8.917159464 | -5.47237583 | 2.30E-005 | 0.000262029 | makorin, ring finger protein, 1 /// makorin, ring finger protein, 1 | protein ubiquitination |  | | | | | | | | | | | | | | | | | | | | | | | | | | | |
| 1527 | 218187\_s\_at | FLJ20989 | 1.208821955 | 6.695490104 | 5.47190961 | 2.30E-005 | 0.000262112 | hypothetical protein FLJ20989 | NA |  | | | | | | | | | | | | | | | | | | | | | | | | | | | |
| 1528 | 200610\_s\_at | NCL | 1.274572867 | 10.7735277 | 5.47178279 | 2.30E-005 | 0.000262112 | nucleolin | NA |  | | | | | | | | | | | | | | | | | | | | | | | | | | | |
| 1529 | 202272\_s\_at | FBXO28 | 0.723440721 | 7.411630322 | 5.47140346 | 2.31E-005 | 0.000262202 | F-box protein 28 | NA |  | | | | | | | | | | | | | | | | | | | | | | | | | | | |
| 1530 | 201707\_at | PEX19 | 0.747096593 | 6.957257997 | 5.46670707 | 2.33E-005 | 0.000264856 | peroxisomal biogenesis factor 19 | peroxisome organization and biogenesis |  | | | | | | | | | | | | | | | | | | | | | | | | | | | |
| 1531 | 221565\_s\_at | FAM26B | -1.203104838 | 7.454208758 | -5.46627469 | 2.33E-005 | 0.000264856 | family with sequence similarity 26, member B | NA |  | | | | | | | | | | | | | | | | | | | | | | | | | | | |
| 1532 | 201527\_at | ATP6V1F | 0.822174161 | 9.920511389 | 5.46575702 | 2.34E-005 | 0.000264937 | ATPase, H+ transporting, lysosomal 14kDa, V1 subunit F | ATP synthesis coupled proton transport, proton transport |  | | | | | | | | | | | | | | | | | | | | | | | | | | | |
| 1533 | 203288\_at | KIAA0355 | 0.493729609 | 6.346875512 | 5.46568688 | 2.34E-005 | 0.000264937 | KIAA0355 | NA |  | | | | | | | | | | | | | | | | | | | | | | | | | | | |
| 1534 | 212135\_s\_at | ATP2B4 | -0.68046467 | 9.011524877 | -5.4642911 | 2.34E-005 | 0.00026564 | ATPase, Ca++ transporting, plasma membrane 4 | calcium ion transport, cation transport, metabolism, transport |  | | | | | | | | | | | | | | | | | | | | | | | | | | | |
| 1535 | 212049\_at | WIRE | -0.558161219 | 8.558327936 | -5.46382422 | 2.35E-005 | 0.000265785 | WIRE protein | NA |  | | | | | | | | | | | | | | | | | | | | | | | | | | | |
| 1536 | 208775\_at | XPO1 | 1.104487283 | 9.37591245 | 5.46094959 | 2.36E-005 | 0.000267111 | exportin 1 (CRM1 homolog, yeast) | protein-nucleus import, docking |  | | | | | | | | | | | | | | | | | | | | | | | | | | | |
| 1537 | 201470\_at | GSTO1 | 0.933354704 | 10.39614714 | 5.4581045 | 2.38E-005 | 0.000268541 | glutathione S-transferase omega 1 | metabolism |  | | | | | | | | | | | | | | | | | | | | | | | | | | | |
| 1538 | 207610\_s\_at | EMR2 | -2.074224061 | 7.963565769 | -5.45791589 | 2.38E-005 | 0.000268541 | egf-like module containing, mucin-like, hormone receptor-like 2 | neuropeptide signaling pathway |  | | | | | | | | | | | | | | | | | | | | | | | | | | | |
| 1539 | 221483\_s\_at | ARPP-19 | 0.826339522 | 9.092882718 | 5.45741908 | 2.38E-005 | 0.000268706 | cyclic AMP phosphoprotein, 19 kD | positive regulation of gluconeogenesis, positive regulation of glucose import |  | | | | | | | | | | | | | | | | | | | | | | | | | | | |
| 1540 | 204276\_at | TK2 | -0.652354024 | 6.055841128 | -5.45645706 | 2.39E-005 | 0.000269019 | thymidine kinase 2, mitochondrial | DNA metabolism, nucleobase, nucleoside, nucleotide and nucleic acid metabolism |  | | | | | | | | | | | | | | | | | | | | | | | | | | | |
| 1541 | 215458\_s\_at | SMURF1 | 0.419880913 | 6.005726334 | 5.45615238 | 2.39E-005 | 0.000269068 | SMAD specific E3 ubiquitin protein ligase 1 | cell differentiation, ectoderm development, negative regulation of BMP signaling pathway, protein ubiquitination during ubiquitin-dependent protein catabolism, ubiquitin cycle |  | | | | | | | | | | | | | | | | | | | | | | | | | | | |
| 1542 | 218336\_at | PFDN2 | 1.419004265 | 8.982642624 | 5.4556319 | 2.39E-005 | 0.000269248 | prefoldin 2 | protein folding |  | | | | | | | | | | | | | | | | | | | | | | | | | | | |
| 1543 | 215567\_at | C14orf111 | -0.414773841 | 4.244496973 | -5.4535354 | 2.40E-005 | 0.000270255 | chromosome 14 open reading frame 111 | NA |  | | | | | | | | | | | | | | | | | | | | | | | | | | | |
| 1544 | 218252\_at | CKAP2 | 1.387556449 | 6.882404341 | 5.45246093 | 2.41E-005 | 0.000270776 | cytoskeleton associated protein 2 | NA |  | | | | | | | | | | | | | | | | | | | | | | | | | | | |
| 1545 | 210534\_s\_at | EPPB9 | 0.519603138 | 5.605618297 | 5.45030712 | 2.42E-005 | 0.000271936 | B9 protein | NA |  | | | | | | | | | | | | | | | | | | | | | | | | | | | |
| 1546 | 202167\_s\_at | MMS19L | 0.947606206 | 8.050399319 | 5.45012626 | 2.42E-005 | 0.000271936 | MMS19-like (MET18 homolog, S. cerevisiae) | nucleotide-excision repair, positive regulation of transcription, DNA-dependent, response to UV, response to hormone stimulus, response to organic substance, transcription, two-component signal transduction system (phosphorelay) |  | | | | | | | | | | | | | | | | | | | | | | | | | | | |
| 1547 | 222360\_at | CGI-30 | 1.043956704 | 4.301375819 | 5.44866593 | 2.43E-005 | 0.000272698 | CGI-30 protein | metabolism, peptidyl-diphthamide biosynthesis from peptidyl-histidine |  | | | | | | | | | | | | | | | | | | | | | | | | | | | |
| 1548 | 202781\_s\_at | SKIP | -0.483444532 | 7.66437419 | -5.44802993 | 2.43E-005 | 0.000272953 | skeletal muscle and kidney enriched inositol phosphatase | NA |  | | | | | | | | | | | | | | | | | | | | | | | | | | | |
| 1549 | 220183\_s\_at | NUDT6 | 0.953303798 | 4.912391617 | 5.44532698 | 2.45E-005 | 0.000274352 | nudix (nucleoside diphosphate linked moiety X)-type motif 6 | NA |  | | | | | | | | | | | | | | | | | | | | | | | | | | | |
| 1550 | 217749\_at | COPG | 1.001751948 | 7.421795215 | 5.44506629 | 2.45E-005 | 0.000274376 | coatomer protein complex, subunit gamma | NA |  | | | | | | | | | | | | | | | | | | | | | | | | | | | |
| 1551 | 210589\_s\_at | GBA /// GBAP | -1.116084157 | 8.218000492 | -5.44481039 | 2.45E-005 | 0.000274396 | glucosidase, beta; acid (includes glucosylceramidase) /// glucosidase, beta; acid, pseudogene | carbohydrate metabolism, lysosome organization and biogenesis, sphingolipid metabolism |  | | | | | | | | | | | | | | | | | | | | | | | | | | | |
| 1552 | 218607\_s\_at | SDAD1 | 0.971758492 | 7.796813986 | 5.43964317 | 2.48E-005 | 0.000277218 | SDA1 domain containing 1 | NA |  | | | | | | | | | | | | | | | | | | | | | | | | | | | |
| 1553 | 215096\_s\_at | ESD | 1.379335489 | 9.041143968 | 5.4390844 | 2.48E-005 | 0.000277279 | esterase D/formylglutathione hydrolase | NA |  | | | | | | | | | | | | | | | | | | | | | | | | | | | |
| 1554 | 200006\_at | PARK7 | 0.961270089 | 11.07743842 | 5.43617711 | 2.50E-005 | 0.000278577 | Parkinson disease (autosomal recessive, early onset) 7 /// Parkinson disease (autosomal recessive, early onset) 7 | NA |  | | | | | | | | | | | | | | | | | | | | | | | | | | | |
| 1555 | 214150\_x\_at | ATP6V0E | -0.962375215 | 9.485077961 | -5.43613513 | 2.50E-005 | 0.000278577 | ATPase, H+ transporting, lysosomal 9kDa, V0 subunit e | ATP synthesis coupled proton transport, proton transport |  | | | | | | | | | | | | | | | | | | | | | | | | | | | |
| 1556 | 204279\_at | PSMB9 | -2.098526287 | 8.672106696 | -5.43494678 | 2.50E-005 | 0.000279048 | proteasome (prosome, macropain) subunit, beta type, 9 (large multifunctional protease 2) | immune response, proteolysis and peptidolysis, ubiquitin-dependent protein catabolism |  | | | | | | | | | | | | | | | | | | | | | | | | | | | |
| 1557 | 202008\_s\_at | NID | -1.058552404 | 6.076794167 | -5.4341835 | 2.51E-005 | 0.000279391 | nidogen (enactin) | cell adhesion, cell-matrix adhesion |  | | | | | | | | | | | | | | | | | | | | | | | | | | | |
| 1558 | 218946\_at | HIRIP5 | 0.918981496 | 7.498080572 | 5.43272901 | 2.52E-005 | 0.000280032 | HIRA interacting protein 5 | NA |  | | | | | | | | | | | | | | | | | | | | | | | | | | | |
| 1559 | 204882\_at | ARHGAP25 | -1.457363902 | 8.895345069 | -5.43205575 | 2.52E-005 | 0.00028032 | Rho GTPase activating protein 25 | NA |  | | | | | | | | | | | | | | | | | | | | | | | | | | | |
| 1560 | 205164\_at | GCAT | 1.195730153 | 7.725477164 | 5.43042626 | 2.53E-005 | 0.000281061 | glycine C-acetyltransferase (2-amino-3-ketobutyrate coenzyme A ligase) | amino acid metabolism, biosynthesis, heme biosynthesis |  | | | | | | | | | | | | | | | | | | | | | | | | | | | |
| 1561 | 209624\_s\_at | MCCC2 | 1.024920245 | 6.917600404 | 5.43004363 | 2.53E-005 | 0.000281061 | methylcrotonoyl-Coenzyme A carboxylase 2 (beta) | leucine catabolism |  | | | | | | | | | | | | | | | | | | | | | | | | | | | |
| 1562 | 209341\_s\_at | IKBKB | -1.00587125 | 8.152687829 | -5.42945172 | 2.54E-005 | 0.000281179 | inhibitor of kappa light polypeptide gene enhancer in B-cells, kinase beta | protein amino acid phosphorylation |  | | | | | | | | | | | | | | | | | | | | | | | | | | | |
| 1563 | 213737\_x\_at | DKFZp434P162 | -1.928929898 | 9.485646647 | -5.42938129 | 2.54E-005 | 0.000281179 | hypothetical protein DKFZp434P162 | NA |  | | | | | | | | | | | | | | | | | | | | | | | | | | | |
| 1564 | 208310\_s\_at | C7orf28B /// C7orf28A | 1.254571647 | 7.450311852 | 5.42874884 | 2.54E-005 | 0.000281442 | chromosome 7 open reading frame 28B /// chromosome 7 open reading frame 28A | NA |  | | | | | | | | | | | | | | | | | | | | | | | | | | | |
| 1565 | 215215\_s\_at | LOC81691 | 1.352338259 | 6.576963978 | 5.42812668 | 2.54E-005 | 0.000281618 | exonuclease NEF-sp | NA |  | | | | | | | | | | | | | | | | | | | | | | | | | | | |
| 1566 | 204120\_s\_at | ADK | 0.634534616 | 4.181071873 | 5.42793587 | 2.54E-005 | 0.000281618 | adenosine kinase | purine ribonucleoside salvage, ribonucleoside monophosphate biosynthesis |  | | | | | | | | | | | | | | | | | | | | | | | | | | | |
| 1567 | 218771\_at | PANK4 | 0.505609637 | 7.015139699 | 5.42781383 | 2.55E-005 | 0.000281618 | pantothenate kinase 4 | coenzyme A biosynthesis |  | | | | | | | | | | | | | | | | | | | | | | | | | | | |
| 1568 | 209827\_s\_at | IL16 | -1.387423754 | 7.956260384 | -5.42730627 | 2.55E-005 | 0.000281802 | interleukin 16 (lymphocyte chemoattractant factor) | chemotaxis, immune response |  | | | | | | | | | | | | | | | | | | | | | | | | | | | |
| 1569 | 208910\_s\_at | C1QBP | 2.346649039 | 8.752178076 | 5.42706687 | 2.55E-005 | 0.000281814 | complement component 1, q subcomponent binding protein | immune response |  | | | | | | | | | | | | | | | | | | | | | | | | | | | |
| 1570 | 204407\_at | TTF2 | 1.121063774 | 4.658400929 | 5.42602071 | 2.56E-005 | 0.000282234 | transcription termination factor, RNA polymerase II | transcription termination |  | | | | | | | | | | | | | | | | | | | | | | | | | | | |
| 1571 | 219435\_at | FLJ22170 | -0.572346226 | 7.361047123 | -5.42597166 | 2.56E-005 | 0.000282234 | hypothetical protein FLJ22170 | NA |  | | | | | | | | | | | | | | | | | | | | | | | | | | | |
| 1572 | 221794\_at | DOCK6 | 0.512280926 | 7.372053584 | 5.42527036 | 2.56E-005 | 0.000282402 | dedicator of cytokinesis 6 | NA |  | | | | | | | | | | | | | | | | | | | | | | | | | | | |
| 1573 | 214551\_s\_at | CD7 | -0.821925331 | 5.093605268 | -5.42405924 | 2.57E-005 | 0.000282898 | CD7 antigen (p41) | T-cell activation, calcium ion transport, cellular defense response, transmembrane receptor protein tyrosine kinase signaling pathway |  | | | | | | | | | | | | | | | | | | | | | | | | | | | |
| 1574 | 203576\_at | BCAT2 | 0.64804032 | 7.566255834 | 5.421379 | 2.58E-005 | 0.000284481 | branched chain aminotransferase 2, mitochondrial | branched chain family amino acid biosynthesis, metabolism |  | | | | | | | | | | | | | | | | | | | | | | | | | | | |
| 1575 | 200094\_s\_at | EEF2 | 1.38256166 | 11.82138497 | 5.42003916 | 2.59E-005 | 0.000285205 | eukaryotic translation elongation factor 2 /// eukaryotic translation elongation factor 2 | protein biosynthesis, translational elongation |  | | | | | | | | | | | | | | | | | | | | | | | | | | | |
| 1576 | 212815\_at | ASCC3 | 1.33587654 | 7.27864899 | 5.41788861 | 2.60E-005 | 0.000286315 | activating signal cointegrator 1 complex subunit 3 | regulation of transcription, DNA-dependent |  | | | | | | | | | | | | | | | | | | | | | | | | | | | |
| 1577 | 219320\_at | MYOHD1 | 0.628514417 | 7.567769733 | 5.41683331 | 2.61E-005 | 0.000286717 | myosin head domain containing 1 | NA |  | | | | | | | | | | | | | | | | | | | | | | | | | | | |
| 1578 | 218188\_s\_at | TIMM13 | 1.752218892 | 8.257427751 | 5.4162746 | 2.61E-005 | 0.000286893 | translocase of inner mitochondrial membrane 13 homolog (yeast) | mitochondrial inner membrane protein import, perception of sound, protein transport |  | | | | | | | | | | | | | | | | | | | | | | | | | | | |
| 1579 | 202768\_at | FOSB | -2.751253281 | 8.619943708 | -5.41601619 | 2.61E-005 | 0.000286893 | FBJ murine osteosarcoma viral oncogene homolog B | behavior, development, negative regulation of transcription from Pol II promoter, regulation of cell cycle, regulation of transcription, DNA-dependent |  | | | | | | | | | | | | | | | | | | | | | | | | | | | |
| 1580 | 203046\_s\_at | TIMELESS | 0.888603448 | 7.342439748 | 5.41512741 | 2.62E-005 | 0.000287118 | timeless homolog (Drosophila) | circadian rhythm, detection of abiotic stimulus |  | | | | | | | | | | | | | | | | | | | | | | | | | | | |
| 1581 | 213452\_at | ZNF184 | 1.149282053 | 5.524676098 | 5.41336144 | 2.63E-005 | 0.000287844 | zinc finger protein 184 (Kruppel-like) | NA |  | | | | | | | | | | | | | | | | | | | | | | | | | | | |
| 1582 | 212451\_at | KIAA0256 | -0.751736707 | 6.491243761 | -5.41169002 | 2.64E-005 | 0.000288653 | KIAA0256 gene product | NA |  | | | | | | | | | | | | | | | | | | | | | | | | | | | |
| 1583 | 214264\_s\_at | C14orf143 | 0.617709815 | 6.067181541 | 5.40575307 | 2.68E-005 | 0.000291992 | chromosome 14 open reading frame 143 | NA |  | | | | | | | | | | | | | | | | | | | | | | | | | | | |
| 1584 | 206566\_at | SLC7A1 | 1.431849405 | 5.852404576 | 5.40444956 | 2.68E-005 | 0.00029257 | solute carrier family 7 (cationic amino acid transporter, y+ system), member 1 | amino acid metabolism, amino acid transport, transport |  | | | | | | | | | | | | | | | | | | | | | | | | | | | |
| 1585 | 201012\_at | ANXA1 | -2.51316599 | 11.24833993 | -5.40395246 | 2.69E-005 | 0.000292702 | annexin A1 | cell motility, cell surface receptor linked signal transduction, inflammatory response, lipid metabolism |  | | | | | | | | | | | | | | | | | | | | | | | | | | | |
| 1586 | 219303\_at | C13orf7 | 1.438806971 | 5.968902065 | 5.40381712 | 2.69E-005 | 0.000292702 | chromosome 13 open reading frame 7 | NA |  | | | | | | | | | | | | | | | | | | | | | | | | | | | |
| 1587 | 207667\_s\_at | MAP2K3 | -0.831218741 | 8.988584853 | -5.40288659 | 2.69E-005 | 0.000293034 | mitogen-activated protein kinase kinase 3 | protein amino acid phosphorylation, signal transduction |  | | | | | | | | | | | | | | | | | | | | | | | | | | | |
| 1588 | 219315\_s\_at | C16orf30 | -0.665576545 | 6.651187554 | -5.40242091 | 2.70E-005 | 0.0002932 | chromosome 16 open reading frame 30 | NA |  | | | | | | | | | | | | | | | | | | | | | | | | | | | |
| 1589 | 201284\_s\_at | APEH | 1.068489568 | 8.53462562 | 5.40173048 | 2.70E-005 | 0.000293516 | N-acylaminoacyl-peptide hydrolase | proteolysis and peptidolysis |  | | | | | | | | | | | | | | | | | | | | | | | | | | | |
| 1590 | 219193\_at | FLJ10233 | 0.67635022 | 7.542072436 | 5.3995749 | 2.71E-005 | 0.000294811 | hypothetical protein FLJ10233 | NA |  | | | | | | | | | | | | | | | | | | | | | | | | | | | |
| 1591 | 200911\_s\_at | TACC1 | -0.869174642 | 9.383644242 | -5.39820482 | 2.72E-005 | 0.000295532 | transforming, acidic coiled-coil containing protein 1 | NA |  | | | | | | | | | | | | | | | | | | | | | | | | | | | |
| 1592 | 209088\_s\_at | UBN1 | -1.427916293 | 8.113502173 | -5.39792707 | 2.72E-005 | 0.000295532 | ubinuclein 1 | regulation of transcription from Pol II promoter |  | | | | | | | | | | | | | | | | | | | | | | | | | | | |
| 1593 | 203531\_at | CUL5 | 0.941737776 | 7.166570383 | 5.39785173 | 2.72E-005 | 0.000295532 | Cullin 5 | G1/S transition of mitotic cell cycle, cell cycle arrest, induction of apoptosis by intracellular signals, negative regulation of cell proliferation, ubiquitin cycle |  | | | | | | | | | | | | | | | | | | | | | | | | | | | |
| 1594 | 202077\_at | NDUFAB1 | 1.291728794 | 9.819525092 | 5.39715437 | 2.73E-005 | 0.000295856 | NADH dehydrogenase (ubiquinone) 1, alpha/beta subcomplex, 1, 8kDa | fatty acid biosynthesis |  | | | | | | | | | | | | | | | | | | | | | | | | | | | |
| 1595 | 201578\_at | PODXL | 0.363033751 | 4.985404514 | 5.39529515 | 2.74E-005 | 0.000296962 | podocalyxin-like | cell growth and/or maintenance |  | | | | | | | | | | | | | | | | | | | | | | | | | | | |
| 1596 | 218193\_s\_at | CGI-141 | 1.31778683 | 6.901724041 | 5.39288967 | 2.76E-005 | 0.000298386 | CGI-141 protein | positive regulation of I-kappaB kinase/NF-kappaB cascade, vesicle-mediated transport |  | | | | | | | | | | | | | | | | | | | | | | | | | | | |
| 1597 | 206851\_at | RNASE3 | -2.839736547 | 9.639784343 | -5.39275841 | 2.76E-005 | 0.000298386 | ribonuclease, RNase A family, 3 (eosinophil cationic protein) | RNA catabolism, defense response to bacteria |  | | | | | | | | | | | | | | | | | | | | | | | | | | | |
| 1598 | 212846\_at | KIAA0179 | 1.851582943 | 7.818130704 | 5.38973865 | 2.78E-005 | 0.0003 | KIAA0179 | superoxide metabolism |  | | | | | | | | | | | | | | | | | | | | | | | | | | | |
| 1599 | 218542\_at | C10orf3 | 1.655169105 | 6.160500945 | 5.3892692 | 2.78E-005 | 0.000300174 | chromosome 10 open reading frame 3 | NA |  | | | | | | | | | | | | | | | | | | | | | | | | | | | |
| 1600 | 219165\_at | PDLIM2 | -1.613741199 | 7.379413312 | -5.38823682 | 2.78E-005 | 0.000300404 | PDZ and LIM domain 2 (mystique) | NA |  | | | | | | | | | | | | | | | | | | | | | | | | | | | |
| 1601 | 200030\_s\_at | SLC25A3 | 1.129512012 | 11.98666379 | 5.38807163 | 2.79E-005 | 0.000300404 | solute carrier family 25 (mitochondrial carrier; phosphate carrier), member 3 /// solute carrier family 25 (mitochondrial carrier; phosphate carrier), member 3 | energy pathways, transport |  | | | | | | | | | | | | | | | | | | | | | | | | | | | |
| 1602 | 219306\_at | KNSL7 | 1.43642462 | 6.464990476 | 5.38753254 | 2.79E-005 | 0.000300404 | kinesin-like 7 | cell proliferation, mitosis |  | | | | | | | | | | | | | | | | | | | | | | | | | | | |
| 1603 | 200818\_at | ATP5O | 1.142734702 | 10.59646357 | 5.3874394 | 2.79E-005 | 0.000300404 | ATP synthase, H+ transporting, mitochondrial F1 complex, O subunit (oligomycin sensitivity conferring protein) | ATP synthesis coupled proton transport, proton transport |  | | | | | | | | | | | | | | | | | | | | | | | | | | | |
| 1604 | 203140\_at | BCL6 | -3.473588093 | 8.298469881 | -5.38743401 | 2.79E-005 | 0.000300404 | B-cell CLL/lymphoma 6 (zinc finger protein 51) /// B-cell CLL/lymphoma 6 (zinc finger protein 51) | cell growth and/or maintenance, inflammatory response, negative regulation of transcription from Pol II promoter, positive regulation of cell proliferation, regulation of transcription, DNA-dependent |  | | | | | | | | | | | | | | | | | | | | | | | | | | | |
| 1605 | 212792\_at | KIAA0877 | 0.724592004 | 6.761692183 | 5.3837944 | 2.81E-005 | 0.000302604 | KIAA0877 protein | NA |  | | | | | | | | | | | | | | | | | | | | | | | | | | | |
| 1606 | 219176\_at | FLJ22555 | 1.405540943 | 7.941563203 | 5.38339232 | 2.82E-005 | 0.00030269 | hypothetical protein FLJ22555 | NA |  | | | | | | | | | | | | | | | | | | | | | | | | | | | |
| 1607 | 201473\_at | JUNB | -1.854125419 | 9.72483173 | -5.38324295 | 2.82E-005 | 0.00030269 | jun B proto-oncogene | regulation of transcription from Pol II promoter |  | | | | | | | | | | | | | | | | | | | | | | | | | | | |
| 1608 | 220762\_s\_at | GNB1L | 1.119987016 | 7.558151021 | 5.38289917 | 2.82E-005 | 0.00030278 | guanine nucleotide binding protein (G protein), beta polypeptide 1-like | G-protein coupled receptor protein signaling pathway, intracellular signaling cascade |  | | | | | | | | | | | | | | | | | | | | | | | | | | | |
| 1609 | 200735\_x\_at | NACA | 0.683280505 | 12.46931372 | 5.37775946 | 2.85E-005 | 0.000305744 | nascent-polypeptide-associated complex alpha polypeptide | nascent polypeptide association, protein biosynthesis |  | | | | | | | | | | | | | | | | | | | | | | | | | | | |
| 1610 | 217491\_x\_at | COX7C | 0.81848052 | 10.72229823 | 5.3760482 | 2.86E-005 | 0.000306638 | cytochrome c oxidase subunit VIIc | electron transport, energy pathways |  | | | | | | | | | | | | | | | | | | | | | | | | | | | |
| 1611 | 219625\_s\_at | COL4A3BP | -1.038286103 | 7.505842298 | -5.37512615 | 2.87E-005 | 0.000307133 | collagen, type IV, alpha 3 (Goodpasture antigen) binding protein | immune response, protein amino acid phosphorylation |  | | | | | | | | | | | | | | | | | | | | | | | | | | | |
| 1612 | 213034\_at | KIAA0999 | -0.856749774 | 8.13799578 | -5.37154219 | 2.89E-005 | 0.000309346 | KIAA0999 protein | protein amino acid phosphorylation |  | | | | | | | | | | | | | | | | | | | | | | | | | | | |
| 1613 | 203230\_at | DVL1 | 1.119871548 | 7.153417286 | 5.36998804 | 2.90E-005 | 0.000310142 | dishevelled, dsh homolog 1 (Drosophila) | development, frizzled signaling pathway, heart development, intracellular signaling cascade, morphogenesis |  | | | | | | | | | | | | | | | | | | | | | | | | | | | |
| 1614 | 212591\_at | KIAA0117 | 1.268150889 | 8.438657763 | 5.36967815 | 2.90E-005 | 0.000310211 | KIAA0117 protein | NA |  | | | | | | | | | | | | | | | | | | | | | | | | | | | |
| 1615 | 208184\_s\_at | TMEM1 | -0.387383462 | 6.256483897 | -5.36934999 | 2.91E-005 | 0.000310293 | transmembrane protein 1 | sodium ion transport, transport |  | | | | | | | | | | | | | | | | | | | | | | | | | | | |
| 1616 | 218276\_s\_at | SAV1 | 0.955613968 | 5.067184446 | 5.36521715 | 2.93E-005 | 0.00031292 | salvador homolog 1 (Drosophila) | signal transduction |  | | | | | | | | | | | | | | | | | | | | | | | | | | | |
| 1617 | 201931\_at | ETFA | 1.831650419 | 8.892353258 | 5.36483646 | 2.94E-005 | 0.000313033 | electron-transfer-flavoprotein, alpha polypeptide (glutaric aciduria II) | electron transport |  | | | | | | | | | | | | | | | | | | | | | | | | | | | |
| 1618 | 220560\_at | C11orf21 | -2.023867764 | 8.158865321 | -5.36442531 | 2.94E-005 | 0.000313033 | chromosome 11 open reading frame 21 | NA |  | | | | | | | | | | | | | | | | | | | | | | | | | | | |
| 1619 | 219577\_s\_at | ABCA7 | -1.199069002 | 7.412404113 | -5.36365426 | 2.94E-005 | 0.000313432 | ATP-binding cassette, sub-family A (ABC1), member 7 | transport |  | | | | | | | | | | | | | | | | | | | | | | | | | | | |
| 1620 | 209929\_s\_at | IKBKG | -0.503163271 | 7.977422612 | -5.35873277 | 2.98E-005 | 0.000316232 | inhibitor of kappa light polypeptide gene enhancer in B-cells, kinase gamma | I-kappaB kinase/NF-kappaB cascade, immune response, induction of apoptosis, regulation of transcription, DNA-dependent |  | | | | | | | | | | | | | | | | | | | | | | | | | | | |
| 1621 | 217862\_at | PIAS1 | -0.880628459 | 7.72842456 | -5.35868843 | 2.98E-005 | 0.000316232 | protein inhibitor of activated STAT, 1 | JAK-STAT cascade, signal transduction |  | | | | | | | | | | | | | | | | | | | | | | | | | | | |
| 1622 | 213360\_s\_at | POM121 /// LOC340318 | 0.74675698 | 10.01410779 | 5.35727275 | 2.99E-005 | 0.000316949 | POM121 membrane glycoprotein (rat) /// hypothetical protein LOC340318 | transport |  | | | | | | | | | | | | | | | | | | | | | | | | | | | |
| 1623 | 206316\_s\_at | KNTC1 | 0.898805494 | 7.238364096 | 5.35441179 | 3.01E-005 | 0.000318757 | kinetochore associated 1 | cell cycle, mitosis, mitotic checkpoint, protein complex assembly, regulation of exit from mitosis |  | | | | | | | | | | | | | | | | | | | | | | | | | | | |
| 1624 | 213758\_at | COX4I1 | -0.793413016 | 7.239253934 | -5.35435135 | 3.01E-005 | 0.000318757 | Cytochrome c oxidase subunit IV isoform 1 | electron transport, energy pathways |  | | | | | | | | | | | | | | | | | | | | | | | | | | | |
| 1625 | 201790\_s\_at | DHCR7 | 1.508578654 | 8.311263566 | 5.35246159 | 3.02E-005 | 0.000319824 | 7-dehydrocholesterol reductase | cholesterol biosynthesis |  | | | | | | | | | | | | | | | | | | | | | | | | | | | |
| 1626 | 202246\_s\_at | CDK4 | 1.62197252 | 9.529796711 | 5.34884478 | 3.05E-005 | 0.000322312 | cyclin-dependent kinase 4 | G1/S transition of mitotic cell cycle, cytokinesis, protein amino acid phosphorylation, regulation of cell cycle |  | | | | | | | | | | | | | | | | | | | | | | | | | | | |
| 1627 | 209893\_s\_at | FUT4 | -1.642033393 | 9.052409196 | -5.3478411 | 3.05E-005 | 0.000322895 | fucosyltransferase 4 (alpha (1,3) fucosyltransferase, myeloid-specific) | L-fucose catabolism, carbohydrate metabolism, protein amino acid glycosylation |  | | | | | | | | | | | | | | | | | | | | | | | | | | | |
| 1628 | 209760\_at | KIAA0922 | -0.864560759 | 8.224435242 | -5.34753243 | 3.05E-005 | 0.000322968 | KIAA0922 protein | NA |  | | | | | | | | | | | | | | | | | | | | | | | | | | | |
| 1629 | 220094\_s\_at | C6orf79 | 1.823262609 | 7.821453775 | 5.34717837 | 3.06E-005 | 0.000323074 | chromosome 6 open reading frame 79 | NA |  | | | | | | | | | | | | | | | | | | | | | | | | | | | |
| 1630 | 209606\_at | PSCDBP | -2.433683763 | 7.121541108 | -5.34532378 | 3.07E-005 | 0.000324285 | pleckstrin homology, Sec7 and coiled-coil domains, binding protein /// pleckstrin homology, Sec7 and coiled-coil domains, binding protein | NA |  | | | | | | | | | | | | | | | | | | | | | | | | | | | |
| 1631 | 213073\_at | ZFYVE26 | -0.760181693 | 6.904731962 | -5.34371689 | 3.08E-005 | 0.000325318 | zinc finger, FYVE domain containing 26 | NA |  | | | | | | | | | | | | | | | | | | | | | | | | | | | |
| 1632 | 212334\_at | GNS | -1.423437538 | 9.107322802 | -5.34187324 | 3.09E-005 | 0.00032653 | glucosamine (N-acetyl)-6-sulfatase (Sanfilippo disease IIID) | glycosaminoglycan catabolism, metabolism |  | | | | | | | | | | | | | | | | | | | | | | | | | | | |
| 1633 | 220948\_s\_at | ATP1A1 | 1.012737494 | 9.060692536 | 5.3409887 | 3.10E-005 | 0.000327033 | ATPase, Na+/K+ transporting, alpha 1 polypeptide | ATP hydrolysis coupled proton transport, hydrogen ion homeostasis, metabolism, potassium ion transport, sodium ion transport, sperm motility |  | | | | | | | | | | | | | | | | | | | | | | | | | | | |
[truncated: 2,797,235 more chars]
