# Supplementary material for: Gene expression profiling reveals consistent differences between clinical samples of human leukaemias and their model cell lines
Source: Br J Haematol. 2006 Nov;135(4):520–3. doi: 10.1111/j.1365-2141.2006.06342.x (PMC1654200; doi:10.1111/j.1365-2141.2006.06342.x)
Supplement: Table SII — GO analysis of top 1000 upregulated genes (overexpression in cell lines compared with clinical samples). [file bjh0135-0520-TableSII.html]

GOstat by Tim Beissbarth


|  |  |  |  |
| --- | --- | --- | --- |
| *GO*stat *by Tim Bei�barth beissbarth@wehi.edu.au* | Run from 130.223.122.125 Date: Wed Apr 26 01:20:08 2006 GO Annotation @ EBI (GOA) Input: 1000 IDs Search against: AFFY\_HG\_U133A | Unique Genes: 1000 Annotated Genes: 576 GOs: 3740 Unique GOs: 962 All Unique Sub-GOs: 1079 | GO-DB: goa\_human Min Sub-GO length: 1 P-Value Cutoff: 0.01 GO-Cluster Cutoff: 0 Correct-Method: Yekutieli |

|  |  |  |  |  |  |
| --- | --- | --- | --- | --- | --- |
| **P-value cutoff:**  1e-101e-50.00010.0010.010.10.30.51 | **Show best:**  110305010010000 | **Indication:**  Over- and UnderrepresentedShow Overrepresented onlyShow Underrepresented only | **Cluster GOs:**  -101234510 | **Display:**  HTML, Stats and Go AnnotationHTML, GO Stats onlyHTML, GO Annotation onlyTEXT, Stats and Go AnnotationTEXT, GO Stats onlyTEXT, GO Annotation only |  |

---

|  |  |  |  |  |  |  |  |  |  |  |  |  |  |  |  |  |  |  |  |  |  |  |  |  |  |  |  |  |  |  |  |  |  |  |  |  |  |  |  |  |  |  |  |  |  |  |  |  |  |  |  |  |  |  |  |  |  |  |  |  |  |  |  |  |  |  |  |  |  |  |  |  |  |  |  |  |  |  |  |  |  |  |  |  |  |  |  |  |  |  |  |  |  |  |  |  |  |  |  |  |  |  |  |  |  |  |  |  |  |  |  |  |  |  |  |  |  |  |  |  |  |  |  |  |  |  |  |  |  |  |  |  |  |  |  |  |  |  |  |  |  |  |  |  |  |  |  |  |  |  |  |  |  |  |  |  |  |  |  |  |  |  |  |  |  |  |  |  |  |  |  |  |  |  |  |  |  |  |  |  |  |  |  |  |  |  |  |  |  |  |  |  |  |  |  |  |  |  |  |  |  |  |  |  |  |  |  |  |  |  |  |  |  |  |  |  |  |  |  |  |  |  |  |  |  |  |  |  |  |  |  |  |  |  |  |  |  |  |  |  |  |  |  |  |  |  |  |  |  |  |  |  |  |  |  |  |  |  |  |  |  |  |  |  |  |  |  |  |  |  |  |  |  |  |  |  |  |  |  |  |  |  |  |  |  |  |  |  |  |  |  |  |  |  |  |  |  |  |  |  |  |  |  |  |  |  |  |  |  |  |  |  |  |  |  |  |  |  |  |  |  |  |  |  |  |  |  |  |  |  |  |  |  |  |  |  |  |  |  |  |  |  |  |  |  |  |  |  |  |  |  |  |  |  |  |  |  |  |  |  |  |  |  |  |  |  |  |  |  |  |  |  |  |  |  |  |  |  |  |  |  |  |  |  |  |  |  |  |  |  |  |  |  |  |  |  |  |  |  |  |  |  |  |  |  |  |  |  |  |  |  |  |  |  |  |  |  |  |  |  |  |  |  |  |  |  |  |  |  |  |  |  |  |  |  |  |  |  |  |  |  |  |  |  |  |  |  |  |  |  |  |  |  |  |  |  |  |  |  |  |  |  |  |  |  |  |  |  |  |  |  |  |  |  |  |  |  |  |  |  |  |  |  |  |  |  |  |  |  |  |  |  |  |  |  |  |  |  |  |  |  |  |  |  |  |  |  |  |  |  |  |  |  |  |  |  |  |  |  |  |  |  |  |  |  |  |  |  |  |  |  |  |  |  |  |  |  |  |  |  |  |  |  |  |  |  |  |  |  |  |  |  |  |  |  |  |  |  |  |  |  |  |  |  |  |  |  |  |  |  |  |  |  |  |  |  |  |  |  |  |  |  |  |  |  |  |  |  |  |  |  |  |  |  |  |  |  |  |  |  |  |  |  |  |  |  |  |  |  |  |  |  |  |  |  |  |  |  |  |  |  |  |  |  |  |  |  |  |  |  |  |  |  |  |  |  |  |  |  |  |  |  |  |  |  |  |  |  |  |  |  |  |  |  |  |  |  |  |  |  |  |  |  |  |  |  |  |  |  |  |  |  |  |  |  |  |  |  |  |  |  |  |  |  |  |  |  |  |  |  |  |  |  |  |  |  |  |  |  |  |  |  |  |  |  |  |  |  |  |  |  |  |  |  |  |  |  |  |  |  |  |  |  |  |  |  |  |  |  |  |  |  |  |  |  |  |  |  |  |  |  |  |  |  |  |  |  |  |  |  |  |  |  |  |  |  |  |  |  |  |  |  |  |  |  |  |  |  |  |  |  |  |  |  |  |  |  |  |  |  |  |  |  |  |  |  |  |  |  |  |  |  |  |  |  |  |  |  |  |  |  |  |  |  |  |  |  |  |  |  |  |  |  |  |  |  |  |  |  |  |  |  |  |  |  |  |  |  |  |  |  |  |  |  |  |  |  |  |  |  |  |  |  |  |  |  |  |  |  |  |  |  |  |  |  |  |  |  |  |  |  |  |  |  |  |  |  |  |  |  |  |  |  |  |  |  |  |  |  |  |  |  |  |  |  |  |  |  |  |  |  |  |  |  |  |  |  |  |  |  |  |  |  |  |  |  |  |  |  |  |  |  |  |  |  |  |  |  |  |  |  |  |  |  |  |  |  |  |  |  |  |  |  |  |  |  |  |  |  |  |  |  |  |  |  |  |  |  |  |  |  |  |  |  |  |  |  |  |  |  |  |  |  |  |  |  |  |  |  |  |  |  |  |  |  |  |  |  |  |  |  |  |  |  |  |  |  |  |  |  |  |  |  |  |  |  |  |  |  |  |  |  |  |  |  |  |  |  |  |  |  |  |  |  |  |  |  |  |  |  |  |  |  |  |  |  |  |  |  |  |  |  |  |  |  |  |  |  |  |  |  |  |  |  |  |  |  |  |  |  |  |  |  |  |  |  |  |  |  |  |  |  |  |  |  |  |  |  |  |  |  |  |  |  |  |  |  |  |  |  |  |  |  |  |  |  |  |  |  |  |  |  |  |  |  |  |  |  |  |  |  |  |  |  |  |  |  |  |  |  |  |  |  |  |  |  |  |  |  |  |  |  |  |  |  |  |  |  |  |  |  |  |  |  |  |  |  |  |  |  |  |  |  |  |  |  |  |  |  |  |  |  |  |  |  |  |  |  |  |  |  |  |  |  |  |  |  |  |  |  |  |  |  |  |  |  |  |  |  |  |  |  |  |  |  |  |  |  |  |  |  |  |  |  |  |  |  |  |  |  |  |  |  |  |  |  |  |  |  |  |  |  |  |  |  |  |  |  |  |  |  |  |  |  |  |  |  |  |  |  |  |  |  |  |  |  |  |  |  |  |  |  |  |  |  |  |  |  |  |  |  |  |  |  |  |  |  |  |  |  |  |  |  |  |  |  |  |  |  |  |  |  |  |  |  |  |  |  |  |  |  |  |  |  |  |  |  |  |  |  |  |  |  |  |  |  |  |  |  |  |  |  |  |  |  |  |  |  |  |  |  |  |  |  |  |  |  |  |  |  |  |  |  |  |  |  |  |  |  |  |  |  |  |  |  |  |  |  |  |  |  |  |  |  |  |  |  |  |  |  |  |  |  |  |  |  |  |  |  |  |  |  |  |  |  |  |  |  |  |  |  |  |  |  |  |  |  |  |  |  |  |  |  |  |  |  |  |  |  |  |  |  |  |  |  |  |  |  |  |  |  |  |  |  |  |  |  |  |  |  |  |  |  |  |  |  |  |  |  |  |  |  |  |  |  |  |  |  |  |  |  |  |  |  |  |  |  |  |  |  |  |  |  |  |  |  |  |  |  |  |  |  |  |  |  |  |  |  |  |  |  |  |  |  |  |  |  |  |  |  |  |  |  |  |  |  |  |  |  |  |  |  |  |  |  |  |  |  |  |  |  |  |  |  |  |  |  |  |  |  |  |  |  |  |  |  |  |  |  |  |  |  |  |  |  |  |  |  |  |  |  |  |  |  |  |  |  |  |  |  |  |  |  |  |  |  |  |  |  |  |  |  |  |  |  |  |  |  |  |  |  |  |  |  |  |  |  |  |  |  |  |  |  |  |  |  |  |  |  |  |  |  |  |  |  |  |  |  |  |  |  |  |  |  |  |  |  |  |  |  |  |  |  |  |  |  |  |  |  |  |  |  |  |  |  |  |  |  |  |  |  |  |  |  |  |  |  |  |  |  |  |  |  |  |  |  |  |  |  |  |  |  |  |  |  |  |  |  |  |  |  |  |  |  |  |  |  |  |  |  |  |  |  |  |  |  |  |  |  |  |  |  |  |  |  |  |  |  |  |  |  |  |  |  |  |  |  |  |  |  |  |  |  |  |  |  |  |  |  |  |  |  |  |  |  |  |  |  |  |  |  |  |  |  |  |  |  |  |  |  |  |  |  |  |  |  |  |  |  |  |  |  |  |  |  |  |  |  |  |  |  |  |  |  |  |  |  |  |  |  |  |  |  |  |  |  |  |  |  |  |  |  |  |  |  |  |  |  |  |  |  |  |  |  |  |  |  |  |  |  |  |  |  |  |  |  |  |  |  |  |  |  |  |  |  |  |  |  |  |  |  |  |  |  |  |  |  |  |  |  |  |  |  |  |  |  |  |  |  |  |  |  |  |  |  |  |  |  |  |  |  |  |  |  |  |  |  |  |  |  |  |  |  |  |  |  |  |  |  |  |  |  |  |  |  |  |  |  |  |  |  |  |  |  |  |  |  |  |  |  |  |  |  |  |  |  |  |  |  |  |  |  |  |  |  |  |  |  |  |  |  |  |  |  |  |  |  |  |  |  |  |  |  |  |  |  |  |  |  |  |  |  |  |  |  |  |  |  |  |  |  |  |  |  |  |  |  |  |  |  |  |  |  |  |  |  |  |  |  |  |  |  |  |  |  |  |  |  |  |  |  |  |  |  |  |  |  |  |  |  |  |  |  |  |  |  |  |  |  |  |  |  |  |  |  |  |  |  |  |  |  |  |  |  |  |  |  |  |  |  |  |  |  |  |  |  |  |  |  |  |  |  |  |  |  |  |  |  |  |  |  |  |  |  |  |  |  |  |  |  |  |  |  |  |  |  |  |  |  |  |  |  |  |  |  |  |  |  |  |  |  |  |  |  |  |  |  |  |  |  |  |  |  |  |  |  |  |  |  |  |  |  |  |  |  |  |  |  |  |  |  |  |  |  |  |  |  |  |  |  |  |  |  |  |  |  |  |  |  |  |  |  |  |  |  |  |  |  |  |  |  |  |  |  |  |  |  |  |  |  |  |  |  |  |  |  |  |  |  |  |  |  |  |  |  |  |  |  |  |  |  |  |  |  |  |  |  |  |  |  |  |  |  |  |  |  |  |  |  |  |  |  |  |  |  |  |  |  |  |  |  |  |  |  |  |  |  |  |  |  |  |  |  |  |  |  |  |  |  |  |  |  |  |  |  |  |  |  |  |  |  |  |  |  |  |  |  |  |  |  |  |  |  |  |  |  |  |  |  |  |  |  |  |  |  |  |  |  |  |  |  |  |  |  |  |  |  |  |  |  |  |  |  |  |  |  |  |  |  |  |  |  |  |  |  |  |  |  |  |  |  |  |  |  |  |  |  |  |  |  |  |  |  |  |  |  |  |  |  |  |  |  |  |  |  |  |  |  |  |  |  |  |  |  |  |  |  |  |  |  |  |  |  |  |  |  |  |  |  |  |  |  |  |  |  |  |  |  |  |  |  |  |  |  |  |  |  |  |  |  |  |  |  |  |  |  |  |  |  |  |  |  |  |  |  |  |  |  |  |  |  |  |  |  |  |  |  |  |  |  |  |  |  |  |  |  |  |  |  |  |  |  |  |  |  |  |  |  |  |  |  |  |  |  |  |  |  |  |  |  |  |  |  |  |  |  |  |  |  |  |  |  |  |  |  |  |  |  |  |  |  |  |  |  |  |  |  |  |  |  |  |  |  |  |  |  |  |  |  |  |  |  |  |  |  |  |  |  |  |  |  |  |  |  |  |  |  |  |  |  |  |  |  |  |  |  |  |  |  |  |  |  |  |  |  |  |  |  |  |  |  |  |  |  |  |  |  |  |  |  |  |  |  |  |  |  |  |  |  |  |  |  |  |  |  |  |  |  |  |  |  |  |  |  |  |  |  |  |  |  |  |  |  |  |  |  |  |  |  |  |  |  |  |  |  |  |  |  |  |  |  |  |  |  |  |  |  |  |  |  |  |  |  |  |  |  |  |  |  |  |  |  |  |  |  |  |  |  |  |  |  |  |  |  |  |  |  |  |  |  |  |  |  |  |  |  |  |  |  |  |  |  |  |  |  |  |  |  |  |  |  |  |  |  |  |  |  |  |  |  |  |  |  |  |  |  |  |  |  |  |  |  |  |  |  |  |  |  |  |  |  |  |  |  |  |  |  |  |  |  |  |  |  |  |  |  |  |  |  |  |  |  |  |  |  |  |  |  |  |  |  |  |  |  |  |  |  |  |  |  |  |  |  |  |  |  |  |  |  |  |  |  |  |  |  |  |  |  |  |  |  |  |  |  |  |  |  |  |  |  |  |  |  |  |  |  |  |  |  |  |  |  |  |  |  |  |  |  |  |  |  |  |  |  |  |  |  |  |  |  |  |  |  |  |  |  |  |  |  |  |  |  |  |  |  |  |  |  |  |  |  |  |  |  |  |  |  |  |  |  |  |  |  |  |  |  |  |  |  |  |  |  |  |  |  |  |  |  |  |  |  |  |  |  |  |  |  |  |  |  |  |  |  |  |  |  |  |  |  |  |  |  |  |  |  |  |  |  |  |  |  |  |  |  |  |  |  |  |  |  |  |  |  |  |  |  |  |  |  |  |  |  |  |  |  |  |  |  |  |  |  |  |  |  |  |  |  |  |  |  |  |  |  |  |  |  |  |  |  |  |  |  |  |  |  |  |  |  |  |  |  |  |  |  |  |  |  |  |  |  |  |  |  |  |  |  |  |  |  |  |  |  |  |  |  |  |  |  |  |  |  |  |  |  |  |  |  |  |  |  |  |  |  |  |  |  |  |  |  |  |  |  |  |  |  |  |  |  |  |  |  |  |  |  |  |  |  |  |  |  |  |  |  |  |  |  |  |  |  |  |  |  |  |  |  |  |  |  |  |  |  |  |  |  |  |  |  |  |  |  |  |  |  |  |  |  |  |  |  |  |  |  |  |  |  |  |  |  |  |  |  |  |  |  |  |  |  |  |  |  |  |  |  |  |  |  |  |  |  |  |  |  |  |  |  |  |  |  |  |  |  |  |  |  |  |  |  |  |  |  |  |  |  |  |  |  |  |  |  |  |  |  |  |  |  |  |  |  |  |  |  |  |  |  |  |  |  |  |  |  |  |  |  |  |  |
| --- | --- | --- | --- | --- | --- | --- | --- | --- | --- | --- | --- | --- | --- | --- | --- | --- | --- | --- | --- | --- | --- | --- | --- | --- | --- | --- | --- | --- | --- | --- | --- | --- | --- | --- | --- | --- | --- | --- | --- | --- | --- | --- | --- | --- | --- | --- | --- | --- | --- | --- | --- | --- | --- | --- | --- | --- | --- | --- | --- | --- | --- | --- | --- | --- | --- | --- | --- | --- | --- | --- | --- | --- | --- | --- | --- | --- | --- | --- | --- | --- | --- | --- | --- | --- | --- | --- | --- | --- | --- | --- | --- | --- | --- | --- | --- | --- | --- | --- | --- | --- | --- | --- | --- | --- | --- | --- | --- | --- | --- | --- | --- | --- | --- | --- | --- | --- | --- | --- | --- | --- | --- | --- | --- | --- | --- | --- | --- | --- | --- | --- | --- | --- | --- | --- | --- | --- | --- | --- | --- | --- | --- | --- | --- | --- | --- | --- | --- | --- | --- | --- | --- | --- | --- | --- | --- | --- | --- | --- | --- | --- | --- | --- | --- | --- | --- | --- | --- | --- | --- | --- | --- | --- | --- | --- | --- | --- | --- | --- | --- | --- | --- | --- | --- | --- | --- | --- | --- | --- | --- | --- | --- | --- | --- | --- | --- | --- | --- | --- | --- | --- | --- | --- | --- | --- | --- | --- | --- | --- | --- | --- | --- | --- | --- | --- | --- | --- | --- | --- | --- | --- | --- | --- | --- | --- | --- | --- | --- | --- | --- | --- | --- | --- | --- | --- | --- | --- | --- | --- | --- | --- | --- | --- | --- | --- | --- | --- | --- | --- | --- | --- | --- | --- | --- | --- | --- | --- | --- | --- | --- | --- | --- | --- | --- | --- | --- | --- | --- | --- | --- | --- | --- | --- | --- | --- | --- | --- | --- | --- | --- | --- | --- | --- | --- | --- | --- | --- | --- | --- | --- | --- | --- | --- | --- | --- | --- | --- | --- | --- | --- | --- | --- | --- | --- | --- | --- | --- | --- | --- | --- | --- | --- | --- | --- | --- | --- | --- | --- | --- | --- | --- | --- | --- | --- | --- | --- | --- | --- | --- | --- | --- | --- | --- | --- | --- | --- | --- | --- | --- | --- | --- | --- | --- | --- | --- | --- | --- | --- | --- | --- | --- | --- | --- | --- | --- | --- | --- | --- | --- | --- | --- | --- | --- | --- | --- | --- | --- | --- | --- | --- | --- | --- | --- | --- | --- | --- | --- | --- | --- | --- | --- | --- | --- | --- | --- | --- | --- | --- | --- | --- | --- | --- | --- | --- | --- | --- | --- | --- | --- | --- | --- | --- | --- | --- | --- | --- | --- | --- | --- | --- | --- | --- | --- | --- | --- | --- | --- | --- | --- | --- | --- | --- | --- | --- | --- | --- | --- | --- | --- | --- | --- | --- | --- | --- | --- | --- | --- | --- | --- | --- | --- | --- | --- | --- | --- | --- | --- | --- | --- | --- | --- | --- | --- | --- | --- | --- | --- | --- | --- | --- | --- | --- | --- | --- | --- | --- | --- | --- | --- | --- | --- | --- | --- | --- | --- | --- | --- | --- | --- | --- | --- | --- | --- | --- | --- | --- | --- | --- | --- | --- | --- | --- | --- | --- | --- | --- | --- | --- | --- | --- | --- | --- | --- | --- | --- | --- | --- | --- | --- | --- | --- | --- | --- | --- | --- | --- | --- | --- | --- | --- | --- | --- | --- | --- | --- | --- | --- | --- | --- | --- | --- | --- | --- | --- | --- | --- | --- | --- | --- | --- | --- | --- | --- | --- | --- | --- | --- | --- | --- | --- | --- | --- | --- | --- | --- | --- | --- | --- | --- | --- | --- | --- | --- | --- | --- | --- | --- | --- | --- | --- | --- | --- | --- | --- | --- | --- | --- | --- | --- | --- | --- | --- | --- | --- | --- | --- | --- | --- | --- | --- | --- | --- | --- | --- | --- | --- | --- | --- | --- | --- | --- | --- | --- | --- | --- | --- | --- | --- | --- | --- | --- | --- | --- | --- | --- | --- | --- | --- | --- | --- | --- | --- | --- | --- | --- | --- | --- | --- | --- | --- | --- | --- | --- | --- | --- | --- | --- | --- | --- | --- | --- | --- | --- | --- | --- | --- | --- | --- | --- | --- | --- | --- | --- | --- | --- | --- | --- | --- | --- | --- | --- | --- | --- | --- | --- | --- | --- | --- | --- | --- | --- | --- | --- | --- | --- | --- | --- | --- | --- | --- | --- | --- | --- | --- | --- | --- | --- | --- | --- | --- | --- | --- | --- | --- | --- | --- | --- | --- | --- | --- | --- | --- | --- | --- | --- | --- | --- | --- | --- | --- | --- | --- | --- | --- | --- | --- | --- | --- | --- | --- | --- | --- | --- | --- | --- | --- | --- | --- | --- | --- | --- | --- | --- | --- | --- | --- | --- | --- | --- | --- | --- | --- | --- | --- | --- | --- | --- | --- | --- | --- | --- | --- | --- | --- | --- | --- | --- | --- | --- | --- | --- | --- | --- | --- | --- | --- | --- | --- | --- | --- | --- | --- | --- | --- | --- | --- | --- | --- | --- | --- | --- | --- | --- | --- | --- | --- | --- | --- | --- | --- | --- | --- | --- | --- | --- | --- | --- | --- | --- | --- | --- | --- | --- | --- | --- | --- | --- | --- | --- | --- | --- | --- | --- | --- | --- | --- | --- | --- | --- | --- | --- | --- | --- | --- | --- | --- | --- | --- | --- | --- | --- | --- | --- | --- | --- | --- | --- | --- | --- | --- | --- | --- | --- | --- | --- | --- | --- | --- | --- | --- | --- | --- | --- | --- | --- | --- | --- | --- | --- | --- | --- | --- | --- | --- | --- | --- | --- | --- | --- | --- | --- | --- | --- | --- | --- | --- | --- | --- | --- | --- | --- | --- | --- | --- | --- | --- | --- | --- | --- | --- | --- | --- | --- | --- | --- | --- | --- | --- | --- | --- | --- | --- | --- | --- | --- | --- | --- | --- | --- | --- | --- | --- | --- | --- | --- | --- | --- | --- | --- | --- | --- | --- | --- | --- | --- | --- | --- | --- | --- | --- | --- | --- | --- | --- | --- | --- | --- | --- | --- | --- | --- | --- | --- | --- | --- | --- | --- | --- | --- | --- | --- | --- | --- | --- | --- | --- | --- | --- | --- | --- | --- | --- | --- | --- | --- | --- | --- | --- | --- | --- | --- | --- | --- | --- | --- | --- | --- | --- | --- | --- | --- | --- | --- | --- | --- | --- | --- | --- | --- | --- | --- | --- | --- | --- | --- | --- | --- | --- | --- | --- | --- | --- | --- | --- | --- | --- | --- | --- | --- | --- | --- | --- | --- | --- | --- | --- | --- | --- | --- | --- | --- | --- | --- | --- | --- | --- | --- | --- | --- | --- | --- | --- | --- | --- | --- | --- | --- | --- | --- | --- | --- | --- | --- | --- | --- | --- | --- | --- | --- | --- | --- | --- | --- | --- | --- | --- | --- | --- | --- | --- | --- | --- | --- | --- | --- | --- | --- | --- | --- | --- | --- | --- | --- | --- | --- | --- | --- | --- | --- | --- | --- | --- | --- | --- | --- | --- | --- | --- | --- | --- | --- | --- | --- | --- | --- | --- | --- | --- | --- | --- | --- | --- | --- | --- | --- | --- | --- | --- | --- | --- | --- | --- | --- | --- | --- | --- | --- | --- | --- | --- | --- | --- | --- | --- | --- | --- | --- | --- | --- | --- | --- | --- | --- | --- | --- | --- | --- | --- | --- | --- | --- | --- | --- | --- | --- | --- | --- | --- | --- | --- | --- | --- | --- | --- | --- | --- | --- | --- | --- | --- | --- | --- | --- | --- | --- | --- | --- | --- | --- | --- | --- | --- | --- | --- | --- | --- | --- | --- | --- | --- | --- | --- | --- | --- | --- | --- | --- | --- | --- | --- | --- | --- | --- | --- | --- | --- | --- | --- | --- | --- | --- | --- | --- | --- | --- | --- | --- | --- | --- | --- | --- | --- | --- | --- | --- | --- | --- | --- | --- | --- | --- | --- | --- | --- | --- | --- | --- | --- | --- | --- | --- | --- | --- | --- | --- | --- | --- | --- | --- | --- | --- | --- | --- | --- | --- | --- | --- | --- | --- | --- | --- | --- | --- | --- | --- | --- | --- | --- | --- | --- | --- | --- | --- | --- | --- | --- | --- | --- | --- | --- | --- | --- | --- | --- | --- | --- | --- | --- | --- | --- | --- | --- | --- | --- | --- | --- | --- | --- | --- | --- | --- | --- | --- | --- | --- | --- | --- | --- | --- | --- | --- | --- | --- | --- | --- | --- | --- | --- | --- | --- | --- | --- | --- | --- | --- | --- | --- | --- | --- | --- | --- | --- | --- | --- | --- | --- | --- | --- | --- | --- | --- | --- | --- | --- | --- | --- | --- | --- | --- | --- | --- | --- | --- | --- | --- | --- | --- | --- | --- | --- | --- | --- | --- | --- | --- | --- | --- | --- | --- | --- | --- | --- | --- | --- | --- | --- | --- | --- | --- | --- | --- | --- | --- | --- | --- | --- | --- | --- | --- | --- | --- | --- | --- | --- | --- | --- | --- | --- | --- | --- | --- | --- | --- | --- | --- | --- | --- | --- | --- | --- | --- | --- | --- | --- | --- | --- | --- | --- | --- | --- | --- | --- | --- | --- | --- | --- | --- | --- | --- | --- | --- | --- | --- | --- | --- | --- | --- | --- | --- | --- | --- | --- | --- | --- | --- | --- | --- | --- | --- | --- | --- | --- | --- | --- | --- | --- | --- | --- | --- | --- | --- | --- | --- | --- | --- | --- | --- | --- | --- | --- | --- | --- | --- | --- | --- | --- | --- | --- | --- | --- | --- | --- | --- | --- | --- | --- | --- | --- | --- | --- | --- | --- | --- | --- | --- | --- | --- | --- | --- | --- | --- | --- | --- | --- | --- | --- | --- | --- | --- | --- | --- | --- | --- | --- | --- | --- | --- | --- | --- | --- | --- | --- | --- | --- | --- | --- | --- | --- | --- | --- | --- | --- | --- | --- | --- | --- | --- | --- | --- | --- | --- | --- | --- | --- | --- | --- | --- | --- | --- | --- | --- | --- | --- | --- | --- | --- | --- | --- | --- | --- | --- | --- | --- | --- | --- | --- | --- | --- | --- | --- | --- | --- | --- | --- | --- | --- | --- | --- | --- | --- | --- | --- | --- | --- | --- | --- | --- | --- | --- | --- | --- | --- | --- | --- | --- | --- | --- | --- | --- | --- | --- | --- | --- | --- | --- | --- | --- | --- | --- | --- | --- | --- | --- | --- | --- | --- | --- | --- | --- | --- | --- | --- | --- | --- | --- | --- | --- | --- | --- | --- | --- | --- | --- | --- | --- | --- | --- | --- | --- | --- | --- | --- | --- | --- | --- | --- | --- | --- | --- | --- | --- | --- | --- | --- | --- | --- | --- | --- | --- | --- | --- | --- | --- | --- | --- | --- | --- | --- | --- | --- | --- | --- | --- | --- | --- | --- | --- | --- | --- | --- | --- | --- | --- | --- | --- | --- | --- | --- | --- | --- | --- | --- | --- | --- | --- | --- | --- | --- | --- | --- | --- | --- | --- | --- | --- | --- | --- | --- | --- | --- | --- | --- | --- | --- | --- | --- | --- | --- | --- | --- | --- | --- | --- | --- | --- | --- | --- | --- | --- | --- | --- | --- | --- | --- | --- | --- | --- | --- | --- | --- | --- | --- | --- | --- | --- | --- | --- | --- | --- | --- | --- | --- | --- | --- | --- | --- | --- | --- | --- | --- | --- | --- | --- | --- | --- | --- | --- | --- | --- | --- | --- | --- | --- | --- | --- | --- | --- | --- | --- | --- | --- | --- | --- | --- | --- | --- | --- | --- | --- | --- | --- | --- | --- | --- | --- | --- | --- | --- | --- | --- | --- | --- | --- | --- | --- | --- | --- | --- | --- | --- | --- | --- | --- | --- | --- | --- | --- | --- | --- | --- | --- | --- | --- | --- | --- | --- | --- | --- | --- | --- | --- | --- | --- | --- | --- | --- | --- | --- | --- | --- | --- | --- | --- | --- | --- | --- | --- | --- | --- | --- | --- | --- | --- | --- | --- | --- | --- | --- | --- | --- | --- | --- | --- | --- | --- | --- | --- | --- | --- | --- | --- | --- | --- | --- | --- | --- | --- | --- | --- | --- | --- | --- | --- | --- | --- | --- | --- | --- | --- | --- | --- | --- | --- | --- | --- | --- | --- | --- | --- | --- | --- | --- | --- | --- | --- | --- | --- | --- | --- | --- | --- | --- | --- | --- | --- | --- | --- | --- | --- | --- | --- | --- | --- | --- | --- | --- | --- | --- | --- | --- | --- | --- | --- | --- | --- | --- | --- | --- | --- | --- | --- | --- | --- | --- | --- | --- | --- | --- | --- | --- | --- | --- | --- | --- | --- | --- | --- | --- | --- | --- | --- | --- | --- | --- | --- | --- | --- | --- | --- | --- | --- | --- | --- | --- | --- | --- | --- | --- | --- | --- | --- | --- | --- | --- | --- | --- | --- | --- | --- | --- | --- | --- | --- | --- | --- | --- | --- | --- | --- | --- | --- | --- | --- | --- | --- | --- | --- | --- | --- | --- | --- | --- | --- | --- | --- | --- | --- | --- | --- | --- | --- | --- | --- | --- | --- | --- | --- | --- | --- | --- | --- | --- | --- | --- | --- | --- | --- | --- | --- | --- | --- | --- | --- | --- | --- | --- | --- | --- | --- | --- | --- | --- | --- | --- | --- | --- | --- | --- | --- | --- | --- | --- | --- | --- | --- | --- | --- | --- | --- | --- | --- | --- | --- | --- | --- | --- | --- | --- | --- | --- | --- | --- | --- | --- | --- | --- | --- | --- | --- | --- | --- | --- | --- | --- | --- | --- | --- | --- | --- | --- | --- | --- | --- | --- | --- | --- | --- | --- | --- | --- | --- | --- | --- | --- | --- | --- | --- | --- | --- | --- | --- | --- | --- | --- | --- | --- | --- | --- | --- | --- | --- | --- | --- | --- | --- | --- | --- | --- | --- | --- | --- | --- | --- | --- | --- | --- | --- | --- | --- | --- | --- | --- | --- | --- | --- | --- | --- | --- | --- | --- | --- | --- | --- | --- | --- | --- | --- | --- | --- | --- | --- | --- | --- | --- | --- | --- | --- | --- | --- | --- | --- | --- | --- | --- | --- | --- | --- | --- | --- | --- | --- | --- | --- | --- | --- | --- | --- | --- | --- | --- | --- | --- | --- | --- | --- | --- | --- | --- | --- | --- | --- | --- | --- | --- | --- | --- | --- | --- | --- | --- | --- | --- | --- | --- | --- | --- | --- | --- | --- | --- | --- | --- | --- | --- | --- | --- | --- | --- | --- | --- | --- | --- | --- | --- | --- | --- | --- | --- | --- | --- | --- | --- | --- | --- | --- | --- | --- | --- | --- | --- | --- | --- | --- | --- | --- | --- | --- | --- | --- | --- | --- | --- | --- | --- | --- | --- | --- | --- | --- | --- | --- | --- | --- | --- | --- | --- | --- | --- | --- | --- | --- | --- | --- | --- | --- | --- | --- | --- | --- | --- | --- | --- | --- | --- | --- | --- | --- | --- | --- | --- | --- | --- | --- | --- | --- | --- | --- | --- | --- | --- | --- | --- | --- | --- | --- | --- | --- | --- | --- | --- | --- | --- | --- | --- | --- | --- | --- | --- | --- | --- | --- | --- | --- | --- | --- | --- | --- | --- | --- | --- | --- | --- | --- | --- | --- | --- | --- | --- | --- | --- | --- | --- | --- | --- | --- | --- | --- | --- | --- | --- | --- | --- | --- | --- | --- | --- | --- | --- | --- | --- | --- | --- | --- | --- | --- | --- | --- | --- | --- | --- | --- | --- | --- | --- | --- | --- | --- | --- | --- | --- | --- | --- | --- | --- | --- | --- | --- | --- | --- | --- | --- | --- | --- | --- | --- | --- | --- | --- | --- | --- | --- | --- | --- | --- | --- | --- | --- | --- | --- | --- | --- | --- | --- | --- | --- | --- | --- | --- | --- | --- | --- | --- | --- | --- | --- | --- | --- | --- | --- | --- | --- | --- | --- | --- | --- | --- | --- | --- | --- | --- | --- | --- | --- | --- | --- | --- | --- | --- | --- | --- | --- | --- | --- | --- | --- | --- | --- | --- | --- | --- | --- | --- | --- | --- | --- | --- | --- | --- | --- | --- | --- | --- | --- | --- | --- | --- | --- | --- | --- | --- | --- | --- | --- | --- | --- | --- | --- | --- | --- | --- | --- | --- | --- | --- | --- | --- | --- | --- | --- | --- | --- | --- | --- | --- | --- | --- | --- | --- | --- | --- | --- | --- | --- | --- | --- | --- | --- | --- | --- | --- | --- | --- | --- | --- | --- | --- | --- | --- | --- | --- | --- | --- | --- | --- | --- | --- | --- | --- | --- | --- | --- | --- | --- | --- | --- | --- | --- | --- | --- | --- | --- | --- | --- | --- | --- | --- | --- | --- | --- | --- | --- | --- | --- | --- | --- | --- | --- | --- | --- | --- | --- | --- | --- | --- | --- | --- | --- | --- | --- | --- | --- | --- | --- | --- | --- | --- | --- | --- | --- | --- | --- | --- | --- | --- | --- | --- | --- | --- | --- | --- | --- | --- | --- | --- | --- | --- | --- | --- | --- | --- | --- | --- | --- | --- | --- | --- | --- | --- | --- | --- | --- | --- | --- | --- | --- | --- | --- | --- | --- | --- | --- | --- | --- | --- | --- | --- | --- | --- | --- | --- | --- | --- | --- | --- | --- | --- | --- | --- | --- | --- | --- | --- | --- | --- | --- | --- | --- | --- | --- | --- | --- | --- | --- | --- | --- | --- | --- | --- | --- | --- | --- | --- | --- | --- | --- | --- | --- | --- | --- | --- | --- | --- | --- | --- | --- | --- | --- | --- | --- | --- | --- | --- | --- | --- | --- | --- | --- | --- | --- | --- | --- | --- | --- | --- | --- | --- | --- | --- | --- | --- | --- | --- | --- | --- | --- | --- | --- | --- | --- | --- | --- | --- | --- | --- | --- | --- | --- | --- | --- | --- | --- | --- | --- | --- | --- | --- | --- | --- | --- | --- | --- | --- | --- | --- | --- | --- | --- | --- | --- | --- | --- | --- | --- | --- | --- | --- | --- | --- | --- | --- | --- | --- | --- | --- | --- | --- | --- | --- | --- | --- | --- | --- | --- | --- | --- | --- | --- | --- | --- | --- | --- | --- | --- | --- | --- | --- | --- | --- | --- | --- | --- | --- | --- | --- | --- | --- | --- | --- | --- | --- | --- | --- | --- | --- | --- | --- | --- | --- | --- | --- | --- | --- | --- | --- | --- | --- | --- | --- | --- | --- | --- | --- | --- | --- | --- | --- | --- | --- | --- | --- | --- | --- | --- | --- | --- | --- | --- | --- | --- | --- | --- | --- | --- | --- | --- | --- | --- | --- | --- | --- | --- | --- | --- | --- | --- | --- | --- | --- | --- | --- | --- | --- | --- | --- | --- | --- | --- | --- | --- | --- | --- | --- | --- | --- | --- | --- | --- | --- | --- | --- | --- | --- | --- | --- | --- | --- | --- | --- | --- | --- | --- | --- | --- | --- | --- | --- | --- | --- | --- | --- | --- | --- | --- | --- | --- | --- | --- | --- | --- | --- | --- | --- | --- | --- | --- | --- | --- | --- | --- | --- | --- | --- | --- | --- | --- | --- | --- | --- | --- | --- | --- | --- | --- | --- | --- | --- | --- | --- | --- | --- | --- | --- | --- | --- | --- | --- | --- | --- | --- | --- | --- | --- | --- | --- | --- | --- | --- | --- | --- | --- | --- | --- | --- | --- | --- | --- | --- | --- | --- | --- | --- | --- | --- | --- | --- | --- | --- | --- | --- | --- | --- | --- | --- | --- | --- | --- | --- | --- | --- | --- | --- | --- | --- | --- | --- | --- | --- | --- | --- | --- | --- | --- | --- | --- | --- | --- | --- | --- | --- | --- | --- | --- | --- | --- | --- | --- | --- | --- | --- | --- | --- | --- | --- | --- | --- | --- |
| |  |  |  |  |  | | --- | --- | --- | --- | --- | | **Best GOs** (Max: 10000) | **Genes** | **Count** 576 | **Total** 10875 | **P-Value** | | GO:0009607 GO:0006952 GO:0006955 GO:0051707 GO:0009613 GO:0050896 GO:0006954 GO:0009611 GO:0006950 GO:0009605 GO:0006959 GO:0016064 | IL7R MSRA IFI35 NFKBIA PINK1 CYSLTR2 ZAP70 FOS PSTPIP1 CRIP1 CD28 NBN NINJ1 MAP4K4 GNAQ S100A12 TREM1 DEFA1 IL4R STAT2 C3AR1 IFNAR2 PTAFR PTX3 HLA-DPB1 ANXA1 PPP1R15A CARD4 MYH9 EP300 KIR2DL2 NCF2 LILRA2 BCL6 TLR2 CD74 CD6 PFC LCK TYROBP HLA-DRA APS TNFSF8 DERL2 KLRK1 IKBKG MS4A1 HCP5 MYD88 FCGRT SGK3 SEMA4D RNASE2 IRF1 CSF1R CMTM6 CD19 CD244 NR4A2 F13A1 MGLL HLA-B HPS1 AOAH PPBP TRIM22 TAP1 LTB RNASE6 CSF3R CYBB TNFSF4 ELA2 HLA-A BNIP3L AZU1 ISG20 APOL3 MAP3K5 F8 CD3E IL6R NOTCH1 NFIL3 CIRBP CD53 STAT3 ABCG1 LILRB1 TLR1 CTSG ITIH4 HIF1A CARD15 TBXAS1 CLC PDE6G CFD TAPBP TNFRSF14 PTGS2 B2M RNASE3 TNFRSF7 PLP2 BATF CD2 LY75 PTGER4 ATXN7 DNAJC3 BST1 CCR7 FOSB MVP NDRG1 GBP2 MNDA TP53AP1 SPON2 IL1B KLRB1 LTB4R CD97 NINJ2 TXNDC4 PSMB9 CXCR3 S100A9 CCL4 COL4A3BP KLF6 FPR1 FCER1G PTPRC ARHGDIB | 108 102 95 66 64 136 27 41 82 46 21 16 | 787 752 682 471 460 1622 182 336 881 425 146 105 | 1.22e-23 9.35e-22 1.74e-21 2.98e-14 1.37e-13 2.94e-06 1.75e-05 1.75e-05 3.74e-05 0.000257 0.00111 0.0058 | | GO:0007243 GO:0007242 GO:0007249 GO:0043123 GO:0043122 GO:0007165 | IL7R HCK DGKA GPR109B NFKBIA PINK1 CYSLTR2 GABBR1 ZAP70 IQGAP1 PSTPIP1 RGS2 CD28 RPS6KA4 MAP4K4 RHOB PPM1A GNAQ ULK1 NISCH TREM1 SH2B FES DOCK5 IL4R CHRNE MARK2 STAT2 C3AR1 ITPKB IFNAR2 PTAFR SNX19 ANXA1 CARD4 EP300 AXIN1 ITGAE ADRB2 LILRA2 PLEKHM1 TLR2 CD74 TGFBR2 LCK TYROBP EDG4 STAT6 APS RAF1 TNFSF8 DERL2 ERG IKBKG GPSM3 PECAM1 MS4A1 MYD88 S100A6 AKAP11 SGK3 DGKZ VCPIP1 NRGN PILRB TRAF6 GMIP MAP3K3 CSF1R TLE1 CD19 CD244 BIRC2 PTGIR NR4A2 APBB1IP FRAT1 CASP8 LTB GPR162 CD3Z APH1B RAB8B CSF3R TRIM38 PTGER2 TNFSF4 MAP3K11 DGKG DAPP1 AZU1 ITSN2 CALCOCO1 PILRA APOL3 FLNA MAL MAP3K5 CD3E IL2RB JAK2 RGL2 RABL4 IL6R RAB4B NOTCH1 GSK3B PDPK1 CD53 CASP1 STAT3 NCOA4 CENTD2 TLR1 APBB3 HIF1A TRAF1 CARD15 TMEM9B RAP2B RIN3 EMR2 TNFRSF14 CAP1 VAV1 TNFRSF7 PLP2 IRAK3 CD2 CSF2RB SPEN P2RX1 MICAL1 MKNK1 PTGER4 CCR7 TRADD CHN2 ABR CD38 IL1B ARHGAP1 KLRB1 LTB4R DOK1 CD97 CXCR3 CHRNA7 CCL4 FPR1 FCER1G PTPRC IL17R ARHGEF18 ARHGDIB | 34 80 18 14 14 155 | 233 884 94 67 71 2162 | 5.35e-07 0.000227 0.000997 0.00431 0.00694 0.00694 | | GO:0009967 | MAP3K3 LCK PPM1A TNFRSF7 BIRC2 CARD15 APOL3 MYD88 FLNA TMEM9B TRADD CASP8 CARD4 PTPRC TRIM38 CASP1 TRAF6 | 17 | 79 | 0.000373 | | GO:0006915 GO:0012501 GO:0008219 GO:0042981 GO:0016265 GO:0043067 | LCK ELMO2 TNFRSF7 RAF1 CD2 TNFSF8 BIRC1 NFKBIA P2RX1 IKBKG BNIP3L AZU1 NGFRAP1 CASP4 MAL MAP3K5 CD3E IL2RB TRADD BTG1 BCL2A1 CD38 SEMA4D CD28 IL1B CASP1 EAF2 TRAF6 RHOB MCL1 CIDEC BIRC2 TRAF1 CARD15 SON ANXA1 PPP1R15A CASP8 CARD4 AXIN1 EP300 PTPRC ING4 TNFRSF14 GZMM TLR2 CD74 | 46 46 47 33 47 33 | 465 466 490 307 493 308 | 0.0048 0.00488 0.00825 0.00886 0.00886 0.00924 | | GO:0050874 | CYBB TNFSF4 IL7R ELA2 HLA-A IFI35 GCH1 AZU1 CYSLTR2 FOS ZAP70 APOL3 MAL CD3E F8 IL6R NOTCH1 CRIP1 NFIL3 CD28 CD53 STAT3 NBN LILRB1 GNAQ TLR1 CTSG S100A12 TREM1 ITIH4 IL4R CHRNE C3AR1 HLA-DPB1 PTX3 PTAFR CLC TBXAS1 PDE6G ANXA1 CFD MYH9 CARD4 TAPBP LNPEP TNFRSF14 PTGS2 NCF2 KIR2DL2 LILRA2 B2M BCL6 TLR2 CD74 LCK PFC CD6 TNFRSF7 TYROBP HLA-DRA APS BATF CD2 TNFSF8 LY75 CSF2RB KLRK1 P2RX1 IKBKG MS4A1 MYD88 PTGER4 ATXN7 BST1 CCR7 FCGRT SLC22A4 GBP2 MNDA SPON2 SEMA4D IL1B GABARAP IRF1 KLRB1 LTB4R CD97 CSF1R PPP1R12A CD19 CD244 PSMB9 CXCR3 CHRNA7 F13A1 NR4A2 MGLL HLA-B S100A9 PABPN1 HPS1 CCL4 COL4A3BP AOAH PPBP KLF6 FPR1 TRIM22 FCER1G TAP1 LTB PTPRC TAZ ARHGDIB | 114 | 1503 | 0.00886 | |  | |  |  |  | | --- | --- | --- | | **Gene** | **Search Term** | **GOs** | | 200696\_S\_AT | 200696\_s\_at |  | | 200701\_AT | 200701\_at |  | | 200714\_X\_AT | 200714\_x\_at |  | | 200743\_S\_AT | 200743\_s\_at |  | | 200747\_S\_AT | 200747\_s\_at |  | | 200905\_X\_AT | 200905\_x\_at |  | | 200911\_S\_AT | 200911\_s\_at |  | | 200999\_S\_AT | 200999\_s\_at |  | | 201009\_S\_AT | 201009\_s\_at |  | | 201040\_AT | 201040\_at |  | | 201041\_S\_AT | 201041\_s\_at |  | | 201103\_X\_AT | 201103\_x\_at |  | | 201104\_X\_AT | 201104\_x\_at |  | | 201152\_S\_AT | 201152\_s\_at |  | | 201163\_S\_AT | 201163\_s\_at |  | | 201192\_S\_AT | 201192\_s\_at |  | | 201220\_X\_AT | 201220\_x\_at |  | | 201236\_S\_AT | 201236\_s\_at |  | | 201285\_AT | 201285\_at |  | | 201294\_S\_AT | 201294\_s\_at |  | | 201368\_AT | 201368\_at |  | | 201432\_AT | 201432\_at |  | | 201580\_S\_AT | 201580\_s\_at |  | | 201613\_S\_AT | 201613\_s\_at |  | | 201640\_X\_AT | 201640\_x\_at |  | | 201721\_S\_AT | 201721\_s\_at |  | | 201813\_S\_AT | 201813\_s\_at |  | | 201844\_S\_AT | 201844\_s\_at |  | | 201858\_S\_AT | 201858\_s\_at |  | | 201861\_S\_AT | 201861\_s\_at |  | | 201965\_S\_AT | 201965\_s\_at |  | | 202008\_S\_AT | 202008\_s\_at |  | | 202025\_X\_AT | 202025\_x\_at |  | | 202039\_AT | 202039\_at |  | | 202040\_S\_AT | 202040\_s\_at |  | | 202173\_S\_AT | 202173\_s\_at |  | | 202191\_S\_AT | 202191\_s\_at |  | | 202197\_AT | 202197\_at |  | | 202207\_AT | 202207\_at |  | | 202377\_AT | 202377\_at |  | | 202378\_S\_AT | 202378\_s\_at |  | | 202386\_S\_AT | 202386\_s\_at |  | | 202459\_S\_AT | 202459\_s\_at |  | | 202510\_S\_AT | 202510\_s\_at |  | | 202524\_S\_AT | 202524\_s\_at |  | | 202530\_AT | 202530\_at |  | | 202574\_S\_AT | 202574\_s\_at |  | | 202590\_S\_AT | 202590\_s\_at |  | | 202626\_S\_AT | 202626\_s\_at |  | | 202644\_S\_AT | 202644\_s\_at |  | | 202682\_S\_AT | 202682\_s\_at |  | | 202688\_AT | 202688\_at |  | | 202808\_AT | 202808\_at |  | | 202815\_S\_AT | 202815\_s\_at |  | | 202822\_AT | 202822\_at |  | | 202860\_AT | 202860\_at |  | | 202864\_S\_AT | 202864\_s\_at |  | | 202878\_S\_AT | 202878\_s\_at |  | | 202880\_S\_AT | 202880\_s\_at |  | | 202917\_S\_AT | 202917\_s\_at |  | | 202951\_AT | 202951\_at |  | | 203054\_S\_AT | 203054\_s\_at |  | | 203110\_AT | 203110\_at |  | | 203281\_S\_AT | 203281\_s\_at |  | | 203317\_AT | 203317\_at |  | | 203318\_S\_AT | 203318\_s\_at |  | | 203380\_X\_AT | 203380\_x\_at |  | | 203384\_S\_AT | 203384\_s\_at |  | | 203479\_S\_AT | 203479\_s\_at |  | | 203509\_AT | 203509\_at |  | | 203555\_AT | 203555\_at |  | | 203590\_AT | 203590\_at |  | | 203624\_AT | 203624\_at |  | | 203656\_AT | 203656\_at |  | | 203752\_S\_AT | 203752\_s\_at |  | | 203760\_S\_AT | 203760\_s\_at |  | | 203823\_AT | 203823\_at |  | | 203839\_S\_AT | 203839\_s\_at |  | | 203879\_AT | 203879\_at |  | | 203882\_AT | 203882\_at |  | | 203907\_S\_AT | 203907\_s\_at |  | | 203973\_S\_AT | 203973\_s\_at |  | | 203990\_S\_AT | 203990\_s\_at |  | | 204006\_S\_AT | 204006\_s\_at |  | | 204018\_X\_AT | 204018\_x\_at |  | | 204019\_S\_AT | 204019\_s\_at |  | | 204046\_AT | 204046\_at |  | | 204054\_AT | 204054\_at |  | | 204131\_S\_AT | 204131\_s\_at |  | | 204167\_AT | 204167\_at |  | | 204221\_X\_AT | 204221\_x\_at |  | | 204270\_AT | 204270\_at |  | | 204308\_S\_AT | 204308\_s\_at |  | | 204396\_S\_AT | 204396\_s\_at |  | | 204446\_S\_AT | 204446\_s\_at |  | | 204466\_S\_AT | 204466\_s\_at |  | | 204487\_S\_AT | 204487\_s\_at |  | | 204494\_S\_AT | 204494\_s\_at |  | | 204500\_S\_AT | 204500\_s\_at |  | | 204585\_S\_AT | 204585\_s\_at |  | | 204592\_AT | 204592\_at |  | | 204781\_S\_AT | 204781\_s\_at |  | | 204800\_S\_AT | 204800\_s\_at |  | | 204806\_X\_AT | 204806\_x\_at |  | | 204882\_AT | 204882\_at |  | | 204908\_S\_AT | 204908\_s\_at |  | | 204961\_S\_AT | 204961\_s\_at |  | | 205022\_S\_AT | 205022\_s\_at |  | | 205096\_AT | 205096\_at |  | | 205173\_X\_AT | 205173\_x\_at |  | | 205180\_S\_AT | 205180\_s\_at |  | | 205214\_AT | 205214\_at |  | | 205255\_X\_AT | 205255\_x\_at |  | | 205307\_S\_AT | 205307\_s\_at |  | | 205323\_S\_AT | 205323\_s\_at |  | | 205376\_AT | 205376\_at |  | | 205383\_S\_AT | 205383\_s\_at |  | | 205434\_S\_AT | 205434\_s\_at |  | | 205640\_AT | 205640\_at |  | | 205731\_S\_AT | 205731\_s\_at |  | | 205760\_S\_AT | 205760\_s\_at |  | | 205776\_AT | 205776\_at |  | | 205786\_S\_AT | 205786\_s\_at |  | | 205843\_X\_AT | 205843\_x\_at |  | | 205998\_X\_AT | 205998\_x\_at |  | | 206036\_S\_AT | 206036\_s\_at |  | | 206060\_S\_AT | 206060\_s\_at |  | | 206342\_X\_AT | 206342\_x\_at |  | | 206359\_AT | 206359\_at |  | | 206488\_S\_AT | 206488\_s\_at |  | | 206564\_AT | 206564\_at |  | | 206643\_AT | 206643\_at |  | | 206662\_AT | 206662\_at |  | | 206707\_X\_AT | 206707\_x\_at |  | | 206781\_AT | 206781\_at |  | | 206792\_X\_AT | 206792\_x\_at |  | | 207168\_S\_AT | 207168\_s\_at |  | | 207187\_AT | 207187\_at |  | | 207287\_AT | 207287\_at |  | | 207458\_AT | 207458\_at |  | | 207467\_X\_AT | 207467\_x\_at |  | | 207492\_AT | 207492\_at |  | | 207540\_S\_AT | 207540\_s\_at |  | | 207571\_X\_AT | 207571\_x\_at |  | | 207574\_S\_AT | 207574\_s\_at |  | | 207616\_S\_AT | 207616\_s\_at |  | | 207667\_S\_AT | 207667\_s\_at |  | | 207677\_S\_AT | 207677\_s\_at |  | | 207700\_S\_AT | 207700\_s\_at |  | | 207711\_AT | 207711\_at |  | | 207764\_S\_AT | 207764\_s\_at |  | | 207966\_S\_AT | 207966\_s\_at |  | | 207979\_S\_AT | 207979\_s\_at |  | | 208003\_S\_AT | 208003\_s\_at |  | | 208093\_S\_AT | 208093\_s\_at |  | | 208184\_S\_AT | 208184\_s\_at |  | | 208269\_S\_AT | 208269\_s\_at |  | | 208306\_X\_AT | 208306\_x\_at |  | | 208322\_S\_AT | 208322\_s\_at |  | | 208364\_AT | 208364\_at |  | | 208426\_X\_AT | 208426\_x\_at |  | | 208610\_S\_AT | 208610\_s\_at |  | | 208707\_AT | 208707\_at |  | | 208732\_AT | 208732\_at |  | | 208965\_S\_AT | 208965\_s\_at |  | | 208987\_S\_AT | 208987\_s\_at |  | | 209006\_S\_AT | 209006\_s\_at |  | | 209088\_S\_AT | 209088\_s\_at |  | | 209093\_S\_AT | 209093\_s\_at |  | | 209144\_S\_AT | 209144\_s\_at |  | | 209201\_X\_AT | 209201\_x\_at |  | | 209312\_X\_AT | 209312\_x\_at |  | | 209341\_S\_AT | 209341\_s\_at |  | | 209473\_AT | 209473\_at |  | | 209481\_AT | 209481\_at |  | | 209563\_X\_AT | 209563\_x\_at |  | | 209579\_S\_AT | 209579\_s\_at |  | | 209600\_S\_AT | 209600\_s\_at |  | | 209604\_S\_AT | 209604\_s\_at |  | | 209703\_X\_AT | 209703\_x\_at |  | | 209760\_AT | 209760\_at |  | | 209827\_S\_AT | 209827\_s\_at |  | | 209858\_X\_AT | 209858\_x\_at |  | | 209889\_AT | 209889\_at |  | | 209893\_S\_AT | 209893\_s\_at |  | | 210069\_AT | 210069\_at |  | | 210113\_S\_AT | 210113\_s\_at |  | | 210191\_S\_AT | 210191\_s\_at |  | | 210210\_AT | 210210\_at |  | | 210240\_S\_AT | 210240\_s\_at |  | | 210249\_S\_AT | 210249\_s\_at |  | | 210314\_X\_AT | 210314\_x\_at |  | | 210346\_S\_AT | 210346\_s\_at |  | | 210580\_X\_AT | 210580\_x\_at |  | | 210686\_X\_AT | 210686\_x\_at |  | | 210778\_S\_AT | 210778\_s\_at |  | | 210845\_S\_AT | 210845\_s\_at |  | | 210943\_S\_AT | 210943\_s\_at |  | | 211022\_S\_AT | 211022\_s\_at |  | | 211085\_S\_AT | 211085\_s\_at |  | | 211284\_S\_AT | 211284\_s\_at |  | | 211316\_X\_AT | 211316\_x\_at |  | | 211383\_S\_AT | 211383\_s\_at |  | | 211429\_S\_AT | 211429\_s\_at |  | | 211433\_X\_AT | 211433\_x\_at |  | | 211458\_S\_AT | 211458\_s\_at |  | | 211528\_X\_AT | 211528\_x\_at |  | | 211656\_X\_AT | 211656\_x\_at |  | | 211745\_X\_AT | 211745\_x\_at |  | | 211769\_X\_AT | 211769\_x\_at |  | | 211795\_S\_AT | 211795\_s\_at |  | | 211799\_X\_AT | 211799\_x\_at |  | | 211812\_S\_AT | 211812\_s\_at |  | | 211911\_X\_AT | 211911\_x\_at |  | | 211990\_AT | 211990\_at |  | | 211993\_AT | 211993\_at |  | | 211998\_AT | 211998\_at |  | | 212024\_X\_AT | 212024\_x\_at |  | | 212082\_S\_AT | 212082\_s\_at |  | | 212144\_AT | 212144\_at |  | | 212249\_AT | 212249\_at |  | | 212251\_AT | 212251\_at |  | | 212359\_S\_AT | 212359\_s\_at |  | | 212441\_AT | 212441\_at |  | | 212451\_AT | 212451\_at |  | | 212492\_S\_AT | 212492\_s\_at |  | | 212629\_S\_AT | 212629\_s\_at |  | | 212638\_S\_AT | 212638\_s\_at |  | | 212646\_AT | 212646\_at |  | | 212702\_S\_AT | 212702\_s\_at |  | | 212708\_AT | 212708\_at |  | | 212733\_AT | 212733\_at |  | | 212774\_AT | 212774\_at |  | | 212794\_S\_AT | 212794\_s\_at |  | | 212864\_AT | 212864\_at |  | | 212947\_AT | 212947\_at |  | | 212975\_AT | 212975\_at |  | | 212980\_AT | 212980\_at |  | | 213034\_AT | 213034\_at |  | | 213056\_AT | 213056\_at |  | | 213087\_S\_AT | 213087\_s\_at |  | | 213195\_AT | 213195\_at |  | | 213213\_AT | 213213\_at |  | | 213229\_AT | 213229\_at |  | | 213280\_AT | 213280\_at |  | | 213298\_AT | 213298\_at |  | | 213475\_S\_AT | 213475\_s\_at |  | | 213533\_AT | 213533\_at |  | | 213546\_AT | 213546\_at |  | | 213605\_S\_AT | 213605\_s\_at |  | | 213612\_X\_AT | 213612\_x\_at |  | | 213702\_X\_AT | 213702\_x\_at |  | | 213737\_X\_AT | 213737\_x\_at |  | | 213742\_AT | 213742\_at |  | | 213758\_AT | 213758\_at |  | | 213837\_AT | 213837\_at |  | | 213857\_S\_AT | 213857\_s\_at |  | | 213956\_AT | 213956\_at |  | | 213973\_AT | 213973\_at |  | | 213975\_S\_AT | 213975\_s\_at |  | | 214150\_X\_AT | 214150\_x\_at |  | | 214177\_S\_AT | 214177\_s\_at |  | | 214181\_X\_AT | 214181\_x\_at |  | | 214219\_X\_AT | 214219\_x\_at |  | | 214269\_AT | 214269\_at |  | | 214327\_X\_AT | 214327\_x\_at |  | | 214365\_AT | 214365\_at |  | | 214414\_X\_AT | 214414\_x\_at |  | | 214453\_S\_AT | 214453\_s\_at |  | | 214459\_X\_AT | 214459\_x\_at |  | | 214494\_S\_AT | 214494\_s\_at |  | | 214551\_S\_AT | 214551\_s\_at |  | | 214657\_S\_AT | 214657\_s\_at |  | | 214675\_AT | 214675\_at |  | | 214677\_X\_AT | 214677\_x\_at |  | | 214693\_X\_AT | 214693\_x\_at |  | | 214696\_AT | 214696\_at |  | | 214704\_AT | 214704\_at |  | | 214792\_X\_AT | 214792\_x\_at |  | | 214805\_AT | 214805\_at |  | | 214838\_AT | 214838\_at |  | | 214843\_S\_AT | 214843\_s\_at |  | | 214861\_AT | 214861\_at |  | | 214936\_AT | 214936\_at |  | | 215024\_AT | 215024\_at |  | | 215029\_AT | 215029\_at |  | | 215040\_AT | 215040\_at |  | | 215067\_X\_AT | 215067\_x\_at |  | | 215114\_AT | 215114\_at |  | | 215147\_AT | 215147\_at |  | | 215155\_AT | 215155\_at |  | | 215188\_AT | 215188\_at |  | | 215288\_AT | 215288\_at |  | | 215364\_S\_AT | 215364\_s\_at |  | | 215367\_AT | 215367\_at |  | | 215404\_X\_AT | 215404\_x\_at |  | | 215434\_X\_AT | 215434\_x\_at |  | | 215567\_AT | 215567\_at |  | | 215588\_X\_AT | 215588\_x\_at |  | | 215600\_X\_AT | 215600\_x\_at |  | | 215640\_AT | 215640\_at |  | | 215659\_AT | 215659\_at |  | | 215696\_S\_AT | 215696\_s\_at |  | | 215720\_S\_AT | 215720\_s\_at |  | | 215754\_AT | 215754\_at |  | | 215761\_AT | 215761\_at |  | | 215806\_X\_AT | 215806\_x\_at |  | | 215967\_S\_AT | 215967\_s\_at |  | | 215994\_X\_AT | 215994\_x\_at |  | | 215999\_AT | 215999\_at |  | | 216161\_AT | 216161\_at |  | | 216197\_AT | 216197\_at |  | | 216300\_X\_AT | 216300\_x\_at |  | | 216310\_AT | 216310\_at |  | | 216438\_S\_AT | 216438\_s\_at |  | | 216933\_X\_AT | 216933\_x\_at |  | | 216983\_S\_AT | 216983\_s\_at |  | | 216997\_X\_AT | 216997\_x\_at |  | | 217022\_S\_AT | 217022\_s\_at |  | | 217198\_X\_AT | 217198\_x\_at |  | | 217259\_AT | 217259\_at |  | | 217436\_X\_AT | 217436\_x\_at |  | | 217478\_S\_AT | 217478\_s\_at |  | | 217486\_S\_AT | 217486\_s\_at |  | | 217497\_AT | 217497\_at |  | | 217523\_AT | 217523\_at |  | | 217526\_AT | 217526\_at |  | | 217576\_X\_AT | 217576\_x\_at |  | | 217611\_AT | 217611\_at |  | | 217783\_S\_AT | 217783\_s\_at |  | | 217799\_X\_AT | 217799\_x\_at |  | | 217862\_AT | 217862\_at |  | | 217865\_AT | 217865\_at |  | | 217931\_AT | 217931\_at |  | | 217984\_AT | 217984\_at |  | | 218004\_AT | 218004\_at |  | | 218017\_S\_AT | 218017\_s\_at |  | | 218032\_AT | 218032\_at |  | | 218130\_AT | 218130\_at |  | | 218136\_S\_AT | 218136\_s\_at |  | | 218154\_AT | 218154\_at |  | | 218178\_S\_AT | 218178\_s\_at |  | | 218303\_X\_AT | 218303\_x\_at |  | | 218394\_AT | 218394\_at |  | | 218414\_S\_AT | 218414\_s\_at |  | | 218627\_AT | 218627\_at |  | | 218714\_AT | 218714\_at |  | | 218747\_S\_AT | 218747\_s\_at |  | | 218764\_AT | 218764\_at |  | | 218808\_AT | 218808\_at |  | | 218810\_AT | 218810\_at |  | | 218822\_S\_AT | 218822\_s\_at |  | | 218865\_AT | 218865\_at |  | | 218881\_S\_AT | 218881\_s\_at |  | | 218896\_S\_AT | 218896\_s\_at |  | | 218920\_AT | 218920\_at |  | | 219014\_AT | 219014\_at |  | | 219173\_AT | 219173\_at |  | | 219221\_AT | 219221\_at |  | | 219229\_AT | 219229\_at |  | | 219242\_AT | 219242\_at |  | | 219359\_AT | 219359\_at |  | | 219417\_S\_AT | 219417\_s\_at |  | | 219435\_AT | 219435\_at |  | | 219442\_AT | 219442\_at |  | | 219451\_AT | 219451\_at |  | | 219460\_S\_AT | 219460\_s\_at |  | | 219520\_S\_AT | 219520\_s\_at |  | | 219680\_AT | 219680\_at |  | | 219681\_S\_AT | 219681\_s\_at |  | | 219690\_AT | 219690\_at |  | | 219694\_AT | 219694\_at |  | | 219812\_AT | 219812\_at |  | | 219844\_AT | 219844\_at |  | | 219906\_AT | 219906\_at |  | | 219981\_X\_AT | 219981\_x\_at |  | | 219999\_AT | 219999\_at |  | | 220078\_AT | 220078\_at |  | | 220297\_AT | 220297\_at |  | | 220400\_AT | 220400\_at |  | | 220486\_X\_AT | 220486\_x\_at |  | | 220560\_AT | 220560\_at |  | | 220918\_AT | 220918\_at |  | | 220941\_S\_AT | 220941\_s\_at |  | | 220995\_AT | 220995\_at |  | | 221080\_S\_AT | 221080\_s\_at |  | | 221081\_S\_AT | 221081\_s\_at |  | | 221185\_S\_AT | 221185\_s\_at |  | | 221564\_AT | 221564\_at |  | | 221601\_S\_AT | 221601\_s\_at |  | | 221616\_S\_AT | 221616\_s\_at |  | | 221671\_X\_AT | 221671\_x\_at |  | | 221755\_AT | 221755\_at |  | | 221757\_AT | 221757\_at |  | | 221804\_S\_AT | 221804\_s\_at |  | | 221834\_AT | 221834\_at |  | | 221848\_AT | 221848\_at |  | | 221864\_AT | 221864\_at |  | | 221899\_AT | 221899\_at |  | | 221903\_S\_AT | 221903\_s\_at |  | | 221904\_AT | 221904\_at |  | | 221924\_AT | 221924\_at |  | | 221951\_AT | 221951\_at |  | | 221983\_AT | 221983\_at |  | | 222024\_S\_AT | 222024\_s\_at |  | | 222030\_AT | 222030\_at |  | | 222244\_S\_AT | 222244\_s\_at |  | | 222307\_AT | 222307\_at |  | | 222311\_S\_AT | 222311\_s\_at |  | | 32091\_AT | 32091\_at |  | | 32811\_AT | 32811\_at |  | | 35150\_AT | 35150\_at |  | | 36129\_AT | 36129\_at |  | | 36994\_AT | 36994\_at |  | | 37796\_AT | 37796\_at |  | | 38964\_R\_AT | 38964\_r\_at |  | | 40446\_AT | 40446\_at |  | | 46256\_AT | 46256\_at |  | | 52169\_AT | 52169\_at |  | | 55616\_AT | 55616\_at |  | | 56829\_AT | 56829\_at |  | | 58780\_S\_AT | 58780\_s\_at |  | | 60528\_AT | 60528\_at |  | | 71933\_AT | 71933\_at |  | | ABCA7 | 219577\_s\_at |  | | ABCG1 | 204567\_s\_at | GO:0006869 GO:0008203 GO:0009720 GO:0010033 GO:0042632 | | ABHD2 | 63825\_at | GO:0000004 | | ABR | 212895\_s\_at | GO:0007264 GO:0035023 | | ACADVL | 200710\_at | GO:0006118 GO:0006629 GO:0006631 GO:0006635 GO:0015980 | | ACTR2 | 200727\_s\_at |  | | ADRB2 | 206170\_at | GO:0000187 GO:0006898 GO:0007171 GO:0007186 GO:0007188 GO:0007190 GO:0007243 GO:0008333 | | AFTIPHILIN | 217939\_s\_at | GO:0015031 | | AHNAK | 211986\_at | GO:0007399 | | AIM1 | 212543\_at | GO:0000004 | | AKAP11 | 215336\_at | GO:0007243 | | ALMS1 | 214707\_x\_at | GO:0000004 | | AMPD2 | 212360\_at | GO:0006163 GO:0009168 | | AMT | 204294\_at | GO:0006546 | | ANKFY1 | 219868\_s\_at | GO:0006897 | | ANKRD11 | 219437\_s\_at |  | | ANKRD12 | 212286\_at |  | | ANXA1 | 201012\_at | GO:0006629 GO:0006916 GO:0006928 GO:0006954 GO:0007166 | | AOAH | 205639\_at | GO:0006629 GO:0006954 | | AP1M2 | 65517\_at | GO:0006605 GO:0006897 GO:0006903 | | APBB1IP | 219994\_at | GO:0007165 | | APBB3 | 204650\_s\_at | GO:0007242 | | APH1B | 221036\_s\_at | GO:0007219 GO:0016485 GO:0043085 | | APOB48R | 220023\_at |  | | APOL3 | 221087\_s\_at | GO:0006869 GO:0006954 GO:0042157 GO:0043123 | | APRIN | 215888\_at |  | | APS | 205367\_at | GO:0007242 GO:0019735 | | ARHGAP1 | 202117\_at | GO:0007010 GO:0007266 | | ARHGAP12 | 207606\_s\_at |  | | ARHGAP26 | 205068\_s\_at | GO:0007399 GO:0030036 | | ARHGDIB | 201288\_at | GO:0006928 GO:0006955 GO:0007162 GO:0007266 GO:0007275 GO:0030036 | | ARHGEF18 | 213039\_at | GO:0007264 GO:0008360 GO:0030036 GO:0035023 | | ARL6IP5 | 200760\_s\_at | GO:0015813 | | ARPC1A | 215457\_at | GO:0030036 | | ARSA | 204443\_at | GO:0006629 GO:0006665 GO:0008152 | | ARSD | 206831\_s\_at | GO:0008152 | | ATG16L1 | 220521\_s\_at | GO:0006914 GO:0015031 | | ATG7 | 218673\_s\_at | GO:0006497 GO:0006512 GO:0006914 GO:0006944 GO:0015031 GO:0031401 | | ATP10D | 213238\_at | GO:0006812 | | ATP2B4 | 212135\_s\_at | GO:0006812 GO:0006816 GO:0008152 | | ATP6V1B2 | 201089\_at | GO:0006811 GO:0015986 GO:0015988 | | ATP8B2 | 216873\_s\_at | GO:0006812 GO:0008152 | | ATP8B4 | 220416\_at | GO:0006812 | | ATXN1 | 203231\_s\_at |  | | ATXN7 | 209964\_s\_at | GO:0006997 GO:0007601 | | AXIN1 | 212849\_at | GO:0006915 GO:0007049 GO:0007222 GO:0007275 GO:0007309 GO:0045786 | | AZU1 | 214575\_s\_at | GO:0001774 GO:0006508 GO:0006916 GO:0006935 GO:0007205 GO:0008347 GO:0042117 GO:0042535 GO:0043114 GO:0045123 GO:0045348 GO:0045785 GO:0048246 GO:0050725 GO:0050754 GO:0050766 GO:0050829 GO:0050896 GO:0050930 | | B2M | 216231\_s\_at | GO:0006955 GO:0019883 GO:0019885 | | B3GALT4 | 210205\_at | GO:0006486 | | BACE1 | 217904\_s\_at | GO:0006509 GO:0050435 | | BAT1 | 212384\_at | GO:0006754 GO:0006811 GO:0015992 | | BATF | 205965\_at | GO:0006350 GO:0006355 GO:0019735 | | BAZ1A | 217985\_s\_at | GO:0006350 GO:0006355 | | BCL2A1 | 205681\_at | GO:0006916 GO:0042981 | | BCL6 | 203140\_at | GO:0000122 GO:0006350 GO:0006355 GO:0006954 GO:0008284 | | BIN2 | 219191\_s\_at |  | | BIN3 | 222199\_s\_at | GO:0000917 GO:0007015 GO:0007049 GO:0008104 GO:0009826 | | BIRC1 | 204860\_s\_at | GO:0006915 GO:0006916 GO:0007399 | | BIRC2 | 202076\_at | GO:0006916 GO:0007166 GO:0042981 GO:0043123 | | BIRC4BP | 206133\_at |  | | BLCAP | 201032\_at |  | | BNIP3L | 221479\_s\_at | GO:0006915 GO:0006917 GO:0008634 GO:0051607 | | BST1 | 205715\_at | GO:0006959 GO:0007275 | | BTBD5 | 220374\_at |  | | BTG1 | 200920\_s\_at | GO:0007286 GO:0008285 GO:0016477 GO:0030308 GO:0042981 GO:0043085 GO:0045449 GO:0045603 GO:0045663 GO:0045766 | | C14ORF118 | 219720\_s\_at |  | | C14ORF159 | 218298\_s\_at |  | | C14ORF92 | 201683\_x\_at | GO:0006355 | | C16ORF30 | 219315\_s\_at |  | | C16ORF7 | 205781\_at | GO:0015986 | | C1ORF41 | 214163\_at | GO:0007155 | | C1RL | 218983\_at | GO:0006508 | | C20ORF67 | 222044\_at |  | | C3AR1 | 209906\_at | GO:0006928 GO:0006935 GO:0006939 GO:0006954 GO:0006968 GO:0007165 GO:0007186 GO:0007204 GO:0008015 GO:0050896 | | C3ORF29 | 222143\_s\_at | GO:0006470 | | C5ORF4 | 48030\_i\_at | GO:0008152 | | C5ORF5 | 218518\_at |  | | C6ORF111 | 212176\_at |  | | C9ORF7 | 61874\_at | GO:0000004 | | CALCOCO1 | 209002\_s\_at | GO:0006350 GO:0007165 GO:0030518 | | CAMK2G | 214322\_at | GO:0006468 GO:0030073 | | CAP1 | 213798\_s\_at | GO:0007163 GO:0007165 GO:0007190 | | CARD15 | 220066\_at | GO:0006952 GO:0009595 GO:0042981 GO:0043123 GO:0051092 GO:0051259 | | CARD4 | 221073\_s\_at | GO:0006952 GO:0006954 GO:0007165 GO:0009595 GO:0042981 GO:0043123 GO:0051259 | | CASP1 | 206011\_at | GO:0006508 GO:0007165 GO:0042981 GO:0043123 | | CASP4 | 213596\_at | GO:0006508 GO:0006917 GO:0042981 | | CASP8 | 213373\_s\_at | GO:0006508 GO:0008632 GO:0042981 GO:0043123 | | CCL4 | 204103\_at | GO:0006928 GO:0006935 GO:0006954 GO:0007155 GO:0007163 GO:0007165 GO:0007267 GO:0009615 GO:0019079 GO:0050896 | | CCNL1 | 220046\_s\_at | GO:0000074 GO:0006350 GO:0006355 | | CCR7 | 206337\_at | GO:0006935 GO:0006954 GO:0007165 GO:0007186 GO:0007204 GO:0019735 | | CD19 | 206398\_s\_at | GO:0006968 GO:0007166 | | CD2 | 205831\_at | GO:0001766 GO:0006917 GO:0007166 GO:0016337 GO:0030101 GO:0030887 GO:0042110 GO:0045580 | | CD244 | 220307\_at | GO:0006968 GO:0007165 | | CD28 | 211856\_x\_at | GO:0006959 GO:0007166 GO:0042089 GO:0042102 GO:0045070 GO:0045086 GO:0045727 GO:0045768 GO:0045840 | | CD302 | 203799\_at |  | | CD37 | 204192\_at | GO:0006487 | | CD38 | 205692\_s\_at | GO:0006091 GO:0007165 GO:0008624 | | CD3E | 205456\_at | GO:0006461 GO:0007172 GO:0007186 GO:0042102 GO:0042110 GO:0042981 | | CD3Z | 210031\_at | GO:0007166 | | CD53 | 203416\_at | GO:0007165 GO:0019735 | | CD6 | 213958\_at | GO:0006955 GO:0007155 | | CD63 | 200663\_at |  | | CD68 | 203507\_at |  | | CD74 | 209619\_at | GO:0001516 GO:0006457 GO:0006461 GO:0006886 GO:0006955 GO:0007165 GO:0008283 GO:0016064 GO:0019883 GO:0043030 GO:0043066 GO:0045058 | | CD82 | 203904\_x\_at |  | | CD97 | 202910\_s\_at | GO:0006928 GO:0006954 GO:0007155 GO:0007218 GO:0007267 | | CDC2L6 | 212897\_at | GO:0006468 | | CDC42 | 208727\_s\_at | GO:0000074 GO:0001558 GO:0007049 GO:0051318 | | CDIPT | 201253\_s\_at | GO:0008654 | | CECR1 | 219505\_at | GO:0007275 GO:0009168 | | CENTA2 | 219358\_s\_at | GO:0007507 GO:0043087 | | CENTD2 | 212516\_at | GO:0007165 GO:0043087 | | CFD | 205382\_s\_at | GO:0006508 GO:0006957 GO:0045087 | | CHD3 | 208806\_at | GO:0006333 GO:0006350 GO:0006357 GO:0007001 GO:0016568 | | CHKB | 204193\_at |  | | CHMP6 | 218743\_at | GO:0015031 | | CHN2 | 213385\_at | GO:0007242 | | CHRNA7 | 210123\_s\_at | GO:0000187 GO:0006811 GO:0007165 GO:0007268 | | CHRNE | 214246\_x\_at | GO:0006811 GO:0006936 GO:0007165 GO:0007271 | | CIC | 212784\_at | GO:0006355 | | CIDEC | 219398\_at | GO:0006915 GO:0006917 | | CIRBP | 200810\_s\_at | GO:0009409 | | CLC | 206207\_at | GO:0006644 GO:0007275 GO:0016042 GO:0019735 | | CMTM6 | 217947\_at | GO:0006935 GO:0050896 | | CNTN2 | 206970\_at | GO:0007155 | | COL4A3BP | 219625\_s\_at | GO:0006468 GO:0006955 | | CPNE3 | 202118\_s\_at | GO:0006629 GO:0016192 | | CPVL | 208146\_s\_at | GO:0006508 | | CRIP1 | 205081\_at | GO:0008283 GO:0019735 | | CRISPLD2 | 221541\_at |  | | CSAD | 221139\_s\_at | GO:0019752 | | CSF1R | 203104\_at | GO:0006468 GO:0007165 GO:0007169 GO:0007275 GO:0008283 GO:0019735 | | CSF2RA | 210340\_s\_at |  | | CSF2RB | 205159\_at | GO:0007165 GO:0007585 GO:0019221 | | CSF3R | 203591\_s\_at | GO:0006952 GO:0007155 GO:0007165 | | CSPG2 | 221731\_x\_at | GO:0007155 GO:0007275 GO:0008037 | | CST3 | 201360\_at |  | | CTDSP1 | 217844\_at | GO:0000004 | | CTSG | 205653\_at | GO:0006508 GO:0006955 | | CTSK | 202450\_s\_at | GO:0006508 | | CTSO | 203758\_at | GO:0006508 | | CXCR3 | 207681\_at | GO:0006928 GO:0006935 GO:0007155 GO:0007165 GO:0007186 GO:0007204 GO:0019735 | | CYB561D2 | 209665\_at | GO:0006118 GO:0006511 GO:0007096 GO:0008054 | | CYBA | 203028\_s\_at | GO:0006118 GO:0006801 | | CYBB | 203922\_s\_at | GO:0006118 GO:0006811 GO:0006954 GO:0019735 | | CYSLTR2 | 220813\_at | GO:0006955 GO:0007165 GO:0007186 | | DAPP1 | 219290\_x\_at | GO:0006470 GO:0007242 | | DDX17 | 213998\_s\_at | GO:0006396 | | DEFA1 | 205033\_s\_at | GO:0006805 GO:0009615 GO:0042742 GO:0050832 | | DERL2 | 218333\_at | GO:0008284 GO:0030307 GO:0030433 GO:0030968 GO:0030970 | | DGKA | 211272\_s\_at | GO:0007205 GO:0007242 | | DGKG | 206395\_at | GO:0007205 GO:0007242 | | DGKZ | 207556\_s\_at | GO:0007205 GO:0007242 | | DHRSX | 203043\_at | GO:0008152 | | DNAJC13 | 212467\_at | GO:0006457 | | DNAJC3 | 208499\_s\_at | GO:0006445 GO:0006457 GO:0006952 GO:0006986 GO:0009615 | | DOCK5 | 219921\_s\_at | GO:0007165 | | DOK1 | 216835\_s\_at | GO:0007166 GO:0007169 | | DPEP2 | 219452\_at | GO:0006508 | | DPH1 | 222041\_at | GO:0006412 | | DUSP22 | 218845\_at | GO:0006470 | | EAF2 | 219551\_at | GO:0006350 GO:0006355 GO:0006915 | | ECHDC2 | 218552\_at | GO:0008152 | | EDG4 | 206723\_s\_at | GO:0007165 GO:0007186 GO:0007202 GO:0007204 | | EGR1 | 201693\_s\_at | GO:0006350 GO:0006355 | | EGR3 | 206115\_at | GO:0006350 GO:0006355 GO:0007517 GO:0007623 | | EHD1 | 209039\_x\_at | GO:0000004 | | EHD3 | 218935\_at |  | | EIF2C4 | 219190\_s\_at | GO:0006412 | | ELA2 | 206871\_at | GO:0006508 GO:0006874 GO:0009411 GO:0030163 GO:0030236 GO:0043406 GO:0045079 GO:0045415 GO:0045416 GO:0048661 GO:0050728 GO:0050922 | | ELF1 | 212420\_at | GO:0006350 GO:0006355 | | ELMO2 | 221528\_s\_at | GO:0006909 GO:0006915 | | EMR2 | 207610\_s\_at | GO:0007165 GO:0007218 | | ENTPD4 | 204076\_at | GO:0006256 | | EP300 | 213579\_s\_at | GO:0001666 GO:0006355 GO:0006915 GO:0007049 GO:0007165 GO:0007399 GO:0042592 GO:0051091 | | ERG | 213541\_s\_at | GO:0006350 GO:0006355 GO:0006468 GO:0007165 GO:0007275 GO:0008283 | | EVI2B | 211742\_s\_at |  | | EXOC7 | 214802\_at | GO:0006887 GO:0015031 | | EZH1 | 203249\_at | GO:0006350 GO:0006355 GO:0009653 | | F13A1 | 203305\_at | GO:0007596 GO:0018149 | | F8 | 205756\_s\_at | GO:0006953 GO:0007155 GO:0007596 | | FALZ | 207186\_s\_at | GO:0000122 GO:0006355 GO:0007399 | | FAM108A1 | 221267\_s\_at | GO:0006629 GO:0006631 | | FAM26B | 221565\_s\_at |  | | FAM65A | 45749\_at |  | | FAM89B | 32209\_at |  | | FBP2 | 206844\_at | GO:0005975 GO:0006000 GO:0006094 | | FBXL5 | 209004\_s\_at | GO:0016567 | | FBXO9 | 210638\_s\_at | GO:0016567 | | FCER1G | 204232\_at | GO:0006955 GO:0007166 | | FCGRT | 218831\_s\_at | GO:0007565 GO:0019882 | | FCN1 | 205237\_at | GO:0006817 GO:0008228 | | FES | 205418\_at | GO:0006468 GO:0007242 GO:0007275 GO:0008283 | | FEZ2 | 202305\_s\_at | GO:0030097 | | FGL2 | 204834\_at |  | | FKBP11 | 219117\_s\_at | GO:0006457 | | FLJ11151 | 218610\_s\_at |  | | FLJ12788 | 218838\_s\_at |  | | FLJ21616 | 219269\_at | GO:0006355 | | FLJ21865 | 65635\_at | GO:0005975 | | FLNA | 214752\_x\_at | GO:0006928 GO:0007166 GO:0007399 GO:0030036 GO:0043123 | | FNBP1 | 212288\_at | GO:0006412 | | FOS | 209189\_at | GO:0006306 GO:0006357 GO:0006954 | | FOSB | 202768\_at | GO:0000074 GO:0000122 GO:0006355 GO:0007275 GO:0007610 | | FPR1 | 205119\_s\_at | GO:0000187 GO:0006928 GO:0006935 GO:0006954 GO:0007165 GO:0007186 GO:0007188 GO:0007263 GO:0050896 | | FRAT1 | 219889\_at | GO:0016055 | | FRY | 204072\_s\_at |  | | FXYD5 | 217655\_at | GO:0006811 GO:0030033 GO:0046588 | | FXYD6 | 217897\_at | GO:0000004 GO:0006811 | | GAA | 202812\_at | GO:0005975 GO:0005980 GO:0006091 | | GABARAP | 200645\_at | GO:0006605 GO:0007268 GO:0015031 | | GABBR1 | 203146\_s\_at | GO:0007165 GO:0007194 GO:0007214 | | GALNS | 206335\_at | GO:0008152 GO:0030203 | | GALNT10 | 212256\_at |  | | GBA | 210589\_s\_at | GO:0005975 GO:0006629 GO:0006665 GO:0007040 | | GBP2 | 202748\_at | GO:0006955 | | GCH1 | 204224\_s\_at | GO:0006559 GO:0006729 GO:0006809 GO:0042133 | | GCNT1 | 205505\_at | GO:0006493 | | GLIPR1 | 204222\_s\_at |  | | GLTP | 219267\_at | GO:0046836 | | GMFG | 204220\_at | GO:0006468 | | GMIP | 218913\_s\_at | GO:0007242 | | GNAQ | 202615\_at | GO:0006471 GO:0007165 GO:0007186 GO:0007202 GO:0007596 | | GNS | 212334\_at | GO:0006027 GO:0008152 | | GPD2 | 210007\_s\_at | GO:0006007 GO:0006072 GO:0006118 | | GPR109B | 205220\_at | GO:0007165 GO:0007186 | | GPR162 | 205056\_s\_at | GO:0007165 GO:0007186 | | GPSM3 | 204265\_s\_at | GO:0007165 | | GSK3B | 209945\_s\_at | GO:0005977 GO:0006468 GO:0016055 | | GZMK | 206666\_at | GO:0006508 | | GZMM | 207460\_at | GO:0006508 GO:0019835 | | H1F0 | 208886\_at | GO:0006334 GO:0007001 | | HBB | 211696\_x\_at | GO:0006810 GO:0015671 | | HCK | 208018\_s\_at | GO:0006468 GO:0007242 GO:0007498 | | HCP5 | 206082\_at | GO:0006952 | | HIF1A | 200989\_at | GO:0001666 GO:0006355 GO:0007165 GO:0042592 | | HIPK1 | 212291\_at | GO:0006350 GO:0006355 GO:0006468 | | HIST2H2AA | 214290\_s\_at | GO:0006334 GO:0007001 | | HIST2H2BE | 202708\_s\_at | GO:0006334 GO:0007001 | | HLA-A | 215313\_x\_at | GO:0019883 GO:0019885 | | HLA-B | 208729\_x\_at | GO:0019883 GO:0019885 | | HLA-DPB1 | 201137\_s\_at | GO:0006955 GO:0019884 GO:0019886 | | HLA-DRA | 210982\_s\_at | GO:0006955 GO:0019884 GO:0019886 | | HNRPL | 221860\_at | GO:0006397 | | HOM-TES-103 | 209721\_s\_at |  | | HPS1 | 203308\_x\_at | GO:0007040 GO:0007601 GO:0050896 | | HSMPP8 | 221771\_s\_at | GO:0006333 GO:0007049 | | HTLF | 206708\_at | GO:0006350 GO:0006355 | | ID2 | 213931\_at | GO:0007275 GO:0016481 GO:0043433 | | IFI35 | 209417\_s\_at | GO:0006955 GO:0009615 | | IFNAR2 | 204786\_s\_at | GO:0007166 GO:0007259 GO:0009615 | | IKBKG | 209929\_s\_at | GO:0006350 GO:0006355 GO:0006917 GO:0006955 GO:0007249 | | IL17R | 205707\_at | GO:0007166 | | IL1B | 39402\_at | GO:0000074 GO:0001660 GO:0006915 GO:0007165 GO:0007267 GO:0008283 GO:0008285 GO:0019735 | | IL2RB | 205291\_at | GO:0006461 GO:0007165 GO:0019221 GO:0045885 | | IL3RA | 206148\_at | GO:0006468 GO:0007275 | | IL4R | 203233\_at | GO:0006955 GO:0007165 | | IL6R | 205945\_at | GO:0006955 GO:0007166 GO:0007275 GO:0008283 | | IL7R | 205798\_at | GO:0000018 GO:0006955 GO:0007166 GO:0019735 | | ING4 | 48825\_at | GO:0006473 GO:0006915 GO:0007049 GO:0007050 GO:0008285 GO:0045892 GO:0045926 | | IQGAP1 | 213446\_s\_at | GO:0007165 GO:0007264 GO:0046580 | | IRAK3 | 220034\_at | GO:0006468 GO:0007165 GO:0019221 GO:0043244 | | IRF1 | 202531\_at | GO:0006350 GO:0006355 GO:0006366 GO:0006955 GO:0007049 GO:0045786 | | ISG20 | 33304\_at | GO:0000738 GO:0006401 GO:0008283 GO:0009615 | | ITGAE | 205055\_at | GO:0007155 GO:0007159 GO:0007229 | | ITIH4 | 206287\_s\_at | GO:0006953 GO:0030212 | | ITPKB | 203723\_at | GO:0007165 | | ITSN2 | 209907\_s\_at | GO:0006897 GO:0035023 | | JAK2 | 205842\_s\_at | GO:0000074 GO:0006468 GO:0006928 GO:0007242 GO:0007259 GO:0007498 | | JUNB | 201473\_at | GO:0006350 GO:0006357 | | KCTD2 | 34858\_at | GO:0006813 | | KIAA0247 | 202181\_at | GO:0000004 | | KIAA0317 | 202128\_at | GO:0006512 | | KIAA0494 | 201775\_s\_at |  | | KIAA0553 | 212487\_at | GO:0000004 | | KIAA0804 | 209553\_at | GO:0006512 | | KIDINS220 | 212162\_at | GO:0015977 | | KIR2DL2 | 211397\_x\_at | GO:0006955 | | KLF13 | 219878\_s\_at | GO:0006350 GO:0006355 GO:0006366 | | KLF2 | 219371\_s\_at | GO:0006350 GO:0006355 | | KLF4 | 221841\_s\_at | GO:0006350 GO:0007500 GO:0008285 GO:0045892 | | KLF6 | 208961\_s\_at | GO:0006350 GO:0006355 GO:0030183 | | KLF7 | 204334\_at | GO:0006350 GO:0006357 | | KLRB1 | 214470\_at | GO:0007166 GO:0019735 | | KLRK1 | 205821\_at | GO:0006968 | | LAIR1 | 210644\_s\_at |  | | LAIR2 | 207509\_s\_at |  | | LASP1 | 200618\_at | GO:0006811 GO:0030865 | | LCK | 204891\_s\_at | GO:0000074 GO:0006468 GO:0006882 GO:0006917 GO:0006919 GO:0007242 GO:0007265 GO:0030097 GO:0030217 GO:0042493 GO:0050862 GO:0050870 GO:0051209 GO:0051249 | | LENG4 | 209179\_s\_at | GO:0000004 | | LGALS3 | 208949\_s\_at |  | | LILRA2 | 207857\_at | GO:0006955 GO:0007165 | | LILRB1 | 211336\_x\_at | GO:0006955 GO:0009615 | | LNPEP | 207904\_s\_at | GO:0006508 GO:0007267 GO:0007565 | | LRPAP1 | 201186\_at | GO:0006457 GO:0008283 GO:0016192 | | LTB | 207339\_s\_at | GO:0006955 GO:0007165 GO:0007267 | | LTB4R | 210128\_s\_at | GO:0006928 GO:0006936 GO:0006954 GO:0007165 GO:0007200 | | LTC4S | 206480\_at | GO:0019370 | | LXN | 218729\_at |  | | LY6G5C | 219860\_at |  | | LY75 | 205668\_at | GO:0006897 GO:0006954 | | MAL | 204777\_s\_at | GO:0001766 GO:0006917 GO:0007165 GO:0007417 GO:0007516 GO:0030154 GO:0042552 GO:0045176 | | MAN2A1 | 205105\_at | GO:0005975 GO:0006013 GO:0009101 | | MANBA | 203778\_at | GO:0005975 GO:0006464 | | MAP3K11 | 203652\_at | GO:0000080 GO:0007017 GO:0007257 GO:0008283 GO:0046777 GO:0051259 | | MAP3K14 | 205192\_at | GO:0006468 | | MAP3K3 | 203514\_at | GO:0000165 GO:0043123 GO:0046777 | | MAP3K5 | 203836\_s\_at | GO:0000165 GO:0006468 GO:0006915 GO:0006950 GO:0007257 GO:0008624 | | MAP4K4 | 206571\_s\_at | GO:0006468 GO:0006950 GO:0007243 | | MAPK3 | 212046\_x\_at | GO:0000074 GO:0006468 GO:0007049 | | MARCH2 | 210075\_at |  | | MARCH8 | 221824\_s\_at |  | | MARK2 | 203942\_s\_at | GO:0006468 GO:0007243 GO:0030154 GO:0045197 | | MAST3 | 213045\_at | GO:0006468 | | MAWBP | 219543\_at | GO:0000004 GO:0009058 | | MCL1 | 200797\_s\_at | GO:0001709 GO:0006916 GO:0008632 GO:0019725 GO:0030154 GO:0042981 | | METTL3 | 213653\_at | GO:0001510 GO:0006139 | | MGAT1 | 201126\_s\_at | GO:0005975 GO:0006023 GO:0006487 | | MGC15523 | 212890\_at | GO:0006865 | | MGC3123 | 218419\_s\_at | GO:0006464 | | MGLL | 211026\_s\_at | GO:0006629 GO:0006725 GO:0006954 | | MICAL1 | 218376\_s\_at | GO:0006118 GO:0006725 GO:0007010 GO:0007165 | | MKNK1 | 209467\_s\_at | GO:0006445 GO:0006468 GO:0007243 | | MNDA | 204959\_at | GO:0006350 GO:0006355 GO:0006968 | | MNT | 204206\_at | GO:0000074 GO:0006355 GO:0006366 GO:0007275 GO:0008285 | | MS4A1 | 210356\_x\_at | GO:0006955 GO:0007165 GO:0042113 | | MSL3L1 | 207551\_s\_at | GO:0006333 GO:0006350 GO:0006355 GO:0006366 GO:0007275 GO:0016568 | | MSRA | 219281\_at | GO:0006464 GO:0006555 GO:0006979 GO:0019538 | | MTMR9 | 204837\_at | GO:0046839 | | MVP | 202180\_s\_at | GO:0042493 | | MXD1 | 206877\_at | GO:0006355 GO:0007275 GO:0008283 | | MYD88 | 209124\_at | GO:0006954 GO:0007166 GO:0043123 | | MYH9 | 211926\_s\_at | GO:0007605 GO:0008360 | | MYO5A | 204527\_at | GO:0006810 GO:0030048 | | MYST3 | 202423\_at | GO:0006323 GO:0006334 GO:0006350 GO:0006355 GO:0016568 | | NAGK | 218231\_at | GO:0006044 GO:0006051 | | NAP1L2 | 219368\_at | GO:0006334 | | NBEAL2 | 212443\_at |  | | NBN | 202905\_x\_at | GO:0000077 GO:0000723 GO:0001701 GO:0001832 GO:0006302 GO:0007050 GO:0007095 GO:0030174 GO:0030330 GO:0031575 GO:0045190 GO:0048145 | | NCF2 | 209949\_at | GO:0006801 GO:0006968 | | NCOA4 | 210774\_s\_at | GO:0006350 GO:0007169 GO:0008584 GO:0030521 GO:0045893 | | NDRG1 | 200632\_s\_at | GO:0010038 GO:0030154 | | NDST2 | 214867\_at |  | | NFIL3 | 203574\_at | GO:0006355 GO:0006366 GO:0006955 | | NFKBIA | 201502\_s\_at | GO:0006915 GO:0007253 GO:0009618 GO:0042345 GO:0043392 | | NGFRAP1 | 217963\_s\_at | GO:0006915 GO:0007275 | | NINJ1 | 203045\_at | GO:0007155 GO:0007399 GO:0042246 | | NINJ2 | 219594\_at | GO:0007155 GO:0007158 GO:0007399 GO:0042246 | | NISCH | 201591\_s\_at | GO:0007242 | | NKG7 | 213915\_at |  | | NKTR | 202379\_s\_at | GO:0006457 | | NOTCH1 | 218902\_at | GO:0006355 GO:0006955 GO:0007219 GO:0030154 GO:0045662 GO:0050793 | | NOTCH2NL | 214722\_at |  | | NR4A2 | 204621\_s\_at | GO:0006350 GO:0006355 GO:0007165 GO:0019735 | | NRGN | 204081\_at | GO:0007165 GO:0007399 | | NSD1 | 219084\_at | GO:0000122 GO:0006350 GO:0016568 GO:0016571 GO:0045893 | | NUDT18 | 219665\_at |  | | NXF1 | 208922\_s\_at | GO:0006397 GO:0006406 GO:0006810 | | OBFC2A | 219334\_s\_at |  | | OGFRL1 | 219582\_at |  | | P2RX1 | 210401\_at | GO:0006811 GO:0006915 GO:0007165 GO:0007268 | | PABPN1 | 213046\_at | GO:0006397 GO:0006936 | | PAM | 212958\_x\_at | GO:0006464 GO:0006518 | | PCAF | 203845\_at | GO:0006338 GO:0006350 GO:0006355 GO:0006473 GO:0007049 GO:0007050 GO:0008285 | | PCBP3 | 205663\_at | GO:0016071 | | PCGF3 | 212753\_at |  | | PCYT1A | 204209\_at | GO:0008654 GO:0009058 | | PDE6G | 210060\_at | GO:0007601 GO:0050896 | | PDLIM2 | 219165\_at |  | | PDPK1 | 204524\_at | GO:0006468 GO:0008286 GO:0030036 | | PECAM1 | 208981\_at | GO:0006928 GO:0007155 GO:0007165 GO:0008037 | | PFC | 206380\_s\_at | GO:0006957 GO:0042742 GO:0045087 | | PHC2 | 200919\_at | GO:0007275 | | PHF11 | 221816\_s\_at | GO:0006355 | | PHKA2 | 209439\_s\_at | GO:0005975 GO:0005977 GO:0006091 GO:0006464 | | PILRA | 222218\_s\_at | GO:0007165 GO:0007171 | | PILRB | 220954\_s\_at | GO:0007171 | | PINK1 | 209019\_s\_at | GO:0006468 GO:0006950 GO:0007243 | | PIP5K1C | 212518\_at |  | | PITPNC1 | 219155\_at | GO:0006810 | | PLEC1 | 201373\_at | GO:0007016 | | PLEKHM1 | 212717\_at | GO:0007242 | | PLK3 | 204958\_at | GO:0000074 GO:0006468 | | PLP2 | 201136\_at | GO:0006811 GO:0006935 GO:0019221 | | PLSCR3 | 218828\_at | GO:0017121 | | PLXNC1 | 213241\_at | GO:0007155 GO:0007275 | | PNRC1 | 209034\_at | GO:0006350 GO:0006355 | | PPBP | 214146\_s\_at | GO:0000074 GO:0006935 GO:0006955 GO:0008283 GO:0015758 GO:0042742 GO:0050896 | | PPGB | 200661\_at | GO:0006508 GO:0006886 | | PPM1A | 210407\_at | GO:0006470 GO:0043123 | | PPM2C | 218273\_s\_at | GO:0006470 | | PPP1R12A | 201602\_s\_at | GO:0006937 | | PPP1R15A | 37028\_at | GO:0006915 GO:0006974 GO:0007050 | | PPP1R3D | 204555\_s\_at | GO:0005975 GO:0005977 | | PRDM2 | 203056\_s\_at | GO:0006355 | | PRRG4 | 207291\_at | GO:0000004 | | PSCDBP | 209606\_at | GO:0030155 | | PSMB9 | 204279\_at | GO:0006508 GO:0006511 GO:0006955 | | PSTPIP1 | 211178\_s\_at | GO:0007155 GO:0007165 GO:0009613 | | PTAFR | 206278\_at | GO:0006935 GO:0006954 GO:0007165 GO:0007186 GO:0019735 GO:0048015 GO:0050896 | | PTGER2 | 206631\_at | GO:0007165 GO:0007186 | | PTGER4 | 204897\_at | GO:0006955 GO:0007165 GO:0007186 GO:0007188 | | PTGIR | 206187\_at | GO:0007165 GO:0007187 GO:0007267 | | PTGS2 | 204748\_at | GO:0006633 GO:0006928 GO:0007582 GO:0008217 GO:0019371 GO:0030216 GO:0050727 | | PTPRC | 212587\_s\_at | GO:0001915 GO:0001960 GO:0006469 GO:0006470 GO:0007166 GO:0030217 GO:0042100 GO:0042102 GO:0045860 GO:0050852 GO:0050853 GO:0050857 GO:0051209 GO:0051607 GO:0051726 | | PTPRE | 221840\_at | GO:0006470 | | PTX3 | 206157\_at | GO:0006954 | | QSCN6 | 201482\_at | GO:0001558 GO:0006118 | | RAB4B | 219807\_x\_at | GO:0007264 GO:0015031 GO:0016192 | | RAB8B | 219210\_s\_at | GO:0007264 GO:0015031 | | RABL4 | 213784\_at | GO:0007264 | | RAF1 | 201244\_s\_at | GO:0006468 GO:0006915 GO:0007242 GO:0008283 | | RAP2B | 213923\_at | GO:0007264 | | RARRES3 | 204070\_at | GO:0008285 | | RAVER2 | 201648\_at |  | | RBL2 | 212332\_at | GO:0006350 GO:0006355 GO:0007049 GO:0045786 | | RBM5 | 209936\_at | GO:0006396 GO:0007049 GO:0045786 | | RBMS1 | 215127\_s\_at | GO:0006260 GO:0006396 GO:0006445 | | REC8L1 | 218599\_at | GO:0007001 GO:0007062 GO:0007126 GO:0007131 GO:0007283 | | RERE | 200940\_s\_at | GO:0000004 GO:0006355 GO:0007275 | | RGC32 | 218723\_s\_at | GO:0000079 | | RGL2 | 209110\_s\_at | GO:0007265 GO:0051056 | | RGS2 | 202388\_at | GO:0007049 GO:0007169 GO:0008277 GO:0009968 | | RHOB | 212099\_at | GO:0001525 GO:0006927 GO:0007049 GO:0007155 GO:0007264 GO:0008333 GO:0015031 GO:0030154 GO:0045766 GO:0045786 | | RIC8A | 221913\_at |  | | RIN3 | 60471\_at | GO:0006897 GO:0007242 | | RNASE2 | 206111\_at | GO:0006401 GO:0006935 GO:0050896 | | RNASE3 | 206851\_at | GO:0006401 GO:0042742 | | RNASE6 | 213566\_at | GO:0006401 GO:0006952 | | RNASEL | 221287\_at | GO:0006397 GO:0006468 | | RNF10 | 208632\_at |  | | RNF122 | 219897\_at | GO:0006512 | | RNF139 | 209510\_at | GO:0006512 | | RNF167 | 212047\_s\_at | GO:0006508 | | RNF19 | 220483\_s\_at | GO:0000226 GO:0006512 | | RNF38 | 218528\_s\_at |  | | RPS6KA4 | 204632\_at | GO:0006355 GO:0006468 GO:0007243 | | S100A12 | 205863\_at | GO:0006805 GO:0006954 GO:0042742 GO:0050832 | | S100A4 | 203186\_s\_at |  | | S100A6 | 217728\_at | GO:0000074 GO:0007049 GO:0007165 GO:0007267 GO:0007409 GO:0008283 GO:0048146 | | S100A9 | 203535\_at | GO:0006954 GO:0007267 | | SART2 | 218854\_at |  | | SASH1 | 41644\_at | GO:0007049 GO:0045786 | | SAT | 213988\_s\_at |  | | SATB1 | 203408\_s\_at | GO:0006325 GO:0006355 | | SCAMP2 | 218143\_s\_at | GO:0006892 GO:0015031 | | SEC61A2 | 219499\_at | GO:0006605 GO:0009306 GO:0015031 | | SELL | 204563\_at | GO:0006928 GO:0007155 | | SELPLG | 209879\_at | GO:0007155 | | SEMA4A | 219259\_at | GO:0006259 GO:0007399 GO:0030154 | | SEMA4D | 203528\_at | GO:0006916 GO:0006955 GO:0007155 GO:0007399 GO:0030154 | | SERPINB1 | 213572\_s\_at |  | | SERTAD2 | 202656\_s\_at | GO:0006350 GO:0006355 | | SF1 | 210172\_at | GO:0000245 GO:0000389 GO:0006350 GO:0006355 | | SF3B1 | 201070\_x\_at | GO:0000398 | | SFRS2IP | 213850\_s\_at | GO:0000245 GO:0008380 | | SGK3 | 220038\_at | GO:0006468 GO:0006950 GO:0007242 | | SH2B | 40149\_at | GO:0007242 | | SH3BGRL3 | 221269\_s\_at |  | | SIN3B | 39705\_at | GO:0006350 GO:0006355 | | SIRT7 | 218797\_s\_at | GO:0006342 GO:0006355 | | SKIP | 202781\_s\_at | GO:0030036 | | SLC10A3 | 204928\_s\_at | GO:0006810 GO:0006814 GO:0015711 | | SLC15A2 | 205316\_at | GO:0006857 GO:0015031 | | SLC16A5 | 213590\_at | GO:0006810 GO:0015711 GO:0015718 | | SLC22A4 | 205896\_at | GO:0006811 GO:0006814 GO:0007589 GO:0015695 | | SLC27A3 | 222217\_s\_at | GO:0006629 GO:0006631 GO:0008152 | | SLC2A6 | 220091\_at | GO:0008643 | | SLC31A2 | 204204\_at | GO:0006811 GO:0006825 | | SLC36A1 | 213119\_at | GO:0006810 GO:0006865 | | SMA4 | 214850\_at | GO:0000004 GO:0005975 | | SNRP70 | 213121\_at | GO:0000398 GO:0008380 | | SNX19 | 202358\_s\_at | GO:0007242 GO:0015031 | | SNX26 | 213827\_at | GO:0015031 | | SON | 201085\_s\_at | GO:0006916 | | SPEN | 201996\_s\_at | GO:0006355 GO:0007219 | | SPINT2 | 210715\_s\_at | GO:0006928 | | SPON2 | 218638\_s\_at | GO:0006955 GO:0007155 GO:0007275 GO:0007411 | | SPTLC2 | 203127\_s\_at | GO:0009058 | | SQRDL | 217995\_at |  | | SSH3 | 51192\_at | GO:0006470 | | STAT2 | 205170\_at | GO:0006350 GO:0006357 GO:0007242 GO:0007259 GO:0009615 | | STAT3 | 208991\_at | GO:0000122 GO:0006928 GO:0006953 GO:0007242 GO:0007259 GO:0007399 GO:0019221 | | STAT6 | 201331\_s\_at | GO:0006350 GO:0006357 GO:0007242 | | STK10 | 40420\_at | GO:0006468 | | STX11 | 210190\_at | GO:0006886 GO:0006944 | | STX16 | 221499\_s\_at | GO:0006886 GO:0006891 | | SULT1A1 | 203615\_x\_at | GO:0006584 GO:0006629 GO:0008202 GO:0009308 | | SULT1A2 | 207122\_x\_at | GO:0006584 GO:0006629 GO:0008202 GO:0009309 | | SURF1 | 217646\_at | GO:0006118 GO:0008535 GO:0009060 | | SYT11 | 209197\_at | GO:0006810 | | TAP1 | 202307\_s\_at | GO:0006857 GO:0006955 GO:0015031 | | TAPBP | 208829\_at | GO:0006461 GO:0006890 GO:0006955 GO:0019885 GO:0050823 | | TAZ | 37278\_at | GO:0006936 GO:0007507 GO:0007517 GO:0008152 | | TBC1D13 | 44696\_at |  | | TBC1D2 | 222173\_s\_at |  | | TBXAS1 | 208130\_s\_at | GO:0001516 GO:0006118 GO:0006633 GO:0007596 | | TCEAL4 | 202371\_at |  | | TEX2 | 218099\_at | GO:0009401 | | TFEC | 206715\_at | GO:0045449 | | TGFBR2 | 207334\_s\_at | GO:0006468 GO:0006470 GO:0007178 GO:0008284 | | TGOLN2 | 212040\_at |  | | THBS3 | 209561\_at | GO:0006928 GO:0007155 GO:0007160 | | THRAP2 | 212208\_at | GO:0006350 GO:0006355 | | TIMP2 | 203167\_at |  | | TINF2 | 220052\_s\_at | GO:0007004 | | TK2 | 204276\_at | GO:0006139 GO:0006260 | | TLE1 | 203221\_at | GO:0006355 GO:0007165 GO:0007222 GO:0007275 GO:0009887 | | TLN1 | 203254\_s\_at | GO:0006928 GO:0007016 GO:0007043 | | TLR1 | 210176\_at | GO:0007165 GO:0007250 GO:0042116 GO:0042495 GO:0042535 GO:0045087 GO:0045410 | | TLR2 | 204924\_at | GO:0006917 GO:0006954 GO:0007165 GO:0045087 | | TMEM49 | 220990\_s\_at |  | | TMEM66 | 200847\_s\_at |  | | TMEM9B | 218065\_s\_at | GO:0043123 | | TNFRSF14 | 209354\_at | GO:0006915 GO:0006955 GO:0007166 | | TNFRSF7 | 206150\_at | GO:0006915 GO:0006916 GO:0006917 GO:0006955 GO:0007001 GO:0007166 GO:0007283 GO:0008588 GO:0016064 GO:0042100 GO:0045078 GO:0045579 GO:0045582 GO:0046330 GO:0048305 | | TNFSF4 | 207426\_s\_at | GO:0006955 GO:0007165 GO:0007267 GO:0008284 | | TNFSF8 | 207216\_at | GO:0006917 GO:0006955 GO:0007165 GO:0007267 GO:0008283 | | TNRC6B | 213254\_at |  | | TP53AP1 | 210886\_x\_at | GO:0006950 | | TP53I3 | 210609\_s\_at |  | | TRA2A | 213575\_at | GO:0000398 | | TRADD | 1729\_at | GO:0006915 GO:0006917 GO:0007165 GO:0043123 | | TRAF1 | 205599\_at | GO:0006461 GO:0007165 GO:0042981 | | TRAF3IP3 | 215275\_at |  | | TRAF6 | 205558\_at | GO:0006512 GO:0007165 GO:0042981 GO:0043123 | | TRBV3-1 | 211796\_s\_at |  | | TREM1 | 219434\_at | GO:0006959 GO:0007242 | | TRIM22 | 213293\_s\_at | GO:0006355 GO:0006955 GO:0009615 | | TRIM38 | 203567\_s\_at | GO:0043123 | | TRIM66 | 213748\_at | GO:0006355 | | TRIOBP | 216210\_x\_at | GO:0030047 GO:0051016 | | TSC22D3 | 208763\_s\_at | GO:0006355 | | TTBK2 | 213922\_at | GO:0006468 | | TUBA3 | 209118\_s\_at | GO:0007018 GO:0051258 | | TXNDC4 | 208957\_at | GO:0006118 GO:0006457 GO:0006986 GO:0009100 GO:0030503 GO:0045045 | | TYROBP | 204122\_at | GO:0006968 GO:0007242 | | UBE2W | 218521\_s\_at | GO:0006512 | | UBXD2 | 212007\_at | GO:0006355 | | ULK1 | 209333\_at | GO:0006468 GO:0007165 | | UPP1 | 203234\_at | GO:0009116 GO:0009166 | | VAMP1 | 213326\_at | GO:0016192 | | VAMP3 | 201336\_at | GO:0006461 GO:0006904 GO:0006944 | | VAV1 | 206219\_s\_at | GO:0007242 GO:0035023 | | VCPIP1 | 219810\_at | GO:0000160 GO:0006512 | | VPS39 | 212156\_at | GO:0015031 | | WDFY3 | 212602\_at |  | | WHSC1L1 | 218173\_s\_at | GO:0006355 GO:0016049 GO:0030154 | | WIRE | 212049\_at |  | | XKR8 | 218753\_at |  | | ZA20D2 | 210275\_s\_at | GO:0000004 | | ZAP70 | 214032\_at | GO:0006468 GO:0006955 GO:0007243 GO:0045059 GO:0045582 | | ZBTB25 | 214482\_at | GO:0006350 GO:0006355 | | ZBTB7A | 222082\_at | GO:0006350 GO:0045892 GO:0051216 | | ZC3HAV1 | 213051\_at | GO:0006471 | | ZFP36 | 201531\_at | GO:0006402 | | ZFYVE26 | 213073\_at |  | | ZNF137 | 207394\_at | GO:0006350 GO:0006355 | | ZNF167 | 206314\_at | GO:0006350 GO:0006355 | | ZNF185 | 203585\_at |  | | ZNF264 | 205917\_at | GO:0006350 GO:0006355 | | ZNF337 | 214760\_at | GO:0006350 GO:0006355 | | ZNF345 | 207236\_at | GO:0000122 GO:0006350 GO:0006359 | | ZNF350 | 219266\_at | GO:0006350 GO:0006355 | | ZNF430 | 206829\_x\_at | GO:0006350 GO:0006355 | | ZNF44 | 215359\_x\_at | GO:0006350 GO:0006355 | | ZNF447 | 217593\_at | GO:0006355 | | ZNF611 | 208137\_x\_at |  | | ZNF654 | 219239\_s\_at |  | | ZNF671 | 219849\_at | GO:0006355 | | ZNF688 | 213527\_s\_at | GO:0006355 | | ZNF701 | 220242\_x\_at | GO:0006355 | | ZNFN1A1 | 205039\_s\_at | GO:0006350 GO:0006355 GO:0007498 | | ZXDC | 218639\_s\_at |  | | ZYG11BL | 202452\_at |  | | ZZEF1 | 212601\_at | GO:0030071 | |
