# Supplementary material for: Gene expression profiling reveals consistent differences between clinical samples of human leukaemias and their model cell lines
Source: Br J Haematol. 2006 Nov;135(4):520–3. doi: 10.1111/j.1365-2141.2006.06342.x (PMC1654200; doi:10.1111/j.1365-2141.2006.06342.x)
Supplement: Table SIII — GO analysis of top 1000 downregulated genes (underexpression in cell lines compared with clinical samples). [file bjh0135-0520-TableSIII.html]

GOstat by Tim Beissbarth


|  |  |  |  |
| --- | --- | --- | --- |
| *GO*stat *by Tim Bei�barth beissbarth@wehi.edu.au* | Run from 130.223.122.125 Date: Wed Apr 26 01:25:38 2006 GO Annotation @ EBI (GOA) Input: 1000 IDs Search against: AFFY\_HG\_U133A | Unique Genes: 1000 Annotated Genes: 626 GOs: 3927 Unique GOs: 945 All Unique Sub-GOs: 981 | GO-DB: goa\_human Min Sub-GO length: 1 P-Value Cutoff: 0.01 GO-Cluster Cutoff: 0 Correct-Method: Yekutieli |

|  |  |  |  |  |  |
| --- | --- | --- | --- | --- | --- |
| **P-value cutoff:**  1e-101e-50.00010.0010.010.10.30.51 | **Show best:**  110305010010000 | **Indication:**  Over- and UnderrepresentedShow Overrepresented onlyShow Underrepresented only | **Cluster GOs:**  -101234510 | **Display:**  HTML, Stats and Go AnnotationHTML, GO Stats onlyHTML, GO Annotation onlyTEXT, Stats and Go AnnotationTEXT, GO Stats onlyTEXT, GO Annotation only |  |

---

|  |  |  |  |  |  |  |  |  |  |  |  |  |  |  |  |  |  |  |  |  |  |  |  |  |  |  |  |  |  |  |  |  |  |  |  |  |  |  |  |  |  |  |  |  |  |  |  |  |  |  |  |  |  |  |  |  |  |  |  |  |  |  |  |  |  |  |  |  |  |  |  |  |  |  |  |  |  |  |  |  |  |  |  |  |  |  |  |  |  |  |  |  |  |  |  |  |  |  |  |  |  |  |  |  |  |  |  |  |  |  |  |  |  |  |  |  |  |  |  |  |  |  |  |  |  |  |  |  |  |  |  |  |  |  |  |  |  |  |  |  |  |  |  |  |  |  |  |  |  |  |  |  |  |  |  |  |  |  |  |  |  |  |  |  |  |  |  |  |  |  |  |  |  |  |  |  |  |  |  |  |  |  |  |  |  |  |  |  |  |  |  |  |  |  |  |  |  |  |  |  |  |  |  |  |  |  |  |  |  |  |  |  |  |  |  |  |  |  |  |  |  |  |  |  |  |  |  |  |  |  |  |  |  |  |  |  |  |  |  |  |  |  |  |  |  |  |  |  |  |  |  |  |  |  |  |  |  |  |  |  |  |  |  |  |  |  |  |  |  |  |  |  |  |  |  |  |  |  |  |  |  |  |  |  |  |  |  |  |  |  |  |  |  |  |  |  |  |  |  |  |  |  |  |  |  |  |  |  |  |  |  |  |  |  |  |  |  |  |  |  |  |  |  |  |  |  |  |  |  |  |  |  |  |  |  |  |  |  |  |  |  |  |  |  |  |  |  |  |  |  |  |  |  |  |  |  |  |  |  |  |  |  |  |  |  |  |  |  |  |  |  |  |  |  |  |  |  |  |  |  |  |  |  |  |  |  |  |  |  |  |  |  |  |  |  |  |  |  |  |  |  |  |  |  |  |  |  |  |  |  |  |  |  |  |  |  |  |  |  |  |  |  |  |  |  |  |  |  |  |  |  |  |  |  |  |  |  |  |  |  |  |  |  |  |  |  |  |  |  |  |  |  |  |  |  |  |  |  |  |  |  |  |  |  |  |  |  |  |  |  |  |  |  |  |  |  |  |  |  |  |  |  |  |  |  |  |  |  |  |  |  |  |  |  |  |  |  |  |  |  |  |  |  |  |  |  |  |  |  |  |  |  |  |  |  |  |  |  |  |  |  |  |  |  |  |  |  |  |  |  |  |  |  |  |  |  |  |  |  |  |  |  |  |  |  |  |  |  |  |  |  |  |  |  |  |  |  |  |  |  |  |  |  |  |  |  |  |  |  |  |  |  |  |  |  |  |  |  |  |  |  |  |  |  |  |  |  |  |  |  |  |  |  |  |  |  |  |  |  |  |  |  |  |  |  |  |  |  |  |  |  |  |  |  |  |  |  |  |  |  |  |  |  |  |  |  |  |  |  |  |  |  |  |  |  |  |  |  |  |  |  |  |  |  |  |  |  |  |  |  |  |  |  |  |  |  |  |  |  |  |  |  |  |  |  |  |  |  |  |  |  |  |  |  |  |  |  |  |  |  |  |  |  |  |  |  |  |  |  |  |  |  |  |  |  |  |  |  |  |  |  |  |  |  |  |  |  |  |  |  |  |  |  |  |  |  |  |  |  |  |  |  |  |  |  |  |  |  |  |  |  |  |  |  |  |  |  |  |  |  |  |  |  |  |  |  |  |  |  |  |  |  |  |  |  |  |  |  |  |  |  |  |  |  |  |  |  |  |  |  |  |  |  |  |  |  |  |  |  |  |  |  |  |  |  |  |  |  |  |  |  |  |  |  |  |  |  |  |  |  |  |  |  |  |  |  |  |  |  |  |  |  |  |  |  |  |  |  |  |  |  |  |  |  |  |  |  |  |  |  |  |  |  |  |  |  |  |  |  |  |  |  |  |  |  |  |  |  |  |  |  |  |  |  |  |  |  |  |  |  |  |  |  |  |  |  |  |  |  |  |  |  |  |  |  |  |  |  |  |  |  |  |  |  |  |  |  |  |  |  |  |  |  |  |  |  |  |  |  |  |  |  |  |  |  |  |  |  |  |  |  |  |  |  |  |  |  |  |  |  |  |  |  |  |  |  |  |  |  |  |  |  |  |  |  |  |  |  |  |  |  |  |  |  |  |  |  |  |  |  |  |  |  |  |  |  |  |  |  |  |  |  |  |  |  |  |  |  |  |  |  |  |  |  |  |  |  |  |  |  |  |  |  |  |  |  |  |  |  |  |  |  |  |  |  |  |  |  |  |  |  |  |  |  |  |  |  |  |  |  |  |  |  |  |  |  |  |  |  |  |  |  |  |  |  |  |  |  |  |  |  |  |  |  |  |  |  |  |  |  |  |  |  |  |  |  |  |  |  |  |  |  |  |  |  |  |  |  |  |  |  |  |  |  |  |  |  |  |  |  |  |  |  |  |  |  |  |  |  |  |  |  |  |  |  |  |  |  |  |  |  |  |  |  |  |  |  |  |  |  |  |  |  |  |  |  |  |  |  |  |  |  |  |  |  |  |  |  |  |  |  |  |  |  |  |  |  |  |  |  |  |  |  |  |  |  |  |  |  |  |  |  |  |  |  |  |  |  |  |  |  |  |  |  |  |  |  |  |  |  |  |  |  |  |  |  |  |  |  |  |  |  |  |  |  |  |  |  |  |  |  |  |  |  |  |  |  |  |  |  |  |  |  |  |  |  |  |  |  |  |  |  |  |  |  |  |  |  |  |  |  |  |  |  |  |  |  |  |  |  |  |  |  |  |  |  |  |  |  |  |  |  |  |  |  |  |  |  |  |  |  |  |  |  |  |  |  |  |  |  |  |  |  |  |  |  |  |  |  |  |  |  |  |  |  |  |  |  |  |  |  |  |  |  |  |  |  |  |  |  |  |  |  |  |  |  |  |  |  |  |  |  |  |  |  |  |  |  |  |  |  |  |  |  |  |  |  |  |  |  |  |  |  |  |  |  |  |  |  |  |  |  |  |  |  |  |  |  |  |  |  |  |  |  |  |  |  |  |  |  |  |  |  |  |  |  |  |  |  |  |  |  |  |  |  |  |  |  |  |  |  |  |  |  |  |  |  |  |  |  |  |  |  |  |  |  |  |  |  |  |  |  |  |  |  |  |  |  |  |  |  |  |  |  |  |  |  |  |  |  |  |  |  |  |  |  |  |  |  |  |  |  |  |  |  |  |  |  |  |  |  |  |  |  |  |  |  |  |  |  |  |  |  |  |  |  |  |  |  |  |  |  |  |  |  |  |  |  |  |  |  |  |  |  |  |  |  |  |  |  |  |  |  |  |  |  |  |  |  |  |  |  |  |  |  |  |  |  |  |  |  |  |  |  |  |  |  |  |  |  |  |  |  |  |  |  |  |  |  |  |  |  |  |  |  |  |  |  |  |  |  |  |  |  |  |  |  |  |  |  |  |  |  |  |  |  |  |  |  |  |  |  |  |  |  |  |  |  |  |  |  |  |  |  |  |  |  |  |  |  |  |  |  |  |  |  |  |  |  |  |  |  |  |  |  |  |  |  |  |  |  |  |  |  |  |  |  |  |  |  |  |  |  |  |  |  |  |  |  |  |  |  |  |  |  |  |  |  |  |  |  |  |  |  |  |  |  |  |  |  |  |  |  |  |  |  |  |  |  |  |  |  |  |  |  |  |  |  |  |  |  |  |  |  |  |  |  |  |  |  |  |  |  |  |  |  |  |  |  |  |  |  |  |  |  |  |  |  |  |  |  |  |  |  |  |  |  |  |  |  |  |  |  |  |  |  |  |  |  |  |  |  |  |  |  |  |  |  |  |  |  |  |  |  |  |  |  |  |  |  |  |  |  |  |  |  |  |  |  |  |  |  |  |  |  |  |  |  |  |  |  |  |  |  |  |  |  |  |  |  |  |  |  |  |  |  |  |  |  |  |  |  |  |  |  |  |  |  |  |  |  |  |  |  |  |  |  |  |  |  |  |  |  |  |  |  |  |  |  |  |  |  |  |  |  |  |  |  |  |  |  |  |  |  |  |  |  |  |  |  |  |  |  |  |  |  |  |  |  |  |  |  |  |  |  |  |  |  |  |  |  |  |  |  |  |  |  |  |  |  |  |  |  |  |  |  |  |  |  |  |  |  |  |  |  |  |  |  |  |  |  |  |  |  |  |  |  |  |  |  |  |  |  |  |  |  |  |  |  |  |  |  |  |  |  |  |  |  |  |  |  |  |  |  |  |  |  |  |  |  |  |  |  |  |  |  |  |  |  |  |  |  |  |  |  |  |  |  |  |  |  |  |  |  |  |  |  |  |  |  |  |  |  |  |  |  |  |  |  |  |  |  |  |  |  |  |  |  |  |  |  |  |  |  |  |  |  |  |  |  |  |  |  |  |  |  |  |  |  |  |  |  |  |  |  |  |  |  |  |  |  |  |  |  |  |  |  |  |  |  |  |  |  |  |  |  |  |  |  |  |  |  |  |  |  |  |  |  |  |  |  |  |  |  |  |  |  |  |  |  |  |  |  |  |  |  |  |  |  |  |  |  |  |  |  |  |  |  |  |  |  |  |  |  |  |  |  |  |  |  |  |  |  |  |  |  |  |  |  |  |  |  |  |  |  |  |  |  |  |  |  |  |  |  |  |  |  |  |  |  |  |  |  |  |  |  |  |  |  |  |  |  |  |  |  |  |  |  |  |  |  |  |  |  |  |  |  |  |  |  |  |  |  |  |  |  |  |  |  |  |  |  |  |  |  |  |  |  |  |  |  |  |  |  |  |  |  |  |  |  |  |  |  |  |  |  |  |  |  |  |  |  |  |  |  |  |  |  |  |  |  |  |  |  |  |  |  |  |  |  |  |  |  |  |  |  |  |  |  |  |  |  |  |  |  |  |  |  |  |  |  |  |  |  |  |  |  |  |  |  |  |  |  |  |  |  |  |  |  |  |  |  |  |  |  |  |  |  |  |  |  |  |  |  |  |  |  |  |  |  |  |  |  |  |  |  |  |  |  |  |  |  |  |  |  |  |  |  |  |  |  |  |  |  |  |  |  |  |  |  |  |  |  |  |  |  |  |  |  |  |  |  |  |  |  |  |  |  |  |  |  |  |  |  |  |  |  |  |  |  |  |  |  |  |  |  |  |  |  |  |  |  |  |  |  |  |  |  |  |  |  |  |  |  |  |  |  |  |  |  |  |  |  |  |  |  |  |  |  |  |  |  |  |  |  |  |  |  |  |  |  |  |  |  |  |  |  |  |  |  |  |  |  |  |  |  |  |  |  |  |  |  |  |  |  |  |  |  |  |  |  |  |  |  |  |  |  |  |  |  |  |  |  |  |  |  |  |  |  |  |  |  |  |  |  |  |  |  |  |  |  |  |  |  |  |  |  |  |  |  |  |  |  |  |  |  |  |  |  |  |  |  |  |  |  |  |  |  |  |  |  |  |  |  |  |  |  |  |  |  |  |  |  |  |  |  |  |  |  |  |  |  |  |  |  |  |  |  |  |  |  |  |  |  |  |  |  |  |  |  |  |  |  |  |  |  |  |  |  |  |  |  |  |  |  |  |  |  |  |  |  |  |  |  |  |  |  |  |  |  |  |  |  |  |  |  |  |  |  |  |  |  |  |  |  |  |  |  |  |  |  |  |  |  |  |  |  |  |  |  |  |  |  |  |  |  |  |  |  |  |  |  |  |  |  |  |  |  |  |  |  |  |  |  |  |  |  |  |  |  |  |  |  |  |  |  |  |  |  |  |  |  |  |  |  |  |  |  |  |  |  |  |  |  |  |  |  |  |  |  |  |  |  |  |  |  |  |  |  |  |  |  |  |  |  |  |  |  |  |  |  |  |  |  |  |  |  |  |  |  |  |  |  |  |  |  |  |  |  |  |  |  |  |  |  |  |  |  |  |  |  |  |  |  |  |  |  |  |  |  |  |  |  |  |  |  |  |  |  |  |  |  |  |  |  |  |  |  |  |  |  |  |  |  |  |  |  |  |  |  |  |  |  |  |  |  |  |  |  |  |  |  |  |  |  |  |  |  |  |  |  |  |  |  |  |  |  |  |  |  |  |  |  |  |  |  |  |  |  |  |  |  |  |  |  |  |  |  |  |  |  |  |  |  |  |  |  |  |  |  |  |  |  |  |  |  |  |  |  |  |  |  |  |  |  |  |  |  |  |  |  |  |  |  |  |  |  |  |  |  |  |  |  |  |  |  |  |  |  |  |  |  |  |  |  |  |  |  |  |  |  |  |  |  |  |  |  |  |  |  |  |  |  |  |  |  |  |  |  |  |  |  |  |  |  |  |  |  |  |  |  |  |  |  |  |  |  |  |  |  |  |  |  |  |  |  |  |  |  |  |  |  |  |  |  |  |  |  |  |  |  |  |  |  |  |  |  |  |  |  |  |  |  |  |  |  |  |  |  |  |  |  |  |  |  |  |  |  |  |  |  |  |  |  |  |  |  |  |  |  |  |  |  |  |  |  |  |  |  |  |  |  |  |  |  |  |  |  |  |  |  |  |  |  |  |  |  |  |  |  |  |  |  |  |  |  |  |  |  |  |  |  |  |  |  |  |  |  |  |  |  |  |  |  |  |  |  |  |  |  |  |  |  |  |  |  |  |  |  |  |  |  |  |  |  |  |  |  |  |  |  |  |  |  |  |  |  |  |  |  |  |  |  |  |  |  |  |  |  |  |  |  |  |  |  |  |  |  |  |  |  |  |  |  |  |  |  |  |  |  |  |  |  |  |  |  |  |  |  |  |  |  |  |  |  |  |  |  |  |  |  |  |  |  |  |  |  |  |  |  |  |  |  |  |  |
| --- | --- | --- | --- | --- | --- | --- | --- | --- | --- | --- | --- | --- | --- | --- | --- | --- | --- | --- | --- | --- | --- | --- | --- | --- | --- | --- | --- | --- | --- | --- | --- | --- | --- | --- | --- | --- | --- | --- | --- | --- | --- | --- | --- | --- | --- | --- | --- | --- | --- | --- | --- | --- | --- | --- | --- | --- | --- | --- | --- | --- | --- | --- | --- | --- | --- | --- | --- | --- | --- | --- | --- | --- | --- | --- | --- | --- | --- | --- | --- | --- | --- | --- | --- | --- | --- | --- | --- | --- | --- | --- | --- | --- | --- | --- | --- | --- | --- | --- | --- | --- | --- | --- | --- | --- | --- | --- | --- | --- | --- | --- | --- | --- | --- | --- | --- | --- | --- | --- | --- | --- | --- | --- | --- | --- | --- | --- | --- | --- | --- | --- | --- | --- | --- | --- | --- | --- | --- | --- | --- | --- | --- | --- | --- | --- | --- | --- | --- | --- | --- | --- | --- | --- | --- | --- | --- | --- | --- | --- | --- | --- | --- | --- | --- | --- | --- | --- | --- | --- | --- | --- | --- | --- | --- | --- | --- | --- | --- | --- | --- | --- | --- | --- | --- | --- | --- | --- | --- | --- | --- | --- | --- | --- | --- | --- | --- | --- | --- | --- | --- | --- | --- | --- | --- | --- | --- | --- | --- | --- | --- | --- | --- | --- | --- | --- | --- | --- | --- | --- | --- | --- | --- | --- | --- | --- | --- | --- | --- | --- | --- | --- | --- | --- | --- | --- | --- | --- | --- | --- | --- | --- | --- | --- | --- | --- | --- | --- | --- | --- | --- | --- | --- | --- | --- | --- | --- | --- | --- | --- | --- | --- | --- | --- | --- | --- | --- | --- | --- | --- | --- | --- | --- | --- | --- | --- | --- | --- | --- | --- | --- | --- | --- | --- | --- | --- | --- | --- | --- | --- | --- | --- | --- | --- | --- | --- | --- | --- | --- | --- | --- | --- | --- | --- | --- | --- | --- | --- | --- | --- | --- | --- | --- | --- | --- | --- | --- | --- | --- | --- | --- | --- | --- | --- | --- | --- | --- | --- | --- | --- | --- | --- | --- | --- | --- | --- | --- | --- | --- | --- | --- | --- | --- | --- | --- | --- | --- | --- | --- | --- | --- | --- | --- | --- | --- | --- | --- | --- | --- | --- | --- | --- | --- | --- | --- | --- | --- | --- | --- | --- | --- | --- | --- | --- | --- | --- | --- | --- | --- | --- | --- | --- | --- | --- | --- | --- | --- | --- | --- | --- | --- | --- | --- | --- | --- | --- | --- | --- | --- | --- | --- | --- | --- | --- | --- | --- | --- | --- | --- | --- | --- | --- | --- | --- | --- | --- | --- | --- | --- | --- | --- | --- | --- | --- | --- | --- | --- | --- | --- | --- | --- | --- | --- | --- | --- | --- | --- | --- | --- | --- | --- | --- | --- | --- | --- | --- | --- | --- | --- | --- | --- | --- | --- | --- | --- | --- | --- | --- | --- | --- | --- | --- | --- | --- | --- | --- | --- | --- | --- | --- | --- | --- | --- | --- | --- | --- | --- | --- | --- | --- | --- | --- | --- | --- | --- | --- | --- | --- | --- | --- | --- | --- | --- | --- | --- | --- | --- | --- | --- | --- | --- | --- | --- | --- | --- | --- | --- | --- | --- | --- | --- | --- | --- | --- | --- | --- | --- | --- | --- | --- | --- | --- | --- | --- | --- | --- | --- | --- | --- | --- | --- | --- | --- | --- | --- | --- | --- | --- | --- | --- | --- | --- | --- | --- | --- | --- | --- | --- | --- | --- | --- | --- | --- | --- | --- | --- | --- | --- | --- | --- | --- | --- | --- | --- | --- | --- | --- | --- | --- | --- | --- | --- | --- | --- | --- | --- | --- | --- | --- | --- | --- | --- | --- | --- | --- | --- | --- | --- | --- | --- | --- | --- | --- | --- | --- | --- | --- | --- | --- | --- | --- | --- | --- | --- | --- | --- | --- | --- | --- | --- | --- | --- | --- | --- | --- | --- | --- | --- | --- | --- | --- | --- | --- | --- | --- | --- | --- | --- | --- | --- | --- | --- | --- | --- | --- | --- | --- | --- | --- | --- | --- | --- | --- | --- | --- | --- | --- | --- | --- | --- | --- | --- | --- | --- | --- | --- | --- | --- | --- | --- | --- | --- | --- | --- | --- | --- | --- | --- | --- | --- | --- | --- | --- | --- | --- | --- | --- | --- | --- | --- | --- | --- | --- | --- | --- | --- | --- | --- | --- | --- | --- | --- | --- | --- | --- | --- | --- | --- | --- | --- | --- | --- | --- | --- | --- | --- | --- | --- | --- | --- | --- | --- | --- | --- | --- | --- | --- | --- | --- | --- | --- | --- | --- | --- | --- | --- | --- | --- | --- | --- | --- | --- | --- | --- | --- | --- | --- | --- | --- | --- | --- | --- | --- | --- | --- | --- | --- | --- | --- | --- | --- | --- | --- | --- | --- | --- | --- | --- | --- | --- | --- | --- | --- | --- | --- | --- | --- | --- | --- | --- | --- | --- | --- | --- | --- | --- | --- | --- | --- | --- | --- | --- | --- | --- | --- | --- | --- | --- | --- | --- | --- | --- | --- | --- | --- | --- | --- | --- | --- | --- | --- | --- | --- | --- | --- | --- | --- | --- | --- | --- | --- | --- | --- | --- | --- | --- | --- | --- | --- | --- | --- | --- | --- | --- | --- | --- | --- | --- | --- | --- | --- | --- | --- | --- | --- | --- | --- | --- | --- | --- | --- | --- | --- | --- | --- | --- | --- | --- | --- | --- | --- | --- | --- | --- | --- | --- | --- | --- | --- | --- | --- | --- | --- | --- | --- | --- | --- | --- | --- | --- | --- | --- | --- | --- | --- | --- | --- | --- | --- | --- | --- | --- | --- | --- | --- | --- | --- | --- | --- | --- | --- | --- | --- | --- | --- | --- | --- | --- | --- | --- | --- | --- | --- | --- | --- | --- | --- | --- | --- | --- | --- | --- | --- | --- | --- | --- | --- | --- | --- | --- | --- | --- | --- | --- | --- | --- | --- | --- | --- | --- | --- | --- | --- | --- | --- | --- | --- | --- | --- | --- | --- | --- | --- | --- | --- | --- | --- | --- | --- | --- | --- | --- | --- | --- | --- | --- | --- | --- | --- | --- | --- | --- | --- | --- | --- | --- | --- | --- | --- | --- | --- | --- | --- | --- | --- | --- | --- | --- | --- | --- | --- | --- | --- | --- | --- | --- | --- | --- | --- | --- | --- | --- | --- | --- | --- | --- | --- | --- | --- | --- | --- | --- | --- | --- | --- | --- | --- | --- | --- | --- | --- | --- | --- | --- | --- | --- | --- | --- | --- | --- | --- | --- | --- | --- | --- | --- | --- | --- | --- | --- | --- | --- | --- | --- | --- | --- | --- | --- | --- | --- | --- | --- | --- | --- | --- | --- | --- | --- | --- | --- | --- | --- | --- | --- | --- | --- | --- | --- | --- | --- | --- | --- | --- | --- | --- | --- | --- | --- | --- | --- | --- | --- | --- | --- | --- | --- | --- | --- | --- | --- | --- | --- | --- | --- | --- | --- | --- | --- | --- | --- | --- | --- | --- | --- | --- | --- | --- | --- | --- | --- | --- | --- | --- | --- | --- | --- | --- | --- | --- | --- | --- | --- | --- | --- | --- | --- | --- | --- | --- | --- | --- | --- | --- | --- | --- | --- | --- | --- | --- | --- | --- | --- | --- | --- | --- | --- | --- | --- | --- | --- | --- | --- | --- | --- | --- | --- | --- | --- | --- | --- | --- | --- | --- | --- | --- | --- | --- | --- | --- | --- | --- | --- | --- | --- | --- | --- | --- | --- | --- | --- | --- | --- | --- | --- | --- | --- | --- | --- | --- | --- | --- | --- | --- | --- | --- | --- | --- | --- | --- | --- | --- | --- | --- | --- | --- | --- | --- | --- | --- | --- | --- | --- | --- | --- | --- | --- | --- | --- | --- | --- | --- | --- | --- | --- | --- | --- | --- | --- | --- | --- | --- | --- | --- | --- | --- | --- | --- | --- | --- | --- | --- | --- | --- | --- | --- | --- | --- | --- | --- | --- | --- | --- | --- | --- | --- | --- | --- | --- | --- | --- | --- | --- | --- | --- | --- | --- | --- | --- | --- | --- | --- | --- | --- | --- | --- | --- | --- | --- | --- | --- | --- | --- | --- | --- | --- | --- | --- | --- | --- | --- | --- | --- | --- | --- | --- | --- | --- | --- | --- | --- | --- | --- | --- | --- | --- | --- | --- | --- | --- | --- | --- | --- | --- | --- | --- | --- | --- | --- | --- | --- | --- | --- | --- | --- | --- | --- | --- | --- | --- | --- | --- | --- | --- | --- | --- | --- | --- | --- | --- | --- | --- | --- | --- | --- | --- | --- | --- | --- | --- | --- | --- | --- | --- | --- | --- | --- | --- | --- | --- | --- | --- | --- | --- | --- | --- | --- | --- | --- | --- | --- | --- | --- | --- | --- | --- | --- | --- | --- | --- | --- | --- | --- | --- | --- | --- | --- | --- | --- | --- | --- | --- | --- | --- | --- | --- | --- | --- | --- | --- | --- | --- | --- | --- | --- | --- | --- | --- | --- | --- | --- | --- | --- | --- | --- | --- | --- | --- | --- | --- | --- | --- | --- | --- | --- | --- | --- | --- | --- | --- | --- | --- | --- | --- | --- | --- | --- | --- | --- | --- | --- | --- | --- | --- | --- | --- | --- | --- | --- | --- | --- | --- | --- | --- | --- | --- | --- | --- | --- | --- | --- | --- | --- | --- | --- | --- | --- | --- | --- | --- | --- | --- | --- | --- | --- | --- | --- | --- | --- | --- | --- | --- | --- | --- | --- | --- | --- | --- | --- | --- | --- | --- | --- | --- | --- | --- | --- | --- | --- | --- | --- | --- | --- | --- | --- | --- | --- | --- | --- | --- | --- | --- | --- | --- | --- | --- | --- | --- | --- | --- | --- | --- | --- | --- | --- | --- | --- | --- | --- | --- | --- | --- | --- | --- | --- | --- | --- | --- | --- | --- | --- | --- | --- | --- | --- | --- | --- | --- | --- | --- | --- | --- | --- | --- | --- | --- | --- | --- | --- | --- | --- | --- | --- | --- | --- | --- | --- | --- | --- | --- | --- | --- | --- | --- | --- | --- | --- | --- | --- | --- | --- | --- | --- | --- | --- | --- | --- | --- | --- | --- | --- | --- | --- | --- | --- | --- | --- | --- | --- | --- | --- | --- | --- | --- | --- | --- | --- | --- | --- | --- | --- | --- | --- | --- | --- | --- | --- | --- | --- | --- | --- | --- | --- | --- | --- | --- | --- | --- | --- | --- | --- | --- | --- | --- | --- | --- | --- | --- | --- | --- | --- | --- | --- | --- | --- | --- | --- | --- | --- | --- | --- | --- | --- | --- | --- | --- | --- | --- | --- | --- | --- | --- | --- | --- | --- | --- | --- | --- | --- | --- | --- | --- | --- | --- | --- | --- | --- | --- | --- | --- | --- | --- | --- | --- | --- | --- | --- | --- | --- | --- | --- | --- | --- | --- | --- | --- | --- | --- | --- | --- | --- | --- | --- | --- | --- | --- | --- | --- | --- | --- | --- | --- | --- | --- | --- | --- | --- | --- | --- | --- | --- | --- | --- | --- | --- | --- | --- | --- | --- | --- | --- | --- | --- | --- | --- | --- | --- | --- | --- | --- | --- | --- | --- | --- | --- | --- | --- | --- | --- | --- | --- | --- | --- | --- | --- | --- | --- | --- | --- | --- | --- | --- | --- | --- | --- | --- | --- | --- | --- | --- | --- | --- | --- | --- | --- | --- | --- | --- | --- | --- | --- | --- | --- | --- | --- | --- | --- | --- | --- | --- | --- | --- | --- | --- | --- | --- | --- | --- | --- | --- | --- | --- | --- | --- | --- | --- | --- | --- | --- | --- | --- | --- | --- | --- | --- | --- | --- | --- | --- | --- | --- | --- | --- | --- | --- | --- | --- | --- | --- | --- | --- | --- | --- | --- | --- | --- | --- | --- | --- | --- | --- | --- | --- | --- | --- | --- | --- | --- | --- | --- | --- | --- | --- | --- | --- | --- | --- | --- | --- | --- | --- | --- | --- | --- | --- | --- | --- | --- | --- | --- | --- | --- | --- | --- | --- | --- | --- | --- | --- | --- | --- | --- | --- | --- | --- | --- | --- | --- | --- | --- | --- | --- | --- | --- | --- | --- | --- | --- | --- | --- | --- | --- | --- | --- | --- | --- | --- | --- | --- | --- | --- | --- | --- | --- | --- | --- | --- | --- | --- | --- | --- | --- | --- | --- | --- | --- | --- | --- | --- | --- | --- | --- | --- | --- | --- | --- | --- | --- | --- | --- | --- | --- | --- | --- | --- | --- | --- | --- | --- | --- | --- | --- | --- | --- | --- | --- | --- | --- | --- | --- | --- | --- | --- | --- | --- | --- | --- | --- | --- | --- | --- | --- | --- | --- | --- | --- | --- | --- | --- | --- | --- | --- | --- | --- | --- | --- | --- | --- | --- | --- | --- | --- | --- | --- | --- | --- | --- | --- | --- | --- | --- | --- | --- | --- | --- | --- | --- | --- | --- | --- | --- | --- | --- | --- | --- | --- | --- | --- | --- | --- | --- | --- | --- | --- | --- | --- | --- | --- | --- | --- | --- | --- | --- | --- | --- | --- | --- | --- | --- | --- | --- | --- | --- | --- | --- | --- | --- | --- | --- | --- | --- | --- | --- | --- | --- | --- | --- | --- | --- | --- | --- | --- | --- | --- | --- | --- | --- | --- | --- | --- | --- | --- | --- | --- | --- | --- | --- | --- | --- | --- | --- | --- | --- | --- | --- | --- | --- | --- | --- | --- | --- | --- | --- | --- | --- | --- | --- | --- | --- | --- | --- | --- | --- | --- | --- | --- | --- | --- | --- | --- | --- | --- | --- | --- | --- | --- | --- | --- | --- | --- | --- | --- | --- | --- | --- | --- | --- | --- | --- | --- | --- | --- | --- | --- | --- | --- | --- | --- | --- | --- | --- | --- | --- | --- | --- | --- | --- | --- | --- | --- | --- | --- | --- | --- | --- | --- | --- | --- | --- | --- | --- | --- | --- | --- | --- | --- | --- | --- | --- | --- | --- | --- | --- | --- | --- | --- | --- | --- | --- | --- | --- | --- | --- | --- | --- | --- | --- | --- | --- | --- | --- | --- | --- | --- | --- | --- | --- | --- | --- | --- | --- | --- | --- | --- | --- | --- | --- | --- | --- | --- | --- | --- | --- | --- | --- | --- | --- | --- | --- | --- | --- | --- | --- | --- | --- | --- | --- | --- | --- | --- | --- | --- | --- | --- | --- | --- | --- | --- | --- | --- | --- | --- | --- | --- | --- | --- | --- | --- | --- | --- | --- | --- | --- | --- | --- | --- | --- | --- | --- | --- | --- | --- | --- | --- | --- | --- | --- | --- | --- | --- | --- | --- | --- | --- | --- | --- | --- | --- | --- | --- | --- | --- | --- | --- | --- | --- | --- | --- | --- | --- | --- | --- | --- | --- | --- | --- | --- | --- | --- | --- | --- | --- | --- | --- | --- | --- | --- | --- | --- | --- | --- | --- | --- | --- | --- | --- | --- | --- | --- | --- | --- | --- | --- | --- | --- | --- | --- | --- | --- | --- | --- | --- | --- | --- | --- | --- | --- | --- | --- | --- | --- | --- | --- | --- | --- | --- | --- | --- | --- | --- | --- | --- | --- | --- | --- | --- | --- | --- | --- | --- | --- | --- | --- | --- | --- | --- | --- | --- | --- | --- | --- | --- | --- | --- | --- | --- | --- | --- | --- | --- | --- | --- | --- | --- | --- | --- | --- | --- | --- | --- | --- | --- | --- | --- | --- | --- | --- | --- | --- | --- | --- | --- | --- | --- | --- | --- | --- | --- | --- | --- | --- | --- | --- | --- | --- | --- | --- | --- | --- | --- | --- | --- | --- | --- | --- | --- | --- | --- | --- | --- | --- | --- | --- | --- | --- | --- | --- | --- | --- | --- | --- | --- | --- | --- | --- | --- | --- | --- | --- | --- | --- | --- | --- | --- | --- | --- | --- | --- | --- | --- | --- | --- | --- | --- | --- | --- | --- | --- | --- | --- | --- | --- | --- | --- | --- | --- | --- | --- | --- | --- | --- | --- | --- | --- | --- | --- | --- | --- | --- | --- | --- | --- | --- | --- | --- | --- | --- | --- | --- | --- | --- | --- | --- | --- | --- | --- | --- | --- | --- | --- | --- | --- | --- | --- | --- | --- | --- | --- | --- | --- | --- | --- | --- | --- | --- | --- | --- | --- | --- | --- | --- | --- | --- | --- | --- | --- | --- | --- | --- | --- | --- | --- | --- | --- | --- | --- | --- | --- | --- | --- | --- | --- | --- | --- | --- | --- | --- | --- | --- | --- | --- | --- | --- | --- | --- | --- | --- | --- | --- | --- | --- | --- | --- | --- | --- | --- | --- | --- | --- | --- | --- | --- | --- | --- | --- | --- | --- | --- | --- | --- | --- | --- | --- | --- | --- | --- | --- | --- | --- | --- | --- | --- | --- | --- | --- | --- | --- | --- | --- | --- | --- | --- | --- | --- | --- | --- | --- | --- | --- | --- | --- | --- | --- | --- | --- | --- | --- | --- | --- | --- | --- | --- | --- | --- | --- | --- | --- | --- | --- | --- | --- | --- | --- | --- | --- | --- | --- | --- | --- | --- | --- | --- | --- | --- | --- | --- | --- | --- | --- | --- | --- | --- | --- | --- | --- | --- | --- | --- | --- | --- | --- | --- | --- | --- | --- | --- | --- | --- | --- | --- | --- | --- | --- | --- | --- | --- | --- | --- | --- | --- | --- | --- | --- | --- | --- | --- | --- | --- | --- | --- | --- | --- | --- | --- | --- | --- | --- | --- | --- | --- | --- | --- | --- | --- | --- | --- | --- | --- | --- | --- | --- | --- | --- | --- | --- | --- | --- | --- | --- | --- | --- | --- | --- | --- | --- | --- | --- | --- | --- | --- | --- | --- | --- | --- | --- | --- | --- | --- | --- | --- | --- | --- | --- | --- | --- | --- | --- | --- | --- | --- | --- | --- | --- | --- | --- | --- | --- | --- | --- | --- | --- | --- | --- | --- | --- | --- | --- | --- | --- | --- | --- | --- | --- | --- | --- | --- | --- | --- | --- | --- | --- | --- | --- | --- | --- | --- | --- | --- | --- | --- | --- | --- | --- | --- | --- | --- | --- | --- | --- | --- | --- | --- | --- | --- | --- | --- | --- | --- | --- | --- | --- | --- | --- | --- | --- | --- | --- | --- | --- | --- | --- | --- | --- | --- | --- | --- | --- | --- | --- | --- | --- | --- | --- | --- | --- | --- | --- | --- | --- | --- | --- | --- | --- | --- | --- | --- | --- | --- | --- | --- | --- | --- | --- | --- | --- | --- | --- | --- | --- | --- | --- | --- | --- | --- | --- | --- | --- | --- | --- | --- | --- | --- | --- | --- | --- | --- | --- | --- | --- | --- | --- | --- | --- | --- | --- | --- | --- | --- | --- | --- | --- | --- | --- | --- | --- | --- | --- | --- | --- | --- | --- | --- | --- | --- | --- | --- | --- | --- | --- | --- | --- | --- | --- | --- | --- | --- | --- | --- | --- | --- | --- | --- | --- | --- | --- | --- | --- | --- | --- | --- | --- | --- | --- | --- | --- | --- | --- | --- | --- | --- | --- | --- | --- | --- | --- | --- | --- | --- | --- | --- | --- | --- | --- | --- | --- | --- | --- | --- | --- | --- | --- | --- | --- | --- | --- | --- | --- | --- | --- | --- | --- | --- | --- | --- | --- | --- | --- | --- | --- | --- | --- | --- | --- | --- | --- | --- | --- | --- | --- | --- | --- | --- | --- | --- | --- | --- | --- | --- | --- | --- | --- | --- | --- | --- | --- | --- | --- | --- | --- | --- | --- | --- | --- | --- | --- | --- | --- | --- | --- | --- | --- | --- | --- | --- | --- | --- | --- | --- |
| |  |  |  |  |  | | --- | --- | --- | --- | --- | | **Best GOs** (Max: 10000) | **Genes** | **Count** 626 | **Total** 10875 | **P-Value** | | GO:0043170 GO:0016070 GO:0008152 GO:0044238 GO:0006259 GO:0006396 GO:0006412 GO:0044249 GO:0009058 GO:0006260 GO:0009059 GO:0043283 GO:0006139 GO:0000377 GO:0000398 GO:0000375 GO:0008380 GO:0043037 GO:0006397 GO:0006974 GO:0044267 GO:0044260 GO:0009719 GO:0006457 GO:0006281 GO:0019538 GO:0016071 GO:0006261 GO:0006364 GO:0016072 GO:0006399 GO:0007001 GO:0006119 GO:0006732 GO:0006413 GO:0006270 GO:0007582 GO:0051301 GO:0051641 GO:0016568 | TRIP6 UCHL3 MRPS18A SMNDC1 PPP1CC UBA2 SLC30A9 RPL23 GOT2 ENO1 UBL4A ABCD3 CKS1B MCM3 ACVR2B VPS4A BYSL DUS4L DHCR24 RNASEH2A CCT4 ATP5F1 PDAP1 SNRPE COX5A NDUFA13 CTPS HNRPD APPBP1 NUP155 GTF2F2 TIMM44 KIAA0971 NUTF2 MNAT1 SATB2 FAF1 RNU3IP2 TAF4B PITX1 ALG3 FAU PAFAH1B3 GFPT1 CYCS TPX2 EIF2S3 SLC29A2 VBP1 NSBP1 GLO1 SLC25A13 XAB2 UGCGL2 CD320 MRPS10 NOLA1 PHB2 MRPS35 CSTF1 CBX3 TRIB3 ZNF473 ATP5G2 IMP4 EXOSC4 MRPS33 RPLP0 UBE2M EIF4E YARS TAF6 ARL2 ITGB4BP TAF1A ACP1 ATP5J2 MDN1 COX15 CTNNAL1 PPIH RPL13A C20ORF20 ACN9 C6ORF75 RPL36 ARL1 RPL41 TTLL12 GOT1 DLD UBE2S PAICS RFC2 ILKAP ORC2L DUSP14 RPL36A ORC1L HBS1L HMMR MRPS15 CDK9 YEATS4 C20ORF18 PSMB2 NOC4L RPS7 ERP29 15E1.2 PPP3CB SNRPC SNRPD1 FADS1 DNAJA2 NONO POLR3K ASS FASN PRIM1 SET RFC3 DKFZP779L1558 RDBP CHAF1B RBBP4 MAD2L1BP ILF2 CSTF2 SLC39A14 ACY1 DDX19B RGS16 IARS2 CHST5 AURKAIP1 MGC5297 EXO1 IGBP1 TXNL2 NDUFB7 BLMH ACTL6A CAND1 TIMM9 BMPR1A LIAS ETV4 METTL5 SLC5A6 WBSCR1 LSM2 TFB1M RPL35 DENR ZNHIT3 SRM SNRPD3 PHGDH MCFD2 PSMB7 SARS2 BXDC2 RUVBL1 MAD1L1 PPM1G PRSS15 PDCD5 E2F6 GPS1 CDK5RAP1 NDUFA9 GMNN NHP2L1 HADHSC ZNF593 ALDOC PHOSPHO2 CCT5 NUP93 TBRG4 SUV39H1 SLC25A12 TIMM10 HDAC1 C15ORF15 COPE PLK1 RAD51C EIF2S2 DAP3 NUP133 PRPS1 MIF ICMT COX7B DDX47 MRPL42 MDH1 MRPL19 STIL UCRC SLC25A4 RAD23B FRG1 BBS7 ST13 SLC27A2 MRPL16 KDELR1 NUP205 AOF2 WARS2 PPIE GTF3C3 UPF3B PSMC3 MRPL35 TIMM17B EIF3S2 RNF8 AP3M2 TNPO3 TPI1 GLRX2 SLC25A1 EZH2 ZNF282 PSIP1 SLC25A17 POLR2F BNIP1 SLC25A5 GLMN POLE2 PCNP MCM7 POP7 ATP5G3 USP21 RPL31 HMGCS1 AFG3L2 PSMB3 MRPL39 DCLRE1A GTF2H4 KDELR2 SMS HSPD1 TLK2 GPX4 MRPL23 NDUFV1 PRKCI HOMER1 PSAT1 PRPF19 MRPL12 WDR57 MTIF2 RPS28 NDUFS8 TDG TEX10 STX18 ADH5 ICT1 DEK EIF2B3 RPA2 CEBPZ FANCE MASA GMPS ABCF1 APH1A CDT1 TUBB MUT ANAPC1 CSNK2A2 SMARCB1 RP4-691N24.2 PUS7 DIABLO ASCC1 MELK SNRPG RABGGTB ZZZ3 GPR68 MEIS2 COQ3 HNRPC DLAT TRIT1 PRAF1 CCNB1IP1 MPHOSPH6 PPP1R8 NDUFA10 GEMIN6 SC4MOL MRPS17 CDC20 NUP153 ACAT2 NUP37 HMG20A NKRF LARS2 LIN7C NDUFB6 SF3B2 RPL28 COX8A FANCL ATP5B UTP14A SMAD4 MDH2 IARS MRPL48 ERH RPL18A TBP TCERG1 MPP6 MRPL2 DNAJC12 COG5 POLR2I SMARCA3 THOP1 CSPG6 TOP2A UQCRC1 RBBP8 UBA52 DPAGT1 MTX2 TUFM MRPL11 ATP5J SF3B5 DDX39 HMGA1 CAPN7 MRPL34 HSF2 C1ORF33 ZNF24 MYC RPS27A UBE2L3 BCOR LDHB RFC1 MOCS2 H2AFZ TOMM22 ATIC RFC4 BAG2 ARD1A GOSR1 TTK CLPP PRKD3 NCBP1 RUVBL2 KNTC2 COX7A2 DRG1 CS HDAC2 DAXX G10 USP39 ENDOG NME1 SART1 PRDX4 ORC3L CCT3 GTF3C2 SLC3A2 ABT1 MRPL49 PBK RPL26L1 SNRPF CSNK2A1 EXOSC5 HSPE1 RFC5 TWIST1 DDX56 EIF3S4 EXOSC8 EIF4EBP1 UTP11L HIRA CDK7 ISOC2 SORD PMPCB SEH1L COX10 SUCLA2 DNAJA3 WDHD1 DHX16 IDH3B CHEK2 LSM8 NDUFV2 POLA2 VARS CBR4 PSMA5 CDC45L DCTN3 WHSC2 DBN1 SLC1A5 SLC16A1 RFXANK SHFM1 TRIP13 KIF18A MYBL2 TCEA1 SRP9 AARS PTTG1 SLC27A5 CYP51A1 HOXD13 CLPX HSPA4L TRAP1 SHMT2 ANAPC10 UQCRH RPP30 AHCY CDC7 PWP1 RAN VDAC2 CDC2 FKBP4 MLC1SA ADSL NDUFA7 NSDHL METAP1 MSH2 GARS SLC3A1 WEE1 NR2F2 PDHX CRY1 EXOSC9 PSMC6 SLC19A2 BET1 GNL2 FLJ21839 MRPL22 NUP107 CDC37 PES1 MAD2L1 BRRN1 SSR3 TGDS CCNH ITGB3BP TIMM8A ODC1 NEDD8 RPA3 PSMF1 RAC3 MRPS2 PSPH EFTUD1 POLR3B FLJ10292 RPL39L RABEPK POT1 COX6A1 MYB CDC25A SNRPD2 CDCA8 ARNTL2 | 287 61 415 384 69 51 65 96 103 29 68 183 209 24 24 24 26 25 28 30 165 166 30 25 26 175 28 16 14 14 14 27 13 18 11 7 502 18 47 16 | 3081 383 5403 4939 481 313 451 799 897 140 511 1974 2361 114 114 114 134 132 176 203 1984 2025 214 164 176 2186 197 71 59 62 66 214 62 122 46 19 7960 124 467 106 | 1.47e-19 2.15e-14 2.89e-14 2.88e-13 3.17e-13 1.15e-12 1.42e-12 4.12e-12 1.64e-11 4.68e-11 6.62e-11 8.66e-11 1.8e-10 2.63e-09 2.63e-09 2.63e-09 1.06e-08 6.65e-08 4.09e-06 1.57e-05 2.08e-05 4.9e-05 7.19e-05 7.19e-05 9.98e-05 0.000105 0.000109 0.000334 0.000703 0.00123 0.00232 0.00322 0.00482 0.00489 0.00506 0.00593 0.00598 0.00605 0.00689 0.00812 | | GO:0044237 GO:0050875 GO:0042254 GO:0007046 GO:0007049 GO:0016043 GO:0000087 GO:0000278 GO:0006996 GO:0006605 GO:0007067 GO:0051276 GO:0000279 GO:0000074 GO:0051726 GO:0006913 GO:0046907 GO:0051649 GO:0006626 | TRIP6 UCHL3 MRPS18A SMNDC1 PPP1CC UBA2 SLC30A9 RPL23 GOT2 ENO1 UBL4A ABCD3 CKS1B MCM3 ACVR2B VPS4A DUS4L DHCR24 CCT4 RNASEH2A ATP5F1 PDAP1 SNRPE COX5A NDUFA13 CTPS APPBP1 HNRPD NUP155 GTF2F2 TIMM44 KIAA0971 NUTF2 MNAT1 FAF1 SATB2 RNU3IP2 PITX1 FAU TAF4B ALG3 GFPT1 CYCS TPX2 EIF2S3 SLC29A2 VBP1 GLO1 NSBP1 SLC25A13 XAB2 UGCGL2 CD320 MRPS10 NOLA1 PHB2 MRPS35 CSTF1 CBX3 TRIB3 ZNF473 ATP5G2 IMP4 EXOSC4 MRPS33 RPLP0 UBE2M YARS EIF4E TAF6 ITGB4BP ARL2 ACP1 TAF1A ATP5J2 MDN1 CTNNAL1 PPIH C20ORF20 RPL13A ACN9 C6ORF75 RPL36 ARL1 RPL41 TTLL12 GOT1 UBE2S DLD RFC2 PAICS ILKAP ORC2L DUSP14 RPL36A ORC1L HBS1L HMMR MRPS15 CDK9 YEATS4 C20ORF18 PSMB2 NOC4L RPS7 ERP29 15E1.2 SNRPC PPP3CB SNRPD1 FADS1 DNAJA2 NONO POLR3K ASS FASN PRIM1 RFC3 SET DKFZP779L1558 RDBP RBBP4 CHAF1B MAD2L1BP ILF2 CSTF2 ACY1 SLC39A14 DDX19B CHST5 IARS2 AURKAIP1 MGC5297 EXO1 TXNL2 BLMH NDUFB7 ACTL6A CAND1 TIMM9 BMPR1A METTL5 ETV4 LIAS SLC5A6 WBSCR1 TFB1M LSM2 RPL35 DENR ZNHIT3 SNRPD3 SRM PHGDH MCFD2 PSMB7 SARS2 BXDC2 RUVBL1 MAD1L1 PPM1G PRSS15 PDCD5 E2F6 GPS1 CDK5RAP1 GMNN NHP2L1 NDUFA9 HADHSC ALDOC ZNF593 CCT5 NUP93 TBRG4 SUV39H1 SLC25A12 TIMM10 HDAC1 C15ORF15 COPE PLK1 RAD51C EIF2S2 DAP3 NUP133 PRPS1 ICMT MIF COX7B DDX47 MRPL42 MDH1 MRPL19 STIL SLC25A4 UCRC RAD23B FRG1 ST13 SLC27A2 MRPL16 KDELR1 NUP205 AOF2 WARS2 PPIE UPF3B GTF3C3 MRPL35 TIMM17B EIF3S2 RNF8 AP3M2 TNPO3 TPI1 GLRX2 SLC25A1 EZH2 PSIP1 ZNF282 SLC25A17 POLR2F BNIP1 SLC25A5 GLMN POLE2 PCNP POP7 MCM7 USP21 ATP5G3 RPL31 AFG3L2 HMGCS1 PSMB3 DCLRE1A GTF2H4 MRPL39 KDELR2 SMS HSPD1 TLK2 GPX4 NDUFV1 MRPL23 PRKCI PSAT1 MRPL12 WDR57 PRPF19 MTIF2 RPS28 NDUFS8 TDG TEX10 STX18 ADH5 ICT1 EIF2B3 DEK RPA2 CEBPZ FANCE GMPS ABCF1 TUBB APH1A CDT1 SMARCB1 CSNK2A2 ANAPC1 RP4-691N24.2 PUS7 DIABLO MELK ASCC1 RABGGTB SNRPG ZZZ3 MEIS2 HNRPC COQ3 DLAT TRIT1 PRAF1 CCNB1IP1 MPHOSPH6 PPP1R8 NDUFA10 GEMIN6 SC4MOL CDC20 MRPS17 NUP153 NUP37 HMG20A NKRF LARS2 LIN7C NDUFB6 RPL28 SF3B2 COX8A FANCL ATP5B UTP14A SMAD4 MDH2 MRPL48 IARS RPL18A ERH TBP TCERG1 MRPL2 DNAJC12 COG5 POLR2I SMARCA3 THOP1 CSPG6 TOP2A UQCRC1 UBA52 RBBP8 MTX2 TUFM MRPL11 SF3B5 ATP5J DDX39 HMGA1 CAPN7 MRPL34 HSF2 C1ORF33 ZNF24 RPS27A MYC UBE2L3 BCOR LDHB RFC1 MOCS2 H2AFZ TOMM22 ATIC RFC4 BAG2 ARD1A GOSR1 TTK PRKD3 CLPP NCBP1 RUVBL2 KNTC2 DRG1 COX7A2 CS HDAC2 DAXX G10 USP39 ENDOG NME1 SART1 ORC3L PRDX4 CCT3 SLC3A2 GTF3C2 PBK ABT1 MRPL49 RPL26L1 CSNK2A1 SNRPF EXOSC5 HSPE1 RFC5 TWIST1 DDX56 EIF3S4 EXOSC8 EIF4EBP1 UTP11L HIRA CDK7 SORD PMPCB SEH1L COX10 DNAJA3 SUCLA2 DHX16 WDHD1 IDH3B CHEK2 LSM8 POLA2 NDUFV2 VARS CDC45L PSMA5 DCTN3 WHSC2 DBN1 SLC1A5 SLC16A1 RFXANK SHFM1 TRIP13 KIF18A SRP9 TCEA1 MYBL2 AARS PTTG1 CYP51A1 SLC27A5 HOXD13 CLPX HSPA4L TRAP1 SHMT2 ANAPC10 RPP30 UQCRH AHCY CDC7 PWP1 RAN VDAC2 CDC2 FKBP4 MLC1SA ADSL NDUFA7 NSDHL METAP1 MSH2 GARS SLC3A1 WEE1 NR2F2 CRY1 EXOSC9 PSMC6 SLC19A2 BET1 GNL2 FLJ21839 MRPL22 NUP107 CDC37 PES1 MAD2L1 BRRN1 SSR3 TGDS CCNH ITGB3BP TIMM8A ODC1 NEDD8 RPA3 PSMF1 RAC3 MRPS2 PSPH EFTUD1 POLR3B FLJ10292 RPL39L RABEPK POT1 COX6A1 MYB CDC25A SNRPD2 CDCA8 ARNTL2 | 401 484 21 19 62 104 20 25 64 20 19 29 22 43 43 16 47 47 7 | 5054 7169 77 73 584 1171 125 178 643 132 123 229 157 398 399 100 456 462 19 | 5.48e-16 2.33e-07 3.67e-07 4.87e-06 7.19e-05 0.000285 0.000334 0.000601 0.000601 0.00114 0.00125 0.00152 0.00226 0.00228 0.00239 0.00322 0.00382 0.00537 0.00593 | | GO:0007028 | GNL2 C15ORF15 IMP4 EXOSC4 PRKCI RPLP0 EXOSC5 BXDC2 DDX56 RNU3IP2 NHP2L1 EXOSC8 NOC4L ARL2 UTP11L FRG1 NOLA1 EXOSC9 C1ORF33 UTP14A TFB1M ARL1 | 22 | 93 | 2.12e-10 | |  | |  |  |  | | --- | --- | --- | | **Gene** | **Search Term** | **GOs** | | 15E1.2 | 214711\_at | GO:0000004 GO:0006450 | | 200022\_AT | 200022\_at |  | | 200025\_S\_AT | 200025\_s\_at |  | | 200031\_S\_AT | 200031\_s\_at |  | | 200038\_S\_AT | 200038\_s\_at |  | | 200040\_AT | 200040\_at |  | | 200092\_S\_AT | 200092\_s\_at |  | | 200659\_S\_AT | 200659\_s\_at |  | | 200662\_S\_AT | 200662\_s\_at |  | | 200687\_S\_AT | 200687\_s\_at |  | | 200691\_S\_AT | 200691\_s\_at |  | | 200755\_S\_AT | 200755\_s\_at |  | | 200792\_AT | 200792\_at |  | | 200823\_X\_AT | 200823\_x\_at |  | | 200843\_S\_AT | 200843\_s\_at |  | | 200845\_S\_AT | 200845\_s\_at |  | | 200860\_S\_AT | 200860\_s\_at |  | | 200873\_S\_AT | 200873\_s\_at |  | | 200957\_S\_AT | 200957\_s\_at |  | | 200995\_AT | 200995\_at |  | | 201001\_S\_AT | 201001\_s\_at |  | | 201016\_AT | 201016\_at |  | | 201027\_S\_AT | 201027\_s\_at |  | | 201043\_S\_AT | 201043\_s\_at |  | | 201088\_AT | 201088\_at |  | | 201112\_S\_AT | 201112\_s\_at |  | | 201115\_AT | 201115\_at |  | | 201121\_S\_AT | 201121\_s\_at |  | | 201139\_S\_AT | 201139\_s\_at |  | | 201173\_X\_AT | 201173\_x\_at |  | | 201189\_S\_AT | 201189\_s\_at |  | | 201226\_AT | 201226\_at |  | | 201246\_S\_AT | 201246\_s\_at |  | | 201263\_AT | 201263\_at |  | | 201306\_S\_AT | 201306\_s\_at |  | | 201317\_S\_AT | 201317\_s\_at |  | | 201327\_S\_AT | 201327\_s\_at |  | | 201458\_S\_AT | 201458\_s\_at |  | | 201475\_X\_AT | 201475\_x\_at |  | | 201477\_S\_AT | 201477\_s\_at |  | | 201478\_S\_AT | 201478\_s\_at |  | | 201624\_AT | 201624\_at |  | | 201634\_S\_AT | 201634\_s\_at |  | | 201662\_S\_AT | 201662\_s\_at |  | | 201687\_S\_AT | 201687\_s\_at |  | | 201713\_S\_AT | 201713\_s\_at |  | | 201747\_S\_AT | 201747\_s\_at |  | | 201782\_S\_AT | 201782\_s\_at |  | | 201871\_S\_AT | 201871\_s\_at |  | | 201873\_S\_AT | 201873\_s\_at |  | | 201937\_S\_AT | 201937\_s\_at |  | | 201955\_AT | 201955\_at |  | | 201979\_S\_AT | 201979\_s\_at |  | | 202020\_S\_AT | 202020\_s\_at |  | | 202029\_X\_AT | 202029\_x\_at |  | | 202070\_S\_AT | 202070\_s\_at |  | | 202138\_X\_AT | 202138\_x\_at |  | | 202169\_S\_AT | 202169\_s\_at |  | | 202223\_AT | 202223\_at |  | | 202279\_AT | 202279\_at |  | | 202370\_S\_AT | 202370\_s\_at |  | | 202406\_S\_AT | 202406\_s\_at |  | | 202416\_AT | 202416\_at |  | | 202453\_S\_AT | 202453\_s\_at |  | | 202467\_S\_AT | 202467\_s\_at |  | | 202483\_S\_AT | 202483\_s\_at |  | | 202550\_S\_AT | 202550\_s\_at |  | | 202591\_S\_AT | 202591\_s\_at |  | | 202606\_S\_AT | 202606\_s\_at |  | | 202679\_AT | 202679\_at |  | | 202683\_S\_AT | 202683\_s\_at |  | | 202697\_AT | 202697\_at |  | | 202737\_S\_AT | 202737\_s\_at |  | | 202743\_AT | 202743\_at |  | | 202754\_AT | 202754\_at |  | | 202852\_S\_AT | 202852\_s\_at |  | | 202904\_S\_AT | 202904\_s\_at |  | | 202911\_AT | 202911\_at |  | | 203023\_AT | 203023\_at |  | | 203032\_S\_AT | 203032\_s\_at |  | | 203062\_S\_AT | 203062\_s\_at |  | | 203077\_S\_AT | 203077\_s\_at |  | | 203091\_AT | 203091\_at |  | | 203100\_S\_AT | 203100\_s\_at |  | | 203119\_AT | 203119\_at |  | | 203177\_X\_AT | 203177\_x\_at |  | | 203195\_S\_AT | 203195\_s\_at |  | | 203212\_S\_AT | 203212\_s\_at |  | | 203244\_AT | 203244\_at |  | | 203246\_S\_AT | 203246\_s\_at |  | | 203294\_S\_AT | 203294\_s\_at |  | | 203349\_S\_AT | 203349\_s\_at |  | | 203395\_S\_AT | 203395\_s\_at |  | | 203432\_AT | 203432\_at |  | | 203493\_S\_AT | 203493\_s\_at |  | | 203503\_S\_AT | 203503\_s\_at |  | | 203594\_AT | 203594\_at |  | | 203622\_S\_AT | 203622\_s\_at |  | | 203625\_X\_AT | 203625\_x\_at |  | | 203654\_S\_AT | 203654\_s\_at |  | | 203664\_S\_AT | 203664\_s\_at |  | | 203667\_AT | 203667\_at |  | | 203721\_S\_AT | 203721\_s\_at |  | | 203738\_AT | 203738\_at |  | | 203782\_S\_AT | 203782\_s\_at |  | | 203816\_AT | 203816\_at |  | | 203820\_S\_AT | 203820\_s\_at |  | | 203867\_S\_AT | 203867\_s\_at |  | | 203967\_AT | 203967\_at |  | | 204025\_S\_AT | 204025\_s\_at |  | | 204031\_S\_AT | 204031\_s\_at |  | | 204089\_X\_AT | 204089\_x\_at |  | | 204207\_S\_AT | 204207\_s\_at |  | | 204238\_S\_AT | 204238\_s\_at |  | | 204331\_S\_AT | 204331\_s\_at |  | | 204355\_AT | 204355\_at |  | | 204426\_AT | 204426\_at |  | | 204531\_S\_AT | 204531\_s\_at |  | | 204700\_X\_AT | 204700\_x\_at |  | | 204831\_AT | 204831\_at |  | | 204905\_S\_AT | 204905\_s\_at |  | | 204957\_AT | 204957\_at |  | | 205061\_S\_AT | 205061\_s\_at |  | | 205063\_AT | 205063\_at |  | | 205086\_S\_AT | 205086\_s\_at |  | | 205129\_AT | 205129\_at |  | | 205135\_S\_AT | 205135\_s\_at |  | | 205167\_S\_AT | 205167\_s\_at |  | | 205195\_AT | 205195\_at |  | | 205252\_AT | 205252\_at |  | | 205361\_S\_AT | 205361\_s\_at |  | | 205394\_AT | 205394\_at |  | | 205395\_S\_AT | 205395\_s\_at |  | | 205446\_S\_AT | 205446\_s\_at |  | | 205519\_AT | 205519\_at |  | | 205545\_X\_AT | 205545\_x\_at |  | | 205677\_S\_AT | 205677\_s\_at |  | | 205748\_S\_AT | 205748\_s\_at |  | | 206499\_S\_AT | 206499\_s\_at |  | | 207170\_S\_AT | 207170\_s\_at |  | | 207239\_S\_AT | 207239\_s\_at |  | | 207515\_S\_AT | 207515\_s\_at |  | | 207824\_S\_AT | 207824\_s\_at |  | | 207891\_S\_AT | 207891\_s\_at |  | | 208079\_S\_AT | 208079\_s\_at |  | | 208264\_S\_AT | 208264\_s\_at |  | | 208289\_S\_AT | 208289\_s\_at |  | | 208336\_S\_AT | 208336\_s\_at |  | | 208424\_S\_AT | 208424\_s\_at |  | | 208445\_S\_AT | 208445\_s\_at |  | | 208645\_S\_AT | 208645\_s\_at |  | | 208694\_AT | 208694\_at |  | | 208753\_S\_AT | 208753\_s\_at |  | | 208793\_X\_AT | 208793\_x\_at |  | | 208799\_AT | 208799\_at |  | | 208802\_AT | 208802\_at |  | | 208825\_X\_AT | 208825\_x\_at |  | | 208907\_S\_AT | 208907\_s\_at |  | | 208941\_S\_AT | 208941\_s\_at |  | | 208947\_S\_AT | 208947\_s\_at |  | | 208975\_S\_AT | 208975\_s\_at |  | | 208995\_S\_AT | 208995\_s\_at |  | | 209056\_S\_AT | 209056\_s\_at |  | | 209059\_S\_AT | 209059\_s\_at |  | | 209085\_X\_AT | 209085\_x\_at |  | | 209247\_S\_AT | 209247\_s\_at |  | | 209248\_AT | 209248\_at |  | | 209262\_S\_AT | 209262\_s\_at |  | | 209408\_AT | 209408\_at |  | | 209418\_S\_AT | 209418\_s\_at |  | | 209434\_S\_AT | 209434\_s\_at |  | | 209445\_X\_AT | 209445\_x\_at |  | | 209461\_X\_AT | 209461\_x\_at |  | | 209551\_AT | 209551\_at |  | | 209645\_S\_AT | 209645\_s\_at |  | | 209715\_AT | 209715\_at |  | | 209861\_S\_AT | 209861\_s\_at |  | | 209891\_AT | 209891\_at |  | | 210005\_AT | 210005\_at |  | | 210023\_S\_AT | 210023\_s\_at |  | | 210041\_S\_AT | 210041\_s\_at |  | | 210093\_S\_AT | 210093\_s\_at |  | | 210097\_S\_AT | 210097\_s\_at |  | | 210216\_X\_AT | 210216\_x\_at |  | | 210317\_S\_AT | 210317\_s\_at |  | | 210334\_X\_AT | 210334\_x\_at |  | | 210396\_S\_AT | 210396\_s\_at |  | | 210465\_S\_AT | 210465\_s\_at |  | | 210466\_S\_AT | 210466\_s\_at |  | | 210482\_X\_AT | 210482\_x\_at |  | | 210691\_S\_AT | 210691\_s\_at |  | | 210759\_S\_AT | 210759\_s\_at |  | | 210802\_S\_AT | 210802\_s\_at |  | | 210820\_X\_AT | 210820\_x\_at |  | | 211013\_X\_AT | 211013\_x\_at |  | | 211025\_X\_AT | 211025\_x\_at |  | | 211042\_X\_AT | 211042\_x\_at |  | | 211080\_S\_AT | 211080\_s\_at |  | | 211088\_S\_AT | 211088\_s\_at |  | | 211558\_S\_AT | 211558\_s\_at |  | | 211615\_S\_AT | 211615\_s\_at |  | | 211698\_AT | 211698\_at |  | | 211708\_S\_AT | 211708\_s\_at |  | | 211727\_S\_AT | 211727\_s\_at |  | | 211804\_S\_AT | 211804\_s\_at |  | | 211937\_AT | 211937\_at |  | | 211951\_AT | 211951\_at |  | | 211953\_S\_AT | 211953\_s\_at |  | | 212018\_S\_AT | 212018\_s\_at |  | | 212083\_AT | 212083\_at |  | | 212104\_S\_AT | 212104\_s\_at |  | | 212108\_AT | 212108\_at |  | | 212139\_AT | 212139\_at |  | | 212160\_AT | 212160\_at |  | | 212186\_AT | 212186\_at |  | | 212214\_AT | 212214\_at |  | | 212222\_AT | 212222\_at |  | | 212282\_AT | 212282\_at |  | | 212296\_AT | 212296\_at |  | | 212308\_AT | 212308\_at |  | | 212371\_AT | 212371\_at |  | | 212458\_AT | 212458\_at |  | | 212474\_AT | 212474\_at |  | | 212482\_AT | 212482\_at |  | | 212532\_S\_AT | 212532\_s\_at |  | | 212610\_AT | 212610\_at |  | | 212635\_AT | 212635\_at |  | | 212653\_S\_AT | 212653\_s\_at |  | | 212676\_AT | 212676\_at |  | | 212709\_AT | 212709\_at |  | | 212896\_AT | 212896\_at |  | | 212946\_AT | 212946\_at |  | | 212978\_AT | 212978\_at |  | | 212985\_AT | 212985\_at |  | | 213007\_AT | 213007\_at |  | | 213025\_AT | 213025\_at |  | | 213107\_AT | 213107\_at |  | | 213152\_S\_AT | 213152\_s\_at |  | | 213175\_S\_AT | 213175\_s\_at |  | | 213211\_S\_AT | 213211\_s\_at |  | | 213305\_S\_AT | 213305\_s\_at |  | | 213318\_S\_AT | 213318\_s\_at |  | | 213320\_AT | 213320\_at |  | | 213338\_AT | 213338\_at |  | | 213365\_AT | 213365\_at |  | | 213687\_S\_AT | 213687\_s\_at |  | | 213701\_AT | 213701\_at |  | | 213951\_S\_AT | 213951\_s\_at |  | | 214383\_X\_AT | 214383\_x\_at |  | | 214452\_AT | 214452\_at |  | | 214661\_S\_AT | 214661\_s\_at |  | | 214931\_S\_AT | 214931\_s\_at |  | | 215023\_S\_AT | 215023\_s\_at |  | | 215068\_S\_AT | 215068\_s\_at |  | | 215084\_S\_AT | 215084\_s\_at |  | | 215380\_S\_AT | 215380\_s\_at |  | | 215416\_S\_AT | 215416\_s\_at |  | | 215509\_S\_AT | 215509\_s\_at |  | | 215708\_S\_AT | 215708\_s\_at |  | | 215728\_S\_AT | 215728\_s\_at |  | | 215991\_S\_AT | 215991\_s\_at |  | | 216242\_X\_AT | 216242\_x\_at |  | | 216295\_S\_AT | 216295\_s\_at |  | | 216305\_S\_AT | 216305\_s\_at |  | | 216326\_S\_AT | 216326\_s\_at |  | | 216422\_AT | 216422\_at |  | | 216574\_S\_AT | 216574\_s\_at |  | | 216863\_S\_AT | 216863\_s\_at |  | | 216969\_S\_AT | 216969\_s\_at |  | | 217185\_S\_AT | 217185\_s\_at |  | | 217336\_AT | 217336\_at |  | | 217353\_AT | 217353\_at |  | | 217457\_S\_AT | 217457\_s\_at |  | | 217640\_X\_AT | 217640\_x\_at |  | | 217720\_AT | 217720\_at |  | | 217786\_AT | 217786\_at |  | | 217805\_AT | 217805\_at |  | | 217841\_S\_AT | 217841\_s\_at |  | | 217851\_S\_AT | 217851\_s\_at |  | | 217926\_AT | 217926\_at |  | | 217935\_S\_AT | 217935\_s\_at |  | | 217954\_S\_AT | 217954\_s\_at |  | | 217968\_AT | 217968\_at |  | | 217972\_AT | 217972\_at |  | | 217987\_AT | 217987\_at |  | | 218046\_S\_AT | 218046\_s\_at |  | | 218047\_AT | 218047\_at |  | | 218074\_AT | 218074\_at |  | | 218085\_AT | 218085\_at |  | | 218097\_S\_AT | 218097\_s\_at |  | | 218195\_AT | 218195\_at |  | | 218213\_S\_AT | 218213\_s\_at |  | | 218225\_AT | 218225\_at |  | | 218256\_S\_AT | 218256\_s\_at |  | | 218258\_AT | 218258\_at |  | | 218314\_S\_AT | 218314\_s\_at |  | | 218393\_S\_AT | 218393\_s\_at |  | | 218398\_AT | 218398\_at |  | | 218438\_S\_AT | 218438\_s\_at |  | | 218504\_AT | 218504\_at |  | | 218513\_AT | 218513\_at |  | | 218538\_S\_AT | 218538\_s\_at |  | | 218561\_S\_AT | 218561\_s\_at |  | | 218577\_AT | 218577\_at |  | | 218594\_AT | 218594\_at |  | | 218602\_S\_AT | 218602\_s\_at |  | | 218645\_AT | 218645\_at |  | | 218646\_AT | 218646\_at |  | | 218663\_AT | 218663\_at |  | | 218712\_AT | 218712\_at |  | | 218719\_S\_AT | 218719\_s\_at |  | | 218772\_X\_AT | 218772\_x\_at |  | | 218842\_AT | 218842\_at |  | | 218884\_S\_AT | 218884\_s\_at |  | | 218936\_S\_AT | 218936\_s\_at |  | | 218949\_S\_AT | 218949\_s\_at |  | | 218957\_S\_AT | 218957\_s\_at |  | | 219000\_S\_AT | 219000\_s\_at |  | | 219006\_AT | 219006\_at |  | | 219037\_AT | 219037\_at |  | | 219122\_S\_AT | 219122\_s\_at |  | | 219166\_AT | 219166\_at |  | | 219182\_AT | 219182\_at |  | | 219188\_S\_AT | 219188\_s\_at |  | | 219220\_X\_AT | 219220\_x\_at |  | | 219240\_S\_AT | 219240\_s\_at |  | | 219258\_AT | 219258\_at |  | | 219294\_AT | 219294\_at |  | | 219418\_AT | 219418\_at |  | | 219479\_AT | 219479\_at |  | | 219494\_AT | 219494\_at |  | | 219530\_AT | 219530\_at |  | | 219531\_AT | 219531\_at |  | | 219555\_S\_AT | 219555\_s\_at |  | | 219979\_S\_AT | 219979\_s\_at |  | | 220011\_AT | 220011\_at |  | | 220060\_S\_AT | 220060\_s\_at |  | | 220083\_X\_AT | 220083\_x\_at |  | | 220147\_S\_AT | 220147\_s\_at |  | | 220239\_AT | 220239\_at |  | | 220346\_AT | 220346\_at |  | | 220587\_S\_AT | 220587\_s\_at |  | | 220642\_X\_AT | 220642\_x\_at |  | | 220647\_S\_AT | 220647\_s\_at |  | | 220773\_S\_AT | 220773\_s\_at |  | | 220934\_S\_AT | 220934\_s\_at |  | | 220942\_X\_AT | 220942\_x\_at |  | | 221069\_S\_AT | 221069\_s\_at |  | | 221079\_S\_AT | 221079\_s\_at |  | | 221090\_S\_AT | 221090\_s\_at |  | | 221214\_S\_AT | 221214\_s\_at |  | | 221488\_S\_AT | 221488\_s\_at |  | | 221510\_S\_AT | 221510\_s\_at |  | | 221514\_AT | 221514\_at |  | | 221531\_AT | 221531\_at |  | | 221580\_S\_AT | 221580\_s\_at |  | | 221586\_S\_AT | 221586\_s\_at |  | | 221587\_S\_AT | 221587\_s\_at |  | | 221598\_S\_AT | 221598\_s\_at |  | | 221620\_S\_AT | 221620\_s\_at |  | | 221622\_S\_AT | 221622\_s\_at |  | | 221652\_S\_AT | 221652\_s\_at |  | | 221685\_S\_AT | 221685\_s\_at |  | | 221777\_AT | 221777\_at |  | | 221851\_AT | 221851\_at |  | | 221923\_S\_AT | 221923\_s\_at |  | | 222029\_X\_AT | 222029\_x\_at |  | | 222163\_S\_AT | 222163\_s\_at |  | | 222209\_S\_AT | 222209\_s\_at |  | | 222231\_S\_AT | 222231\_s\_at |  | | 40148\_AT | 40148\_at |  | | 43977\_AT | 43977\_at |  | | 49485\_AT | 49485\_at |  | | 52285\_F\_AT | 52285\_f\_at |  | | 91816\_F\_AT | 91816\_f\_at |  | | AARS | 201000\_at | GO:0006412 GO:0006419 GO:0008033 | | AATF | 209165\_at |  | | ABCD3 | 202850\_at | GO:0006810 GO:0007031 GO:0015910 | | ABCF1 | 200045\_at | GO:0006412 GO:0006954 | | ABT1 | 218405\_at | GO:0006366 | | ACAT2 | 209608\_s\_at | GO:0006629 | | ACN9 | 218981\_at | GO:0006094 | | ACP1 | 201629\_s\_at | GO:0006470 | | ACTL6A | 202666\_s\_at | GO:0001558 GO:0006338 GO:0006350 GO:0006355 GO:0007165 GO:0009613 | | ACTR6 | 218395\_at |  | | ACVR2B | 220028\_at | GO:0006468 GO:0007178 | | ACY1 | 202740\_at | GO:0006508 GO:0006520 | | ADH5 | 208847\_s\_at | GO:0006069 | | ADSL | 210250\_x\_at | GO:0009152 | | AFG3L2 | 202486\_at | GO:0006508 GO:0030163 | | AHCY | 200903\_s\_at | GO:0006730 | | ALDOC | 202022\_at | GO:0006000 GO:0006096 | | ALG3 | 207396\_s\_at | GO:0006486 | | ANAPC1 | 218575\_at | GO:0000074 GO:0006512 GO:0007049 GO:0007067 GO:0051301 | | ANAPC10 | 207845\_s\_at | GO:0000074 GO:0000086 GO:0000090 GO:0006511 GO:0006512 GO:0007049 GO:0030071 GO:0051301 | | ANKHD1 | 208773\_s\_at |  | | AOF2 | 212348\_s\_at | GO:0006118 GO:0006350 GO:0006355 GO:0016568 | | AP3M2 | 203410\_at | GO:0006886 | | APH1A | 218389\_s\_at | GO:0006509 GO:0007220 GO:0016485 GO:0031293 GO:0042987 GO:0043085 | | APIP | 218698\_at |  | | APPBP1 | 202268\_s\_at | GO:0006260 GO:0006512 GO:0007049 GO:0007165 GO:0031574 GO:0042981 GO:0045116 | | ARD1A | 203025\_at | GO:0006323 GO:0006474 GO:0006475 GO:0007001 | | ARL1 | 201657\_at | GO:0006364 GO:0007264 | | ARL2 | 202564\_x\_at | GO:0006364 GO:0007021 GO:0007264 | | ARL6IP4 | 218216\_x\_at |  | | ARNTL2 | 220658\_s\_at | GO:0006355 GO:0007165 | | ASCC1 | 219336\_s\_at | GO:0006355 | | ASS | 207076\_s\_at | GO:0000050 GO:0006526 GO:0008652 | | ATAD3A | 219068\_x\_at |  | | ATIC | 208758\_at | GO:0006139 GO:0006164 | | ATP5B | 201322\_at | GO:0006091 GO:0006811 GO:0015986 GO:0015992 | | ATP5F1 | 211755\_s\_at | GO:0006811 GO:0015986 GO:0015992 | | ATP5G2 | 208764\_s\_at | GO:0006811 GO:0015986 GO:0015992 | | ATP5G3 | 207507\_s\_at | GO:0006091 GO:0006811 GO:0015986 GO:0015992 | | ATP5J | 202325\_s\_at | GO:0006091 GO:0006811 GO:0015986 GO:0015992 | | ATP5J2 | 202961\_s\_at | GO:0006754 GO:0006811 GO:0015992 | | ATPBD1B | 218799\_at |  | | ATPBD1C | 218461\_at |  | | AUP1 | 220525\_s\_at |  | | AURKAIP1 | 218580\_x\_at | GO:0045839 GO:0045862 | | BAG2 | 209406\_at | GO:0006457 GO:0006915 GO:0019538 | | BBS7 | 219688\_at | GO:0007601 GO:0050896 | | BCOR | 219433\_at | GO:0006350 GO:0016568 GO:0045892 | | BET1 | 202710\_at | GO:0006888 GO:0015031 | | BLMH | 202179\_at | GO:0006508 | | BMPR1A | 213578\_at | GO:0006468 GO:0007179 | | BNIP1 | 37226\_at | GO:0006915 GO:0006916 | | BRRN1 | 212949\_at | GO:0000278 GO:0007067 GO:0007076 GO:0051301 | | BXDC2 | 219177\_at | GO:0007046 | | BYSL | 203612\_at | GO:0007155 GO:0007565 | | C10ORF70 | 218597\_s\_at |  | | C13ORF24 | 213239\_at |  | | C15ORF15 | 217915\_s\_at | GO:0006412 GO:0007046 | | C17ORF42 | 219146\_at |  | | C17ORF75 | 203830\_at | GO:0007283 | | C1D | 200056\_s\_at |  | | C1ORF103 | 220235\_s\_at |  | | C1ORF112 | 220840\_s\_at |  | | C1ORF160 | 221512\_at |  | | C1ORF163 | 219420\_s\_at |  | | C1ORF33 | 220688\_s\_at | GO:0007046 | | C1ORF73 | 222250\_s\_at |  | | C1ORF77 | 209927\_s\_at |  | | C20ORF18 | 221827\_at | GO:0006512 | | C20ORF20 | 218586\_at | GO:0001558 GO:0006355 GO:0016568 | | C2ORF25 | 217883\_at |  | | C6ORF75 | 218877\_s\_at | GO:0006306 | | CAND1 | 208838\_at | GO:0006350 GO:0006355 GO:0016567 GO:0043086 | | CAPN7 | 203356\_at | GO:0006508 | | CBR4 | 213626\_at | GO:0008152 | | CBX3 | 201091\_s\_at | GO:0006333 GO:0006350 GO:0006355 GO:0016568 | | CCNB1IP1 | 217988\_at | GO:0006512 | | CCNH | 204093\_at | GO:0000079 GO:0006281 GO:0006350 GO:0006355 GO:0007049 | | CCT3 | 200910\_at | GO:0006457 | | CCT4 | 200877\_at | GO:0000074 GO:0006457 | | CCT5 | 208696\_at | GO:0006457 | | CD320 | 218529\_at | GO:0001558 | | CDC2 | 203213\_at | GO:0000074 GO:0006468 GO:0007049 GO:0007067 GO:0007089 GO:0051301 | | CDC20 | 202870\_s\_at | GO:0000074 GO:0006511 GO:0006512 GO:0007049 GO:0007067 GO:0051301 | | CDC25A | 204695\_at | GO:0000079 GO:0006470 GO:0007049 GO:0007067 GO:0008283 GO:0051301 | | CDC37 | 209953\_s\_at | GO:0000079 GO:0006457 GO:0006605 | | CDC45L | 204126\_s\_at | GO:0000074 GO:0000076 GO:0006260 GO:0006270 GO:0007049 | | CDC7 | 204510\_at | GO:0000082 GO:0006270 GO:0006468 GO:0007049 GO:0007089 GO:0008285 GO:0051301 | | CDCA8 | 221520\_s\_at | GO:0051301 | | CDK5RAP1 | 218315\_s\_at | GO:0007420 GO:0008283 GO:0045664 GO:0045736 | | CDK7 | 211297\_s\_at | GO:0000079 GO:0006281 GO:0006350 GO:0006367 GO:0006468 GO:0007049 GO:0008283 GO:0030521 GO:0045893 GO:0051301 | | CDK9 | 203198\_at | GO:0000074 GO:0006350 GO:0006355 GO:0006367 GO:0006368 GO:0006468 GO:0008283 | | CDT1 | 209832\_s\_at | GO:0000076 GO:0006260 GO:0007049 GO:0030174 | | CEBPZ | 203341\_at | GO:0006350 GO:0006355 GO:0006366 | | CHAF1B | 204775\_at | GO:0006260 GO:0006281 GO:0006335 GO:0006350 GO:0006355 GO:0006461 GO:0007049 | | CHEK2 | 210416\_s\_at | GO:0000077 GO:0006468 GO:0006974 GO:0007049 | | CHST5 | 64900\_at | GO:0005975 GO:0006044 GO:0006790 | | CKS1B | 201897\_s\_at | GO:0007049 GO:0051301 | | CLPP | 202799\_at | GO:0006508 | | CLPX | 204809\_at | GO:0006457 GO:0015031 | | COG5 | 203629\_s\_at | GO:0006891 GO:0015031 | | COPE | 201264\_at | GO:0006886 GO:0006890 GO:0006891 | | COPS6 | 201405\_s\_at |  | | COPS7B | 219997\_s\_at |  | | COQ3 | 221227\_x\_at | GO:0006744 | | COX10 | 203858\_s\_at | GO:0006783 | | COX15 | 221550\_at | GO:0006461 GO:0007585 | | COX5A | 203663\_s\_at | GO:0006118 | | COX6A1 | 200925\_at | GO:0006118 | | COX7A2 | 201597\_at | GO:0006118 | | COX7B | 202110\_at | GO:0006118 | | COX8A | 201119\_s\_at | GO:0006118 | | CRY1 | 209674\_at | GO:0006281 GO:0007601 GO:0007623 | | CS | 208660\_at | GO:0006099 | | CSNK2A1 | 212072\_s\_at | GO:0006468 GO:0016055 | | CSNK2A2 | 203575\_at | GO:0006468 GO:0007165 GO:0007286 GO:0016055 | | CSNK2B | 201390\_s\_at | GO:0016055 | | CSPG6 | 209259\_s\_at | GO:0006281 GO:0007001 GO:0007049 GO:0007052 GO:0007062 GO:0007126 GO:0007165 GO:0009294 GO:0051301 | | CSTF1 | 202190\_at | GO:0006378 GO:0006379 | | CSTF2 | 204459\_at | GO:0006378 GO:0006379 | | CTNNAL1 | 202468\_s\_at | GO:0006915 GO:0007155 | | CTPS | 202613\_at | GO:0006139 GO:0006221 GO:0006541 GO:0042493 | | CYCS | 208905\_at | GO:0006118 GO:0006309 GO:0006915 GO:0008635 GO:0045333 | | CYP51A1 | 202314\_at | GO:0006118 GO:0006695 | | DAP3 | 208822\_s\_at | GO:0006915 GO:0008624 | | DAXX | 201763\_s\_at | GO:0006350 GO:0006915 GO:0007257 GO:0008625 GO:0030521 GO:0045892 | | DAZAP1 | 218443\_s\_at | GO:0007283 GO:0030154 | | DBF4 | 204244\_s\_at |  | | DBN1 | 217025\_s\_at | GO:0007015 GO:0007399 GO:0030154 GO:0048168 GO:0050773 | | DCLRE1A | 209804\_at | GO:0006281 GO:0007049 GO:0007067 GO:0051301 | | DCTN3 | 204246\_s\_at | GO:0000910 GO:0007067 | | DDX10 | 204977\_at |  | | DDX18 | 208897\_s\_at |  | | DDX19B | 202576\_s\_at | GO:0006406 | | DDX21 | 208152\_s\_at |  | | DDX39 | 201584\_s\_at | GO:0000398 GO:0006406 | | DDX47 | 220890\_s\_at | GO:0016070 | | DDX49 | 31807\_at |  | | DDX50 | 221699\_s\_at |  | | DDX52 | 210320\_s\_at |  | | DDX56 | 217754\_at | GO:0006364 | | DEK | 200934\_at | GO:0006357 GO:0006614 GO:0007165 GO:0019079 | | DENR | 221509\_at | GO:0000004 GO:0006413 | | DHCR24 | 200862\_at | GO:0006118 GO:0006695 GO:0006915 GO:0006916 GO:0006979 GO:0007050 GO:0009888 GO:0043154 GO:0043526 GO:0043588 | | DHX16 | 203694\_s\_at | GO:0000074 GO:0000398 GO:0008380 | | DIABLO | 219350\_s\_at | GO:0006915 GO:0006917 GO:0008625 GO:0008635 | | DKFZP779L1558 | 218545\_at | GO:0015031 | | DLAT | 213149\_at | GO:0006085 GO:0006096 GO:0008152 | | DLD | 209095\_at | GO:0006118 | | DNA2L | 213647\_at |  | | DNAJA2 | 209157\_at | GO:0000074 GO:0000080 GO:0006457 GO:0008284 | | DNAJA3 | 205963\_s\_at | GO:0006457 GO:0042981 | | DNAJC12 | 218976\_at | GO:0006457 | | DOK4 | 214263\_x\_at |  | | DOM3Z | 38157\_at |  | | DONSON | 221677\_s\_at | GO:0000004 | | DPAGT1 | 209509\_s\_at | GO:0006629 | | DRG1 | 202810\_at | GO:0006350 GO:0007275 | | DSCR2 | 203405\_at |  | | DSG2 | 217901\_at | GO:0007155 GO:0007156 | | DUS4L | 205761\_s\_at | GO:0008033 | | DUSP14 | 203367\_at | GO:0006470 | | E2F6 | 203957\_at | GO:0000074 GO:0000122 GO:0006350 GO:0006355 GO:0007049 | | EFTUD1 | 218973\_at | GO:0006412 | | EIF2B3 | 218488\_at | GO:0006412 GO:0006413 GO:0009058 GO:0009615 | | EIF2S2 | 208726\_s\_at | GO:0006412 GO:0006413 | | EIF2S3 | 205321\_at | GO:0006412 | | EIF3S2 | 208756\_at | GO:0006412 GO:0006446 | | EIF3S4 | 208887\_at | GO:0006412 GO:0006446 | | EIF4E | 201436\_at | GO:0006413 GO:0006445 | | EIF4EBP1 | 221539\_at | GO:0006445 GO:0017148 GO:0045947 | | ENDOG | 204824\_at | GO:0006259 | | ENO1 | 201231\_s\_at | GO:0006096 GO:0006350 GO:0006355 | | ERAL1 | 212087\_s\_at | GO:0000004 | | ERH | 200043\_at | GO:0000074 GO:0006139 GO:0006213 | | ERP29 | 201216\_at | GO:0006457 GO:0006886 | | ETV4 | 211603\_s\_at | GO:0006350 GO:0006355 | | EXO1 | 204603\_at | GO:0006284 GO:0006289 GO:0006298 GO:0006310 GO:0006955 GO:0007126 | | EXOSC4 | 218695\_at | GO:0006364 | | EXOSC5 | 218481\_at | GO:0006364 | | EXOSC8 | 215136\_s\_at | GO:0000004 GO:0006364 | | EXOSC9 | 213226\_at | GO:0006364 GO:0006955 | | EZH2 | 203358\_s\_at | GO:0006325 GO:0006350 GO:0006355 | | FADS1 | 208964\_s\_at | GO:0006636 GO:0007267 GO:0008654 GO:0009267 GO:0045449 GO:0045595 GO:0046456 | | FAF1 | 218080\_x\_at | GO:0006915 GO:0007253 GO:0008219 GO:0031334 GO:0042176 GO:0043065 GO:0043161 | | FANCE | 220255\_at | GO:0006281 | | FANCL | 218397\_at | GO:0006281 GO:0006512 | | FASN | 212218\_s\_at | GO:0006633 | | FAU | 200019\_s\_at | GO:0000004 GO:0006464 | | FKBP4 | 200894\_s\_at | GO:0006457 | | FLJ10292 | 218894\_s\_at | GO:0000398 GO:0006406 GO:0006810 GO:0007530 | | FLJ11806 | 204216\_s\_at |  | | FLJ20397 | 218460\_at |  | | FLJ21820 | 222192\_s\_at |  | | FLJ21839 | 218480\_at | GO:0006508 | | FLJ21945 | 219120\_at |  | | FRG1 | 204145\_at | GO:0006364 | | G10 | 205690\_s\_at | GO:0006357 | | GADD45GIP1 | 212891\_s\_at |  | | GARS | 208693\_s\_at | GO:0006412 GO:0006426 | | GEMIN6 | 219539\_at | GO:0000245 GO:0000387 | | GFPT1 | 202721\_s\_at | GO:0005975 GO:0006002 GO:0006112 GO:0006541 GO:0008152 GO:0016051 | | GLMN | 207153\_s\_at | GO:0001570 GO:0040029 GO:0042130 GO:0042327 GO:0042692 GO:0045086 GO:0050715 | | GLO1 | 200681\_at | GO:0005975 GO:0006916 | | GLRX2 | 219933\_at | GO:0006118 GO:0006467 GO:0006749 GO:0006915 GO:0006980 GO:0009266 GO:0009268 GO:0009966 GO:0010033 GO:0030154 GO:0030503 GO:0042262 GO:0042542 GO:0045449 | | GMNN | 218350\_s\_at | GO:0007049 GO:0008156 GO:0045786 | | GMPS | 214431\_at | GO:0006164 GO:0006177 GO:0006541 GO:0009058 GO:0009113 | | GNL2 | 201948\_at | GO:0000004 GO:0007046 | | GORASP2 | 208843\_s\_at |  | | GOSR1 | 213021\_at | GO:0006886 GO:0006888 GO:0006891 | | GOT1 | 208813\_at | GO:0006520 GO:0006533 GO:0009058 | | GOT2 | 200708\_at | GO:0006520 GO:0006533 GO:0009058 | | GPR68 | 211249\_at | GO:0006954 GO:0007165 GO:0007186 | | GPS1 | 217782\_s\_at | GO:0000188 GO:0007049 GO:0007254 | | GPX4 | 201106\_at | GO:0006644 GO:0006979 GO:0007275 | | GTF2F2 | 209595\_at | GO:0006350 GO:0006355 GO:0006367 GO:0006368 | | GTF2H4 | 203577\_at | GO:0006281 GO:0006350 GO:0006355 GO:0006366 | | GTF3C2 | 210620\_s\_at | GO:0006350 GO:0006383 | | GTF3C3 | 218343\_s\_at | GO:0006350 GO:0006383 | | GTPBP8 | 221046\_s\_at |  | | H2AFZ | 213911\_s\_at | GO:0006334 GO:0007001 | | HADHSC | 211569\_s\_at | GO:0006629 GO:0006631 | | HAX1 | 201145\_at |  | | HBS1L | 209314\_s\_at | GO:0006412 GO:0007165 | | HCAP-D3 | 212789\_at |  | | HDAC1 | 201209\_at | GO:0006350 GO:0006355 GO:0006916 GO:0016568 GO:0016575 | | HDAC2 | 201833\_at | GO:0006350 GO:0006355 GO:0016568 GO:0016575 | | HDDC2 | 203259\_s\_at |  | | HIRA | 217427\_s\_at | GO:0006357 GO:0009653 | | HMG20A | 218152\_at | GO:0006325 GO:0006355 | | HMGA1 | 210457\_x\_at | GO:0006350 GO:0006355 GO:0007001 GO:0007169 | | HMGCS1 | 221750\_at | GO:0006084 GO:0006629 GO:0006695 | | HMMR | 207165\_at | GO:0006928 | | HNRPC | 212626\_x\_at | GO:0000398 GO:0008380 | | HNRPD | 221480\_at | GO:0000723 GO:0006350 GO:0006355 GO:0006396 GO:0006401 | | HOMER1 | 213793\_s\_at | GO:0007206 GO:0007268 | | HOXD13 | 207397\_s\_at | GO:0001501 GO:0006355 GO:0006366 GO:0007275 | | HS2ST1 | 203284\_s\_at |  | | HSF2 | 211220\_s\_at | GO:0006350 GO:0006355 GO:0006366 GO:0006457 GO:0006986 | | HSPA4L | 205543\_at | GO:0006457 GO:0006986 | | HSPC152 | 217774\_s\_at | GO:0000004 | | HSPD1 | 200806\_s\_at | GO:0006457 GO:0006986 GO:0030150 GO:0042981 | | HSPE1 | 205133\_s\_at | GO:0006457 GO:0006919 GO:0006986 | | IARS | 204744\_s\_at | GO:0006412 GO:0006428 | | IARS2 | 217900\_at | GO:0006428 | | ICMT | 201609\_x\_at | GO:0006464 GO:0006481 GO:0006612 | | ICT1 | 204868\_at | GO:0000004 GO:0006415 | | IDH3B | 201509\_at | GO:0005975 GO:0006099 GO:0006102 GO:0008152 | | IGBP1 | 202105\_at | GO:0009607 GO:0009966 GO:0042113 | | ILF2 | 200052\_s\_at | GO:0006350 GO:0045893 | | ILKAP | 221548\_s\_at | GO:0006470 GO:0007229 | | IMMT | 200955\_at | GO:0000004 | | IMP4 | 212411\_at | GO:0006364 | | IQCB1 | 211707\_s\_at |  | | IQWD1 | 213129\_s\_at |  | | ISOC2 | 218893\_at | GO:0008152 | | ITGB3BP | 205176\_s\_at | GO:0006350 GO:0006355 GO:0006915 GO:0007155 GO:0007165 | | ITGB4BP | 210213\_s\_at | GO:0006412 GO:0006413 | | KDELR1 | 200922\_at | GO:0006621 GO:0006886 GO:0006888 | | KDELR2 | 200698\_at | GO:0006621 GO:0006886 GO:0006888 | | KIAA0286 | 212619\_at | GO:0000004 | | KIAA0971 | 216996\_s\_at | GO:0006915 | | KIF18A | 221258\_s\_at | GO:0007018 GO:0015031 | | KNTC2 | 204162\_at | GO:0000070 GO:0007049 GO:0007051 GO:0048015 | | KRIT1 | 216713\_at | GO:0007264 | | LARP4 | 214155\_s\_at |  | | LARS2 | 34764\_at | GO:0006412 GO:0006429 | | LAS1L | 208117\_s\_at |  | | LDHB | 201030\_x\_at | GO:0006100 GO:0019642 | | LIAS | 214045\_at | GO:0009107 | | LIN7C | 221568\_s\_at | GO:0006887 GO:0015031 | | LOC388345 | 209063\_x\_at |  | | LSM16 | 219207\_at |  | | LSM2 | 209449\_at | GO:0000398 | | LSM8 | 219119\_at | GO:0000398 | | LUC7L2 | 217842\_at |  | | MAD1L1 | 214877\_at | GO:0000089 GO:0000090 GO:0000093 GO:0007049 GO:0007093 GO:0051301 | | MAD2L1 | 203362\_s\_at | GO:0007049 GO:0007067 GO:0007093 GO:0051301 | | MAD2L1BP | 203094\_at | GO:0007096 | | MASA | 217956\_s\_at | GO:0008152 | | MCFD2 | 212245\_at | GO:0006888 GO:0015031 | | MCM3 | 201555\_at | GO:0006260 GO:0006270 GO:0006350 GO:0006355 GO:0007049 | | MCM7 | 210983\_s\_at | GO:0006260 GO:0006270 GO:0006350 GO:0006355 GO:0007049 | | MCTS1 | 218163\_at |  | | MDH1 | 200978\_at | GO:0006096 GO:0006099 GO:0006108 | | MDH2 | 213333\_at | GO:0000004 GO:0006096 GO:0006099 GO:0006108 | | MDN1 | 212693\_at | GO:0006457 GO:0043254 | | MEA1 | 218061\_at | GO:0007283 GO:0008584 GO:0030154 | | MEIS2 | 207480\_s\_at | GO:0000122 GO:0006355 | | MELK | 204825\_at | GO:0006468 | | METAP1 | 212673\_at | GO:0006508 | | METTL5 | 221570\_s\_at | GO:0006479 | | MGC5297 | 219200\_at | GO:0006915 | | MIF | 217871\_s\_at | GO:0001516 GO:0006954 GO:0007166 GO:0008283 GO:0043030 GO:0043066 | | MLC1SA | 204173\_at | GO:0007519 GO:0030049 | | MNAT1 | 203565\_s\_at | GO:0000079 GO:0006281 GO:0006350 GO:0006357 GO:0006461 GO:0007049 GO:0008283 | | MOCS2 | 218212\_s\_at | GO:0006777 | | MPHOSPH6 | 203740\_at | GO:0000074 GO:0000087 | | MPP6 | 205429\_s\_at | GO:0006461 | | MRPL11 | 219162\_s\_at | GO:0006412 | | MRPL12 | 203931\_s\_at | GO:0006412 | | MRPL16 | 217980\_s\_at | GO:0006412 | | MRPL19 | 203465\_at | GO:0006412 | | MRPL2 | 218887\_at | GO:0006412 | | MRPL22 | 218339\_at | GO:0006412 | | MRPL23 | 213897\_s\_at | GO:0006412 | | MRPL34 | 221692\_s\_at | GO:0006412 | | MRPL35 | 218890\_x\_at | GO:0006412 | | MRPL39 | 218558\_s\_at | GO:0000004 GO:0006118 | | MRPL42 | 217919\_s\_at | GO:0006412 | | MRPL46 | 219244\_s\_at | GO:0000004 | | MRPL48 | 218281\_at | GO:0006412 | | MRPL49 | 201717\_at | GO:0006412 GO:0006413 | | MRPS10 | 218106\_s\_at | GO:0000004 GO:0006412 | | MRPS15 | 221437\_s\_at | GO:0006412 | | MRPS17 | 218982\_s\_at | GO:0006412 GO:0006810 | | MRPS18A | 221693\_s\_at | GO:0006412 | | MRPS2 | 218001\_at | GO:0006412 | | MRPS27 | 212145\_at |  | | MRPS33 | 218654\_s\_at | GO:0006412 | | MRPS34 | 218112\_at |  | | MRPS35 | 217942\_at | GO:0042769 GO:0043043 | | MSH2 | 209421\_at | GO:0006298 GO:0006301 GO:0007049 GO:0045786 | | MTIF2 | 203095\_at | GO:0006412 GO:0006446 | | MTX2 | 203517\_at | GO:0006839 GO:0015031 | | MUT | 202959\_at | GO:0008152 | | MYB | 215152\_at | GO:0006355 | | MYBL2 | 201710\_at | GO:0000074 GO:0006355 GO:0006366 GO:0006916 GO:0007275 | | MYC | 202431\_s\_at | GO:0006357 GO:0006879 GO:0007050 GO:0008283 GO:0008284 | | NCBP1 | 209520\_s\_at | GO:0006379 GO:0006406 GO:0006810 GO:0008380 GO:0031442 | | NDUFA10 | 217860\_at | GO:0006091 GO:0006139 | | NDUFA13 | 220864\_s\_at | GO:0006606 GO:0006915 GO:0008624 GO:0017148 GO:0030262 GO:0030308 GO:0045892 | | NDUFA4 | 217773\_s\_at |  | | NDUFA7 | 202785\_at | GO:0042773 | | NDUFA8 | 218160\_at |  | | NDUFA9 | 208969\_at | GO:0006814 GO:0009225 | | NDUFB6 | 203613\_s\_at | GO:0006120 | | NDUFB7 | 202839\_s\_at | GO:0006118 | | NDUFS8 | 203189\_s\_at | GO:0006118 GO:0006120 | | NDUFV1 | 208714\_at | GO:0006118 GO:0006120 | | NDUFV2 | 202941\_at | GO:0006120 GO:0007399 | | NEDD8 | 201840\_at | GO:0006508 GO:0006511 GO:0006512 GO:0009653 | | NHP2L1 | 201077\_s\_at | GO:0000074 GO:0000398 GO:0042254 | | NIPSNAP1 | 201708\_s\_at |  | | NKRF | 205004\_at | GO:0006350 GO:0006355 GO:0016481 | | NME1 | 201577\_at | GO:0006183 GO:0006228 GO:0006241 GO:0007049 GO:0008285 GO:0009117 GO:0009142 GO:0042981 GO:0045786 | | NOC2L | 202115\_s\_at |  | | NOC3L | 218889\_at |  | | NOC4L | 218860\_at | GO:0006365 | | NOLA1 | 219110\_at | GO:0006364 | | NONO | 210470\_x\_at | GO:0000398 GO:0006281 GO:0006310 GO:0006350 GO:0006355 GO:0008380 | | NR2F2 | 209120\_at | GO:0006350 GO:0006357 GO:0006629 GO:0007165 | | NSBP1 | 221606\_s\_at | GO:0006350 GO:0006355 | | NSDHL | 209279\_s\_at | GO:0006695 | | NUP107 | 218768\_at | GO:0006406 GO:0015031 | | NUP133 | 202184\_s\_at | GO:0006406 GO:0015031 GO:0050658 | | NUP153 | 202097\_at | GO:0006810 | | NUP155 | 206550\_s\_at | GO:0006810 GO:0006913 | | NUP205 | 212247\_at | GO:0000059 GO:0006810 GO:0006913 | | NUP37 | 218622\_at | GO:0015031 | | NUP43 | 219007\_at |  | | NUP93 | 202188\_at | GO:0006810 | | NUTF2 | 202397\_at | GO:0015031 | | NVL | 207877\_s\_at |  | | OBFC2B | 218903\_s\_at |  | | ODC1 | 200790\_at | GO:0006596 | | ORC1L | 205085\_at | GO:0006260 GO:0006270 | | ORC2L | 204853\_at | GO:0000122 GO:0006260 GO:0006270 | | ORC3L | 210028\_s\_at | GO:0006260 | | P15RS | 218209\_s\_at |  | | PAFAH1B3 | 203228\_at | GO:0007399 GO:0016042 | | PAICS | 201013\_s\_at | GO:0006164 GO:0006189 GO:0009113 | | PAQR3 | 213372\_at |  | | PARG | 205060\_at |  | | PARVB | 216253\_s\_at | GO:0007155 | | PBK | 219148\_at | GO:0006468 GO:0007067 | | PCNP | 217816\_s\_at | GO:0007049 | | PDAP1 | 202290\_at | GO:0007165 GO:0008283 | | PDCD5 | 219275\_at | GO:0006915 GO:0006917 | | PDHX | 203067\_at | GO:0008152 | | PELO | 218472\_s\_at | GO:0007155 GO:0007160 GO:0007229 | | PES1 | 202212\_at | GO:0008283 GO:0009653 | | PHB2 | 201600\_at | GO:0006350 GO:0006355 GO:0016481 | | PHGDH | 201397\_at | GO:0006564 GO:0007420 GO:0008152 GO:0008652 | | PHOSPHO2 | 213610\_s\_at | GO:0008152 | | PITX1 | 208502\_s\_at | GO:0001501 GO:0006355 GO:0009653 | | PLK1 | 202240\_at | GO:0000074 GO:0006468 GO:0007067 GO:0008283 | | PLS1 | 205190\_at |  | | PLXNA1 | 221538\_s\_at | GO:0007275 | | PMPCB | 201682\_at | GO:0006508 | | POLA2 | 204441\_s\_at | GO:0006260 | | POLE2 | 205909\_at | GO:0006260 GO:0006281 | | POLR2F | 209511\_at | GO:0006351 GO:0045449 | | POLR2I | 212955\_s\_at | GO:0006354 GO:0006355 GO:0006366 | | POLR3B | 219459\_at | GO:0006350 | | POLR3K | 218866\_s\_at | GO:0006354 GO:0006355 GO:0006383 | | POP7 | 209482\_at | GO:0008033 | | POT1 | 204353\_s\_at | GO:0000723 GO:0006260 | | PPIE | 210502\_s\_at | GO:0006457 GO:0045449 | | PPIH | 204228\_at | GO:0000398 GO:0006457 GO:0006461 GO:0006608 | | PPM1G | 200913\_at | GO:0006470 GO:0007050 | | PPP1CC | 200726\_at | GO:0005975 GO:0005977 GO:0007049 GO:0051301 | | PPP1R8 | 207830\_s\_at | GO:0000398 GO:0006350 GO:0006355 GO:0006401 | | PPP2R2D | 221772\_s\_at | GO:0007165 | | PPP2R5D | 211159\_s\_at | GO:0007165 GO:0007399 | | PPP3CB | 209817\_at | GO:0000074 GO:0006351 GO:0006470 GO:0007165 | | PRAF1 | 218997\_at | GO:0006350 GO:0009303 | | PRDX4 | 201923\_at | GO:0007252 | | PRIM1 | 205053\_at | GO:0006260 GO:0006269 | | PRKCI | 213518\_at | GO:0006468 GO:0006612 GO:0007010 GO:0007242 GO:0016044 GO:0016192 GO:0045197 GO:0045216 GO:0046903 | | PRKD3 | 218236\_s\_at | GO:0006468 GO:0007205 GO:0007242 | | PRNPIP | 208973\_at |  | | PRPF19 | 203103\_s\_at | GO:0000398 GO:0006281 GO:0016567 | | PRPS1 | 209440\_at | GO:0009116 GO:0009156 GO:0009165 | | PRR3 | 204795\_at |  | | PRSS15 | 209017\_s\_at | GO:0006510 | | PSAT1 | 220892\_s\_at | GO:0006564 GO:0008152 GO:0008615 GO:0008652 | | PSIP1 | 209337\_at | GO:0006350 GO:0006355 | | PSMA5 | 201274\_at | GO:0006511 | | PSMB2 | 200039\_s\_at | GO:0006511 | | PSMB3 | 201400\_at | GO:0006511 | | PSMB7 | 200786\_at | GO:0006511 | | PSMC3 | 201267\_s\_at | GO:0030163 | | PSMC6 | 201699\_at | GO:0006511 GO:0030163 | | PSMD13 | 201232\_s\_at |  | | PSMD3 | 201388\_at |  | | PSMD7 | 201705\_at |  | | PSMF1 | 201052\_s\_at | GO:0006511 | | PSPH | 205194\_at | GO:0006564 GO:0008152 GO:0008652 | | PTD004 | 219293\_s\_at |  | | PTPLAD1 | 217777\_s\_at | GO:0007249 GO:0007257 GO:0007264 | | PTTG1 | 203554\_x\_at | GO:0006281 GO:0006366 GO:0007049 GO:0007059 GO:0007067 GO:0007283 GO:0051276 GO:0051301 | | PUS7 | 218984\_at | GO:0008033 | | PWP1 | 201607\_at | GO:0006350 | | RABEPK | 203150\_at | GO:0006898 GO:0006904 | | RABGGTB | 213704\_at | GO:0006464 GO:0007601 | | RAC3 | 206103\_at | GO:0007264 GO:0030031 GO:0030036 | | RAD23B | 201222\_s\_at | GO:0006289 GO:0006464 | | RAD51C | 209849\_s\_at | GO:0006281 GO:0006310 | | RAN | 200750\_s\_at | GO:0000074 GO:0006259 GO:0006405 GO:0006611 GO:0006886 GO:0007052 GO:0007165 GO:0007264 GO:0030521 GO:0045893 | | RANGAP1 | 212125\_at | GO:0007165 | | RBBP4 | 210371\_s\_at | GO:0006260 GO:0006350 GO:0006355 GO:0007049 GO:0008285 GO:0016568 | | RBBP8 | 203344\_s\_at | GO:0000075 GO:0006281 GO:0006357 | | RBM13 | 211686\_s\_at |  | | RBM19 | 205115\_s\_at |  | | RBM7 | 218379\_at |  | | RBMX | 213762\_x\_at | GO:0000004 | | RDBP | 209219\_at | GO:0000004 GO:0006350 GO:0006355 | | RFC1 | 209084\_s\_at | GO:0006261 GO:0006350 GO:0006355 GO:0007004 | | RFC2 | 203696\_s\_at | GO:0006260 | | RFC3 | 204127\_at | GO:0006260 GO:0006271 | | RFC4 | 204023\_at | GO:0006260 GO:0006271 GO:0006281 GO:0048015 | | RFC5 | 203209\_at | GO:0006260 GO:0006281 | | RFWD3 | 218564\_at |  | | RFXANK | 202758\_s\_at | GO:0006350 GO:0006355 GO:0006366 GO:0006959 | | RGS16 | 209324\_s\_at | GO:0007601 GO:0008277 GO:0009968 | | RNASEH2A | 203022\_at | GO:0006260 GO:0006401 | | RNF8 | 203160\_s\_at | GO:0006512 | | RNU3IP2 | 204133\_at | GO:0006364 | | RP4-691N24.2 | 206102\_at | GO:0006260 | | RPA2 | 201756\_at | GO:0006261 | | RPA3 | 209507\_at | GO:0006260 GO:0006281 | | RPL13A | 200715\_x\_at | GO:0006412 | | RPL18A | 200869\_at | GO:0006412 | | RPL23 | 200888\_s\_at | GO:0006412 GO:0006610 | | RPL26L1 | 218830\_at | GO:0006412 | | RPL28 | 213223\_at | GO:0006412 | | RPL31 | 200963\_x\_at | GO:0006412 | | RPL35 | 200002\_at | GO:0006412 | | RPL36 | 219762\_s\_at | GO:0006412 | | RPL36A | 201406\_at | GO:0000004 GO:0006412 | | RPL39L | 210115\_at | GO:0006412 GO:0007283 | | RPL41 | 201492\_s\_at | GO:0006412 | | RPLP0 | 214167\_s\_at | GO:0006412 GO:0006414 GO:0042254 | | RPP30 | 203436\_at | GO:0008033 | | RPS25 | 200091\_s\_at |  | | RPS27A | 200017\_at | GO:0006464 GO:0007049 GO:0007411 GO:0016567 GO:0030433 GO:0042062 GO:0045941 GO:0048167 | | RPS28 | 208904\_s\_at | GO:0006412 | | RPS7 | 200082\_s\_at | GO:0006412 | | RUVBL1 | 201614\_s\_at | GO:0001558 GO:0006310 GO:0006350 GO:0006357 GO:0007283 GO:0016568 | | RUVBL2 | 201459\_at | GO:0001558 GO:0006281 GO:0006310 GO:0006350 GO:0006355 GO:0006457 GO:0016568 | | RWDD1 | 219598\_s\_at |  | | SARS2 | 218702\_at | GO:0006412 GO:0006434 | | SART1 | 200051\_at | GO:0000398 GO:0007050 GO:0008629 GO:0045585 | | SATB2 | 213435\_at | GO:0006355 | | SC4MOL | 209146\_at | GO:0006631 GO:0008152 GO:0016126 | | SCC-112 | 212138\_at |  | | SEH1L | 221931\_s\_at | GO:0015031 | | SET | 40189\_at | GO:0006260 GO:0006334 GO:0006337 GO:0006913 GO:0035067 | | SF3B2 | 200619\_at | GO:0000398 GO:0008380 | | SF3B5 | 221263\_s\_at | GO:0000398 | | SHFM1 | 202276\_at | GO:0006508 | | SHMT2 | 214095\_at | GO:0006544 GO:0006563 GO:0006730 | | SLC16A1 | 209900\_s\_at | GO:0006810 GO:0015711 GO:0015728 | | SLC19A2 | 209681\_at | GO:0006810 GO:0007605 GO:0015888 | | SLC1A5 | 208916\_at | GO:0006810 GO:0006835 GO:0015804 | | SLC25A1 | 210010\_s\_at | GO:0006810 GO:0006843 | | SLC25A12 | 203339\_at | GO:0006810 | | SLC25A13 | 203775\_at | GO:0006810 | | SLC25A17 | 211754\_s\_at | GO:0006810 GO:0006839 | | SLC25A4 | 202825\_at | GO:0000002 GO:0006091 GO:0006810 GO:0006839 | | SLC25A5 | 200657\_at | GO:0006810 GO:0006839 | | SLC27A2 | 205768\_s\_at | GO:0000038 GO:0006629 GO:0008152 | | SLC27A5 | 219733\_s\_at | GO:0000038 GO:0006629 GO:0008152 | | SLC29A2 | 204717\_s\_at | GO:0006139 GO:0006810 GO:0008283 GO:0015858 | | SLC30A9 | 202614\_at | GO:0000004 GO:0006812 | | SLC35F2 | 218826\_at | GO:0000004 | | SLC39A14 | 212110\_at | GO:0030001 | | SLC3A1 | 212215\_at | GO:0005975 GO:0006520 GO:0006810 GO:0015802 GO:0015811 | | SLC3A2 | 200924\_s\_at | GO:0005975 GO:0006816 GO:0006865 GO:0016049 | | SLC4A1AP | 218682\_s\_at |  | | SLC5A6 | 204087\_s\_at | GO:0006811 GO:0006814 | | SMAD4 | 202527\_s\_at | GO:0006350 GO:0006355 GO:0007183 | | SMARCA3 | 202983\_at | GO:0006350 GO:0006355 GO:0016568 | | SMARCB1 | 212167\_s\_at | GO:0006338 GO:0006350 GO:0006357 GO:0007049 GO:0015074 GO:0045090 GO:0045786 | | SMNDC1 | 200071\_at | GO:0000245 GO:0006915 GO:0006917 GO:0008380 | | SMS | 202043\_s\_at | GO:0006555 GO:0006595 | | SNRPC | 201342\_at | GO:0008380 | | SNRPD1 | 202690\_s\_at | GO:0000398 | | SNRPD2 | 200826\_at | GO:0000398 | | SNRPD3 | 202567\_at | GO:0000398 | | SNRPE | 203316\_s\_at | GO:0000245 | | SNRPF | 203832\_at | GO:0000398 | | SNRPG | 205644\_s\_at | GO:0000245 GO:0008380 | | SORD | 201563\_at | GO:0006060 GO:0007601 | | SRM | 201516\_at | GO:0008295 | | SRP9 | 201273\_s\_at | GO:0006605 GO:0045900 | | SSR3 | 217790\_s\_at | GO:0006613 | | ST13 | 208666\_s\_at | GO:0006457 | | STIL | 205339\_at | GO:0008283 | | STX18 | 218763\_at | GO:0006886 GO:0006888 GO:0006904 | | SUCLA2 | 202930\_s\_at | GO:0006099 GO:0006104 GO:0006781 GO:0008152 | | SUPV3L1 | 212894\_at |  | | SUV39H1 | 218619\_s\_at | GO:0006333 GO:0016568 | | TAF1A | 206613\_s\_at | GO:0006360 GO:0006366 | | TAF4B | 216226\_at | GO:0006350 GO:0006352 GO:0006355 | | TAF6 | 203572\_s\_at | GO:0006350 GO:0006352 GO:0006355 GO:0051090 | | TBL2 | 212685\_s\_at | GO:0000004 | | TBL3 | 209820\_s\_at | GO:0007199 | | TBP | 203135\_at | GO:0006350 GO:0006355 GO:0006367 | | TBRG4 | 220789\_s\_at | GO:0000080 GO:0006915 GO:0007050 GO:0008284 | | TCEA1 | 216241\_s\_at | GO:0006354 GO:0006357 GO:0006952 | | TCERG1 | 202396\_at | GO:0006350 GO:0006355 GO:0006366 | | TDG | 203743\_s\_at | GO:0005975 GO:0006284 | | TEX10 | 218104\_at | GO:0006810 | | TFB1M | 219169\_s\_at | GO:0000154 GO:0006364 | | TGDS | 208249\_s\_at | GO:0009225 | | THAP4 | 220417\_s\_at |  | | THOP1 | 203235\_at | GO:0006508 | | THYN1 | 218491\_s\_at |  | | TIMM10 | 218408\_at | GO:0007605 GO:0015031 GO:0045039 | | TIMM17B | 203342\_at | GO:0006626 | | TIMM44 | 203092\_at | GO:0006626 | | TIMM8A | 205217\_at | GO:0007399 GO:0007605 GO:0015031 GO:0045039 | | TIMM9 | 218316\_at | GO:0007605 GO:0015031 GO:0045039 | | TLK2 | 212997\_s\_at | GO:0001672 GO:0006468 GO:0006974 GO:0007049 GO:0007242 GO:0016568 | | TMEM41B | 212623\_at |  | | TMEM48 | 218073\_s\_at |  | | TMEM5 | 204807\_at |  | | TNFSF5IP1 | 218467\_at |  | | TNPO3 | 214550\_s\_at | GO:0015031 | | TOMM22 | 217960\_s\_at | GO:0006626 GO:0015031 | | TOMM70A | 201512\_s\_at |  | | TOP2A | 201292\_at | GO:0006260 GO:0006265 GO:0006268 GO:0006281 GO:0048015 | | TPI1 | 213011\_s\_at | GO:0006094 GO:0006096 GO:0006098 GO:0006633 GO:0008152 | | TPRKB | 219030\_at |  | | TPX2 | 210052\_s\_at | GO:0007067 GO:0008283 | | TRAP1 | 201391\_at | GO:0000004 GO:0006457 | | TRIB3 | 218145\_at | GO:0006350 GO:0006355 GO:0006468 GO:0006469 GO:0006915 GO:0043405 | | TRIP13 | 204033\_at | GO:0006366 | | TRIP6 | 209129\_at | GO:0008588 GO:0030335 GO:0048041 | | TRIT1 | 218617\_at | GO:0008033 | | TTC18 | 213092\_x\_at |  | | TTK | 204822\_at | GO:0000074 GO:0006468 GO:0007052 GO:0007094 GO:0008284 | | TTLL12 | 216251\_s\_at | GO:0006464 | | TUBB | 209026\_x\_at | GO:0007018 GO:0042267 GO:0051258 | | TUFM | 201113\_at | GO:0006412 GO:0006414 | | TWIST1 | 213943\_at | GO:0000122 GO:0001501 GO:0006355 GO:0007001 GO:0009653 GO:0030154 | | TXNL2 | 209080\_x\_at | GO:0006118 GO:0045454 | | UBA2 | 201177\_s\_at | GO:0006512 | | UBA52 | 221700\_s\_at | GO:0006412 GO:0006464 | | UBE2L3 | 200676\_s\_at | GO:0006511 GO:0006512 | | UBE2M | 203109\_at | GO:0006512 | | UBE2S | 202779\_s\_at | GO:0006512 | | UBL4A | 221746\_at | GO:0006464 | | UCHL3 | 204616\_at | GO:0006511 GO:0006512 | | UCRC | 218190\_s\_at | GO:0006118 GO:0006122 | | UGCGL2 | 218801\_at | GO:0006486 GO:0051084 | | UPF3B | 218757\_s\_at | GO:0000184 GO:0006397 GO:0006406 GO:0006810 | | UQCRC1 | 201903\_at | GO:0006118 GO:0006119 GO:0009060 | | UQCRH | 202233\_s\_at | GO:0006118 GO:0006119 GO:0006122 GO:0009060 | | USP21 | 218367\_x\_at | GO:0006511 GO:0006512 | | USP39 | 217829\_s\_at | GO:0000398 GO:0006511 | | UTP11L | 218235\_s\_at | GO:0006364 GO:0006917 GO:0007399 GO:0048011 | | UTP14A | 221513\_s\_at | GO:0007046 | | VARS | 201796\_s\_at | GO:0006412 GO:0006414 GO:0006438 | | VBP1 | 201472\_at | GO:0006457 | | VDAC2 | 211662\_s\_at | GO:0006820 | | VPS4A | 217913\_at | GO:0015031 GO:0016192 | | WARS2 | 218766\_s\_at | GO:0006412 GO:0006436 | | WBSCR1 | 206621\_s\_at | GO:0006412 GO:0006446 | | WBSCR22 | 207628\_s\_at | GO:0000004 | | WDHD1 | 216228\_s\_at | GO:0006355 | | WDR12 | 218512\_at |  | | WDR3 | 218882\_s\_at |  | | WDR43 | 214662\_at |  | | WDR57 | 215905\_s\_at | GO:0000398 | | WDR58 | 218848\_at |  | | WDR74 | 221712\_s\_at |  | | WEE1 | 212533\_at | GO:0000074 GO:0006468 GO:0007049 GO:0007067 GO:0051301 | | WHSC2 | 34225\_at | GO:0006350 GO:0006355 GO:0007275 | | XAB1 | 209313\_at | GO:0007264 | | XAB2 | 218110\_at | GO:0006281 GO:0006283 GO:0006350 GO:0006396 | | XTP3TPA | 218069\_at |  | | YARS | 212048\_s\_at | GO:0006412 GO:0006437 GO:0006915 GO:0006928 | | YEATS4 | 218911\_at | GO:0001558 GO:0006350 GO:0006355 GO:0016568 | | YRDC | 218647\_s\_at |  | | ZFAND1 | 218919\_at |  | | ZNF24 | 212534\_at | GO:0006350 GO:0006355 GO:0016481 | | ZNF282 | 212892\_at | GO:0006350 GO:0006355 | | ZNF473 | 213124\_at | GO:0006355 | | ZNF593 | 204175\_at | GO:0000122 GO:0006350 GO:0006355 | | ZNHIT3 | 212544\_at | GO:0006355 | | ZWILCH | 218349\_s\_at |  | | ZZZ3 | 212893\_at | GO:0045449 | |
